# Supplementary material for: Understanding what happens to attendees after an NHS Health Check: a realist review
Source: BMJ Open. 2022 Nov 10;12(11):e064237. doi: 10.1136/bmjopen-2022-064237 (PMC9660666; doi:10.1136/bmjopen-2022-064237)
Supplement: Supplementary data [file bmjopen-2022-064237supp001.pdf]

Supplementary File 2

This file provides the full details of the search strategies employed in the review to identify documents containing relevant data.

Searches to locate existing theories (Step 1)

We searched PubMed and Web of Science (Core Collection) using a combination of terms relating to HCs, and terms relating to theory, adopting a slightly modified version of Booth and Carroll's (2015) 'BeHEMoTh' approach. As the HC programme is a specific health context (H) and we were interested in theorisation of all steps in the programme, no individual behaviours (Be) were specified.

PubMed (11th October 2020)

1. "Health Check" OR "Health Checks" (3847)
2. UK OR United Kingdom OR England OR Britain OR British (1790010)
3. model OR models OR modelling OR theor* OR concept* OR framework* (4625627)
4. 1 AND 2 AND 3 (130 hits)

Web of Science (SCI-EXPANDED, SSCI, AHCI, ESCI) (11th October 2020)

1. "Health Check" OR "Health Checks" (Topic) (3474)
2. UK OR "United Kingdom" OR England OR Britain OR British (Topic) (623802)
3. Model OR models OR modelling OR theor* OR concept* OR framework* (Topic) (11284654)
4. 1 AND 2 AND 3 (70 hits)

These searches identified 11 studies utilising 6 theoretical models or frameworks. An additional search for each of these named theories, plus terms relating to the NHSHC, was conducted in Google Scholar. In addition, we also searched Google Scholar for papers relating to the NHSHC that also cited the key citations related to each identified theory. These two steps were conducted in October 2020 and identified a further 8 (5+3) papers.

Searches for evidence on NHSHC (Step 2)

MEDLINE (via Ovid, 11th November 2020)

1. health check*.ti,ab,kw (6084)
2. (NHS or National Health Service or United Kingdom or UK or England or English).ti,ab,kw (370078)
3. exp England/ (107308)
4. 2 or 3 (440075)
5. 1 and 4 (468)

6. limit 5 to (english language and yr="2008-Current") (325)

Embase (via Ovid, 11th November 2020)

1. health check*.ti,ab,kw (8572)
2. (NHS or National Health Service or United Kingdom or UK or England or English).ti,ab,kw (487112)
3. england/ (24573)
4. 2 or 3 (494513)
5. 1 and 4 (521)
6. limit 5 to (english language and yr="2008-Current") (459)

CINAHL (via EbscoHost, 11th November 2020)

1. TX "health check* (2391)
2. TI (NHS OR "National Health Service" OR "United Kingdom" OR UK OR England OR English) OR AB (NHS OR "National Health Service" OR "United Kingdom" OR UK or England OR English) (178298)
3. MH "England" (62863)
4. S2 OR S3
5. S1 AND S4
6. Limiters: Published Date: 20080101-20201231; English Language; Expanders: Apply equivalent subjects (378)

HMIC (via Ovid, 11th November 2020)

1. health check*.mp (598)
2. (NHS or National Health Service or United Kingdom or UK or England or English).mp (118285)
3. exp england/ (26501)
4. exp health authorities in england/ (6680)
5. or/2-4 (135421)
6. 1 and 5 (321)
7. limit 6 to (english language and yr="2008-Current") (191)

Web of Science (SCI-EXPANDED, SSCI; 11th November 2020)

1. TOPIC: ("health check*") (5144)
2. TOPIC: (NHS OR "national health service" OR "united kingdom" OR UK OR England OR English) (522053)
3. #1 AND #2 (358)

4. Refined by: PUBLICATION YEARS: (2020 OR 2012 OR 2019 OR 2011 OR 2018 OR 2010 OR 2017 OR 2009 OR 2016 OR 2008 OR 2015 OR 2014 OR 2013) AND LANGUAGES: (ENGLISH) (309)

Additional searches for theory

A short series of highly focused searches for documents describing street-level bureaucrats or street-level bureaucracy were run in Google Scholar in October and November 2021. The details of these searches and results screened (on screen) are provided below.

Google Scholar, 6th October 2021

("street level bureaucracy" OR "street level bureaucrats") AND ("public health") (8,160)

("street level bureaucracy" OR "street level bureaucrats") AND ("primary care") (2090)

("street level bureaucracy" OR "street level bureaucrats") AND ("general practice" OR “general practices”) (889)

("street level bureaucracy" OR "street level bureaucrats") AND ("local authority" OR “local authorities”) (6320)
