# Supplementary material for: Understanding what happens to attendees after an NHS Health Check: a realist review
Source: BMJ Open. 2022 Nov 10;12(11):e064237. doi: 10.1136/bmjopen-2022-064237 (PMC9660666; doi:10.1136/bmjopen-2022-064237)
Supplement: Supplementary data [file bmjopen-2022-064237supp002.pdf]

Supplementary File 3

This file provides the full details of the documents included in the review.

First author	Year	Document type	Aim or purpose of document	Study design (if applicable)	Perspective(s)	Local area(s)	Commis-sioning period[a]	Contributed data to CMOCs			Total contrib-utions to CMOCs	Source
								C-CMOCs	P-CMOCs	A-CMOCs		
Alageel(1)	2018 (1)	Conference presentation	Summary of results of qualitative interview study (Alageel 2018)	Qualitative interview study	Providers	Lambeth and Lewisham	LA		P7		1	NHSHC website
Alageel(2)	2018 (2)	Journal article	To identify barriers and facilitators to implementing multiple health behaviour change interventions for CVD risk reduction in primary care	Qualitative interview study	Providers	Lambeth and Lewisham	LA	C14	P1, P2, P6, P7, P8, P10, P11, P13, P15, P20, P22, P24, P27	A1, A2, A27, A30, A32, A35, A38, A39	22	Existing review
Alageel(3)	2020	Journal article	To examine factors that might influence engagement and adherence to lifestyle change interventions and medication amongst people recently	Qualitative interview study	Attendees	Lambeth and Lewisham	LA	C14	P23	A2, A6, A10, A12, A15, A25, A28, A30, A31, A32, A35, A36, A38, A43, A44	17	Existing review

			assessed at medium or high risk of CVD									
Alford(4)	2010	Evaluation report	To explore behaviour changes made following community based NHSHC and experience of NHSHCs	Qualitative interview study	Attendees	Knowsley	PCT			A40, A44	2	Existing review
Al-Osaimi(5)	2020	Conference abstract	To describe the implementation of software to manage the NHSHC	n/a	Commissioners	Sandwell	LA	C2, C7			2	NHSHC website
Artac(6)	2013	Journal article	To assess whether the NHSHC was associated with a reduction in CVD risk after one year	Pre/post	Attendees	Hammersmith and Fulham	PCT		P12		1	Existing review
Atkins(7)	2020	Journal article	To identify behaviours and actors relevant to uptake, delivery and follow up of NHSHCs and influences on those behaviours	Systematic review	n/a	n/a	n/a	C14		A35	2	Searches - MEDLINE

Baker(8)	2014 (1)	Evaluation report	To assess the impact of the NHSHC programme and inform future commissioning (summary)	Cross-sectional	Attendees	Gloucestershire	PCT	C9	P12, P17		3	NHSHC website
Baker(9)	2014 (2)	Journal article	To investigate the perceptions and opinions of patients who attended an NHSHC	Survey	Attendees	Gloucestershire	PCT	C14	P1, P8, P20, P25	A1, A15, A18, A22, A23, A26, A34	12	Existing review
Baker(10)	2015 (1)	Journal article	To investigate health professionals' experiences and perspectives of implementation of the NHSHC	Survey including qualitative analysis of free text responses	Providers	Gloucestershire	PCT	C2	P10, P11, P18		4	Existing review
Baker(11)	2015 (2)	Journal article	To investigate how the local NHSHC pathway was followed and interpreted	Cross-sectional	n/a	Gloucestershire	PCT		P2, P12		2	Existing review
Bell(12)	2019	Conference abstract	To describe the outcomes of NHSHCs delivered in an integrated model with lifestyle services	Cross-sectional	Commissioners	Medway	LA	C3	P19		2	NHSHC website

Boase(13)	2012	Journal article	To explore the perspectives of practice nurses in their role of communicating CVD risk to patients	Qualitative interview study	Providers	Cambridge	PCT		P4, P22, P27	A8, A19	5	Searches - MEDLINE
Boseley(14)	2020	Conference presentation	To describe a local model of NHSHC delivery with integrated lifestyle services	Mixed methods	Commissioners	East Sussex	LA	C3, C6, C7	P14, P15, P19	A4	7	NHSHC website
Brutus(15)	2013	Evaluation report	To review the design of the local NHSHC programme and analyse delivery options available	Cross-sectional	Various	Croydon	PCT	C1, C2, C7	P1, P13, P15, P19	A6, A40	9	NHSHC website
Burgess(16)	2016	Conference presentation	Summary results of a qualitative interview study (Alageel 2020)	Qualitative interview study	Attendees	Lambeth and Lewisham	LA		P1, P23	A2, A5, A10, A20, A23, A28, A33, A42	10	NHSHC website
Burke(17)	2015	Conference presentation	To describe local delivery of NHSHCs with a focus on vulnerable groups	n/a	Commissioners	Leeds	LA			A5, A38	2	NHSHC website
Carter(18)	2016	Journal article	To evaluate local outcomes of NHSHCs with a focus on diagnosis and management after checks	Retrospective cohort	Attendees	Leicester	PCT, Transition	C1			1	Existing review

Centre for Public Scrutiny(19)	2014	Other report	A report on the role of Council Scrutiny in local reviews of NHSHC programmes	n/a	n/a	Various	LA	C1, C11	P1		3	Searches - HMIC
Chatterjee(20)	2017	Journal article	To assess the knowledge, use, and confidence in national physical activity and Chief Medical Officer guidelines and tools among GPs in England	Survey	Providers	England	LA		P21		1	Searches - MEDLINE
Chipchase(21)	2011	Evaluation report	To explore people's views and experiences of the NHSHC	Qualitative interview study	Various	Birmingham	PCT		P1, P14	A1, A2, A11, A12, A19, A20, A40	9	Existing review
Clarke(22)	2020	Conference presentation	To describe the of data to inform local CVD prevention work	n/a	Commissioners	Barnsley	LA	C8		A33	2	NHSHC website
Cochrane(23)	2012 (1)	Journal article	To compare changes in population CVD risk factors between those who receive an NHSHC and those who receive an NHSHC with additional	RCT	n/a	Stoke on Trent	PCT		P26	A9	2	Existing review

			lifestyle support									
Cochrane(24)	2013	Journal article	To review response, attendance and treatment uptake following NHSHCs	Cross-sectional	n/a	Stoke on Trent	PCT			A9	1	Existing review
Coffey(25)	2014	Evaluation report	To assess the level of uptake of NHSHCs	Cross-sectional	n/a	Salford	PCT		P12, P20		2	Existing review
Coghill(26)	2018	Journal article	To assess uptake and outcomes of NHSHCs in general practice	Cross-sectional	n/a	Bristol	PCT, Transition, LA		P12, P16		2	Existing review
Collins(27)	2011	Evaluation report	To evaluate the effectiveness of an 'outreach' delivery model for NHSHCs	Mixed methods	n/a	Medway	PCT		P17	A4	2	NHSHC website
Corlett(28)	2016	Journal article	To assess the findings of NHSHCs delivered in pharmacy settings, referrals to GPs and client views	Mixed methods	n/a	Lewisham	Transition, LA			A2, A13, A15, A25, A35, A38	6	Existing review
Coward(29)	2020	Conference presentation	To describe processes linking NHSHCs with	n/a	n/a	Dorset	LA	C2, C7, C8	P17, P19, P26, P27, P28		8	NHSHC website

			local services that aim to promote physical activity									
Cupit(30)	2018	PhD thesis	To explore how patients' and healthcare professionals' knowledge and practices about CVD prevention are socially organised	Ethnography	n/a	Unknown	LA	C8, C13, C14	P2, P4, P9, P10, P20, P24	A11, A23, A25, A27, A31, A35, A38, A44	17	NHSHC website
Dalton(31)	2011	Journal article	To examine the uptake of NHSHCs and statin prescribing	Cross-sectional	n/a	Ealing	PCT		P2, P3		2	Existing review
Derbyshire County Council(32)	2015	Evaluation report (Health Equity Audit)	To identify health inequalities in relation to the NHSHC programme	Cross-sectional	Commissioner	Derbyshire	LA	C9, C10	P2, P12, P15, P16, P17, P20, P22, P25	A35	11	NHSHC website
Edmans(33)	2013	Other report	To describe local training provision in relation to the dementia component of NHSHCs	n/a	Providers	Southwark	LA		P20		1	NHSHC website
Fenton(34)	2018	Conference presentation	To describe local CVD prevention work and NHSHCs	Cross-sectional	n/a	Southwark	LA		P1, P3		2	NHSHC website
Forster(35)	2015	Journal article	To evaluate the 'yield' of NHSHCs (in relation to identification of	Retrospective cohort	n/a	England	PCT, Transition, LA		P2, P6, P12, P16		4	Existing review

			risk factors, diagnoses and prescribing)									
Forsyth(36)	2012	Conference abstract	To test a delivery model using pharmacists to deliver health checks to the South Asian community (NB describes checks delivered in Scotland)	Mixed methods	n/a	Glasgow	PCT			A17	1	Searches - Embase
Frazer(37)	2020	Conference presentation	To describe the delivery of NHSHCs with integrated lifestyle services	n/a	Commissioners	West Sussex	LA	C3			1	NHSHC website
Gidlow(38)	2020	Journal article	To examine the content of NHSHC, patient-practitioner communication balance and differences when using QRISK2 versus JBS3 CVD risk calculators	Observation (video recorded NHSHCs)	n/a	West Midlands	LA		P10, P20	A6, A13, A39	5	Searches - MEDLINE
Gidlow(39)	2021 (1)	Research report	To explore the extent of physical activity and alcohol measurement in the NHSHC	Observation (video recorded NHSHCs)	n/a	West Midlands	LA		P20		1	NHSHC website

Gidlow(40)	2021 (2)	Research report	To explore practitioner and patient understanding of CVD risk, associated advice or treatment and the response of patients to the NHSHC supported by the QRISK2 or JBS3 risk calculators	Mixed methods	n/a	West Midlands	LA		P10	A9, A10, A13, A29	4	Search alerts
Graley(41)	2011	Journal article	To describe a 'postcode lottery' effect in relation to NHSHCs	Cross-sectional	Commissioner	North West London	PCT	C9, C10, C13, C14	P12, P13		6	Existing review
Greaves(42)	2015	Journal article	To assess the feasibility of delivering an intervention to promote healthy eating and physical activity and of conducting a full scale RCT	Pilot RCT	n/a	Bath	PCT		P28	A30, A37, A40	4	Searches - MEDLINE
Green(43)	2018	Evaluation report	To understand the NHSHC patient journey from invitation through to behaviour change or clinical intervention	Mixed methods	Attendees	East Sussex	LA	C7	P10, P24	A1, A8, A12, A24, A29, A35	9	Stakeholder group
Gregory(44)	2018	Conference presentation	To describe the role of healthcare professionals	n/a	n/a	England	LA		P25		1	NHSHC website

			in treatment of tobacco dependency									
Gulliford(45)	2018	Journal article	To compare CVD risk scores for 'invited' and 'opportunistic' NHSHCs	Retrospective cohort	n/a	Lambeth and Lewisham	Transition, LA		P11		1	Existing review
Hardman(46)	2014	Evaluation report	To describe local delivery of an NHSHC programme with Health Trainers	n/a	Various	Bolton	PCT	C3	P7, P14, P19	A13, A30	6	NHSHC website
Haringay Council(47)	2012	Evaluation report	Scrutiny review' focused on men's health needs and life expectancy gap	Mixed methods	n/a	Haringay	PCT	C7		A10, A13, A14, A16, A30, A37	7	NHSHC website
Hawking(48)	2018	Conference poster	To describe a visual risk communication tool designed for use in NHSHCs	n/a	Providers, attendees	Newham	LA			A44	1	NHSHC website
Hawking(49)	2019	Journal article	To explore patient perspectives and experiences of a personalised risk report during an NHSHC	Qualitative interview study	Attendees	Newham	LA		P28	A1, A9, A10, A18, A19, A20, A40, A44	9	Existing review
Hinde(50)	2017	Journal article	To assess the cost-effectiveness of NHSHCs	Economic evaluation	n/a	England	LA	C12, C13			2	Existing review

Homer(51)	2015	Journal article	To determine the characteristics of patients prescribed statins for primary prevention according to their CVD risk	Cross-sectional	n/a	Newham, City & Hackney, Tower Hamlets (London)	LA		P3		1	Serendipity
Honey(52)	2013	Journal article	To identify the attitudes of primary healthcare professionals towards the delivery of lifestyle advice in NHSHCs	Q methodology	Providers	Leeds	PCT		P2, P6, P7, P18, P22, P24, P27		7	Searches - MEDLINE
Honey(53)	2015	Journal article	To examine the perspectives of patients identified as being at 'high risk' of CVD events	Qualitative interview study	Attendees	Leeds	PCT		P27	A2, A3, A4, A5, A7, A9, A10, A13, A19, A20, A22, A27, A29	14	Searches - MEDLINE
Hooper(54)	2014	Journal article	To estimate the case detection of five health conditions by NHSHCs	Cross-sectional	Attendees	Warwickshire	PCT	C10			1	Existing review
Hyseni(55)	2020	Journal article	To report the results of a workshop that aimed to facilitate engagement with stakeholders who will be involved in co-	Qualitative interview study	Commissioners, Providers, Policymakers	Various	LA	C5, C7, C11, C12, C13, C14	P12		7	Searches - MEDLINE

			production of an NHSHC modelling tool for commissioners									
Ismail(56)	2015	Journal article	To explore the challenges and barriers faced by staff involved in the delivery of the NHSHC	Qualitative interview study	Providers	Leeds	LA	C1, C7, C9, C14	P6, P10, P12	A35, A36, A44	10	Existing review
Ismail(57)	2016	Journal article	To provide an insight into the process of patients receiving NHSHCs and determine the extent to which they were supported to reduce CVD risk through behaviour change	Qualitative interview study	Attendees	Leeds	PCT		P2, P27	A2, A6, A8, A11, A12, A30, A31, A38	10	Existing review
Ismail(58)	2019	Research report (NIHR)	To test the clinical and cost-effectiveness of an 'enhanced lifestyle motivational interviewing' intervention for patients at high risk of CVD in group and individual	RCT	n/a	South London	LA		P6, P7	A6, A16, A17, A26, A27, A30, A31, A32, A34, A40, A44	13	Searches - WoS

			settings, compared with usual care									
Jones(59)	2020	Conference poster	To describe local CVD prevention work and NHSHCs	n/a	n/a	Telford	LA / CCG	C1	P6, P17	A14	4	NHSHC website
Kearney(60)	2015	Conference presentation	To describe factors that influence GP engagement with NHSHCs and potential solutions	n/a	n/a	n/a	LA	C4	P1		2	NHSHC website
Kennedy(61)	2019	Journal article	To evaluate uptake, risk factor detection and management from the NHSHC	Quasi-RCT	n/a	Hampshire	PCT, Transition, LA		P13		1	Existing review
Kirkpatrick(62)	2016	Conference presentation	To describe staff development and training for NHSHC providers	n/a	Providers	Salford	LA		P8		1	NHSHC website
Krska(63)	2010	Evaluation report	To evaluate a CVD screening service offered in pharmacy settings and obtain views of prospective users about	Mixed methods	n/a	Sefton	PCT		P25	A2	2	NHSHC website

			the acceptability of the service								
Krska(64)	2013	Conference abstract	To compare the views of the general public, pharmacists and patients invited to the NHSHC and to compare experiences of NHSHCs delivered by pharmacies and general practices	Survey and qualitative interviews	Providers, attendees	Sefton	PCT			A14	1	Searches - Embase
Krska(65)	2014	Journal article	To explore the views and experiences of patients with potentially high-CVD risk	Survey	Attendees	Sefton	PCT		P7, P10	A15	3	Existing review
Krska(66)	2016 (1)	Journal article	To seek the views of GPs and practice managers on NHSHC implementatio n	Survey	Providers	Sefton	PCT	C9	P10, P13		3	Existing review
Krska(67)	2016 (2)	Journal article	To evaluate NHSHC implementatio n in relation to data recording, advice provided, referrals,	Cross-sectional	Providers	Sefton	PCT	C10			1	Existing review

			prescribing and new diagnoses									
Kumar(68)	2015	Conference presentation	To describe work to improve GP engagement with NHSHCs	n/a	n/a	Stoke on Trent, Havering (London)	LA	C1			1	NHSHC website
Lake(69)	2010	Conference presentation	To describe a community based NHSHC service	n/a	n/a	Camden	PCT	C11	P19, P28	A5, A14	4	NHSHC website
Lambert(70)	2016	Journal article	To assess indicators of programme reach available to local service commissioners	Assessment of indicators	n/a	NE England	PCT, Transition		P12		1	Existing review
Lennon(71)	2020	Conference presentation	To describe local responses to Covid-19 in relation to NHSHCs and social prescribing	n/a	n/a	Redbridge	LA	C6			1	Stakeholder group
Liverpool City Council(72)	2020	Conference poster	To describe the local Health Trainer service available to NHSHC attendees	n/a	n/a	Liverpool	LA		P6, P8, P19, P26, P27, P28	A6, A14, A16, A30, A36, A40	12	NHSHC website
Local Government Association(73)	2015	Other report	Report on the transfer of public health responsibilities to LAs	n/a	Commissioners	Various	LA	C1, C5, C8, C11, C12, C13			6	Existing review

Local Authority A(74)[b]	2019	Evaluation report	Evaluation of local NHSHC programme	Mixed methods	Commissioners	[Redacted]	LA	C1, C2, C11	P16, P17	A15	6	Survey respondent
Local Authority B(75)[b]	2019	Evaluation report	Evaluation of local NHSHC programme	Mixed methods	Commissioners	[Redacted]	LA	C7, C11, C12			3	Survey respondent
Local Authority C(76)[b]	nd	Working document	Report describing quality assurance considerations in relation to NHSHCs	n/a	Commissioners	[Redacted]	LA	C1, C2, C4, C7	P9		5	Survey respondent
Local Authority C(77)[b]	2019	Evaluation report	To measure, monitor and report on performance and quality of NHSHCs against a quality framework	Mixed methods	Commissioners	[Redacted]	LA	C2, C7, C10, C12			4	Survey respondent
London Borough of Bromley(78)	2018	Conference presentation	To assess the prevention of diabetes through NHSHCs	Cross-sectional	n/a	Bromley	LA	C14	P8, P25		3	NHSHC website
Loo(79)	2011	Conference abstract	To derive information on community pharmacists' activities and attitudes in relation to NHSHCs	Survey	Providers	Various	PCT		P13		1	Existing review
Lumley(80)	2015	Conference presentation	To describe the role of the NHSHC in addressing high blood pressure	n/a	Commissioners, providers	Blackpool	LA	C1, C7			2	NHSHC website

Maddern(81)	2020	Conference abstract	To describe the role of local political scrutiny in developing and improving NHSHCs	n/a	Commissioners	Wiltshire	LA	C11			1	NHSHC website
Martin(82)	2011	Practitioner article	To describe challenges in delivery of NHSHCs	n/a	Provider	East Sussex	PCT	C13	P14		2	Searches - CINAHL
McDermott(83)	2015	Conference presentation	Summary of research exploring the implementation of NHSHCs in primary care settings	Qualitative interview study	Providers	Lambeth and Lewisham	PCT, Transition	C9	P1, P10, P13		4	NHSHC website
McMillan(84)	2018	Journal article	Report on a workshop with members of the public to inform design of a 'digital adjunct' intervention for the NHSHC	Co-production	Attendees	Manchester	LA			A6, A44	2	Searches - MEDLINE
McNaughton (85)	2011	Journal article	To evaluate delivery of the NHSHC in community pharmacies	Qualitative interview study	Providers	Tees Valley	PCT	C13	P20, P27	A14, A15	5	Existing review
McNaughton (86)	2014	Journal article	To understand factors that influenced adherence to medication and advice in 'high risk' patients identified by the NHSHC	Qualitative interview study	Attendees	North East England	PCT			A3, A9, A10, A25, A27, A28, A35, A38, A40, A42	10	Existing review

Mills(87)	2017	Journal article	To synthesise data concerning the views of commissioners, managers and healthcare professionals towards the NHSHC	Systematic review	Commissioners, providers	n/a	n/a		P10, P13		2	Searches - MEDLINE
Nahar(88)	2020	Journal article	Protocol for a mixed methods implementation study of a community based CVD risk assessment and coaching intervention	Mixed methods (protocol)	n/a	Sussex, Nottingham	LA			A14	1	Searches - MEDLINE
Nasir(89)	2018	Conference abstract	To describe a local project that aimed to increase NHSHC uptake and referrals to local lifestyle services	Cross-sectional	n/a	Kingston (London)	LA			A35, A37	2	NHSHC website
Newman(90)	2018	Conference presentation	To describe Health Coaching techniques	n/a	n/a	n/a	LA		P27		1	NHSHC website
NHS Greenwich(91)	2011	Evaluation report	To assess the outcomes of a community-based NHSHC delivery model	Mixed methods	n/a	Greenwich	PCT		P14	A4, A13, A17	4	Existing review
NHS Midlands and East(92)	2011	Evaluation report	To evaluate a pilot programme aiming to	Mixed methods	n/a	Bedfordshire; Great Yarmouth	PCT	C2, C7	P1, P7, P10, P17, P19	A40	8	NHSHC website

			deliver lifestyle interventions through the NHSHC			and Waveney						
NICE(93)	2014	Guidance	Guidance for local authorities on commissioning and delivery of the NHSHC	n/a	Commissioners	England	LA	C11			1	Searches - HMIC
Nicholas(94)	2012	Journal article	To evaluate the organisation of the NHSHC in general practices	Survey	Providers	Lambeth and Lewisham	PCT		P2		1	Existing review
O'Flaherty(95)	2021	Research report	To develop a model for commissioners to quantify cost-effectiveness and potential for equitable population health again of the NHSHC	Mixed methods	Commissioners	Various	LA	C7			1	Search alerts
Onyia(96)	2016	Conference presentation	To describe the impact of Health Trainers on uptake of lifestyle services after NHSHCs	Cross-sectional	n/a	Halton	LA		P6, P8, P25, P26		4	NHSHC website
Oswald(97)	2010	Evaluation report	To evaluate the implementation ad outcomes of the 'Tees Vascular Assessment Programme'	Mixed methods	n/a	Tees Valley	PCT	C8, C9, C13, C14	P1, P2, P7, P11, P14, P28	A1, A2, A4, A9, A13, A10, A18, A28, A29, A31, A32, A33, A40, A41, A42, A43	26	Existing review

			(precursor to NHSHC)									
Palladino(98)	2020	Journal article	To assess associations between coverage of the NHSHC and detection and management of incident cases of non-diabetic hyperglycaemia and type 2 diabetes	Retrospective cohort	n/a	England	PCT		P5		1	Searches - MEDLINE
Patel(99)	2020	Journal article	To describe the uptake and outputs of the NHSHC programme	Cross-sectional	n/a	England	Transition, LA		P3, P12		2	Searches - MEDLINE
Paxton(100)	2020	Journal article	To assess the fidelity of delivery of NHSHCs in general practice	Fidelity assessment	n/a	East of England	PCT		P20, P21		2	Searches - MEDLINE
Perkins(101)	2020	Conference presentation	Presentation describing CVD prevention initiatives	n/a	n/a	England	LA			A2, A38	2	NHSHC website
Perry(102)	2014	Journal article	To explore experiences of engaging with a community-based NHSHC	Qualitative interview study	Attendees	Knowsley	PCT		P14, P28	A2, A4, A7, A13, A18, A25, A35, A38, A40, A44	12	Existing review

Public Health England(103)	2020	Guidance	Best Practice Guidance for commissioners and providers	n/a	n/a	England	LA		P12	A12	2	NHSHC website
Rawlinson(104)	2019	Journal article	To evaluate an 'enhanced health promotion service' in a physiotherapy-led musculoskeletal service providing NHSHCs and diabetes checks	Mixed methods	n/a	Salford	LA	C6	P6, P7, P8, P14, P17, P19, P26		8	Searches - CINAHL
Research Works(105)	2013	Evaluation report	To assess commissioners' and providers' experiences of the NHSHC and gain an understanding of the engagement of public health professionals with NHSHCs	Qualitative interview study	Commissioners, providers	Various	PCT, Transition	C1, C4, C7, C11, C13	P7, P9, P10, P13, P14, P19	A13	12	Existing review
Richardson(106)	2016	News article	Article responding to research demonstrating lower coverage of NHSHCs and limited evidence of effectiveness of NHSHCs	n/a	n/a	n/a	LA		P18	A16	2	Searches - CINAHL

Riley(107)	2015	Journal article	To examine the feasibility and acceptability of community outreach NHSHCs targeted at the Afro-Caribbean community	Ethnography	Providers, attendees	Bristol	LA		P10	A2, A3, A13, A14, A16, A17, A21, A23, A25, A26, A40, A44	13	Existing review
Riley(108)	2016	Journal article	To examine the experiences of patients attending and healthcare professionals conducting NHSHCs	Qualitative interview study	Providers, attendees	Bristol	LA		P4, P8, P10, P27	A1, A3, A11, A19, A20, A21, A22, A25, A33, A35, A38, A44	16	Existing review
Robson(109)	2015	Journal article	To describe implementation and results of the NHSHCs	Cross-sectional	n/a	City & Hackney, Newham, Tower Hamlets	PCT		P9		1	Existing review
Robson(110)	2017	Journal article	To describe the coverage and impact of NHSHCs on cardiovascular risk management and identification of new comorbidities	Retrospective cohort	n/a	City & Hackney, Newham, Tower Hamlets	PCT, Transition, LA		P9		1	Existing review

Saramunee(111)	2015	Journal article	To explore the experience of and willingness to use seven pharmacy public health services related to cardiovascular risk among the general public in England (including the NHSHC)	Mixed methods	Attendees	Sefton				A15, A29	2	Searches - MEDLINE
Shaw(112)	2015	Journal article	To explore health care professionals' and patients' experiences of delivering and receiving the NHSHC	Qualitative interview study	Providers, attendees	Birmingham	PCT	C14	P8, P12, P23, P24, P27	A2, A3, A4, A6, A9, A24, A26, A32, A35, A36, A38, A40	18	Existing review
Shaw(113)	2016	Journal article	To evaluate the implementation of the NHSHC from the perspective of GPs	Qualitative interview study	Providers	Birmingham	PCT		P1, P5, P6, P9, P10, P11, P13, P17, P20, P21, P26, P27	A1, A6, A10, A12, A20, A30, A39	19	Existing review
Simon(114)	2020	Conference presentation	Presentation describing physical activity interventions for prevention and management of health conditions	n/a	n/a	England	LA		P26		1	NHSHC website

Solutions Strategy(115)	2017	Evaluation report	To evaluate the pilot and assess the feasibility of extending the NHSHC to include a dementia risk reduction component	Mixed methods	n/a	Birmingham, Bury, Manchester, Southampton			P26	A13, A15, A26	4	Searches - HMIC
Strutt(116)	2011	PhD thesis	To investigate patients' experiences of and responses to NHSHCs	Qualitative interview study / ethnography	Attendees	n/a	PCT		P1, P16	A2, A5, A6, A7, A11, A13, A15, A20, A21, A22, A25, A26, A29, A30, A34, A35, A44	19	Existing review
Tanner(117)	2020	Research report	To update PHE-commissioned rapid evidence synthesis and summarise evidence to address several research questions relating to the implementation of NHSHCs	Systematic review	Various	n/a	n/a		P2		1	NHSHC website
Thompson(118)	2016	Conference presentation	Presentation describing PHE's 'StARS' framework for assessment of NHSHCs	n/a	Commissioners	England	LA	C11			1	NHSHC website
Thompson(119)	2019	Conference abstract	Summary of implementation and outcomes of an integrated	Cross-sectional	Commissioner	Slough	LA	C3	P19		2	NHSHC website

			cardiovascular service									
Trueland(120)	2013	News article	Article based around an interview with NHSHC supporter Professor Michael Kirby	n/a	Commissioners	n/a	LA	C8			1	Searches - HMIC
Turner(121)	2013	Journal article	To evaluate variation in statin take up following risk assessment in the NHSHC	Qualitative interview study	Attendees	Nottingham	PCT			A15, A28, A41, A42, A43	5	Searches - HMIC
Usher-Smith(122)	2017	Research report	To provide a rapid synthesis of published research evidence on NHSHCs	Systematic review	n/a	n/a	n/a		P8		1	NHSHC website
Visram(123)	2012	Evaluation report	To investigate the implementation and accessibility of the 'health trainer community check' service	Mixed methods	Various	Durham	PCT, Transition	C1, C5, C9	P6, P12, P19, P27	A2, A3, A4, A5, A31, A32, A33	14	NHSHC website
Williams(124)	2018	Conference presentation	Presentation describing physical activity training and use of the GPPAQ tool to assess physical activity levels	n/a	n/a	England	LA		P21, P26		2	NHSHC website

nd=no date

[a]*Commissioning period here refers to whether the data included in the document relate to the period when NHSHCs were commissioned by PCTs or LAs. Documents contributing data that spans the 'transition' period (from mid-2012 to 2013) are also noted.*

[b] *Identifying details of LAs have been removed where unpublished material was shared by survey respondents*

Reference list (included documents)

1. Alageel S. Implementing multiple behaviour change interventions after health checks in primary care: a qualitative study. Cardiovascular Disease Prevention Conference 2018: Getting serious about prevention: reducing variation and optimizing care London, UK: NHS England; 2018.
2. Alageel S, Gulliford MC, McDermott L, Wright AJ. Implementing multiple health behaviour change interventions for cardiovascular risk reduction in primary care: a qualitative study. BMC Fam Pract. 2018;19(1):171.
3. Alageel S, Gulliford MC, Wright A, Khoshaba B, Burgess C. Engagement with advice to reduce cardiovascular risk following a health check programme: A qualitative study. Health Expect. 2020;23(1):193-201.
4. Alford S, Perry C. Knowsley at Heart community NHS health checks: Behaviour change evaluation. 2010. Accessed
5. Al-Osaimi A. Improving lifestyle support following the NHS Health Check programme; a whole system approach. Cardiovascular Disease Prevention Conference 2020: proactive, predictive, personalised Kia Oval, London: NHS England; 2020.
6. Artac M, Dalton AR, Majeed A, Car J, Millett C. Effectiveness of a national cardiovascular disease risk assessment program (NHS Health Check): results after one year. Prev Med. 2013;57(2):129-34.
7. Atkins L, Stefanidou C, Chadborn T, Thompson K, Michie S, Lorencatto F. Influences on NHS Health Check behaviours: a systematic review. BMC Public Health. 2020;20(1):1359.
8. Baker C, Loughren E, Crone D. Evaluation of the Gloucestershire NHS Health Check Programme, for the period July 2011 to July 2012. 2014. Available from: https://www.healthcheck.nhs.uk/commissioners-and-providers/evidence/local-evaluation/. Accessed
9. Baker C, Loughren EA, Crone D, Kallfa N. Patients' perceptions of a NHS Health Check in the primary care setting. Qual Prim Care. 2014;22:232-37.
10. Baker C, Loughren EA, Crone D, Kallfa N. Perceptions of health professionals involved in a NHS Health Check care pathway. Practice Nursing. 2015;26(12):608-12.
11. Baker C, Loughren EA, Crone D, Kallfa N. A process evaluation of the NHS Health Check care pathway in a primary care setting. J Public Health (Oxf). 2015;37(2):202-9.
12. Bell K. Integrating an outreach NHS Health Check Programme into a Lifestyle Interventions Hub. Cardiovascular Disease Prevention Conference 2019: Saving Hearts and Minds Together Manchester, UK2019.
13. Boase S, Mason D, Sutton S, Cohn S. Tinkering and tailoring individual consultations: how practice nurses try to make cardiovascular risk communication meaningful. J Clin Nurs. 2012;21(17-18):2590-8.
14. Boseley R. Personalised and proactive CVD Prevention: NHS Health Checks and Integrated Lifestyle services in East Sussex. Cardiovascular Disease Prevention Conference 2020: proactive, predictive, personalised Kia Oval, London: NHS England; 2020.
15. Brutus L, Fluke R. Croydon NHS Health Check Programme: Review and Options Appraisal. 2013. Available from: https://www.healthcheck.nhs.uk/commissioners-and-providers/evidence/local-evaluation/. Accessed
16. Burgess C. Engagement with advice to reduce cardiovascular risk following an NHS Health Check. NHS Health Check Conference: Getting Serious about Prevention London, UK: NHS England; 2016.

17. Burke D, Kirby H. Leeds patient insight and engagement from vulnerable groups: how to reduce inqualities. NHS Health Check 2015 - Improvement through collaboration 2015.
18. Carter P, Bodicoat DH, Davies MJ, Ashra NB, Riley D, Joshi N, et al. A retrospective evaluation of the NHS Health Check Programme in a multi-ethnic population. J Public Health (Oxf). 2016;38(3):534-42.
19. Centre for Public Scrutiny. Checking the nation's health: the value of council scrutiny. London: CfPS, 2014; 2014. Available from: http://www.cfps.org.uk/includes/scripts/force_download.php?file=../../domains/cfps.org.uk/local/media/downloads/CfPS_Nations_Health_final_online.pdf. Accessed
20. Chatterjee R, Chapman T, Brannan MG, Varney J. GPs' knowledge, use, and confidence in national physical activity and health guidelines and tools: a questionnaire-based survey of general practice in England. Br J Gen Pract. 2017;67(663):e668-e75.
21. Chipchase L, Hill P, Waterall J. NHS Birmingham East & North. An insight into the NHS Health Check Programme in Birmingham: Summary Report. 2011. Accessed
22. Clarke R, Brierley A. Using NHS Health Checks data to galvanise public health prevention. Cardiovascular Disease Prevention Conference 2020: proactive, predictive, personalised Kia Oval, London: NHS England; 2020.
23. Cochrane T, Davey R, Iqbal Z, Gidlow C, Kumar J, Chambers R, et al. NHS health checks through general practice: randomised trial of population cardiovascular risk reduction. BMC Public Health. 2012;12:944.
24. Cochrane T, Gidlow CJ, Kumar J, Mawby Y, Iqbal Z, Chambers RM. Cross-sectional review of the response and treatment uptake from the NHS Health Checks programme in Stoke on Trent. J Public Health (Oxf). 2013;35(1):92-8.
25. Coffey M, Cooper A, Brown T, Cook P, Clarke-Cornwell A. Vascular health checks in Salford: an exploration using FARSITE data. University of Salford,; 2014. Accessed
26. Coghill N, Garside L, Montgomery AA, Feder G, Horwood J. NHS health checks: a cross- sectional observational study on equity of uptake and outcomes. BMC Health Serv Res. 2018;18(1):238.
27. Collins K-a. Evaluation of the NHS Health Check Outreach Programme in Medway. NHS Medway; 2011. Available from: https://www.healthcheck.nhs.uk/commissioners-and-providers/evidence/local-evaluation/. Accessed
28. Corlett SA, Krska J. Evaluation of NHS Health Checks provided by community pharmacies. J Public Health (Oxf). 2016;38(4):e516-e23.
29. Coward C. Understanding and accessing the local physical activity system. Cardiovascular Disease Prevention Conference 2020: proactive, predictive, personalised Kia Oval, London: NHS England,; 2020.
30. Cupit C. An ethnographic study of cardiovascular disease prevention: the social organisation of measures, knowledge, interventions and tensions in English general practice: University of Leicester; 2018.
31. Dalton AR, Bottle A, Okoro C, Majeed A, Millett C. Uptake of the NHS Health Checks programme in a deprived, culturally diverse setting: cross-sectional study. J Public Health (Oxf). 2011;33(3):422-9.
32. Derbyshire County Council, Services PHIaK. Health Equity Audit: NHS Health Check Programme in Derbyshire County. 2015. Available from: https://www.healthcheck.nhs.uk/commissioners-and-providers/evidence/local-evaluation/. Accessed

33. Edmans T. Training for the dementia component of the NHS Health Check, NHS Southwark. Southwark Council,; 2013. Available from: https://www.healthcheck.nhs.uk/commissioners-and-providers/evidence/case-studies/. Accessed
34. Fenton K. Getting serious about preventing cardiovascular disease. Cardiovascular Disease Prevention Conference 2018: Getting serious about prevention: reducing variation and optimizing care London, UK2018.
35. Forster AS, Dodhia H, Booth H, Dregan A, Fuller F, Miller J, et al. Estimating the yield of NHS Health Checks in England: a population-based cohort study. J Public Health (Oxf). 2015;37(2):234-40.
36. Forsyth P, Ali S, Ameen M, Khan R, Khan F, Sheikh AR, et al. Pharmacist-led anticipatory care for the South Asian community. International Journal of Pharmacy Practice. Royal Pharmaceutical Society, RPS Annual Conference 2012. Birmingham United Kingdom.

(var.pagings). 20: Pharmaceutical Press; 2012. p. 88-9.
37. Frazer H. Implementing NHS Health Checks within a non-clinical integrated lifestyle service. Cardiovascular Disease Prevention Conference 2020: proactive, predictive, personalised Kia Oval, London: NHS England; 2020.
38. Gidlow CJ, Ellis NJ, Cowap L, Riley VA, Crone D, Cottrell E, et al. Quantitative examination of video-recorded NHS Health Checks: comparison of the use of QRISK2 versus JBS3 cardiovascular risk calculators. BMJ Open. 2020;10(9):e037790.
39. Gidlow C, Riley V. Physical activity and alcohol measurement in NHS Health Checks. Staffordshire University, Public Health England; 2021. Available from: https://www.healthcheck.nhs.uk/commissioners-and-providers/evidence/. Accessed
40. Gidlow CJ, Ellis NJ, Cowap L, Riley V, Crone D, Cottrell E, et al. Cardiovascular disease risk communication in NHS Health Checks using QRISK®2 and JBS3 risk calculators: the RICO qualitative and quantitative study. Health Technol Assess. 2021;25(50).
41. Graley C, Katherine MF, McCoy DC. Postcode Lotteries in Public Health - The NHS Health Checks Programme in North West London. BMC Public Health. 2011;11(738).
42. Greaves C, Gillison F, Stathi A, Bennett P, Reddy P, Dunbar J, et al. Waste the waist: a pilot randomised controlled trial of a primary care based intervention to support lifestyle change in people with high cardiovascular risk. Int J Behav Nutr Phys Act. 2015;12:1.
43. Green K, Forshaw A, D'Souza H, Macherianakis A, MEL Research. NHS Health Check Patient Journey Evaluation: Final Evaluation Report. East Sussex County Council Public Health, MEL Research; 2018. Accessed
44. Gregory A, Chowdary Q, Tobacco Control Programme P. The role of healthcare professionals in treating tobacco dependency: opportunities to reduce CVD for commissioners and practitioners. Cardiovascular Disease Prevention Conference 2018: Getting serious about prevention: reducing variation and optimizing care London, UK2018.
45. Gulliford MC, Khoshaba B, McDermott L, Cornelius V, Ashworth M, Fuller F, et al. Cardiovascular risk at health checks performed opportunistically or following an invitation letter. Cohort study. J Public Health (Oxf). 2018;40(2):e151-e6.
46. Hardman L. The BIG Bolton Health Check. 2014 October 2014. Available from: https://www.healthcheck.nhs.uk/commissioners-and-providers/evidence/case-studies/. Accessed
47. Haringay Council. Scrutiny Review Men's Health: Getting to the Heart of the Matter. A review by the Overview and Scrutiny Committee. 2012. Available from: https://www.healthcheck.nhs.uk/commissioners-and-providers/evidence/local-evaluation/. Accessed

48. Hawking M. The CVD Risk Report, an intervention to support CVD risk communication in NHS Health Checks: results from the pilot trial. Cardiovascular Disease Prevention Conference 2018: Getting serious about prevention: reducing variation and optimizing care London, UK: NHS England; 2018.
49. Hawking MKD, Timmis A, Wilkins F, Potter JL, Robson J. Improving cardiovascular disease risk communication in NHS Health Checks: a qualitative study. BMJ Open. 2019;9(8):e026058.
50. Hinde S, Bojke L, Richardson G, Retat L, Webber L. The cost-effectiveness of population Health Checks: have the NHS Health Checks been unfairly maligned? Journal of Public Health: From Theory to Practice. 2017;25(4):425-31.
51. Homer K, Boomla K, Hull S, Dostal I, Mathur R, Robson J. Statin prescribing for primary prevention of cardiovascular disease: a cross-sectional, observational study. Br J Gen Pract. 2015(e538).
52. Honey S, Bryant LD, Murray J, Hill K, House A. Differences in the perceived role of the healthcare provider in delivering vascular health checks: a Q methodology study. BMC Fam Pract. 2013;14:172.
53. Honey S, Hill K, Murray J, Craigs C, House A. Patients' responses to the communication of vascular risk in primary care: a qualitative study. Prim Health Care Res Dev. 2015;16(1):61-70.
54. Hooper J, Chohan P, Caley M. Case detection of disease by NHS Health Checks in Warwickshire, England and comparison with predicted performance. Public Health. 2014;128(5):475-7.
55. Hyseni L, Guzman-Castillo M, Kypridemos C, Collins B, Schwaller E, Capewell S, et al. Engaging with stakeholders to inform the development of a decision-support tool for the NHS health check programme: qualitative study. BMC Health Serv Res. 2020;20(1):394.
56. Ismail H, Kelly S. Lessons learned from England's Health Checks Programme: using qualitative research to identify and share best practice. BMC Fam Pract. 2015;16:144.
57. Ismail H, Atkin K. The NHS Health Check programme: insights from a qualitative study of patients. Health Expect. 2016;19(2):345-55.
58. Ismail K, Stahl D, Bayley A, Twist K, Stewart K, Ridge K, et al. Enhanced motivational interviewing for reducing weight and increasing physical activity in adults with high cardiovascular risk: the MOVE IT three-arm RCT. Health Technol Assess. 2019;23(69).
59. Jones T. Telford Healthy Hearts. Cardiovascular Disease Prevention Conference 2020: proactive, predictive, personalised Kia Oval, London2020.
60. Kearney M. Engaging with GPs to help deliver the NHS Health Checks. NHS Health Check 2015 - Improvement through collaboration 2015.
61. Kennedy O, Su F, Pears R, Walmsley E, Roderick P. Evaluating the effectiveness of the NHS Health Check programme in South England: a quasi-randomised controlled trial. BMJ Open. 2019;9(9):e029420.
62. Kirkpatrick W, Eden A. Developing a resilient community & primary care based workforce to deliver the NHS Health Check. NHS Health Check Conference: Getting Serious about Prevention London, UK: NHS England; 2016.
63. Krska J, Mackridge AJ, Taylor J. An Evaluation of the Cardiovascular Screening Service provided by community pharmacies in Sefton PCT: Final Report. Liverpool John Moores University; 2010. Available from: https://www.healthcheck.nhs.uk/commissioners-and-providers/evidence/local-evaluation/. Accessed
64. Krska J, Taylor J, Du Plessis R. Views and experiences of the NHS Health Check provided in pharmacies and medical practices. International Journal of Pharmacy Practice Health Services Research and Pharmacy Practice Conference 2013. Preston United Kingdom.

(var.pagings). 21: Pharmaceutical Press; 2013. p. 16.
65. Krska J, du Plessis R, Chellaswamy H. Views and experiences of the NHS Health Check provided by general medical practices: cross-sectional survey in high-risk patients. J Public Health (Oxf). 2014;37(2):210-7.
66. Krska J, du Plessis R, Chellaswamy H. Views of practice managers and general practitioners on implementing NHS Health Checks. Prim Health Care Res Dev. 2016;17(2):198-205.
67. Krska J, du Plessis R, Chellaswamy H. Implementation of NHS Health Checks in general practice: variation in delivery between practices and practitioners. Prim Health Care Res Dev. 2016;17(4):385-92.
68. Kumar J. GP Engagement "If they know you, it's harder for them to say no". NHS Health Check 2015 - Improvement through collaboration 2015.
69. Lake G. The Healthy Heart Centre: NHS Camden's approach to reducing Health Inequalities. NHS Camden; 2010. Available from: https://www.healthcheck.nhs.uk/commissioners-and-providers/evidence/local-evaluation/. Accessed
70. Lambert MF. Assessing potential local routine monitoring indicators of reach for the NHS health checks programme. Public Health. 2016;131:92-8.
71. Lennon S, London Borough of Redbridge. Supporting LTC Prevention During the COVID-19 Pandemic: Improving NHS Health Checks and Social Prescribing Initiatives (Presentation). 2020.
72. Liverpool City Council. LiveWire Liverpool Health Trainers. Cardiovascular Disease Prevention Conference 2020: proactive, predictive, personalised Kia Oval, London2020.
73. Local Government Association. Public health transformation twenty months on: adding value to tackle local health needs.; 2015. Accessed
74. Local Authority A. The [Local Authority Borough] NHS Health Checks Programme 2019 Review [Unpublished; identifying information redacted]. 2019. Accessed
75. Local Authority B. NHS Health Checks in [Local Authority Borough] [Unpublished; identifying information redacted]. 2019. Accessed
76. Local Authority C. Health Check Quality Assurance - Working Document [Unpublished; identifying information redacted]. Accessed
77. Local Authority C. NHS Health Checks [Local area] Performance and Quality Framework 2019 [Unpublished; identifying information redacted]. 2019. Accessed
78. London Borough of Bromley. Re audit of the Prevention of Diabetes through NHS Health Checks 2014-15. Public Health England,; 2018 January 2018. Available from: https://www.healthcheck.nhs.uk/commissioners-and-providers/evidence/case-studies/. Accessed
79. Loo R, Diaper C, Salami O, Kundu M, Lalkia M, Airhiavbere E, et al. The NHS Health Check: the views of community pharmacists. Int J Pharm Pract. 2011;19(13).
80. Lumley B. Tackling high blood pressure: system wide action and the role of the NHS Health Check. NHS Health Check 2015 - Improvement through collaboration 2015.
81. Maddern S. Partnership Working - the role of local political scrutiny in driving forward the NHS Health Check Programme in Wiltshire. Cardiovascular Disease Prevention Conference 2020: proactive, predictive, personalised. Kia Oval, London: NHS England; 2020.
82. Martin H. Delivering NHS health checks to all. British Journal of Healthcare Management. 2011;17(6):250-5.
83. McDermott L. Exploring the Health Check programme in Primary Care. NHS Health Check 2015 - Improvement through collaboration 2015.

84. McMillan B, Fox S, Lyons M, Bourke S, Mistry M, Ruddock A, et al. Using patient and public involvement to improve the research design and funding application for a project aimed at fostering a more collaborative approach to the NHS health check: the CaVIAR project (better Care Via Improved Access to Records). Research Involv Engagem. 2018;4:18.
85. McNaughton RJ, Oswald NT, Shucksmith JS, Heywood PJ, Watson PS. Making a success of providing NHS Health Checks in community pharmacies across the Tees Valley: a qualitative study. BMC Health Serv Res. 2011;11:222.
86. McNaughton RJ, Shucksmith J. Reasons for (non)compliance with intervention following identification of 'high-risk' status in the NHS Health Check programme. J Public Health (Oxf). 2014;37(2):218-25.
87. Mills K, Harte E, Martin A, MacLure C, Griffin SJ, Mant J, et al. Views of commissioners, managers and healthcare professionals on the NHS Health Check programme: a systematic review. BMJ Open. 2017;7(11):e018606.
88. Nahar P, van Marwijk H, Gibson L, Musinguzi G, Anthierens S, Ford E, et al. A protocol paper: community engagement interventions for cardiovascular disease prevention in socially disadvantaged populations in the UK: an implementation research study. Glob Health Res Policy. 2020;5:12.
89. Nasir N. Can alternate providers improve the uptake of Health Checks among high risk populations and subsequent referrals to the local lifestyle services? Getting Serious About Cardiovascular Disease Prevention 2018: Reducing Variation and Optimising Care London, UK2018.
90. Newman P. An Introduction to Health Coaching: Better Conversations, Better Health. Cardiovascular Disease Prevention Conference 2018: Getting serious about prevention: reducing variation and optimizing care London, UK2018.
91. NHS Greenwich. Evaluation of NHS Health Check Plus Community Outreach Programme in Greenwich. 2011. Accessed
92. NHS Midlands and East. A study into increasing uptake of lifestyle changes - Report. 2011. Accessed
93. NICE. Encouraging people to have NHS Health Checks and supporting them to reduce risk factors. Manchester: NICE, 2014; 2014. Available from: http://publications.nice.org.uk/encouraging-people-to-have-nhs-health-checks-and-supporting-them-to-reduce-risk-factors-lgb15. Accessed
94. Nicholas JM, Burgess C, Dodhia H, Miller J, Fuller F, Cajeat E, et al. Variations in the organization and delivery of the 'NHS health check' in primary care. J Public Health (Oxf). 2012;35(1):85-91.
95. O'Flaherty M, Lloyd-Williams F, Capewell S, Boland A, Maden M, Collins B, et al. Modelling tool to support decision-making in the NHS Health Check programme: workshops, systematic review and co-production with users. Health Technol Assess. 2021;25(35).
96. Onyia I. Delivery of the NHS Health Check by health trainers can improve conversion into uptake of lifestyle service. NHS Health Check Conference: Getting Serious about Prevention London, UK: NHS England; 2016.
97. Oswald N, McNaughton R, Watson P, Shucksmith J. Tees Vascular Assessment Programme: Evaluation commissioned by the Tees Primary Care Trusts (PCT) from the Centre for Translational Research in Public Health. 2010. Available from: https://www.healthcheck.nhs.uk/commissioners-and-providers/evidence/local-evaluation/. Accessed
98. Palladino R, Vamos EP, Chang KC, Khunti K, Majeed A, Millett C. Evaluation of the Diabetes Screening Component of a National Cardiovascular Risk Assessment Programme in England: a Retrospective Cohort Study. Sci Rep. 2020;10(1):1231.
99. Patel R, Barnard S, Thompson K, Lagord C, Clegg E, Worrall R, et al. Evaluation of the uptake and delivery of the NHS Health Check programme in England, using primary care data from 9.5 million people: a cross-sectional study. BMJ Open. 2020;10(11):e042963.
100. Paxton B, Mills K, Usher-Smith JA. Fidelity of the delivery of NHS Health Checks in general practice: an observational study. BJGP Open. 2020;4(4).

101. Perkins C. Cardiovascular disease prevention in the 2020s. Cardiovascular Disease Prevention Conference 2020: proactive, predictive, personalised Kia Oval, London2020.
102. Perry C, Thurston M, Alford S, Cushing J, Panter L. The NHS health check programme in England: a qualitative study. Health Promot Int. 2014;31(1):106-15.
103. Public Health England. NHS Health Check Best practice guidance For commissioners and providers. 2020. Available from: https://www.healthcheck.nhs.uk/commissioners-and-providers/national-guidance/. Accessed 31st October 2019.
104. Rawlinson G. Health promotion in physiotherapy services using NHS health and diabetes checks. British Journal of Healthcare Management. 2019;25(1):22-31.
105. Research Works, Public Health England. Understanding the implementation of NHS Health Checks. 2013. Accessed
106. Richardson J. The fall in NHS Health Checks. Nursing in Practice: The Journal for Today's Primary Care Nurse. 2016(91):1-3.
107. Riley R, Coghill N, Montgomery A, Feder G, Horwood J. The provision of NHS health checks in a community setting: an ethnographic account. BMC Health Serv Res. 2015;15:546.
108. Riley R, Coghill N, Montgomery A, Feder G, Horwood J. Experiences of patients and healthcare professionals of NHS cardiovascular health checks: a qualitative study. J Public Health (Oxf). 2016;38(3):543-51.
109. Robson J, Dostal I, Madurasinghe V, Sheikh A, Hull S, Boomla K, et al. The NHS Health Check programme: implementation in east London 2009-2011. BMJ Open. 2015;5(4):e007578.
110. Robson J, Dostal I, Madurasinghe V, Sheikh A, Hull S, Boomla K, et al. NHS Health Check comorbidity and management: an observational matched study in primary care. Br J Gen Pract. 2017;67(655):e86-e93.
111. Saramunee K, Krska J, Mackridge A, Richards J, Suttajit S, Phillips-Howard P. General public's views on pharmacy public health services: current situation and opportunities in the future. Public health. 2015;129(6):705-15.
112. Shaw RL, Pattison HM, Holland C, Cooke R. Be SMART: examining the experience of implementing the NHS Health Check in UK primary care. BMC Fam Pract. 2015;16:1.
113. Shaw RL, Lowe H, Holland C, Pattison H, Cooke R. GPs' perspectives on managing the NHS Health Check in primary care: a qualitative evaluation of implementation in one area of England. BMJ Open. 2016;6(7):e010951.
114. Simon C. Physical activity for prevention and management of health conditions and practitioner resources. Cardiovascular Disease Prevention Conference 2020: proactive, predictive, personalised Kia Oval, London: NHS England,; 2020.
115. Solutions Strategy Research Facilitation Ltd, Alzheimer's Society. NHS Health Check 40-64 dementia pilot research findings: summary research report. 2017.
116. Strutt E. Patient-centred care: Patients' experiences of and responses to the National Health Service (NHS) Health Check programme in general practice. Durham: Durham University; 2011.
117. Tanner L, Kenny R, Still M, Pearson F, Bhardwaj-Gosling R. NHS Health Check Programme Rapid Review Update. University of Sunderland, Newcastle University, Public Health England; 2020. Available from: https://www.healthcheck.nhs.uk/commissioners-and-providers/evidence/. Accessed 31st October 2020.

118. Thompson K. NHS Health Check StARS framework: A Systems Approach for Raising Standards. NHS Health Check Conference: Getting Serious about Prevention London, UK: NHS England; 2016.
119. Thompson S. Integrated Cardiac Disease Prevention Programme. Cardiovascular Disease Prevention Conference 2019: Saving Hearts and Minds Together. Manchester, UK2019.
120. Trueland J. 'The signs were there, but weren't picked up'. Health Serv J. 2013;123(6369):6-7.
121. Turner A. Exploratory evaluation of variation in statin take up among high risk patients in Nottingham City. Public Health. 2013.
122. Usher-Smith JA, Mant J, Martin A, Harte E, MacLure C, Meads C, et al. NHS Health Check Programme rapid evidence synthesis. Cambridge, UK: The Primary Care Unit, University of Cambridge, RAND Europe, Public Health England; 2017. Accessed 31st October 2020.
123. Visram S, Geddes L, Carr SM. Formative Evaluation of the Health Trainer Community Health Check Service in County Durham. Northumbria University, County Durham and Darlington NHS Foundation Trust; 2012. Available from: https://www.healthcheck.nhs.uk/commissioners-and-providers/evidence/local-evaluation/. Accessed
124. Williams Z. Let's talk about Physical Activity: Physical Activity Clinical Champions training and using the GPPAQ in a health check setting. Cardiovascular Disease Prevention Conference 2018: Getting serious about prevention: reducing variation and optimizing care London, UK2018.
