# Supplementary material for: Understanding what happens to attendees after an NHS Health Check: a realist review
Source: BMJ Open. 2022 Nov 10;12(11):e064237. doi: 10.1136/bmjopen-2022-064237 (PMC9660666; doi:10.1136/bmjopen-2022-064237)
Supplement: Supplementary data [file bmjopen-2022-064237supp003.pdf]

Supplementary File 4

This file contains 3 tables outlining the full set of CMOCs (n=86) developed during the review. The three tables split the CMOCs up into those centred on commissioner (Table S1), provider (Table S2) and attendee (Table S3) perspectives.

Table S1 Overview of CMOCs focused on LA commissioners

	**CMOC**	**Summary of data**
Understanding and engagement with the NHSHC programme: case finding or enabling behaviour change?		
CMOC C1	When commissioners view the NHSHC as a means to improve people's lives through behaviour change (C) they will try to exert their influence over providers to ensure the programme is delivered with this in mind (O) because they believe this will maximise the potential benefits of the programme (M)	Data extracted from 12 documents:<br><br>2 research articles (one cohort study, one qualitative interview study); 4 local evaluation reports; 3 conference materials; 1 unpublished LA working document; 2 other reports focused on LA roles in NHSHCs.
CMOC C2	When commissioners view the NHSHC as a means to improve people's lives through behaviour change (C) they will try to establish closer working relationships between different parts of the wider system (the NHSHC, lifestyle services and primary care) to improve referral pathways (O) because they believe this will maximise the potential benefits of the programme (M)	Data extracted from 8 documents:<br><br>1 research article (survey); 4 local evaluation reports; 2 conference materials; 1 unpublished LA working document.

CMOC C3	When commissioners view the NHSHC as a means to improve people's lives through behaviour change (C) they may develop 'integrated' models of service delivery with the NHSHC delivered alongside lifestyle services (O) because they believe this will maximise the potential benefits of the programme (M)3,	Data extracted from 5 documents:<br>1 local evaluation report; 4 conference materials.
CMOC C4	When providers are sceptical and less engaged with the NHSHC programme (C) commissioners may be unable to exert their influence over them (O1) or establish close working relationships between different parts of the system (O2) because providers are resistant and unwilling to engage (M)	Data extracted from 3 documents:<br>1 local evaluation report; 1 conference presentation; 1 unpublished LA working document.
CMOC C5	When commissioners have a focus on the wider social determinants of health (C) they are more likely to commission 'alternative' NHSHC providers (i.e. to move away from a medical model based in primary care) (O) because they believe this will maximise the benefits of the programme (M)	Data extracted from 3 documents:<br>1 research article (qualitative interviews/workshop); 1 local evaluation report; 1 report focused on LA roles in NHSHCs.
CMOC C6	When commissioners have a focus on the wider social determinants of health (C) they may integrate NHSHC delivery with other services that address other problems (O) because they believe this will maximise the potential benefits of the programme (M)	Data extracted from 3 documents:<br>1 research article (mixed methods evaluation); 2 conference materials.

CMOC C7	When commissioners view the NHSHC as a means to improve people's lives through behaviour change (C) they are more likely to collect data related to what happens after the measurements and risk assessment are completed in a check (O) because they believe these are important data to monitor and evaluate programme performance (M)	Data extracted from 15 documents:<br><br>2 research articles (1 qualitative interviews/workshop, 1 qualitative interview study); 1 research report (mixed methods study); 7 local evaluation reports; 4 conference materials; 1 unpublished LA working document.
CMOC C8	When commissioners view the NHSHC as a means to improve people's lives through behaviour change (C) they are more likely to collect data related to the needs of the local population to inform the commissioning of lifestyle support services (O) because they believe this will maximise the benefits of the programme (M)	Data extracted from 6 documents:<br><br>1 PhD thesis (ethnography); 1 local evaluation report; 2 conference materials; 1 report focused on LA roles in NHSHCs; 1 news article.
CMOC C9	In some circumstances, commissioners may cede more control over delivery to primary care based providers (e.g. GP practices) (O) but the contexts in which this happens and the mechanisms underpinning this outcome are not clear (C, M not defined)	Data extracted from 8 documents:<br><br>3 research articles (1 qualitative interview study, 1 survey, 1 cross-sectional study); 4 local evaluation reports; 1 conference materials.
CMOC C10	In some circumstances, commissioners may focus only on mandatory data collection (monitoring invitation, uptake and coverage) (O) but the contexts in which this happen and the mechanisms underpinning this outcome are not clear (C, M not defined)	Data extracted from 5 documents:<br><br>3 research articles (3 cross-sectional studies); 2 local evaluation reports.
Practical constraints: politics and funding		

CMOC C11	Where there is local political support and engagement with the NHSHC programme (C), local delivery is more likely to be evaluated (O1) and developed or improved (O2) because commissioners and public health teams are empowered to focus on the programme (M)	Data extracted from 10 documents:<br><br>1 research article (qualitative interview study); 3 local evaluation reports; 3 conference materials; 2 other reports focused on LA roles in NHSHCs; 1 guidance document
CMOC C12	When funding for public health programmes is constrained (C1) and commissioners or public health teams are convinced of the NHSHCs long term effectiveness and value (C2) local delivery is more likely to be evaluated (O1) and developed or improved (O2) because these activities are considered worthwhile (M)	Data extracted from 5 documents:<br><br>2 research articles (1 qualitative interview study; 1 economic evaluation); 2 local evaluation reports; 1 other report focused on LA roles in NHSHCs.
CMOC C13	When funding for public health programmes is constrained (C) commissioners may select providers who offer the best value for money / lowest cost per NHSHC delivered (O) because they must prioritise mandatory public health programme delivery ('prescribed functions') before funding additional services (M)	Data extracted from 9 documents:<br><br>4 research articles (2 qualitative interview studies; 1 cross-sectional, 1 economic evaluation); 2 local evaluation reports; 1 PhD thesis (ethnography); 1 other report focused on LA roles in NHSHCs; 1 practitioner-facing article
CMOC C14	When funding for public health programmes is constrained (C) commissioners must prioritise funding mandatory services ('prescribed functions') (M) leading to cuts and reduced capacity in non-mandatory services (e.g. local lifestyle support services) (O)	Data extracted from 11 documents:<br><br>8 research articles (5 qualitative interview studies, 1 survey, 1 cross-sectional study, 1 systematic review); 1 local evaluation report; 1 PhD thesis (ethnography); 1 conference presentation.

Table S2 Overview of CMOCs focused on NHSHC providers

	CMOC	Summary of data
Understanding and engagement with the NHSHC programme: scepticism versus 'buy in'		
CMOC P1	When providers are sceptical about the NHSHC programme as a behaviour change intervention (C) they may prioritise completing the mandatory elements of the check and fail to engage with the delivery of advice, brief interventions or referrals (O) because they do not believe these will help attendees (M)	Data extracted from 13 documents:<br><br>3 research articles (2 qualitative interview studies, 1 survey); 3 local evaluation reports; 4 conference materials; 1 other report focused on LA roles in NHSHCs; 1 PhD thesis (ethnography).
CMOC P2	When providers are sceptical about the NHSHC programme as a behaviour change intervention (C1) or sceptical about the effectiveness of behaviour change to reduce the risk of cardiovascular disease (C2) they are more likely to consider medication (e.g. statins or antihypertensives) as an appropriate intervention for those assessed at higher risk (O) because they believe these will help attendees (M)	Data extracted from 11 documents:<br><br>7 research articles (2 qualitative interview studies; 1 survey; 2 cross-sectional studies; 1 cohort study; 1 Q-methodology study); 2 local evaluation reports; 1 research report (systematic review); 1 PhD thesis (ethnography).
CMOC P3	When providers (who are able to prescribe) are sceptical about the recommended thresholds for prescription (C) they are less likely to prescribe (O) because they do not believe it will help attendees (M).<br><br>*This CMOC may apply to other interventions but we lack data to confirm or refute this.*	Data extracted from 4 documents:<br><br>3 research articles (3 cross-sectional studies); 1 conference presentation.

CMOC P4	When providers are worried about labelling healthy people as sick (C) they may avoid offering advice, referrals or prescriptions (O) because they are concerned about the potential harms of overdiagnosis (M)	Data extracted from 3 documents:<br><br>2 research articles (2 qualitative interview studies); 1 PhD thesis (ethnography).
CMOC P5	When providers are highly engaged with preventive health care (C) they are more likely to be highly engaged with the NHSHC programme (O) as they understand it to be a useful means of reaching more patients with this agenda (M)	Data extracted from 2 documents:<br><br>2 research articles (1 qualitative interview study, 1 cohort).
CMOC P6	When providers believe that lifestyle modification is an effective means of reducing CVD risk (C) they are more likely to offer attendees advice, brief interventions or referrals to lifestyle support services (especially as a first line of action) (O) because they believe these may help attendees (M)	Data extracted from 11 documents:<br><br>6 research articles (2 qualitative interview studies; 1 cohort study; 1 mixed methods study; 1 Q-methodology study); 1 research report (RCT); 1 local evaluation report; 3 conference materials
CMOC P7	When providers buy in to the NHSHC as an opportunity to support behaviour change (C) they are more likely to offer attendees advice, brief interventions or referrals to lifestyle support services (O) because they believe these will help attendees (M)	Data extracted from 11 documents:<br><br>5 research articles (1 qualitative interview study; 1 survey; 1 mixed methods study; 1 Q-methodology study); 1 research report (RCT); 4 local evaluation reports; 1 conference presentation
CMOC P8	When providers have the specific skills they need to support the delivery of advice, brief interventions and referrals (C) they are more likely to engage	Data extracted from 10 documents:

	with and prioritise these activities (O) because they feel confident to deliver them during the NHSHC encounter (M)	3 research articles (2 qualitative interview studies, 1 mixed methods study); 1 research report (systematic review); 4 conference materials.
Practical constraints: time and money		
CMOC P9	When funding arrangements for delivery of checks incentivise volume of delivery (C) providers may prioritise completing mandatory elements of the check and minimise time spent delivering advice, brief interventions or offering referrals (O) because they are aware they do not have to complete these (M)	Data extracted from 6 documents:<br><br>3 research articles (1 qualitative interview study, 1 retrospective cohort, 1 cross-sectional); 1 local evaluation report; 1 PhD thesis (ethnography); 1 LA internal working document.
CMOC P10	When providers have many competing priorities (C) they may prioritise completing mandatory elements of the check and minimise time spent delivering advice, brief interventions or offering referrals (O) because of expediency (M)	Data extracted from 16 documents:<br><br>10 research articles (4 qualitative interview studies, 1 systematic review, 3 surveys, 1 ethnography, 1 observational study based on video recordings); 3 local evaluation reports; 1 research report (mixed methods); 1 PhD thesis (ethnography), 1 conference presentation
CMOC P11	When there is a focus on increasing the volume of checks delivered (C) providers may offer more checks 'opportunistically' (i.e. not in a standalone appointment) (O) as they feel this is more efficient (M)	Data extracted from 5 documents:<br><br>4 research articles (2 qualitative interview studies, 1 retrospective cohort, 1 survey, 1 mixed methods); 1 local evaluation report

CMOC P12	It is clear that data about non-mandatory elements of a check are often under-recorded or recorded inconsistently (O) but the contexts in which this happens and the mechanisms underpinning this outcome are not clear (C, M not defined). Potentially important contexts here include competing priorities, a focus on delivery of mandatory elements of checks, lack of monitoring and/or incentivisation to collect particular data items, or difficulty in recording certain activities within existing data collection systems	Data extracted from 15 documents:<br><br>10 research articles (3 qualitative interview studies, 4 cross-sectional studies, 1 pre/post study, 1 retrospective cohort, 1 study assessing the validity of indicators); 3 local evaluation reports; 1 guidance document; 1 conference abstract.
CMOC P13	When providers do not feel they are adequately compensated for delivering checks (C) they may prioritise completing mandatory elements of the check and minimise time spent delivering advice, brief interventions, or offering referrals (O) because they do not feel it is worth the cost (M)	Data extracted from 10 documents:<br><br>6 research articles (2 qualitative interview studies; 1 cross-sectional studies, 1 quasi-RCT, 1 surveys, 1 systematic review); 2 local evaluation reports; 2 conference materials
CMOC P14	When providers 'buy in' to the NHSHC programme (see CMOC P7 above) (C1) and have adequate time and/or flexibility to deliver each check (C2) they may offer more personalised and in-depth advice and support (O) because they believe these may help attendees (M)	Data extracted from 9 documents:<br><br>2 research articles (1 qualitative interview study, 1 mixed methods); 5 local evaluation reports; 1 conference presentation; 1 practitioner-facing article
Practical constraints: referrals and follow-up		
CMOC P15	When multiple modifiable risk factors are identified during a check (C1) and separate lifestyle services exist for each (C2) providers (and attendees)	Data extracted from 4 documents:

	may agree to prioritise addressing one risk factor first (M) so the delivery of advice, brief interventions and referrals reflect this priority (O)	1 research article (qualitative interview study); 2 local evaluation reports, 1 conference presentation
CMOC P16	When providers don't perceive available lifestyle services to be a good 'fit' for individuals (C) they may avoid making referrals to these services (O) because they do not believe it will help attendees (M)	Data extracted from 4 documents:<br><br>2 research articles (1 retrospective cohort, 1 cross-sectional study); 2 local evaluation reports; 1 PhD thesis (interviews/ethnography)
CMOC P17	When information about local lifestyle services and referral routes is disparate and difficult to access (C) it is harder for providers to make referrals (O) because providers are unaware of available services and how to refer (M)	Data extracted from 9 documents:<br><br>2 research articles (1 qualitative interview study, 1 mixed methods); 4 local evaluation reports; 3 conference materials
CMOC P18	When providers have concerns about the quality of lifestyle support services (C) they may avoid making referrals (O) because they doubt they will help attendees (M)	Data extracted from 3 documents:<br><br>2 research articles (1 survey, 1 Q-methodology study); 1 news article
CMOC P19	When providers have established relationships and referral pathways to trusted lifestyle services (C) they are more likely to offer referrals (O) because this becomes a delivery norm (M)	Data extracted from 12 documents:<br><br>1 research article (mixed methods); 5 local evaluation reports; 6 conference materials
Practical constraints: relationships and confidence in the delivery of advice		

CMOC P20	When providers are concerned that discussion of a particular risk factor may cause offence or upset an attendee (C) they may avoid bringing it up or discussing it in-depth (O) because they lack confidence and want to avoid confrontation (M)	Data extracted from 11 documents:<br><br>6 research articles (3 qualitative interview studies, 1 survey, 1 observational study based on video recordings, 1 fidelity assessment); 2 local evaluation reports; 1 research report (observational study); 1 PhD thesis (ethnography); 1 other report
CMOC P21	When providers lack knowledge about recommendations in relation to a particular risk factor (C) they may avoid bringing it up or discussing it in-depth during a check (O) because they lack confidence in their advice (M)	Data extracted from 4 documents:<br><br>3 research articles (1 qualitative interview study; 1 survey; 1 fidelity assessment); one conference presentation
CMOC P22	When providers perceive that an attendee is unlikely to want to, or be able to change their lifestyle (C) they may avoid giving them advice or offering referrals to support this (O) because they do not think it will help (M1) or because they are worried it could damage their relationship (M2)	Data extracted from 4 documents:<br><br>3 research articles (2 qualitative interview studies; 1 Q-methodology study); 1 local evaluation report
CMOC P23	When providers feel they themselves are not good role models for healthy lifestyles (C) they may be reluctant to deliver advice or brief interventions, or make referrals (O) because they are worried about appearing hypocritical and lacking credibility (M)	Data extracted from 2 documents:<br><br>1 research article (qualitative interview study) and 1 conference presentation

CMOC P24	When providers have lived experience of (trying to) make lifestyle changes (C) they may share this and empathise with attendees (O) because they want to build rapport and a therapeutic alliance during checks (M)	Data extracted from 5 documents:<br><br>3 research articles (2 qualitative interview studies, 1 Q-methodology study); 1 local evaluation report; 1 PhD thesis (ethnography)
CMOC P25	When discussing a risk factor is normalised and routine (C) providers may be more likely to deliver advice, brief interventions and offer referrals related to that risk factor (O) because they feel comfortable and practiced in doing so (M)	Data extracted from 6 documents:<br><br>1 research article (survey); 2 evaluation reports; 3 conference materials
CMOC P26	When providers receive training (C1) or have regular practice (C2) in delivering lifestyle advice, they are more likely to deliver it regularly during checks (O) because they feel more confident (M)	Data extracted from 9 documents:<br><br>3 research articles (1 qualitative interview study, 1 mixed methods, 1 RCT); 1 local evaluation reports; 5 conference materials
CMOC P27	When providers take into account attendees' own priorities, constraints and wishes during a check (C) they may adapt the advice, brief interventions or referrals offered to take these into account (e.g. make fewer but more appropriate referrals) (O) because they share the decision with attendees (M)	Data extracted from 12 documents:<br><br>8 research articles (7 qualitative interview studies, 1 Q-methodology study); 1 local evaluation report; 3 conference materials
CMOC P28	When providers are aware of attendees' own priorities, constraints and wishes (C) they may identify and emphasise the benefits of simple changes	Data extracted 7 from documents:

	that are more acceptable and achievable for attendees (O) because they believe this will help attendees (M)	3 research articles (2 qualitative interview studies, 1 pilot RCT); 1 local evaluation report; 3 conference materials

Table S3 Overview of CMOCs focused on NHSHC attendees

	**CMOC**	**Summary of data**
Understanding and engagement with the NHSHC programme: expectations and priorities		
CMOC A1	When attendees understand the NHSHC as a screening opportunity aimed at identifying individuals with disease (C) they may be less likely to engage with advice, brief interventions or offers of referrals (O) because what is offered does not meet their expectations (M)	Data extracted from 8 documents:<br><br>5 research articles (4 qualitative interview studies, 3 local evaluation reports)
CMOC A2	When attendees are already aware of risk factors and/or potential improvements they could make to their lifestyle (C) they may be more receptive to receiving advice, brief interventions or referrals (O) because they are mentally prepared for it (M)	Data extracted from 15 documents:<br><br>8 research articles (6 qualitative interview studies, 1 ethnography, 1 mixed methods); 4 local evaluation reports; 1 PhD (ethnography); 2 conference materials
CMOC A3	When attendees are unaware that they have risk factors for CVD and receive results that indicate that they are at high risk (C) they may need extra support and information from providers (O) because they are shocked and upset (M)	Data extracted from 6 documents:<br><br>5 research articles (4 qualitative interview studies, 1 ethnography); 1 local evaluation report

CMOC A4	In some circumstances attendees who receive results that indicate that they are at high risk may be prompted to make immediate lifestyle changes (O) because they are shocked and upset (M), but the contexts in which this happens (and for whom) are not clear (C undefined)	Data extracted from 8 documents:<br><br>3 research articles (3 qualitative interview studies); 4 local evaluation reports; 1 conference presentation
CMOC A5	When providers are able to explain the implications of risk factors to attendees in a way they can understand (C) attendees may be more receptive to advice, brief interventions or referrals (O) because they appreciate its importance for their own lives (M)	Data extracted from 6 documents:<br><br>1 research article (qualitative interview study); 1 local evaluation report; 1 PhD thesis (interviews/ethnography); 3 conference materials
CMOC A6	When providers are able to link advice, brief interventions and offers of referrals to attendees' own priorities for their health and lifestyle (C) attendees may be more likely to engage with these (O) because they want to achieve these (M)	Data extracted from 10 documents:<br><br>6 research articles (4 qualitative interview studies, 1 co-production study, 1 observational study using video-recordings); 1 research report (RCT); 1 local evaluation report; 1 PhD thesis (interviews/ethnography); 1 conference poster
CMOC A7	When attendees have multiple risk factors (C) they may choose to focus on the advice, brief interventions or referrals offered in relation to those lifestyle changes that are easier to change (O) because they feel it is better than nothing (M)	Data extracted from 3 documents:<br><br>2 research articles (qualitative interview studies); 1 PhD thesis (interviews/ethnography)

CMOC A8	When attendees feel a personal responsibility for their own health and lifestyle (C) they may be unlikely to take up offers of referrals or ongoing support or follow up (O) because they feel obliged to try to make changes on their own (M)	Data extracted from 3 documents:<br><br>2 research articles (2 qualitative interview studies); 1 local evaluation report
CMOC A9	When attendees are not motivated to change their lifestyle or behaviour (C) they are unlikely to engage with advice, brief interventions or take up offers of referrals to lifestyle services (O) because they do not believe they need to, and have other priorities (M)	Data extracted from 8 documents:<br><br>6 research articles (4 qualitative interview studies, 1 RCT, 1 cross-sectional study); 1 research report (mixed methods); 1 local evaluation report
CMOC A10	When attendees are fatalistic about their health (C) they are unlikely to engage with advice, brief interventions or take up offers of referrals to lifestyle services (O) because they think they are pointless (M)	Data extracted from 8 documents:<br><br>4 research articles (4 qualitative interview studies); 1 research report (mixed methods); 1 local evaluation report; 1 conference presentation
CMOC A11	When attendees have health concerns and priorities that fall outside the remit of the NHSHC programme (C) they may be disappointed with the check (O1) and unlikely to engage with advice, brief interventions or take up offers of referrals to lifestyle services (O2) because these do not feel important to them, and they have other priorities (M)	Data extracted from 5 documents:<br><br>2 research articles (2 qualitative interview studies); 1 local evaluation report; 2 PhD theses (ethography, interviews/ethnography).

CMOC A12	When attendees receive an ‘opportunistic’ check (C) they are less likely to (receive and) engage with advice, brief interventions or offers of referrals (O) because they do not understand that this is the purpose of the check (M)	Data extracted from 6 documents:<br><br>3 research articles (3 qualitative interview studies); 2 local evaluation reports; 1 guidance document
CMOC A13	When attendees have the opportunity and time to discuss and ask questions during a check (C) they may be more likely to receive and engage with advice, brief interventions and offers of referrals (O) because they understand that this is the purpose of the check (M)	Data extracted from 14 documents:<br><br>5 research articles (2 qualitative interview studies, 1 ethnography, 1 mixed methods study, 1 observational study using video-recordings); 6 local evaluation reports; 1 research report (mixed methods); 1 PhD thesis (interviews/ethnography)
CMOC A14	When checks are delivered in a non-medical setting (i.e. not in general practice) (C) attendees may be more likely to engage in discussions about risk factors and lifestyle change (O) because they feel relaxed and comfortable (M)	Data extracted from 8 documents:<br><br>3 research articles (1 qualitative interview study, 1 ethnography, 1 protocol for a mixed methods study); 1 local evaluation report; 4 conference materials
Understanding and engagement with the NHSHC programme: credibility and trust		
CMOC A15	When attendees receive advice, brief interventions, offers of referral or prescriptions from a professional they consider to be suitably qualified (C) they may be more likely to engage with or accept these interventions (O) because they consider them to be credible (M)	Data extracted from 10 documents:<br><br>7 research articles (3 qualitative interview studies, 2 surveys, 2 mixed methods studies); 2 local evaluation reports; 1 PhD thesis (interviews/ethnography).

CMOC A16	When attendees receive advice, brief interventions or offers of referral from a provider who seems to understand their circumstances (C) they may be more likely to engage with these interventions (O) because they consider them to be credible (M)	Data extracted from 5 documents:<br><br>1 research article (ethnography); 1 research report (RCT); 1 local evaluation report; 1 conference poster; 1 news article
CMOC A17	When providers can deliver culturally appropriate lifestyle advice (C) attendees may be more likely to engage with it (O) because they consider it to be more credible and relevant (M)	Data extracted from 4 documents:<br><br>1 research article (ethnography); 1 research report (RCT); 1 local evaluation report; 1 conference abstract
CMOC A18	When the measurements and risk assessment completed during a check identify attendees as 'low risk' (C) attendees may be less receptive to any advice, brief interventions or referrals offered (O) because they are reassured and do not think they need to take action (O)	Data extracted from 4 documents:<br><br>3 research articles (1 qualitative interview study, 1 survey, 1 mixed methods study); 1 local evaluation report
CMOC A19	When providers downplay risks or temper advice about lifestyle (C) attendees may feel there is no need to make any changes (O) because they are reassured (M)	Data extracted from 5 documents:<br><br>4 research articles (4 qualitative interview studies); 1 local evaluation report
CMOC A20	When providers are able to convey the importance and urgency of NHSHC risk assessments to attendees (C) attendees may be more receptive to advice, brief interventions or referrals (O) because they feel important (M)	Data extracted from 6 documents:<br><br>3 research articles (3 qualitative interview studies); 1 local evaluation report; 1 PhD thesis (interviews/ethnography); 1 conference presentation

CMOC A21	When the measurements and risk assessment completed during a check identify attendees as 'low risk' (C) attendees' healthy lifestyle habits may be reinforced (O) because they understand these have tangible benefits, reflected in their results (M)	Data extracted from 3 documents:<br><br>2 research articles (1 qualitative interview study, 1 ethnography); 1 PhD thesis (interviews/ethnography)
CMOC A22	When attendees receive measurements and risk assessment results from non-professionals (C1) or do not receive results at all (C2) they may be less likely to consider the results to be important (O) because they trust that providers will alert them to significant results that require action (M)	Data extracted from 4 documents:<br><br>3 research articles (2 qualitative interview studies, 1 survey); 1 PhD thesis (interviews/ethnography)
CMOC A23	When attendees perceive providers to be disengaged with the check (C) they may be less likely to engage with advice, brief interventions or offers of referral (O) because they do not believe these are important (M)	Data extracted from 4 documents:<br><br>2 research articles (1 ethnography, 1 survey); 1 PhD thesis (ethnography); 1 conference presentation
CMOC A24	When attendees receive advice, brief interventions or offers of referral from a provider who does not seem to 'practice what they preach' (C) they may be less likely to engage with these interventions (O) because they consider these to lack credibility (M)	Data extracted from 2 documents:<br><br>1 research article (qualitative interview study); 1 local evaluation report
CMOC A25	When attendees receive advice or information relating to healthy lifestyles that they already familiar with during a check (C) they may perceive the advice to be useless (O) because they have heard it before (M)	Data extracted from 8 documents:<br><br>6 research articles (4 qualitative interview studies, 1 ethnography, 1 mixed methods study); 2 PhD theses (ethnography, interviews/ethnography)

CMOC A26	When attendees learn something new and important to them during a check (C) they may attempt to make changes to their lifestyle in light of this (O) because they have an improved understanding of risk factors or lifestyle advice (M)	Data extracted from 6 documents:<br><br>3 research articles (1 qualitative interview study, 1 ethnography, 1 survey); 1 research report (RCT); 1 local evaluation report; 1 PhD thesis (interviews/ethnography)
CMOC A27	When attendees are aware of conflicting or inconsistent guidance about healthy lifestyles (C) they may reject or ignore advice delivered during a check (O) because they doubt its credibility (M1) or are confused (M2)	Data extracted from 4 documents:<br><br>2 research articles (2 qualitative interview studies); 1 research report (RCT); 1 PhD thesis (ethnography)
CMOC A28	When attendees are aware of controversy in relation to recommended medication (statins) (C) they may be ambivalent about accepting or adhering to a prescription (O) because they are uncertain of the benefits (M)	Data extracted from 5 documents:<br><br>3 research articles (3 qualitative interview studies; 1 local evaluation reports; 1 conference presentation
CMOC A29	When attendees have doubts about the accuracy of the tests and tools used to measure and assess risk during a check (C) they may be less receptive to advice, brief interventions, referrals or prescriptions offered to address identified risks (O) because they are unsure that the assessments are credible (M)	Data extracted from 6 documents:<br><br>2 research articles (1 qualitative interview study, 1 mixed methods study); 1 research report (mixed methods study); 2 local evaluation reports; 1 PhD thesis (interviews/ethnography)
Practical constraints: referrals and follow-up		

CMOC A30	When attendees receive continuity of care and follow up after a check (C) they may be more likely to engage with advice, take up referrals or attempt to make lifestyle changes (O) because they are reminded of what they are meant to be doing and why (M)	Data extracted from 10 documents:<br><br>5 research articles (4 qualitative interview studies, 1 pilot RCT); 1 research report (RCT); 2 local evaluation reports; 1 PhD thesis (interviews/ethnography); 1 conference poster
CMOC A31	When attendees receive continuity of care and follow up after a check (C) they may be more likely to engage with advice, take up referrals or attempt to make lifestyle changes (O) because they feel supported and valued (M)	Data extracted from 5 documents:<br><br>2 research articles (2 qualitative interview studies); 1 research reports (RCT); 1 local evaluation report; 1 PhD thesis (ethnography)
CMOC A32	When attendees are repeatedly offered follow up and feedback on progress after a check (C) they may be motivated to attempt and maintain lifestyle changes (O) because they can monitor their progress (M)	Data extracted from 6 documents:<br><br>3 research articles (3 qualitative interview studies); 1 research report (RCT); 2 local evaluation reports
CMOC A33	When attendees can see tangible benefits of making lifestyle changes after a check (C) they are more likely to maintain these (O) because they are motivated to continue (M)	Data extracted from 5 documents:<br><br>1 research article (qualitative interview study); 2 local evaluation reports; 2 conference materials

CMOC A34	When attendees are not offered any follow up (beyond the five-year NHSHC programme cycle) (C) they may lack motivation to attempt any lifestyle changes (O) because they interpret the absence of follow up to mean there is no urgent need to make changes (M)	Data extracted from 3 documents:<br><br>1 research article (survey); 1 research report (RCT); 1 PhD thesis (interviews/ethnography)
CMOC A35	When attendees don't consider local lifestyle services to be convenient, appropriate or likely to meet their needs (C) they are less likely to take up referrals or attend these services (O) because they feel it is pointless (M)	Data extracted from 14 documents:<br><br>9 research articles (7 qualitative interviews studies, 1 systematic review, 1 mixed methods study); 2 local evaluation reports; 2 PhD theses (ethnography, interviews/ethnography); 1 conference abstract
CMOC A36	When local lifestyle support services are designed to be more accessible (e.g. in terms of timing, location, cost) (C) attendees may be more likely to start and continue to attend (O) because they feel they are more convenient, affordable or relevant (M)	Data extracted from 4 documents:<br><br>3 research articles (3 qualitative interview studies); 1 conference poster
CMOC A37	When attendees have the option to try out a lifestyle service or are supported to try one by a provider (C) they may be more likely to take up an offer of a referral (O) because they feel more confident to do so (M)	Data extracted from 3 documents:<br><br>1 research article (pilot RCT); 1 local evaluation report; 1 conference abstract
Practical constraints for attendees: person-centredness		

CMOC A38	When attendees receive advice about healthy lifestyles that does not take account of their personal circumstances (C) they are less likely to engage with it (O) because they believe it is unworkable for them (M)	Data extracted from 10 documents:<br><br>8 research articles (7 qualitative interview studies, 1 mixed methods study); 1 PhD thesis (ethnography); 1 conference presentation
CMOC A39	When attendees receive advice about healthy lifestyles that they believe they cannot achieve (e.g. because it seems to require big changes) (C) they are less likely to engage with it (O) because they feel overwhelmed and hopeless (M)	Data extracted from 3 documents:<br><br>3 research articles (2 qualitative interview studies, 1 observational study using video recordings)
CMOC A40	When attendees receive advice about healthy lifestyles that attendees feel they can fit into their lives (e.g. around other commitments) (C) they may be more likely to engage with it (O) because they perceive the changes to be less disruptive (M)	Data extracted from 13 documents:<br><br>6 research articles (4 qualitative interview studies, 1 ethnography, 1 pilot RCT); 1 research report (RCT); 5 local evaluation reports; 1 conference poster
CMOC A41	When attendees feel that significant lifestyle change is unworkable for them (C) they may be more likely to accept prescriptions (e.g. for statins) (O) because they still want to do something to reduce their CVD risk (M)	Data extracted from 2 documents:<br><br>1 research article (qualitative interview study); 1 local evaluation report
CMOC A42	When attendees anticipate or experience medication side effects or burdens (C) they may be more ambivalent about accepting or adhering to prescriptions (O) because they are uncertain of the benefits and concerned about harms (M)	Data extracted from 4 documents:<br><br>2 research articles (2 qualitative interview studies); 1 local evaluation report; 1 conference presentation

CMOC A43	When attendees anticipate or experience medication side effects or burdens (C) they attempt lifestyle change (O) because they prefer this option (M)	Data extracted from 3 documents:<br><br>2 research articles (2 qualitative interview studies); 1 local evaluation report
CMOC A44	When attendees are encouraged and supported by friends, family or peers to make and sustain lifestyle changes (C) they may be more likely to attempt and maintain changes (O) but the mechanism for this is unclear (M not defined)	Data extracted from 11 documents:<br><br>7 research articles (5 qualitative interview studies, 1 ethnography, 1 co-production study); 1 research report (RCT); 1 local evaluation report; 2 PhD theses (ethnography, interviews/ethnography)
