# Supplementary material for: Understanding what happens to attendees after an NHS Health Check: a realist review
Source: BMJ Open. 2022 Nov 10;12(11):e064237. doi: 10.1136/bmjopen-2022-064237 (PMC9660666; doi:10.1136/bmjopen-2022-064237)
Supplement: Supplementary data [file bmjopen-2022-064237supp004.pdf]

# Supplementary File 1

This file contains a glossary of realist terminology, a diagram summarising our initial programme theory and the focus of the realist review and PRISMA and RAMESES checklists.

## Glossary of realist terminology

**Context**: in realist research, the conditions or circumstances in which mechanisms that generate observed outcomes are ‘triggered’ or activated.

**Context-mechanism-outcome configuration (CMOC)**: a heuristic used to present a realist causal explanation for an outcome, presented as a relationship between some particular context(s) and mechanism(s).

**Demi-regularity**: a semi-predictable pattern of outcomes that occur in the same context(s).

**Mechanism**: in realist research, the underlying context-sensitive causal force that generates an outcome, often conceptualised as the response of an individual actor to important context(s).

**Programme theory**: a set of theoretical explanations about how a programme, intervention or process is understood to work. Realist programme theories explain or the process by which outcomes of interest are thought to be generated, using causal explanations captured in the form of CMOCs.

**Substantive theory**: an established, formal theory drawn from any discipline that can be used to help understand the programme, intervention or process under examination.

## Initial programme theory diagram

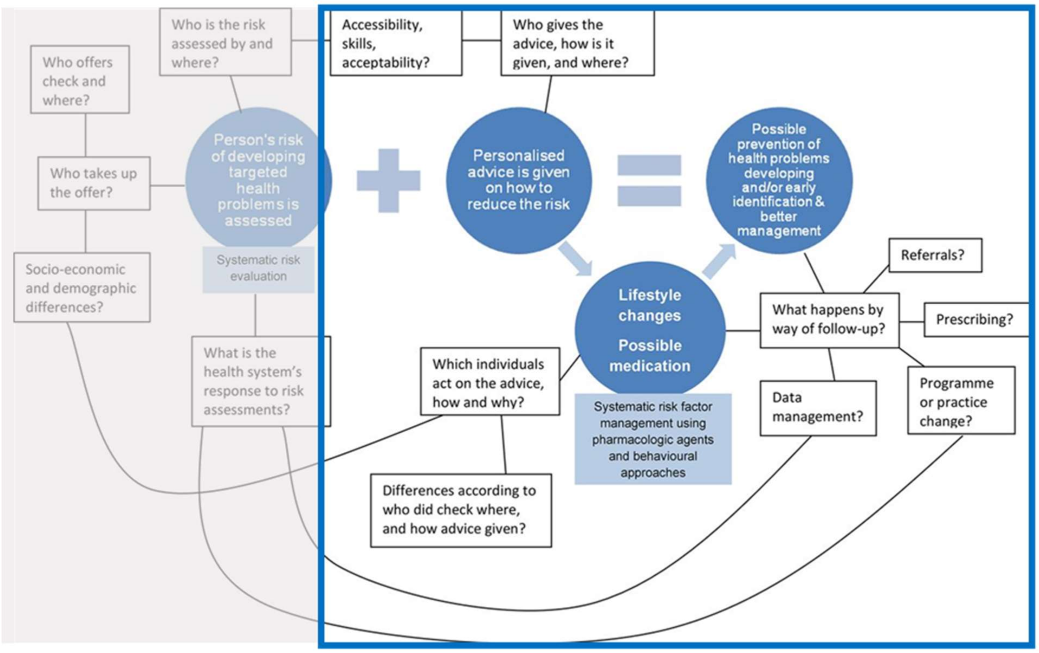


*Initial programme theory for the realist review with project focus highlighted*

# PRISMA checklist

## Reporting checklist for systematic review (with or without a meta-analysis).

Based on the PRISMA guidelines.

## Instructions to authors

Complete this checklist by entering the page numbers from your manuscript where readers will find each of the items listed below.

Your article may not currently address all the items on the checklist. Please modify your text to include the missing information. If you are certain that an item does not apply, please write "n/a" and provide a short explanation.

Upload your completed checklist as an extra file when you submit to a journal.

In your methods section, say that you used the PRISMAreporting guidelines, and cite them as:

Page MJ, McKenzie JE, Bossuyt PM, Boutron I, Hoffmann TC, Mulrow CD, Shamseer L, Tetzlaff JM, Akl EA, Brennan SE, Chou R, Glanville J, Grimshaw JM, Hróbjartsson A, Lalu MM, Li T, Loder EW, Mayo-Wilson E, McDonald S, McGuinness LA, Stewart LA, Thomas J, Tricco AC, Welch VA, Whiting P, Moher D. The PRISMA 2020 statement: An updated guideline for reporting systematic reviews

| | | Reporting Item | Page Number |
|---|---|---|---|
| **Title** | | | |
| Title | #1 | Identify the report as a systematic review | 1 |
| **Abstract** | | | |
| Abstract | #2 | Report an abstract addressing each item in the PRISMA 2020 for Abstracts checklist | 2 |
| **Introduction** | | | |
| Background/rationale | #3 | Describe the rationale for the review in the context of existing knowledge | 3-6 |
| Objectives | #4 | Provide an explicit statement of the objective(s) or question(s) the review addresses | 6, protocol |
| **Methods** | | | |

| | | | |
|---|---|---|---|
| Eligibility criteria | #5 | Specify the inclusion and exclusion criteria for the review and how studies were grouped for the syntheses | 8-9, protocol |
| Information sources | #6 | Specify all databases, registers, websites, organisations, reference lists, and other sources searched or consulted to identify studies. Specify the date when each source was last searched or consulted | 8, Supplementary File 2 |
| Search strategy | #7 | Present the full search strategies for all databases, registers, and websites, including any filters and limits used | Supplementary File 2 |
| Selection process | #8 | Specify the methods used to decide whether a study met the inclusion criteria of the review, including how many reviewers screened each record and each report retrieved, whether they worked independently, and, if applicable, details of automation tools used in the process | 8, Table 1 and protocol |
| Data collection process | #9 | Specify the methods used to collect data from reports, including how many reviewers collected data from each report, whether they worked independently, any processes for obtaining or confirming data from study investigators, and, if applicable, details of automation tools used in the process | 8-9, Table 1 and protocol |
| Data items | #10a | List and define all outcomes for which data were sought. Specify whether all results that were compatible with each outcome domain in each study were sought (for example, for all measures, time points, analyses), and, if not, the methods used to decide which results to collect | n/a |

| | | | |
|---|---|---|---|
| Study risk of bias assessment | #11 | Specify the methods used to assess risk of bias in the included studies, including details of the tool(s) used, how many reviewers assessed each study and whether they worked independently, and, if applicable, details of automation tools used in the process | n/a |
| Effect measures | #12 | Specify for each outcome the effect measure(s) (such as risk ratio, mean difference) used in the synthesis or presentation of results | n/a |
| Synthesis methods | #13a | Describe the processes used to decide which studies were eligible for each synthesis (such as tabulating the study intervention characteristics and comparing against the planned groups for each synthesis (item #5)) | 8-9, Table 1, protocol |
| Synthesis methods | #13b | Describe any methods required to prepare the data for presentation or synthesis, such as handling of missing summary statistics or data conversions | n/a |
| Synthesis methods | #13c | Describe any methods used to tabulate or visually display results of individual studies and syntheses | n/a |
| Synthesis methods | #13d | Describe any methods used to synthesise results and provide a rationale for the choice(s). If meta-analysis was performed, describe the model(s), method(s) to identify the presence and extent of statistical heterogeneity, and software package(s) used | 8-9, Table 1, protocol |
| Synthesis methods | #13e | Describe any methods used to explore possible causes of heterogeneity among study results (such as subgroup analysis, meta-regression) | n/a |

| | | | |
|---|---|---|---|
| Synthesis methods | #13f | Describe any sensitivity analyses conducted to assess robustness of the synthesised results | n/a |
| Reporting bias assessment | #14 | Describe any methods used to assess risk of bias due to missing results in a synthesis (arising from reporting biases) | n/a |
| Certainty assessment | #15 | Describe any methods used to assess certainty (or confidence) in the body of evidence for an outcome | n/a |
| Data items | #10b | List and define all other variables for which data were sought (such as participant and intervention characteristics, funding sources). Describe any assumptions made about any missing or unclear information | n/a |
| **Results** | | | |
| Study selection | #16a | Describe the results of the search and selection process, from the number of records identified in the search to the number of studies included in the review, ideally using a flow diagram (http://www.prisma-statement.org/PRISMAStatement/FlowDiagram) | 9, Figure 1 |
| Study selection | #16b | Cite studies that might appear to meet the inclusion criteria, but which were excluded, and explain why they were excluded | n/a |
| Study characteristics | #17 | Cite each included study and present its characteristics | Supplementary File 3 |
| Risk of bias in studies | #18 | Present assessments of risk of bias for each included study | n/a |
| Results of individual studies | #19 | For all outcomes, present for each study (a) summary statistics for each group (where appropriate) and (b) an effect estimate and its | n/a |

| | | | |
|---|---|---|---|
| | | precision (such as confidence/credible interval), ideally using structured tables or plots | |
| Results of syntheses | #20a | For each synthesis, briefly summarise the characteristics and risk of bias among contributing studies | n/a |
| Results of syntheses | #20b | Present results of all statistical syntheses conducted. If meta-analysis was done, present for each the summary estimate and its precision (such as confidence/credible interval) and measures of statistical heterogeneity. If comparing groups, describe the direction of the effect | n/a |
| Results of syntheses | #20c | Present results of all investigations of possible causes of heterogeneity among study results | n/a |
| Results of syntheses | #20d | Present results of all sensitivity analyses conducted to assess the robustness of the synthesised results | n/a |
| Risk of reporting biases in syntheses | #21 | Present assessments of risk of bias due to missing results (arising from reporting biases) for each synthesis assessed | n/a |
| Certainty of evidence | #22 | Present assessments of certainty (or confidence) in the body of evidence for each outcome assessed | n/a |
| **Discussion** | | | |
| Results in context | #23a | Provide a general interpretation of the results in the context of other evidence | 16-18 |
| Limitations of included studies | #23b | Discuss any limitations of the evidence included in the review | 20-21 |
| Limitations of the review methods | #23c | Discuss any limitations of the review processes used | 20-21 |

| | | | |
|---|---|---|---|
| Implications | #23d | Discuss implications of the results for practice, policy, and future research | 19-20 |
| **Other information** | | | |
| Registration and protocol | #24a | Provide registration information for the review, including register name and registration number, or state that the review was not registered | 3 |
| Registration and protocol | #24b | Indicate where the review protocol can be accessed, or state that a protocol was not prepared | 6, 8 |
| Registration and protocol | #24c | Describe and explain any amendments to information provided at registration or in the protocol | 8-9 |
| Support | #25 | Describe sources of financial or non-financial support for the review, and the role of the funders or sponsors in the review | 23 |
| Competing interests | #26 | Declare any competing interests of review authors | 23 |
| Availability of data, code, and other materials | #27 | Report which of the following are publicly available and where they can be found: template data collection forms; data extracted from included studies; data used for all analyses; analytic code; any other materials used in the review | 23 |

Notes:

- 4: 6, protocol
- 5: 8-9, protocol
- 6: 8, Supplementary File 2
- 7: Supplementary File 2
- 8: 8, Table 1 and protocol

- 9: 8-9, Table 1 and protocol
- 13a: 8-9, Table 1, protocol
- 13d: 8-9, Table 1, protocol
- 16a: 9, Figure 1
- 17: Supplementary File 3 The PRISMA checklist is distributed under the terms of the Creative Commons Attribution License CC-BY. This checklist was completed on 25. April 2022 using https://www.goodreports.org/, a tool made by the EQUATOR Network in collaboration with Penelope.ai

## RAMESES checklist

List of items when reporting a realist synthesis

| Reporting item | | Description of item | Reported on page(s): |
|---|---|---|---|
| TITLE | | | |
| 1 | | In the title, identify the document as a realist synthesis or review | 1 |
| ABSTRACT | | | |
| 2 | | While acknowledging publication requirements and house style, abstracts should ideally contain brief details of: the study's background, review question or objectives; search strategy; methods of selection, appraisal, analysis and synthesis of sources; main results; and implications for practice. | 2 |
| INTRODUCTION | | | |
| 3 | Rationale for review | Explain why the review is needed and what it is likely to contribute to existing understanding of the topic area. | 5-6 |
| 4 | Objectives and focus of review | State the objective(s) of the review and/or the review question(s). Define and provide a rationale for the focus of the review. | 5-6 |
| METHODS | | | |
| 5 | Changes in the review process | Any changes made to the review process that was initially planned should be briefly described and justified. | 9 |
| 6 | Rationale for using realist synthesis | Explain why realist synthesis was considered the most appropriate method to use. | 6 |
| 7 | Scoping the literature | Describe and justify the initial process of exploratory scoping of the literature. | Table 1, page 8 Supplementary File 1 |
| 8 | Searching processes | While considering specific requirements of the journal or other publication outlet, state and provide a rationale for how the iterative searching was done. Provide details on all the sources accessed for information in the review. Where searching in electronic databases has taken place, the details should include, for example, name of database, search terms, dates of coverage and date last searched. If individuals | Table 1, page 8 Supplementary File 2 |

| | | | |
|---|---|---|---|
| | | familiar with the relevant literature and/or topic area were contacted, indicate how they were identified and selected. | |
| 9 | Selection and appraisal of documents | Explain how judgements were made about including and excluding data from documents, and justify these. | Table 1, page 8 |
| 10 | Data extraction | Describe and explain which data or information were extracted from the included documents and justify this selection. | Table 1, page 8 |
| 11 | Analysis and synthesis processes | Describe the analysis and synthesis processes in detail. This section should include information on the constructs analyzed and describe the analytic process. | Table 1, page 9 |
| RESULTS | | | |
| 12 | Document flow diagram | Provide details on the number of documents assessed for eligibility and included in the review with reasons for exclusion at each stage as well as an indication of their source of origin (for example, from searching databases, reference lists and so on). You may consider using the example templates (which are likely to need modification to suit the data) that are provided. | Figure 1, page 10 |
| 13 | Document characteristics | Provide information on the characteristics of the documents included in the review. | Supplementary File 3 |
| 14 | Main findings | Present the key findings with a specific focus on theory building and testing. | 10-16 |
| DISCUSSION | | | |
| 15 | Summary of findings | Summarize the main findings, taking into account the review's objective(s), research question(s), focus and intended audience(s). | 17-19 |
| 16 | Strengths, limitations and future research directions | Discuss both the strengths of the review and its limitations. These should include (but need not be restricted to) (a) consideration of all the steps in the review process and (b) comment on the overall strength of evidence supporting the explanatory insights which emerged.<br>The limitations identified may point to areas where further work is needed. | 22 |
| 17 | Comparison with existing literature | Where applicable, compare and contrast the review's findings with the existing literature (for example, other reviews) on the same topic. | 17-18 |
| 18 | Conclusion and recommendations | List the main implications of the findings and place these in the context of other relevant literature. If appropriate, offer recommendations for policy and practice. | 19-23 |

| | | | |
|---|---|---|---|
| 19 | Funding | Provide details of funding source (if any) for the review, the role played by the funder (if any) and any conflicts of interests of the reviewers. | 24 |
