# Supplementary material for: Understanding what happens to attendees after an NHS Health Check: a realist review
Source: BMJ Open. 2022 Nov 10;12(11):e064237. doi: 10.1136/bmjopen-2022-064237 (PMC9660666; doi:10.1136/bmjopen-2022-064237)
Supplement: Author's manuscript [file bmjopen-2022-064237.draft_revisions.pdf]

BMJ Open

BMJ Open is committed to open peer review. As part of this commitment we make the peer review history of every article we publish publicly available.

When an article is published we post the peer reviewers' comments and the authors' responses online. We also post the versions of the paper that were used during peer review. These are the versions that the peer review comments apply to.

The versions of the paper that follow are the versions that were submitted during the peer review process. They are not the versions of record or the final published versions. They should not be cited or distributed as the published version of this manuscript.

BMJ Open is an open access journal and the full, final, typeset and author-corrected version of record of the manuscript is available on our site with no access controls, subscription charges or pay-per-view fees (http://bmjopen.bmj.com).

If you have any questions on BMJ Open's open peer review process please email info.bmjopen@bmj.com

BMJ Open

Understanding what happens to attendees after an NHS Health Check: a realist review

Journal:	*BMJ Open*
Manuscript ID	bmjopen-2022-064237
Article Type:	Original research
Date Submitted by the Author:	26-Apr-2022
Complete List of Authors:	Duddy, Claire; University of Oxford, Nuffield Department of Primary Care Health Sciences<br>Gadsby, Erica; University of Stirling, Faculty of Health Sciences and Sport<br>Hibberd, Vivienne; University of Greenwich Medway School of Pharmacy, Public Involvement in Pharmacy Studies Group; University of Kent Medway School of Pharmacy<br>Krska, Janet; University of Greenwich Medway School of Pharmacy; University of Kent Medway School of Pharmacy<br>Wong, Geoff; Oxford University, Nuffield Department of Primary Care Health Sciences
Keywords:	PUBLIC HEALTH, CARDIOLOGY, Health policy < HEALTH SERVICES ADMINISTRATION & MANAGEMENT, Risk management < HEALTH SERVICES ADMINISTRATION & MANAGEMENT, PREVENTIVE MEDICINE

SCHOLARONE™
Manuscripts

I, the Submitting Author has the right to grant and does grant on behalf of all authors of the Work (as defined in the below author licence), an exclusive licence and/or a non-exclusive licence for contributions from authors who are: i) UK Crown employees; ii) where BMJ has agreed a CC-BY licence shall apply, and/or iii) in accordance with the terms applicable for US Federal Government officers or employees acting as part of their official duties; on a worldwide, perpetual, irrevocable, royalty-free basis to BMJ Publishing Group Ltd ("BMJ") its licensees and where the relevant Journal is co-owned by BMJ to the co-owners of the Journal, to publish the Work in this journal and any other BMJ products and to exploit all rights, as set out in our licence.

The Submitting Author accepts and understands that any supply made under these terms is made by BMJ to the Submitting Author unless you are acting as an employee on behalf of your employer or a postgraduate student of an affiliated institution which is paying any applicable article publishing charge ("APC") for Open Access articles. Where the Submitting Author wishes to make the Work available on an Open Access basis (and intends to pay the relevant APC), the terms of reuse of such Open Access shall be governed by a Creative Commons licence – details of these licences and which Creative Commons licence will apply to this Work are set out in our licence referred to above.

Other than as permitted in any relevant BMJ Author's Self Archiving Policies, I confirm this Work has not been accepted for publication elsewhere, is not being considered for publication elsewhere and does not duplicate material already published. I confirm all authors consent to publication of this Work and authorise the granting of this licence.

For peer review only

Understanding what happens to attendees after an NHS Health Check: a realist review

Claire Duddy[1*], Erica Gadsby[2], Vivienne Hibberd[3], Janet Krska[4], Geoff Wong[1]

[1] Nuffield Department of Primary Care Health Sciences, University of Oxford, Oxford, UK

[2] Faculty of Health Sciences and Sport, University of Stirling, Stirling, UK and Centre for Health Services Studies, University of Kent, Canterbury, UK.

[3] Public Involvement in Pharmacy Studies Group, Medway School of Pharmacy, Universities of Greenwich and Kent, Chatham Maritime, UK

[4] Medway School of Pharmacy, Universities of Greenwich and Kent, Chatham Maritime, UK.

*Corresponding author

claire.duddy@phc.ox.ac.uk

01865 617855

Radcliffe Primary Care Building

Radcliffe Observatory Quarter

Woodstock Road

Oxford OX2 6GG.

Word count: **5787** (excluding title page, abstract, references, tables, acknowledgements)

For peer review only - http://bmjopen.bmj.com/site/about/guidelines.xhtml

Abstract

Objectives

The NHS Health Check offers adults aged 40-74 an assessment of their risk of developing cardiovascular disease. Attendees should be offered appropriate clinical or behavioural interventions to help them to manage or reduce these risks. This project focused on understanding variation in the advice and support offered to Health Check attendees.

Design

We conducted a realist review, examining existing evidence to explain the causes of observed outcomes. We gathered a diverse body of research and grey literature, and synthesised data extracted from documents using a realist logic of analysis. Our aim was to develop an understanding of contexts affecting delivery of the NHS Health Check and the underlying mechanisms producing outcomes related to the offer for attendees post-Check.

Results

Our findings demonstrate differences in how NHS Health Check commissioners, providers and attendees understand the primary purpose of the programme. A focus on screening for disease can produce an emphasis on high-volume delivery in primary care. When delivery models are organised around behavioural approaches to risk reduction, more emphasis is placed on advice, and referrals to 'lifestyle services'. However, constrained funding and competing priorities for providers limit what can be delivered within the programme's remit. Attendees' experiences and responses to the programme are affected by how the programme is delivered, and by the difficulty of incorporating its outputs into their lives.

Conclusions

The remit of the NHS Health Check should be reviewed with consideration of what can be effectively delivered within existing resources. Variation in delivery may be appropriate to meet local needs, but differences in how the programme's primary purpose is understood contributes to a 'postcode lottery' in post-Check advice and support. Our findings underline existing concerns that the programme may

For peer review only

generate inequitable outcomes and raise questions about whether it can deliver positive outcomes for the majority of attendees.

Study registration

PROSPERO CRD42020163822

Keywords

cardiovascular diseases, prevention, public health, lifestyle, health promotion, screening, realist review

Article Summary

Strengths and limitations of this study

- This is the first realist review focused on the NHS Health Check programme and it has generated new understanding of the causes of variation in delivery at the end of the Health Check pathway;
- The review is inclusive of a wide range of literature, including grey literature, allowing us to draw on learning from local delivery models;
- This project was strengthened by strong and consistent PPI and professional stakeholder input, which shaped and tested the relevance of our theories throughout;
- As with any review, our findings were limited by the availability and quality of the available evidence – we sought to fill a gap in the existing literature focused on the end of the Health Check pathway but this necessarily limited the number and quality of documents available.

Background

The NHS Health Check in England is a large-scale public health programme that aims to offer all adults (aged 40-74) a five-yearly assessment of their risk of developing cardiovascular disease (CVD). The NHS Health Check involves the measurement of multiple CVD risk factors and the delivery of advice and discussion of appropriate clinical and behavioural approaches that might help individuals to manage and reduce their CVD risk. These could include, for example, referral to a GP to discuss recommended pharmacological options (generally statins or antihypertensives) or the delivery of

For peer review only - http://bmjopen.bmj.com/site/about/guidelines.xhtml

advice, signposting or formal referral to 'lifestyle' services, such as smoking cessation and weight management programmes.(1)

The programme is concerned with both the early identification of risk factors for CVD and the delivery of early interventions to address them, via both pharmacological and non-pharmacological, 'lifestyle' or 'behavioural' means. It is also tasked with helping to address population-level health disparities, reflecting an understanding that the burden of CVD is not spread equally throughout the population, but is strongly linked to factors including deprivation and ethnicity. The intention to use the programme to address health inequalities has been present since its launch; most recently, the programme is cited in the UK Government's recent 'Levelling Up' white paper as a potential means of delivering its prevention agenda.(2) To meet its aims of preventing CVD at a population level, and contributing to reducing the health inequalities associated with CVD, the NHS Health Check must be widely taken up (especially by those with most to gain), and be effective in both assessing and reducing or managing any risks identified.

The NHS Health Check was first introduced in 2009 but the programme was relaunched in 2013, when responsibility for commissioning the programme transferred from NHS Primary Care Trusts (PCTs) to local authorities (LAs). Minimum standards for programme delivery – focused on the collection of certain measurements and targets to increase the volume of checks delivered - became statutory requirements at this time(3, 4) and Public Health England (PHE) was formed and given oversight of the programme. In 2021, the new Office for Health Improvement and Disparities (OHID) took on responsibility for the programme and published a major review (instigated and conducted by PHE), setting out an updated 'vision' for the NHS Health Check. The review's recommendations include aims to launch a digital service and to increase the size and remit of the programme by extending it to cover younger age groups and to address more conditions beyond CVD.(5)

The NHS Health Check has long been the subject of controversy, with some calling its effectiveness and cost-effectiveness into question,(6, 7, 8, 9) and PHE responding by collating evidence that supports the programme under the auspices of the Expert Scientific and Clinical Advisory Panel (ESCAP).(10) Observational studies included in two PHE-commissioned rapid reviews suggest that

For peer review only - http://bmjopen.bmj.com/site/about/guidelines.xhtml

the programme is associated with increased rates of CVD risk factor and disease detection, statin prescribing and referrals to 'lifestyle services' (including smoking cessation, weight management, exercise and alcohol support services). Work undertaken to support the recent PHE/OHID review of the programme has also identified an association between Health Check attendance and improvement in a range of indicators, including: BMI, rates of smoking, blood pressure, total cholesterol, hospital admissions related to CVD or type 2 diabetes, and all-cause death, after five years,(11) although the direction of causality is unclear.

Regional and local studies of the implementation and outcomes of the programme demonstrate wide variation in programme delivery and outcomes across England. Evidence on the delivery of advice, referrals and on behaviour changes post-Health Check is especially sparse. The rapid reviews identified only six primary studies examining behaviour change, all focused only on smoking cessation.(12, 13)

Our scoping searches and an initial review of the existing research evidence relating to the programme identified a clear focus on invitation, uptake and coverage of the NHS Health Check. Far less attention has been paid to what happens after the measurements and risk assessments have been undertaken, especially in relation to the delivery of advice, onward signposting or referral and ongoing support for behaviour changes that might reduce CVD risks. The capacity of the NHS Health Check to provide attendees with appropriate advice, referrals and support – and the extent to which attendees respond to these – is a critical assumption underpinning the effectiveness of the programme.

Current best practice guidance makes recommendations for NHS Health Check commissioners and providers, describing a range of possible advice and referral options that may be made available, as well as clinical interventions that may be appropriate to offer attendees.(1) It is clear that responsibility for clinical follow up rests with primary care, and the guidance suggests that commissioners "may wish to" put referral pathways to onward services, such as smoking cessation services, in place. Patel et al's large-scale observational study of the NHS Health Check for the period 2012-2017 includes data relating to the provision of "advice, information or referral" for different risk factors that may be flagged during a check.(14) Although data recording for these

For peer review only - http://bmjopen.bmj.com/site/about/guidelines.xhtml

activities is likely to be incomplete, the figures presented in this study suggest that there is wide variation in the delivery and recording of these activities for different risk factors, and that rates of delivery fall well below the recommended thresholds for intervention. For example, only one quarter of attendees recorded as meeting the threshold for intervention are recorded as receiving advice, information or referral in relation to diet. In addition, this national-level study obscures the wide regional variation in what they term 'post-delivery management' following a check, identified in existing reviews that include local studies.(12, 13)

In consultation with our stakeholder groups (see Methods below for more details on group membership) we focused our review on these final steps in the NHS Health Check pathway. Our aim was to develop an understanding of how the NHS Health Check programme works in different settings, and for different groups to achieve its outcomes, with a specific focus on what happens after the measurements and risk assessment are complete.

Methods

We conducted a realist review to synthesise evidence that could help us to develop an understanding of the important contexts that influence the delivery of the NHS Health Check and the mechanisms that produce intended and unintended outcomes. Realist review is an interpretive, theory-driven approach, chosen because existing research clearly demonstrates that the NHS Health Check programme is a complex intervention with context-sensitive outcomes. Our realist analysis used data extracted from the literature to develop context-mechanism-outcome configurations (CMOCs). CMOCs are causal explanations that describe why and how (by which mechanisms) particular outcomes are generated in particular contexts. The NHS Health Check programme is characterised by wide variation in commissioning and delivery and hence in the experience of attendees. Our review sought to make sense of this variation, by developing a realist programme theory, based on CMOCs, that elucidates the causes of outcomes related to the final steps in the patient pathway, i.e. on what happens after the measurements and risk assessments are complete.

For peer review only - http://bmjopen.bmj.com/site/about/guidelines.xhtml

Our methods are described in brief below, while full details are available in a detailed protocol paper.(15) The conduct and reporting of the review followed the RAMESES quality(16) and reporting standards.(17) A glossary of realist terminology is included in **Supplementary File 1**.

Patient and public involvement

Our review was informed by the involvement of two stakeholder groups, who provided us with content expertise based on their lived experience of commissioning, providing or receiving (or being eligible to receive) the NHS Health Check. Our patient and public involvement (PPI) group comprised 10 members of the public from 6 English regions and were selected to be as diverse as possible in relation to age, gender, ethnicity and geography. Our 'professional' stakeholder group were recruited via our existing networks and snowballing, and included policy makers, LA commissioners, NHS Health Check providers and a trainer, and representatives from relevant health charities. Our final meeting with the latter group was expanded to include 36 individuals, to ensure a broad range of feedback on our findings and recommendations for policy and practice.

Both groups were consulted via regular online meetings throughout the project. We asked these groups to provide feedback and responses to our emerging findings as the project developed. For example, we asked them to reflect on how our findings fit with (or did not fit with) their own knowledge and experience of commissioning, providing or attending an NHS Health Check. These discussions helped to shape our analysis, highlight areas of importance to different groups and inform the development of our recommendations. Our PPI group consistently observed a 'mismatch' between the focus of the NHS Health Check programme and their own health priorities, and a lack of understanding of the programme's purpose. This informed our analysis of data relating to attendees' understanding of their health needs and our overall focus on different groups' understanding of the purpose of the programme.

In our final meetings with each group, we asked our stakeholders for their input to help us to develop and refine recommendations to inform NHS Health Check policy, commissioning and delivery. We

For peer review only - http://bmjopen.bmj.com/site/about/guidelines.xhtml

also sought their input to help us to develop appropriate dissemination strategies, tailored project outputs and important audiences to share our findings.

Realist review methods

Our realist review followed Pawson's five iterative steps(18), as described in detail in our protocol.(15) These steps are summarised in **Table 1** below. We used the PRISMA checklist (where applicable) when writing our report (see **Supplementary File 1** for details).(19)

Table 1: Summary of methods

	**Aim**	**Approach**
Step 1: Locate existing theories	To identify existing theories that offer explanations from when, how and for whom the NHS Health Checks programme 'works'<br><br>See **Supplementary File 1**	Informal literature searching, focused on existing reviews and programme documentation<br>Drawing on experience and knowledge of project team and stakeholders
Step 2: Search for evidence	To gather a body of literature containing data that could be used to refine the initial programme theory	Screening material included in two existing reviews(12, 13)<br>Additional searches across multiple databases (see **Supplementary File 2**)<br>Trawling NHS Health Check website for grey literature<br>Citation chaining<br>Email alert to capture new material
Step 3: Article selection	Within the material identified as described above, to select documents that could contribute relevant data for theory development	Screening title and abstract and then in full text<br>Inclusion of documents based on assessment of relevance and rigour(20)<br>10% of records screened in duplicate, with discrepancies resolved by discussion
Step 4: Extracting and organising data	To describe and organise included documents<br>To extract and code relevant data from included documents and begin analysis<br><br>See **Supplementary File 3.** The full set of extracted data is available on request.	Key characteristics of documents captured in an Excel spreadsheet<br>Relevant data related to our research question and focus coded in NVivo<br>10% of records coded in duplicate and checked by a third reviewer
Step 5: Synthesising evidence and drawing conclusions	To apply a realist logic of analysis to extracted data to develop context-mechanism-outcome configurations (CMOCs) related to the end of the NHS Health Check pathway<br>To develop a final programme theory	Close examination and interpretation of data coded within each category to build CMOCs.<br>Cross-case comparison to identify data that demonstrated similar or contrasting contexts and mechanisms that produced patterns of outcomes.<br>Iterative development of CMOCs as more data was considered and discussed by the project team and stakeholder groups

For peer review only - http://bmjopen.bmj.com/site/about/guidelines.xhtml

As the project progressed, we made some minor changes to the protocol.

In Step 2, we anticipated a potential need for further searching for empirical data, but our project team agreed that the processes described above had identified sufficient material to meet our needs. During the analysis, we conducted a series of focused searches to identify material related to one substantive theory – 'street-level bureaucracy', which has been used to support our explanations of variation in delivery of the NHS Health Check.(21) The details of these searches are also reproduced in **Supplementary File 2**.

For Step 3, our original criteria were updated in light of the review project's chosen focus on what happens after measurements and risk assessments are completed. We sought in particular documents that contained data relating to later steps in the NHS Health Check pathway, including the provision of advice, signposting, referrals or prescriptions, and outcomes associated with these.

In Step 4, CD and EG coded a 10% set of the included documents independently and GW provided an additional check to ensure that all relevant data was captured and coding was applied consistently.

Results

Documents included in the review

In total, 124 documents contributed data to the review, including: 59 published research papers or reports; 20 documents reporting local evaluations; 34 conference materials (presentations, abstracts and posters) and 11 others (including policy reports, guidance, news articles and theses). Of these, 21 contributed data relating to the NHS Health Check programme as a whole, and the rest focused on specific localities. The processes of identifying, screening and selecting these are summarised below in Figure 1 (an adapted version of the Preferred Reporting Items for Systematic Reviews and Meta-Analyses (PRISMA) flow diagram).(19)

Figure 1: Summary of searching and selection processes

[Figure 1 here]

For peer review only

The main characteristics of the included documents are provided in **Supplementary File 3.** This table also indicates the extent to which each document contributed to the analysis, by showing the contribution of extracted data from each to specific CMOCs.

Review findings

Our findings describe what our included data told us about what happens at the end of an NHS Health Check, after the measurements and risk assessments are completed. Our explanations describe the reasoning and responses of three important groups involved in the programme: LA commissioners, NHS Health Check providers, and attendees. A narrative summary of our findings follows; it is based on our realist analysis that developed 86 CMOCs underpinned by the data included in the review. Overall, our findings illustrate how the delivery and outcomes of local NHS Health Check programmes and individual Heath Check encounters are shaped by a wide range of influences. To provide transparency, a detailed summary of the CMOCs developed and the data underpinning each is available in **Supplementary File 4**. We have included references to illustrative CMOCs to support the narrative that follows.

Understanding and engagement with the NHS Health Check

Our review findings point to variation in understandings of the primary purpose of the NHS Health Check. This variation is a critical factor that determines how the programme is commissioned, delivered and received. The programme was designed with two aims in mind: early identification of cases of diagnoseable illness or individuals with 'high' risk, and early intervention to support individuals to manage and reduce their CVD risk, via prescribing or by prompting or enabling behaviour change. Further to these, the programme is also cast as an opportunity to address disparities in CVD risk factors across England, although it maintains a focus on individual behaviours. Whilst there is a focus in the literature on inequality in relation to invitation and coverage of the check,(22, 23) our review uncovered very little data and almost no focus on the relationship between inequalities and the delivery of advice and referral. This gap is also reflected in the evidence collated to support the recently published PHE/OHID-led review.(11)

For peer review only - http://bmjopen.bmj.com/site/about/guidelines.xhtml

Our analyses of the data suggest that commissioners and providers may have a tendency to prioritise some aspects of the programme over others, for example, increasing volume of delivery and delivery of risk assessments over risk management. This prioritisation is then reflected in local commissioning decisions and delivery models. When the programme is understood primarily as an opportunity to screen for cases of existing CVD or near-CVD (very high risk factors), responsibility for programme delivery and outcomes is likely to rest with primary care, and specifically in general practice, where most NHS Health Checks are provided, and where clinical and especially pharmacological follow-up should happen (see, e.g. *CMOCs C9-C10* in **Supplementary File 4**). This perspective leads to an emphasis on high-volume throughput, increasing coverage of the programme, and a focus on efficient delivery of the minimum requirements for each check, collecting mandatory data and communicating risk scores. At the other end of the spectrum, where commissioners and providers are more oriented towards using the programme as a means of supporting behaviour change in order to reduce or manage CVD risk, delivery reflects this, with greater emphasis on advice or coaching, and facilitating onward referrals to 'lifestyle services', such as smoking cessation or weight management programmes (*CMOCs C1-C8*).

Our data demonstrates how such differences in local priorities for the NHS Health Check can drive variation in who delivers the check and the training they receive, the settings in which checks are delivered and the time allocated to each check (*CMOCs C1-C8, P14, P26*), all of which go on to affect how attendees experience the programme (*CMOCs A12-A17, A30-A37)*. In addition, these differences can affect the availability, accessibility and connectedness of referral pathways to local 'lifestyle services' that might be offered to attendees post-check, and important differences in the information that LAs collect, how they monitor and evaluate programme delivery, as well as funding models that can incentivise certain aspects of delivery (*CMOCs C2-C3, C5-C8)*. Commissioners and providers each influence how the programme is shaped and implemented at local levels, exercising discretion in determining the remit and focus of the programme.

This double layer of discretion in how the NHS Health Check is ultimately enacted in different local areas means that the relationship between LA commissioners and providers is an important factor in

For peer review only - http://bmjopen.bmj.com/site/about/guidelines.xhtml

determining what local delivery looks like. Commissioners determine service specifications and funding models and the scope of monitoring of programme delivery, but (unless they are themselves providers, as is the case for some provision in a few LAs) their day-to-day influence on actual delivery is limited (*CMOC C4*). This is a particular concern as prevailing scepticism (in particular amongst GPs) about the programme's effectiveness and concerns about the potential for overdiagnosis has led to disengagement amongst some providers. Such concerns, however, must be balanced with their reliance on income generated by the programme (*CMOCs P1-P5*). Differences in levels of engagement with the programme can reflect different understandings about its primary purpose and concerns about its ability to meet its aims, but they can also be fuelled by more practical concerns, including competing priorities and workload pressures, and the need to deliver NHS Health Checks and appropriate follow-up with limited resources (see below and *CMOCs P9-P13, P15-P18*).

Local delivery models send important signals to attendees that inform how they understand and engage with the programme in turn. Checks delivered in general practice with a focus on completing mandatory measurements and risk assessments carry the implication that the check should be understood as a screening test or a health 'MOT', that may or may not result in the need for clinical intervention (*CMOCs A1*, *A12*). Delivery in community settings, by providers trained specifically to deliver coaching or behavioural support, or otherwise with a greater focus on delivering advice or referrals, sends a different message *(CMOC A14).* In some areas, NHS Health Checks have been delivered within a wider integrated lifestyle service, facilitating connections with lifestyle services, and potentially additional services such as link workers or social prescribers. These models may enable providers to offer more holistic support that better meets the needs of individual attendees; much data from the attendee perspective suggests that this may help to generate better engagement with the programme and its aims.

Our data also suggest that attendees' prior expectations of the check also inform their response, and so it is important that national and local advertising about the programme are clear about its remit and purpose. Providers can transmit 'soft' signals to attendees about the programme's purpose and value, and attendees will pick up on these (*CMOCs A17-24)*. They have the potential to convey urgency, and

For peer review only - http://bmjopen.bmj.com/site/about/guidelines.xhtml

even induce fear and anxiety in attendees, but they may also (intentionally or otherwise) imply that the check is a 'box ticking' exercise.

Finally, it is important to acknowledge that attendees' themselves can exercise discretion in their response to their check, regardless of how providers approach its delivery. The programme's focus on individual behaviour creates high expectations of individual action to address any 'lifestyle risk factors' identified. Inevitably, attendees may face considerable challenges in implementing behaviour changes within their own lives, where they too may have limited resources (*CMOCs A38-A34*). Some literature has drawn attention to the risks inherent in this individual focus, including the potential for positive health impacts to be realised only by those with the capacity to make significant lifestyle changes, and so unfairly distributing any benefits amongst attendees.(24) Such a focus risks increasing rather than decreasing health inequalities.

Practical constraints limiting the NHS Health Check

Commissioners and providers face substantial practical constraints that limit and inform their exercise of discretion in programme delivery. As noted above, the decisions of LA commissioners that are enacted in funding models, service specifications and monitoring regimes set important boundaries for providers, potentially incentivising different delivery methods and priorities. Commissioners' decisions must take into account restricted funding for public health initiatives (*CMOCs C12-C14*). Central government grants to LAs have fallen substantially since 2010. Since public health responsibilities were transferred to LAs in 2013, the public health grant has decreased by 13% in real terms.(30) Spending on the NHS Health Check programme fell by 21% between 2015/16 and 2019/20, and spending on 'lifestyle services' that could potentially support attendees post-check has also fallen: expenditure on smoking cessation initiatives fell by one third over the same period, adult alcohol and drug services by 17% and weight management services by 5%.(31) Reductions in funding of this scale necessarily limit what commissioners can purchase (*CMOCs C14, A35-A37)*. Our findings raise the question of whether public health funding overall is adequate to support the programme's more ambitious aim of preventing CVD and reducing CVD inequalities by reducing or managing behavioural risk factors. In particular, it is apparent that the delivery of personalised,

For peer review only - http://bmjopen.bmj.com/site/about/guidelines.xhtml

individual advice and discussion during checks, and the offer of further support afterwards, is a more intensive proposition than a programme focused on case finding (*CMOCs P9-P13)*.

Providers face other constraints. There is a need to ensure that staff involved in delivering checks are competent to do so, but some data suggest that not all providers feel confident in delivering support for behaviour change (*CMOCs P20-P23)*. Training, too, must reflect the programme's aims both to identify and to address CVD risk factors (*CMOC P26*). Wherever checks are delivered in general practice or community pharmacy settings, providers face competing priorities and demands on their time. Workload and time pressure may push providers towards 'leaner' delivery, focused on mandatory tests and capturing required measurements, leaving little time for discussion, advice and offers of referral (*CMOCs P9-P14)*.

There is a crucial relationship between the practical constraints that commissioners and providers face and their understanding and engagement with the programme. It is likely that both groups of actors adapt their understanding of the programme based on what they know about the limited resources available to deliver and support it. Downward pressure on funding inevitably incentivises leaner delivery models and, as our findings make clear, these models tend to favour the 'early identification' or case finding purpose of the programme. Provider scepticism is also likely to be compounded by the sense that both the programme itself, and subsequent services on which it might depend are underfunded and inaccessible (*CMOCs P1-P3)*.

Finally, our data make clear the impact of the varied delivery models on attendees' experience of the programme and on what they are offered (or not) post-check. It is unclear which delivery models allow or incentivise providers to deliver the meaningful, personalised and ongoing advice and support attendees might need. Existing ethnographic research has demonstrated that the time-constrained and highly structured nature of the NHS Health Check assessments impede meaningful discussion that prioritises understanding individuals' circumstances.(25, 26) Limited access to 'lifestyle services' means these are inaccessible to many.

For peer review only - http://bmjopen.bmj.com/site/about/guidelines.xhtml

Variation in NHS Health Check delivery models: street-level bureaucracy

Our review findings demonstrate wide variation in how the NHS Health Check programme is implemented locally, with a specific focus in variation in the delivery of what happens after measurements and risk assessments have been completed. Inconsistencies in the recording of these activities (especially the delivery and uptake of advice and referrals post-check) prevent the development of a comprehensive picture of this variation across England. However, our review findings provide a starting point to improve understanding of what influences delivery in these areas, highlighting the discretion available to LA commissioners and individual NHS Health Check providers in making decisions about how the programme is delivered on the ground. In addition, they highlight how differences in delivery models affect how the checks are experienced by attendees, and what colours their responses to the information they receive and any offers of further intervention.

Our understanding of the processes at work in driving variation draws on Lipsky's concept of 'street-level bureaucracy', borrowed from the international relations literature.(21, 27) Previous research on the NHS Health Check,(28) and the implementation of other health policies in the UK(29, 30) have used the same theory to add explanatory value. Lipsky's framework emphasises the discretion of those charged with implementing national policies or programmes, as well as their responses to working with limited resources. For the NHS Health Check programme, it is clear that while commissioners and providers are working within the broad constraints of a legal framework(3, 4) and guidance issued by PHE(1) (now OHID), their decisions and everyday practice in delivering checks effectively determine the remit and purpose of the programme at local levels. As Lipsky describes it: "*the decisions of street-level bureaucrats, the routines they establish, and the devices they invent to cope with uncertainties and work pressures, effectively become the public policies they carry out".(27)*

The extent to which discretion can be exercised in relation to the NHS Health Check may be greatest at the end of the programme pathway. Processes relating to earlier steps – the identification of the eligible population, invitation and the actual measurements and risk assessments to be administered – are restricted by the programme's legal framework and clear guidance, leaving little room for local

For peer review only - http://bmjopen.bmj.com/site/about/guidelines.xhtml

interpretation or adaptation. The later steps – especially the delivery of advice and referrals – are less prescribed and more dependent on local delivery models and other local services. Activity in these areas is less well recorded and monitored, leaving LA commissioners and providers with more discretion to determine what day-to-day local delivery of checks will look like.

Although Lipsky's original framework focuses on those directly engaged in the delivery of policies and their interaction with the recipients or subjects of those policies (in our case, the interaction of providers with attendees) our analysis also highlights the discretion of LA commissioners who make decisions about local programme specifications and support. Whilst they may not directly interact with the public, commissioners must also interpret the requirements of the programme and exercise their own discretion to ensure it meets local needs and is delivered within local constraints. The approach of the LA directly affects commissioned providers, and this double layer of discretion forms the local contexts in which NHS Health Checks are delivered across England.

Taken together, the CMOCs developed in this review point to the influence of both commissioners' and providers' understanding and engagement with the programme, as well as the effects of practical constraints that drive decision making in relation to programme delivery. These two aspects mirror Lipsky's concepts of *discretion* in the enactment of policies and programmes, and the effects of their responses to *limited resources*. Attendees' experience of, and response to the programme is affected both by the outcomes of commissioner and provider decisions, but also by other external factors related to their individual circumstances, which may be difficult to align with what the NHS Health Check expects of them.

Discussion

Summary of findings

The success of the NHS Health Check programme as an intervention that aims to support individuals to manage and reduce their CVD risk rests on what happens to attendees when the measurements and risk assessments have been completed. While case-finding is an important indicator of success, the delivery of advice, offers of signposting, referrals or other support, and attendees' responses to these,

For peer review only - http://bmjopen.bmj.com/site/about/guidelines.xhtml

is equally crucial for the programme to prevent ill-health and reduce inequalities in the longer term. Our focus on this area reflected the importance of this step – confirmed by our two stakeholder groups – but also the relative lack of attention that it has received in the existing research literature. Our review therefore sought to examine the final steps in the NHS Health Check programme pathway, to understand the factors that influence the delivery of these parts of the check and what follows, and how they are received by those who receive checks.

The NHS Health Check as a whole has been the subject of several previous evidence synthesis projects, which have considered many aspects of the programme, and have similarly identified wide variation in delivery models and outcomes. Our review is the first to use a realist approach, and the first to focus specifically on the steps that follow the measurements and risk assessments made during each check. Relevant findings from existing reviews correspond with our own. The two PHE-commissioned rapid reviews identified some limited evidence of geographical variation in referrals, and captured qualitative data describing providers' doubts and scepticism about the programme, and perceived training needs. These reviews also included qualitative studies focused on attendees, which described attendees' perspectives on the quality of information delivered during checks and the important constraints imposed by "environmental", "resource" and "time" factors that limit attendees' capacity to make and sustain behaviour changes.(12, 13) Another synthesis coded evidence relating to the behaviours of commissioners, providers and attendees in relation to the programme, and reported similar findings related to providers' skills, attitudes and beliefs, as well as the resources available to deliver checks and the need to take account attendees' wider "social contexts" when delivering advice.(31)

Our review extends the work undertaken in these reviews, by explaining how and why variation in how the programme is delivered comes about, with a particular focus on what happens after the measurements and risk assessments are completed. Our findings point to the significance of the exercise of discretion by LA commissioners and NHS Health Check providers in the delivery of checks. Commissioners' decisions in relation to programme implementation, funding and monitoring, and providers' actions to deliver checks on the ground are influenced by multiple factors. These

For peer review only - http://bmjopen.bmj.com/site/about/guidelines.xhtml

include their understanding of the primary purpose of the programme and engagement with its aims, but these attitudes towards the programme – and commissioners' and providers' actions in organising and providing checks – are constrained by important practical factors.

Prevailing conditions, including limited funding for public health programmes and services overall, current funding and monitoring arrangements for the NHS Health Check programme, and competing priorities for many providers tend to push towards a delivery model that prioritises the programme's aim of early detection – case finding. The prioritisation of this element of the programme may be to the detriment of work that could help to prompt or support behaviour change. These constraints raise ethical questions about the programme: if it is oriented towards early identification of risk, but cannot genuinely support early intervention to help manage and reduce risks (bar prescribing), where does this leave attendees? There is a need to consider the relationship between the NHS Health Check programme and the wider landscape of local and national services that can be offered to attendees.

In some local areas, there is evidence that commissioners and providers work against this tide, using their local NHS Health Checks as a means of supporting attendees to make behaviour changes that could help to reduce their CVD risk. Our final programme theory diagram summarises these findings and is presented below in **Figure 2**.

Figure 2: Final programme theory

[Figure 2 here]

Covid-19 and the NHS Health Check

We undertook this review during the Covid-19 pandemic, which had a major impact on delivery of the NHS Health Check. In April 2020, the programme was effectively paused.(32, 33) To support resumption of delivery, PHE issued 'restart guidance' in April 2021, encouraging LAs to consider restarting the programme, dependent on local safety assessments and the need to prioritise the vaccination programme.(34) This document acknowledged the pressures faced by general practice in particular, and urged LA commissioners to consider 'alternative' providers. Other communications from PHE and the Department of Health and Social care during the pandemic highlighted the

For peer review only - http://bmjopen.bmj.com/site/about/guidelines.xhtml

For peer review only

potential benefits of the NHS Health Check in relation to identifying risk factors for severe Covid-19 outcomes, as many CVD risk factors are also associated with higher risks of hospitalisation and death from Covid-19.(35, 36, 37)

In light of our findings, we note that the effects of the pandemic have the potential to exacerbate some existing contexts that may adversely affect delivery of the programme's aims. In particular, pressure to 'catch up' and concerns about delayed or missed diagnoses could lead to a more intense focus on early detection and case finding.(38) Workload pressures affecting primary care providers may also increase disengagement from the NHS Health Check, and LA commissioners must consider these effects as they restart the programme in each local area.

Implications for policy and practice

Our review findings raise a number of important questions for policy makers, commissioners and providers to consider in relation to the NHS Health Check. Our main recommendation is that all three of these groups (and future researchers) should increase their focus on evaluating and improving the delivery of the later steps in the programme pathway, i.e. on what happens to attendees after the measurements and risk assessments have been completed. Our more detailed recommendations are summarised below in **Table 2**. They are organised around four important and inter-connected principles, developed in consultation with our PPI and professional stakeholder groups: clarifying the purpose of the programme; increasing engagement with the programme; focusing the Health Check on attendee needs; and improving links between the NHS Health Check and other programmes and services.

Table 2: Summary of recommendations based on review findings

Our recommendation	Rationale
Ensure all national and local guidance and programme documentation reflects the importance of the programme's aims in relation to both early identification of CVD risk, and early intervention to manage/reduce that risk.	Different interpretations of the primary purpose of the programme drive variation in commissioning and delivery, and subsequently in attendee experience.

For peer review only - http://bmjopen.bmj.com/site/about/guidelines.xhtml

Assess overall programme funding and local funding arrangements in relation to delivery of (all aspects of) the programme's aims.	Commissioner, provider and attendee scepticism about the programme can undermine its delivery; inadequate resources for the programme and support services for attendees post-check can reduce engagement. Funding arrangements may incentivise leaner or more intensive checks.
Review national and local data monitoring and evaluation of the programme, to ensure that data relating to all key aspects of the programme (and especially the final steps in the NHS Health Check pathway) are captured.	Data collection is currently focused on invitation, uptake and coverage, incentivising high-volume, less intensive delivery that focuses on mandatory data points. Data on advice and referral (including data relating to potential disparities in provision) are needed to evaluate the delivery of these steps.
Review training for providers to assess its ability to support all aspects of programme delivery, including the final steps in the NHS Health Check pathway.	Providers and attendees have identified deficiencies in skills and knowledge, especially in relation to the delivery of advice, support and referrals. All providers should receive adequate training to ensure that they can deliver these aspects of a check or signpost/refer attendees to an appropriate source of support.
Review connections between the programme and national and local services that could offer further support for attendees, and options for longer term follow-up after measurements and risk assessments are completed.	The success of the programme rests in part on its connections with other services that could offer further support for attendees. Strengthening these connections could allow the NHS Health Check to act as a gateway to these services (as well as to primary care/general practice when necessary)
Continue to produce evidence for programme effectiveness and address the relative scarcity of evidence focused on later steps in the NHS Health Check pathway.	Commissioner, provider and attendee scepticism about the programme can undermine its delivery; evidence from research and evaluation should go beyond a focus on increasing coverage of the programme and inform practical recommendations for good practice in delivery.

Strengths and limitations of this review

Our review has developed novel interpretations of existing secondary data relating to what happens at the end of the NHS Health Check pathway. Our close examination of what happens after the measurements and risk assessments have been completed during checks helps to address the relative lack of research on this particular aspect of programme delivery. The review project was strengthened by close working with our diverse PPI and professional stakeholder groups, who helped us to focus the review, provided detailed feedback on emerging findings and shaped our interpretation of the data and the development of recommendations. We included a diverse range of material in the review, and

For peer review only

in particular, drew on the learning captured in a wide range of grey literature including local evaluations and conference materials.

As with any review, our findings are limited by the availability and quality of the literature. The material included in the review covers a wide date range and some older material may be less applicable to the present day. However, we considered each piece of data carefully before inclusion and aimed to select material that spoke to still-relevant contexts, mechanisms and outcomes. Our stakeholders also helped us to confirm the contemporary relevance of our findings. Our CMOCs vary in terms of the volume and rigour of the data that underpin them. We have provided a full and transparent account of that data in **Supplementary Files 3 and 4,** so that the strength of each is made clear to readers.

Conclusions

Our review has revealed wide variation in the delivery of advice and support, and onward signposting and referral for attendees and identified explanations for this variation. We have identified a wide range of influences that affect how LA commissioners and NHS Health Check providers develop and deliver the programme at local levels across England, which affect how attendees experience and respond to their check. Our analysis explains how differences in understanding of the primary purpose of the programme influences commissioning and implementation, and how practical constraints limit what can be delivered within the programme's remit and existing resources. Based on our findings, we developed a set of recommendations for policy makers, commissioners and providers to inform future programme development. Our recommendations centre on the need for greater emphasis to be placed on the final steps in the NHS Health Check programme pathway, including in national and local guidance and programme documentation, funding models, provider training, monitoring and evaluation of the programme, and in considering how the NHS Health Check can be better linked with wider services and programmes.

List of abbreviations

BMI: Body mass index

CINAHL: Cumulative Index to Nursing and Allied Health Literature

For peer review only - http://bmjopen.bmj.com/site/about/guidelines.xhtml

CMOC: Context-mechanism-outcome configuration (see glossary for a definition)

CVD: Cardiovascular Disease

DHSC: Department of Health and Social Care

GP: General Practitioner

HMIC: Health Management Information Consortium

IPT: Initial Programme Theory

LA: Local authority

MOT: 'Ministry of Transport', test to determine if cars are roadworthy

NHS: National Health Service

NICE: National Institute for Health and Care Excellence

OHID: Office for Health Improvement and Disparities

PHE: Public Health England

PPI: Patient and Public Involvement

PRISMA: Preferred Reporting Items for Systematic Reviews and Meta-Analyses

Required statements

Acknowledgements

We would like to express our thanks for the help, feedback and advice provided by all of our wonderful stakeholders and patient and public involvement contributors, and for the continued engagement of PHE and OHID and their help with the recruitment of stakeholders and opportunities for dissemination of our findings and outputs.

Ethics approval

Not applicable for this review

For peer review only

For peer review only - http://bmjopen.bmj.com/site/about/guidelines.xhtml

Author contributions

JK and GW conceived the research project and all of the authors contributed to its design. GW provided methodological support. CD devised and carried out the literature searches and led the analysis and interpretation of the data. The other authors contributed to the analysis. VH led the PPI involvement. CD drafted the manuscript and the other authors provided feedback. All of the authors read and approved the final manuscript.

Funding statement

This project was funded by the National Institute for Health Research (NIHR) Health Services and Delivery Research programme (NIHR129209). The views expressed are those of the authors and not necessarily those of the NIHR or the Department of Health and Social Care.

Competing interests

CD and GW are both members of the Royal College of General Practitioners (UK) Overdiagnosis and Overtreatment Group. GW is an NHS General Practitioner and deputy chair of the National Institute for Health Research Health Technology Assessment Prioritisation Committee: Integrated Community Health and Social Care Panel (A) and member of the Methods Group (A).

Data sharing statement

The datasets generated and/or analysed during the current study are not publicly available due to copyright and licensing restrictions but are available from the corresponding author on reasonable request.

References

1. Public Health England. NHS Health Check Best practice guidance For commissioners and providers. 2020. Available from: https://www.healthcheck.nhs.uk/commissioners-and-providers/national-guidance/. Accessed 31st October 2019.
2. HM Government. Levelling Up. Levelling Up the United Kingdom. Her Majesty's Stationery Office; 2021 2 February 2022. Available from: https://assets.publishing.service.gov.uk/government/uploads/system/uploads/attachment_data/file/1052708/Levelling_up_the_UK_white_paper.pdf. Accessed 9th February 2022.
3. Great Britain. The Local Authorities (Public Health Functions and Entry to Premises by Local Healthwatch Representatives) Regulations. Part 2 Public Health Functions: Conduct of Health Checks. United Kingdom; 2013. Available from:

For peer review only - http://bmjopen.bmj.com/site/about/guidelines.xhtml

https://www.legislation.gov.uk/uksi/2013/351/regulation/4/made/data.pdf. Accessed 31st October 2020.
4. Great Britain. The Local Authorities (Public Health Functions and Entry to Premises by Local Healthwatch Representatives) Regulations. Part 2: Public Health Functions: Health check assessment. United Kingdom; 2013. Available from: https://www.legislation.gov.uk/uksi/2013/351/regulation/4/made/data.pdf. Accessed 31st October 2020.
5. Office for Health Improvement and Disparities. NHS Health Check Programme review. 2021. Available from: https://www.gov.uk/government/publications/nhs-health-check-programme-review. Accessed 9th December 2021.
6. Abdalrahman B, Soljak M. NHS health checks: an update on the debate and program implementation in England. J Ambul Care Manage. 2015;38(1):5-9.
7. McCartney M. Where's the evidence for NHS health checks? BMJ. 2013;347:f5834.
8. Capewell S, McCartney M, Holland W. Invited debate Response to Waterall et al. J Public Health (Oxf). 2015;37(2):185-6.
9. Ford J, Ekeke N, Lahiri A, Kelly MP. Making the case for prevention. Cambridge, UK: A commissioned report by Cambridge Public Health, University of Cambridge, for the Health Foundation,; 2021. Available from: https://www.phpc.cam.ac.uk/pcu/research/research-groups/crmh/research/crmh-health-inequalities/making-the-case-for-prevention/. Accessed 9th February 2022.
10. Public Health England. Review of NHS Health Checks: terms of reference. 2020. Available from: https://www.gov.uk/government/publications/nhs-health-check-programme-review/review-of-nhs-health-checks-terms-of-reference#scope. Accessed
11. Office for Health Improvement and Disparities. NHS Health Check Programme review. Annex B: a summary of analyses and evidence on the current NHS Health Check programme. 2021. Available from: https://www.gov.uk/government/publications/nhs-health-check-programme-review/annex-b-a-summary-of-analyses-and-evidence-on-the-current-nhs-health-check-programme. Accessed 9th December 2021.
12. Usher-Smith JA, Mant J, Martin A, Harte E, MacLure C, Meads C, et al. NHS Health Check Programme rapid evidence synthesis. Cambridge, UK: The Primary Care Unit, University of Cambridge, RAND Europe, Public Health England; 2017. Accessed 31st October 2020.
13. Tanner L, Kenny R, Still M, Pearson F, Bhardwaj-Gosling R. NHS Health Check Programme Rapid Review Update. University of Sunderland, Newcastle University, Public Health England; 2020. Available from: https://www.healthcheck.nhs.uk/commissioners-and-providers/evidence/. Accessed 31st October 2020.
14. Patel R, Barnard S, Thompson K, Lagord C, Clegg E, Worrall R, et al. Evaluation of the uptake and delivery of the NHS Health Check programme in England, using primary care data from 9.5 million people: a cross-sectional study. BMJ Open. 2020;10(11):e042963.
15. Duddy C, Wong G, Gadsby EW, Krska J, Hibberd V. NHS Health Check programme: a protocol for a realist review. BMJ Open. 2021;11(4):e048937.
16. The RAMESES Project. Quality Standards for Realist Synthesis (for researchers and peer-reviewers). 2014. Available from: https://www.ramesesproject.org/media/RS_qual_standards_researchers.pdf. Accessed 31st October 2020.
17. Wong G, Greenhalgh T, Westhorp G, Buckingham J, Pawson R. RAMESES publication standards: realist syntheses. BMC Med. 2013;11(1):21.
18. Pawson R. Realist synthesis: new protocols for systematic review. 2006. In: Evidence-based policy: a realist perspective [Internet]. [73-104].
19. Page MJ, McKenzie JE, Bossuyt PM, Boutron I, Hoffmann TC, Mulrow CD, et al. The PRISMA 2020 statement: an updated guideline for reporting systematic reviews. BMJ. 2021;372:n71.
20. Wong G. Data gathering in realist reviews: Looking for needles in haystacks. 2018. In: Doing Realist Research [Internet]. London: SAGE Publications Ltd; [131-46].
21. Lipsky M. Toward a theory of street-level bureaucracy. Madison, WI: Institute for Research on Poverty (IRP), University of Wisconsin,; 1969. Available from: http://www.irp.wisc.edu/publications/dps/pdfs/dp4869.pdf. Accessed 1st November 2021.

For peer review only - http://bmjopen.bmj.com/site/about/guidelines.xhtml

22. Bunten A, Porter L, Gold N, Bogle V. A systematic review of factors influencing NHS health check uptake: invitation methods, patient characteristics, and the impact of interventions. BMC public health. 2020;20(1):93.
23. Martin A, Saunders CL, Harte E, Griffin SJ, MacLure C, Mant J, et al. Delivery and impact of the NHS Health Check in the first 8 years: a systematic review. Br J Gen Pract. 2018;68(672):e449-e59.
24. Stol YH, Schermer MHN, Asscher ECA. Omnipresent Health Checks May Result in Over-responsibilization. Public Health Ethics. 2017;10(1):35-48.
25. Cupit C. An ethnographic study of cardiovascular disease prevention: the social organisation of measures, knowledge, interventions and tensions in English general practice: University of Leicester; 2018.
26. Strutt E. Patient-centred care: Patients' experiences of and responses to the National Health Service (NHS) Health Check programme in general practice. Durham: Durham University; 2011.
27. Lipsky M. Street-Level Bureaucracy: Dilemmas of the Individual in Public Service. 30th Anniversary Edition ed. New York, New York: Russell Sage Foundation; 2010.
28. Gale N, Dowswell G, Greenfield S, Marshall T. Street-level diplomacy? Communicative and adaptive work at the front line of implementing public health policies in primary care. Soc Sci Med. 2017;177:9-18.
29. Bergen A, While A. 'Implementation deficit' and 'street-level bureaucracy': policy, practice and change in the development of community nursing issues. Health Soc Care Community. 2005;13(1):1-10.
30. Cooper MJF, Sornalingam S, O'Donnell C. Street-level bureaucracy: an underused theoretical model for general practice? Br J Gen Pract. 2015;65(636):376.
31. Atkins L, Stefanidou C, Chadborn T, Thompson K, Michie S, Lorencatto F. Influences on NHS Health Check behaviours: a systematic review. BMC Public Health. 2020;20(1):1359.
32. NHS England and Improvement. COVID-19 Prioritisation within Community Health Services [Letter]. 2020. Accessed 20th March 2020.
33. Thompson K. NHS Health Check e-Bulletin - April 2020 Operational Update. 2020. Contract No.: 30th April 2020. Available from: https://www.nhshealthcheck.nhs.uk/nhs-health-check-e-bulletin-april-2020/front-page/nhs-health-check-e-bulletin-april-2020/print. Accessed
34. Public Health England. NHS Health Check: Restart Preparation. 2021. Contract No.: Version 11: 09 April 2021. Available from: https://www.healthcheck.nhs.uk/commissioners-and-providers/national-guidance/. Accessed 9th April 2021.
35. Public Health England. NHS Health Check e-Bulletin - August 2020. 2020. Contract No.: 5th January 2022. Available from: https://www.nhshealthcheck.nhs.uk/nhs-health-check-e-bulletin-august-2020/front-page/nhs-health-check-e-bulletin-august-2020. Accessed
36. Public Health England. Summary: An Umbrella Review on Cardiovascular Risk Factors, Cardiovascular Disease and COVID-19. 2021. Available from: https://www.healthcheck.nhs.uk/latest-news/an-umbrella-review-on-cardiovascular-risk-factors-cardiovascular-disease-and-covid-19/. Accessed 31st May 2021.
37. Cabinet Office. Our plan to rebuild: The UK Government's COVID-19 recovery strategy. 2020. Contract No.: 24th February 2022. Available from: https://www.gov.uk/government/publications/our-plan-to-rebuild-the-uk-governments-covid-19-recovery-strategy/our-plan-to-rebuild-the-uk-governments-covid-19-recovery-strategy. Accessed
38. Oxford Academic Health Science Network. CVD prevention during and after the COVID-19 pandemic. Getting It Right First Time, Association of Directors of Public Health; 2020. Available from: https://www.oxfordahsn.org/wp-content/uploads/2020/12/CVD-prevention-during-the-COVID-19-pandemic-guidance-for-system-level-change-December-2020.pdf. Accessed 31st January 2021.

For peer review only - http://bmjopen.bmj.com/site/about/guidelines.xhtml

For peer review only

For peer review only - http://bmjopen.bmj.com/site/about/guidelines.xhtml

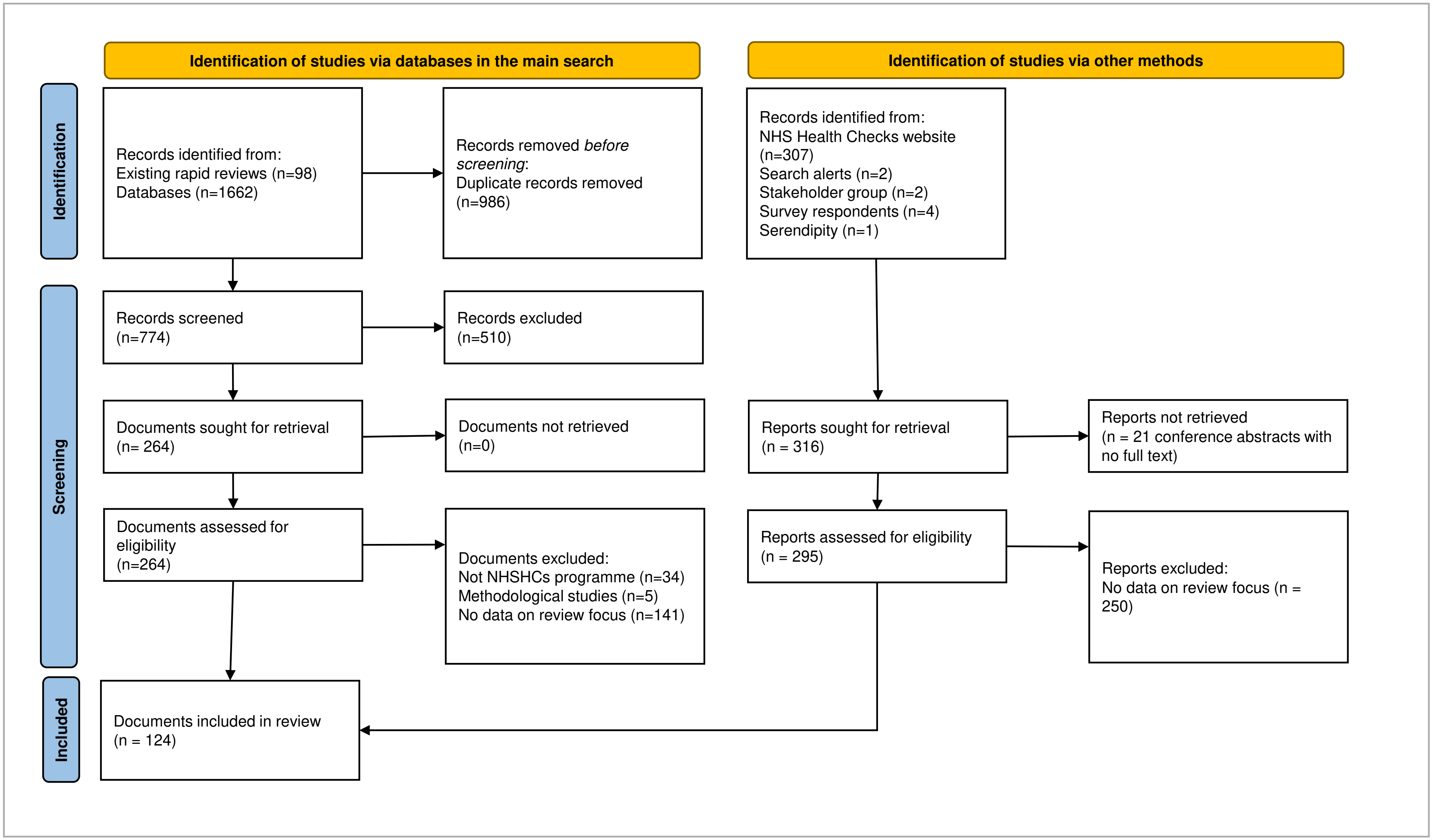
Identification of studies via databases in the main search
Identification of studies via other methods
Identification
Records identified from:
Existing rapid reviews (n=98)
Databases (n=1662)
Records removed *before screening*:
Duplicate records removed
(n=986)
Records identified from:
NHS Health Checks website
(n=307)
Search alerts (n=2)
Stakeholder group (n=2)
Survey respondents (n=4)
Serendipity (n=1)
Screening
Records screened
(n=774)
Records excluded
(n=510)
Documents sought for retrieval
(n= 264)
Documents not retrieved
(n=0)
Reports sought for retrieval
(n = 316)
Reports not retrieved
(n = 21 conference abstracts with no full text)
Documents assessed for eligibility
(n=264)
Documents excluded:
Not NHSHCs programme (n=34)
Methodological studies (n=5)
No data on review focus (n=141)
Reports assessed for eligibility
(n = 295)
Reports excluded:
No data on review focus (n = 250)
Included
Documents included in review
(n = 124)

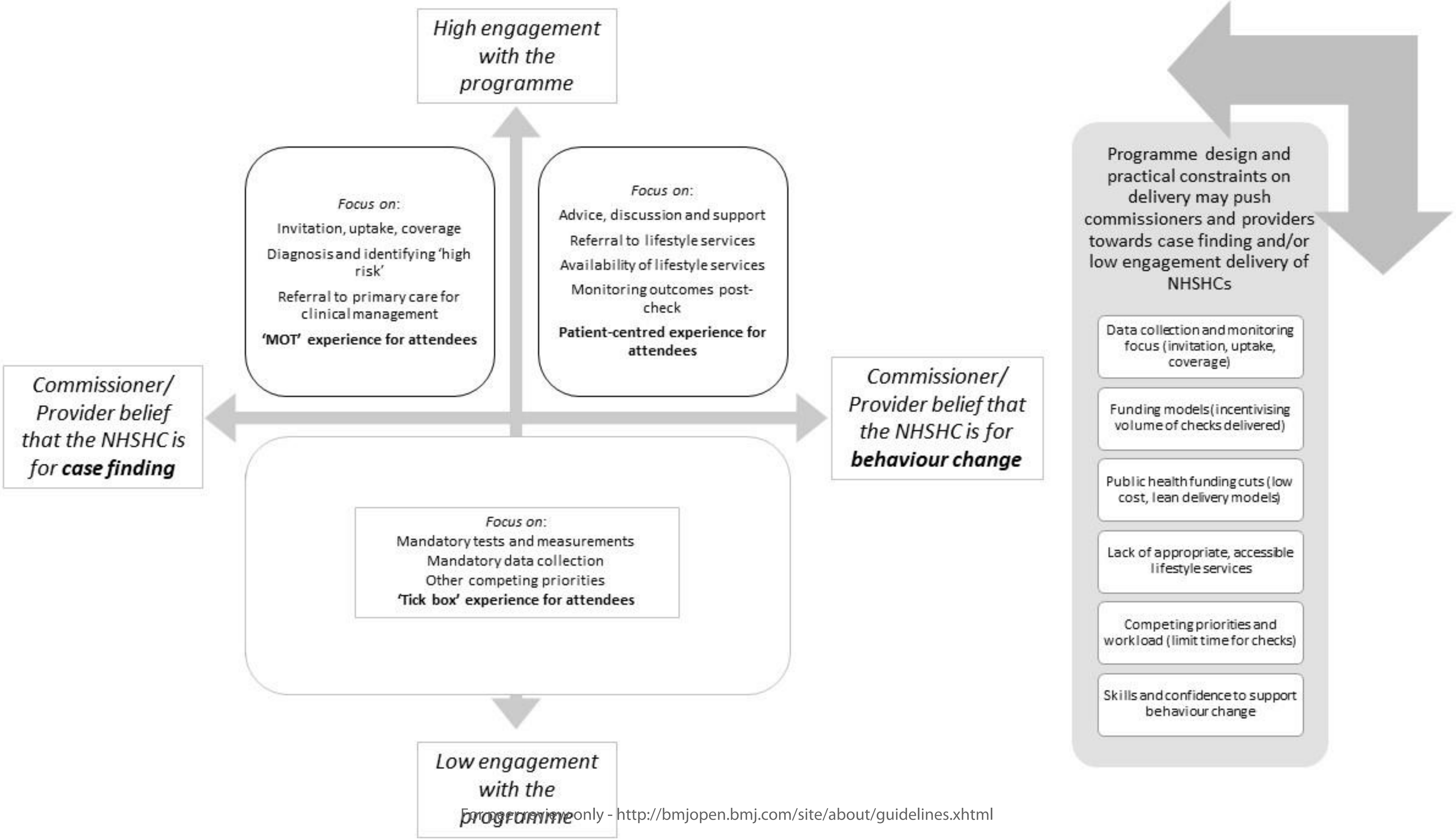
High engagement with the programme
Focus on:
Invitation, uptake, coverage
Diagnosis and identifying 'high risk'
Referral to primary care for clinical management
'MOT' experience for attendees
Focus on:
Advice, discussion and support
Referral to lifestyle services
Availability of lifestyle services
Monitoring outcomes post-check
Patient-centred experience for attendees
Commissioner/ Provider belief that the NHSHC is for case finding
Commissioner/ Provider belief that the NHSHC is for behaviour change
Focus on:
Mandatory tests and measurements
Mandatory data collection
Other competing priorities
'Tick box' experience for attendees
Low engagement with the programme
Programme design and practical constraints on delivery may push commissioners and providers towards case finding and/or low engagement delivery of NHSHCs
Data collection and monitoring focus (invitation, uptake, coverage)
Funding models (incentivising volume of checks delivered)
Public health funding cuts (low cost, lean delivery models)
Lack of appropriate, accessible lifestyle services
Competing priorities and workload (limit time for checks)
Skills and confidence to support behaviour change

Supplementary File 1

This file contains a glossary of realist terminology, a diagram summarising our initial programme theory and the focus of the realist review and the PRISMA checklist.

Glossary of realist terminology

Context: in realist research, the conditions or circumstances in which mechanisms that generate observed outcomes are 'triggered' or activated.

Context-mechanism-outcome configuration (CMOC): a heuristic used to present a realist causal explanation for an outcome, presented as a relationship between some particular context(s) and mechanism(s).

Demi-regularity: a semi-predictable pattern of outcomes that occur in the same context(s).

Mechanism: in realist research, the underlying context-sensitive causal force that generates an outcome, often conceptualised as the response of an individual actor to important context(s).

Programme theory: a set of theoretical explanations about how a programme, intervention or process is understood to work. Realist programme theories explain or the process by which outcomes of interest are thought to be generated, using causal explanations captured in the form of CMOCs.

Substantive theory: an established, formal theory drawn from any discipline that can be used to help understand the programme, intervention or process under examination.

For peer review only

Initial programme theory diagram

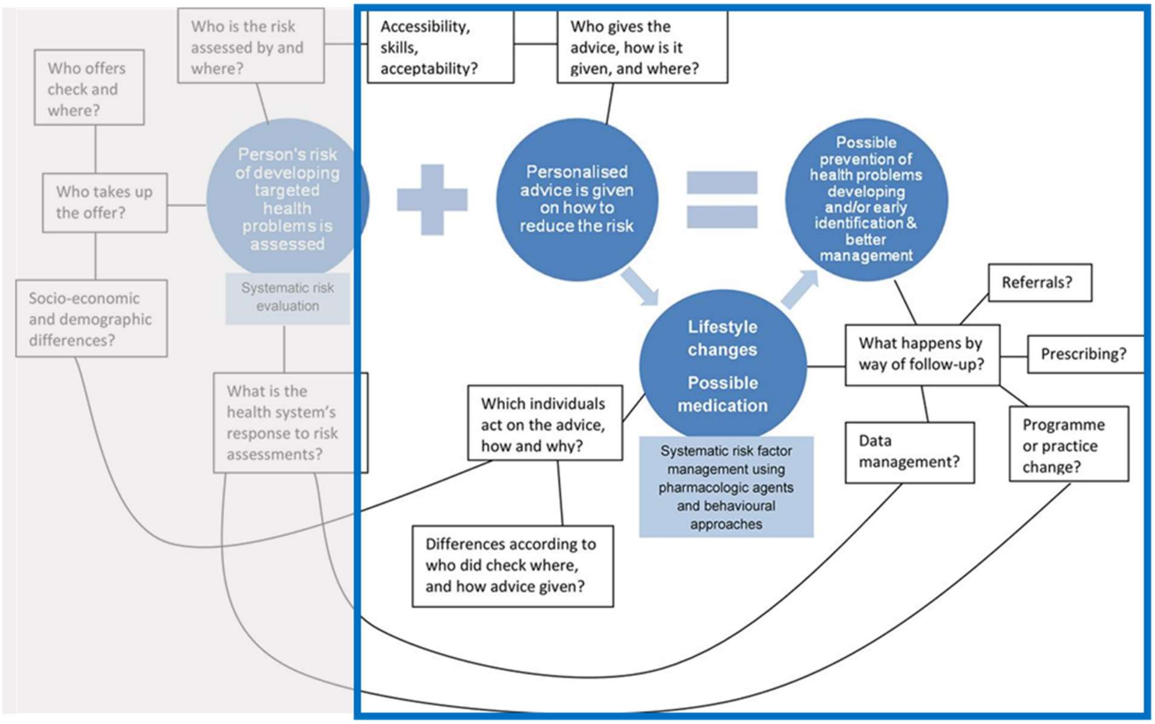

Initial programme theory for the realist review with project focus highlighted

review only

PRISMA checklist

Reporting checklist for systematic review (with or without a meta-analysis).

Based on the PRISMA guidelines.

Instructions to authors

Complete this checklist by entering the page numbers from your manuscript where readers will find each of the items listed below.

Your article may not currently address all the items on the checklist. Please modify your text to include the missing information. If you are certain that an item does not apply, please write "n/a" and provide a short explanation.

Upload your completed checklist as an extra file when you submit to a journal.

In your methods section, say that you used the PRISMAreporting guidelines, and cite them as:

Page MJ, McKenzie JE, Bossuyt PM, Boutron I, Hoffmann TC, Mulrow CD, Shamseer L, Tetzlaff JM, Akl EA, Brennan SE, Chou R, Glanville J, Grimshaw JM, Hróbjartsson A, Lalu MM, Li T, Loder EW, Mayo-Wilson E, McDonald S, McGuinness LA, Stewart LA, Thomas J, Tricco AC, Welch VA, Whiting P, Moher D. The PRISMA 2020 statement: An updated guideline for reporting systematic reviews

		Reporting Item	Page Number
**Title**			
Title	#1	Identify the report as a systematic review	1
**Abstract**			
Abstract	#2	Report an abstract addressing each item in the PRISMA 2020 for Abstracts checklist	2
**Introduction**			
Background/rationale	#3	Describe the rationale for the review in the context of existing knowledge	3-6
Objectives	#4	Provide an explicit statement of the objective(s) or question(s) the review addresses	6, protocol
**Methods**			

For peer review only

Eligibility criteria	#5	Specify the inclusion and exclusion criteria for the review and how studies were grouped for the syntheses	8-9, protocol
Information sources	#6	Specify all databases, registers, websites, organisations, reference lists, and other sources searched or consulted to identify studies. Specify the date when each source was last searched or consulted	8, Supplementary File 2
Search strategy	#7	Present the full search strategies for all databases, registers, and websites, including any filters and limits used	Supplementary File 2
Selection process	#8	Specify the methods used to decide whether a study met the inclusion criteria of the review, including how many reviewers screened each record and each report retrieved, whether they worked independently, and, if applicable, details of automation tools used in the process	8, Table 1 and protocol
Data collection process	#9	Specify the methods used to collect data from reports, including how many reviewers collected data from each report, whether they worked independently, any processes for obtaining or confirming data from study investigators, and, if applicable, details of automation tools used in the process	8-9, Table 1 and protocol
Data items	#10a	List and define all outcomes for which data were sought. Specify whether all results that were compatible with each outcome domain in each study were sought (for example, for all measures, time points, analyses), and, if not, the methods used to decide which results to collect	n/a

For peer review only

Study risk of bias assessment	#11	Specify the methods used to assess risk of bias in the included studies, including details of the tool(s) used, how many reviewers assessed each study and whether they worked independently, and, if applicable, details of automation tools used in the process	n/a
Effect measures	#12	Specify for each outcome the effect measure(s) (such as risk ratio, mean difference) used in the synthesis or presentation of results	n/a
Synthesis methods	#13a	Describe the processes used to decide which studies were eligible for each synthesis (such as tabulating the study intervention characteristics and comparing against the planned groups for each synthesis (item #5))	8-9, Table 1, protocol
Synthesis methods	#13b	Describe any methods required to prepare the data for presentation or synthesis, such as handling of missing summary statistics or data conversions	n/a
Synthesis methods	#13c	Describe any methods used to tabulate or visually display results of individual studies and syntheses	n/a
Synthesis methods	#13d	Describe any methods used to synthesise results and provide a rationale for the choice(s). If meta-analysis was performed, describe the model(s), method(s) to identify the presence and extent of statistical heterogeneity, and software package(s) used	8-9, Table 1, protocol
Synthesis methods	#13e	Describe any methods used to explore possible causes of heterogeneity among study results (such as subgroup analysis, meta-regression)	n/a

For peer review only

Synthesis methods	#13f	Describe any sensitivity analyses conducted to assess robustness of the synthesised results	n/a
Reporting bias assessment	#14	Describe any methods used to assess risk of bias due to missing results in a synthesis (arising from reporting biases)	n/a
Certainty assessment	#15	Describe any methods used to assess certainty (or confidence) in the body of evidence for an outcome	n/a
Data items	#10b	List and define all other variables for which data were sought (such as participant and intervention characteristics, funding sources). Describe any assumptions made about any missing or unclear information	n/a
**Results**			
Study selection	#16a	Describe the results of the search and selection process, from the number of records identified in the search to the number of studies included in the review, ideally using a flow diagram (http://www.prisma-statement.org/PRISMAStatement/FlowDiagram)	9, Figure 1
Study selection	#16b	Cite studies that might appear to meet the inclusion criteria, but which were excluded, and explain why they were excluded	n/a
Study characteristics	#17	Cite each included study and present its characteristics	Supplementary File 3
Risk of bias in studies	#18	Present assessments of risk of bias for each included study	n/a
Results of individual studies	#19	For all outcomes, present for each study (a) summary statistics for each group (where appropriate) and (b) an effect estimate and its	n/a

For peer review only

		precision (such as confidence/credible interval), ideally using structured tables or plots	
Results of syntheses	#20a	For each synthesis, briefly summarise the characteristics and risk of bias among contributing studies	n/a
Results of syntheses	#20b	Present results of all statistical syntheses conducted. If meta-analysis was done, present for each the summary estimate and its precision (such as confidence/credible interval) and measures of statistical heterogeneity. If comparing groups, describe the direction of the effect	n/a
Results of syntheses	#20c	Present results of all investigations of possible causes of heterogeneity among study results	n/a
Results of syntheses	#20d	Present results of all sensitivity analyses conducted to assess the robustness of the synthesised results	n/a
Risk of reporting biases in syntheses	#21	Present assessments of risk of bias due to missing results (arising from reporting biases) for each synthesis assessed	n/a
Certainty of evidence	#22	Present assessments of certainty (or confidence) in the body of evidence for each outcome assessed	n/a
**Discussion**			
Results in context	#23a	Provide a general interpretation of the results in the context of other evidence	16-18
Limitations of included studies	#23b	Discuss any limitations of the evidence included in the review	20-21
Limitations of the review methods	#23c	Discuss any limitations of the review processes used	20-21

For peer review only

Implications	#23d	Discuss implications of the results for practice, policy, and future research	19-20
**Other information**			
Registration and protocol	#24a	Provide registration information for the review, including register name and registration number, or state that the review was not registered	3
Registration and protocol	#24b	Indicate where the review protocol can be accessed, or state that a protocol was not prepared	6, 8
Registration and protocol	#24c	Describe and explain any amendments to information provided at registration or in the protocol	8-9
Support	#25	Describe sources of financial or non-financial support for the review, and the role of the funders or sponsors in the review	23
Competing interests	#26	Declare any competing interests of review authors	23
Availability of data, code, and other materials	#27	Report which of the following are publicly available and where they can be found: template data collection forms; data extracted from included studies; data used for all analyses; analytic code; any other materials used in the review	23

Notes:

- 4: 6, protocol
- 5: 8-9, protocol
- 6: 8, Supplementary File 2
- 7: Supplementary File 2
- 8: 8, Table 1 and protocol

For peer review only

- 9: 8-9, Table 1 and protocol
- 13a: 8-9, Table 1, protocol
- 13d: 8-9, Table 1, protocol
- 16a: 9, Figure 1
- 17: Supplementary File 3 The PRISMA checklist is distributed under the terms of the Creative Commons Attribution License CC-BY. This checklist was completed on 25. April 2022 using https://www.goodreports.org/, a tool made by the EQUATOR Network in collaboration with Penelope.ai

For peer review only

Supplementary File 2

This file provides the full details of the search strategies employed in the review to identify documents containing relevant data.

Searches to locate existing theories (Step 1)

We searched PubMed and Web of Science (Core Collection) using a combination of terms relating to HCs, and terms relating to theory, adopting a slightly modified version of Booth and Carroll's (2015) 'BeHEMoTh' approach. As the HC programme is a specific health context (H) and we were interested in theorisation of all steps in the programme, no individual behaviours (Be) were specified.

PubMed (11th October 2020)

1. "Health Check" OR "Health Checks" (3847)
2. UK OR United Kingdom OR England OR Britain OR British (1790010)
3. model OR models OR modelling OR theor* OR concept* OR framework* (4625627)
4. 1 AND 2 AND 3 (130 hits)

Web of Science (SCI-EXPANDED, SSCI, AHCI, ESCI) (11th October 2020)

1. "Health Check" OR "Health Checks" (Topic) (3474)
2. UK OR "United Kingdom" OR England OR Britain OR British (Topic) (623802)
3. Model OR models OR modelling OR theor* OR concept* OR framework* (Topic) (11284654)
4. 1 AND 2 AND 3 (70 hits)

These searches identified 11 studies utilising 6 theoretical models or frameworks. An additional search for each of these named theories, plus terms relating to the NHSHC, was conducted in Google Scholar. In addition, we also searched Google Scholar for papers relating to the NHSHC that also cited the key citations related to each identified theory. These two steps were conducted in October 2020 and identified a further 8 (5+3) papers.

Searches for evidence on NHSHC (Step 2)

MEDLINE (via Ovid, 11th November 2020)

1. health check*.ti,ab,kw (6084)
2. (NHS or National Health Service or United Kingdom or UK or England or English).ti,ab,kw (370078)
3. exp England/ (107308)
4. 2 or 3 (440075)
5. 1 and 4 (468)

For peer review only

6. limit 5 to (english language and yr=”2008-Current”) (325)

Embase (via Ovid, 11th November 2020)

1. health check*.ti,ab,kw (8572)
2. (NHS or National Health Service or United Kingdom or UK or England or English).ti,ab,kw (487112)
3. england/ (24573)
4. 2 or 3 (494513)
5. 1 and 4 (521)
6. limit 5 to (english language and yr=”2008-Current”) (459)

CINAHL (via EbscoHost, 11th November 2020)

1. TX “health check* (2391)
2. TI (NHS OR “National Health Service” OR “United Kingdom” OR UK OR England OR English) OR AB (NHS OR “National Health Service” OR “United Kingdom” OR UK or England OR English) (178298)
3. MH “England” (62863)
4. S2 OR S3
5. S1 AND S4
6. Limiters: Published Date: 20080101-20201231; English Language; Expanders: Apply equivalent subjects (378)

HMIC (via Ovid, 11th November 2020)

1. health check*.mp (598)
2. (NHS or National Health Service or United Kingdom or UK or England or English).mp (118285)
3. exp england/ (26501)
4. exp health authorities in england/ (6680)
5. or/2-4 (135421)
6. 1 and 5 (321)
7. limit 6 to (english language and yr=”2008-Current”) (191)

Web of Science (SCI-EXPANDED, SSCI; 11th November 2020)

1. TOPIC: (“health check*”) (5144)
2. TOPIC: (NHS OR “national health service” OR “united kingdom” OR UK OR England OR English) (522053)
3. #1 AND #2 (358)

For peer review only

4. Refined by: PUBLICATION YEARS: (2020 OR 2012 OR 2019 OR 2011 OR 2018 OR 2010 OR 2017 OR 2009 OR 2016 OR 2008 OR 2015 OR 2014 OR 2013) AND LANGUAGES: (ENGLISH) (309)

Additional searches for theory

A short series of highly focused searches for documents describing street-level bureaucrats or street-level bureaucracy were run in Google Scholar in October and November 2021. The details of these searches and results screened (on screen) are provided below.

Google Scholar, 6th October 2021

("street level bureaucracy" OR "street level bureaucrats") AND ("public health") (8,160)

("street level bureaucracy" OR "street level bureaucrats") AND ("primary care") (2090)

("street level bureaucracy" OR "street level bureaucrats") AND ("general practice" OR “general practices”) (889)

("street level bureaucracy" OR "street level bureaucrats") AND ("local authority" OR “local authorities”) (6320)

For peer review only

Supplementary File 3

This file provides the full details of the documents included in the review.

First author	Year	Document type	Aim or purpose of document	Study design (if applicable)	Perspective(s)	Local area(s)	Commis-sioning period[a]	Contributed data to CMOCs			Total contrib-utions to CMOCs	Source
								C-CMOCs	P-CMOCs	A-CMOCs		
Alageel(1)	2018 (1)	Conference presentation	Summary of results of qualitative interview study (Alageel 2018)	Qualitative interview study	Providers	Lambeth and Lewisham	LA		P7		1	NHSHC website
Alageel(2)	2018 (2)	Journal article	To identify barriers and facilitators to implementing multiple health behaviour change interventions for CVD risk reduction in primary care	Qualitative interview study	Providers	Lambeth and Lewisham	LA	C14	P1, P2, P6, P7, P8, P10, P11, P13, P15, P20, P22, P24, P27	A1, A2, A27, A30, A32, A35, A38, A39	22	Existing review
Alageel(3)	2020	Journal article	To examine factors that might influence engagement and adherence to lifestyle change interventions and medication amongst people recently	Qualitative interview study	Attendees	Lambeth and Lewisham	LA	C14	P23	A2, A6, A10, A12, A15, A25, A28, A30, A31, A32, A35, A36, A38, A43, A44	17	Existing review

For peer review only

			assessed at medium or high risk of CVD									
Alford(4)	2010	Evaluation report	To explore behaviour changes made following community based NHSHC and experience of NHSHCs	Qualitative interview study	Attendees	Knowsley	PCT			A40, A44	2	Existing review
Al-Osaimi(5)	2020	Conference abstract	To describe the implementation of software to manage the NHSHC	n/a	Commissioners	Sandwell	LA	C2, C7			2	NHSHC website
Artac(6)	2013	Journal article	To assess whether the NHSHC was associated with a reduction in CVD risk after one year	Pre/post	Attendees	Hammersmith and Fulham	PCT		P12		1	Existing review
Atkins(7)	2020	Journal article	To identify behaviours and actors relevant to uptake, delivery and follow up of NHSHCs and influences on those behaviours	Systematic review	n/a	n/a	n/a	C14		A35	2	Searches - MEDLINE

For peer review only

Baker(8)	2014 (1)	Evaluation report	To assess the impact of the NHSHC programme and inform future commissioning (summary)	Cross-sectional	Attendees	Gloucestershire	PCT	C9	P12, P17		3	NHSHC website
Baker(9)	2014 (2)	Journal article	To investigate the perceptions and opinions of patients who attended an NHSHC	Survey	Attendees	Gloucestershire	PCT	C14	P1, P8, P20, P25	A1, A15, A18, A22, A23, A26, A34	12	Existing review
Baker(10)	2015 (1)	Journal article	To investigate health professionals' experiences and perspectives of implementation of the NHSHC	Survey including qualitative analysis of free text responses	Providers	Gloucestershire	PCT	C2	P10, P11, P18		4	Existing review
Baker(11)	2015 (2)	Journal article	To investigate how the local NHSHC pathway was followed and interpreted	Cross-sectional	n/a	Gloucestershire	PCT		P2, P12		2	Existing review
Bell(12)	2019	Conference abstract	To describe the outcomes of NHSHCs delivered in an integrated model with lifestyle services	Cross-sectional	Commissioners	Medway	LA	C3	P19		2	NHSHC website

For peer review only - http://bmjopen.bmj.com/site/about/guidelines.xhtml

Boase(13)	2012	Journal article	To explore the perspectives of practice nurses in their role of communicating CVD risk to patients	Qualitative interview study	Providers	Cambridge	PCT		P4, P22, P27	A8, A19	5	Searches - MEDLINE
Boseley(14)	2020	Conference presentation	To describe a local model of NHSHC delivery with integrated lifestyle services	Mixed methods	Commissioners	East Sussex	LA	C3, C6, C7	P14, P15, P19	A4	7	NHSHC website
Brutus(15)	2013	Evaluation report	To review the design of the local NHSHC programme and analyse delivery options available	Cross-sectional	Various	Croydon	PCT	C1, C2, C7	P1, P13, P15, P19	A6, A40	9	NHSHC website
Burgess(16)	2016	Conference presentation	Summary results of a qualitative interview study (Alageel 2020)	Qualitative interview study	Attendees	Lambeth and Lewisham	LA		P1, P23	A2, A5, A10, A20, A23, A28, A33, A42	10	NHSHC website
Burke(17)	2015	Conference presentation	To describe local delivery of NHSHCs with a focus on vulnerable groups	n/a	Commissioners	Leeds	LA			A5, A38	2	NHSHC website
Carter(18)	2016	Journal article	To evaluate local outcomes of NHSHCs with a focus on diagnosis and management after checks	Retrospective cohort	Attendees	Leicester	PCT, Transition	C1			1	Existing review

For peer review only

Centre for Public Scrutiny(19)	2014	Other report	A report on the role of Council Scrutiny in local reviews of NHSHC programmes	n/a	n/a	Various	LA	C1, C11	P1		3	Searches - HMIC
Chatterjee(20)	2017	Journal article	To assess the knowledge, use, and confidence in national physical activity and Chief Medical Officer guidelines and tools among GPs in England	Survey	Providers	England	LA		P21		1	Searches - MEDLINE
Chipchase(21)	2011	Evaluation report	To explore people's views and experiences of the NHSHC	Qualitative interview study	Various	Birmingham	PCT		P1, P14	A1, A2, A11, A12, A19, A20, A40	9	Existing review
Clarke(22)	2020	Conference presentation	To describe the of data to inform local CVD prevention work	n/a	Commissioners	Barnsley	LA	C8		A33	2	NHSHC website
Cochrane(23)	2012 (1)	Journal article	To compare changes in population CVD risk factors between those who receive an NHSHC and those who receive an NHSHC with additional	RCT	n/a	Stoke on Trent	PCT		P26	A9	2	Existing review

For peer review only

			lifestyle support									
Cochrane(24)	2013	Journal article	To review response, attendance and treatment uptake following NHSHCs	Cross-sectional	n/a	Stoke on Trent	PCT			A9	1	Existing review
Coffey(25)	2014	Evaluation report	To assess the level of uptake of NHSHCs	Cross-sectional	n/a	Salford	PCT		P12, P20		2	Existing review
Coghill(26)	2018	Journal article	To assess uptake and outcomes of NHSHCs in general practice	Cross-sectional	n/a	Bristol	PCT, Transition, LA		P12, P16		2	Existing review
Collins(27)	2011	Evaluation report	To evaluate the effectiveness of an 'outreach' delivery model for NHSHCs	Mixed methods	n/a	Medway	PCT		P17	A4	2	NHSHC website
Corlett(28)	2016	Journal article	To assess the findings of NHSHCs delivered in pharmacy settings, referrals to GPs and client views	Mixed methods	n/a	Lewisham	Transition, LA			A2, A13, A15, A25, A35, A38	6	Existing review
Coward(29)	2020	Conference presentation	To describe processes linking NHSHCs with	n/a	n/a	Dorset	LA	C2, C7, C8	P17, P19, P26, P27, P28		8	NHSHC website

For peer review only

			local services that aim to promote physical activity									
Cupit(30)	2018	PhD thesis	To explore how patients' and healthcare professionals' knowledge and practices about CVD prevention are socially organised	Ethnography	n/a	Unknown	LA	C8, C13, C14	P2, P4, P9, P10, P20, P24	A11, A23, A25, A27, A31, A35, A38, A44	17	NHSHC website
Dalton(31)	2011	Journal article	To examine the uptake of NHSHCs and statin prescribing	Cross-sectional	n/a	Ealing	PCT		P2, P3		2	Existing review
Derbyshire County Council(32)	2015	Evaluation report (Health Equity Audit)	To identify health inequalities in relation to the NHSHC programme	Cross-sectional	Commissioner	Derbyshire	LA	C9, C10	P2, P12, P15, P16, P17, P20, P22, P25	A35	11	NHSHC website
Edmans(33)	2013	Other report	To describe local training provision in relation to the dementia component of NHSHCs	n/a	Providers	Southwark	LA		P20		1	NHSHC website
Fenton(34)	2018	Conference presentation	To describe local CVD prevention work and NHSHCs	Cross-sectional	n/a	Southwark	LA		P1, P3		2	NHSHC website
Forster(35)	2015	Journal article	To evaluate the 'yield' of NHSHCs (in relation to identification of	Retrospective cohort	n/a	England	PCT, Transition, LA		P2, P6, P12, P16		4	Existing review

For peer review only

			risk factors, diagnoses and prescribing)									
Forsyth(36)	2012	Conference abstract	To test a delivery model using pharmacists to deliver health checks to the South Asian community (NB describes checks delivered in Scotland)	Mixed methods	n/a	Glasgow	PCT			A17	1	Searches - Embase
Frazer(37)	2020	Conference presentation	To describe the delivery of NHSHCs with integrated lifestyle services	n/a	Commissioners	West Sussex	LA	C3			1	NHSHC website
Gidlow(38)	2020	Journal article	To examine the content of NHSHC, patient-practitioner communication balance and differences when using QRISK2 versus JBS3 CVD risk calculators	Observation (video recorded NHSHCs)	n/a	West Midlands	LA		P10, P20	A6, A13, A39	5	Searches - MEDLINE
Gidlow(39)	2021 (1)	Research report	To explore the extent of physical activity and alcohol measurement in the NHSHC	Observation (video recorded NHSHCs)	n/a	West Midlands	LA		P20		1	NHSHC website

Gidlow(40)	2021 (2)	Research report	To explore practitioner and patient understanding of CVD risk, associated advice or treatment and the response of patients to the NHSHC supported by the QRISK2 or JBS3 risk calculators	Mixed methods	n/a	West Midlands	LA		P10	A9, A10, A13, A29	4	Search alerts
Graley(41)	2011	Journal article	To describe a 'postcode lottery' effect in relation to NHSHCs	Cross-sectional	Commissioner	North West London	PCT	C9, C10, C13, C14	P12, P13		6	Existing review
Greaves(42)	2015	Journal article	To assess the feasibility of delivering an intervention to promote healthy eating and physical activity and of conducting a full scale RCT	Pilot RCT	n/a	Bath	PCT		P28	A30, A37, A40	4	Searches - MEDLINE
Green(43)	2018	Evaluation report	To understand the NHSHC patient journey from invitation through to behaviour change or clinical intervention	Mixed methods	Attendees	East Sussex	LA	C7	P10, P24	A1, A8, A12, A24, A29, A35	9	Stakeholder group
Gregory(44)	2018	Conference presentation	To describe the role of healthcare professionals	n/a	n/a	England	LA		P25		1	NHSHC website

For peer review only

			in treatment of tobacco dependency									
Gulliford(45)	2018	Journal article	To compare CVD risk scores for 'invited' and 'opportunistic' NHSHCs	Retrospective cohort	n/a	Lambeth and Lewisham	Transition, LA		P11		1	Existing review
Hardman(46)	2014	Evaluation report	To describe local delivery of an NHSHC programme with Health Trainers	n/a	Various	Bolton	PCT	C3	P7, P14, P19	A13, A30	6	NHSHC website
Haringay Council(47)	2012	Evaluation report	Scrutiny review' focused on men's health needs and life expectancy gap	Mixed methods	n/a	Haringay	PCT	C7		A10, A13, A14, A16, A30, A37	7	NHSHC website
Hawking(48)	2018	Conference poster	To describe a visual risk communication tool designed for use in NHSHCs	n/a	Providers, attendees	Newham	LA			A44	1	NHSHC website
Hawking(49)	2019	Journal article	To explore patient perspectives and experiences of a personalised risk report during an NHSHC	Qualitative interview study	Attendees	Newham	LA		P28	A1, A9, A10, A18, A19, A20, A40, A44	9	Existing review
Hinde(50)	2017	Journal article	To assess the cost-effectiveness of NHSHCs	Economic evaluation	n/a	England	LA	C12, C13			2	Existing review

For peer review only

Homer(51)	2015	Journal article	To determine the characteristics of patients prescribed statins for primary prevention according to their CVD risk	Cross-sectional	n/a	Newham, City & Hackney, Tower Hamlets (London)	LA		P3		1	Serendipity
Honey(52)	2013	Journal article	To identify the attitudes of primary healthcare professionals towards the delivery of lifestyle advice in NHSHCs	Q methodology	Providers	Leeds	PCT		P2, P6, P7, P18, P22, P24, P27		7	Searches - MEDLINE
Honey(53)	2015	Journal article	To examine the perspectives of patients identified as being at 'high risk' of CVD events	Qualitative interview study	Attendees	Leeds	PCT		P27	A2, A3, A4, A5, A7, A9, A10, A13, A19, A20, A22, A27, A29	14	Searches - MEDLINE
Hooper(54)	2014	Journal article	To estimate the case detection of five health conditions by NHSHCs	Cross-sectional	Attendees	Warwickshire	PCT	C10			1	Existing review
Hyseni(55)	2020	Journal article	To report the results of a workshop that aimed to facilitate engagement with stakeholders who will be involved in co-	Qualitative interview study	Commissioners, Providers, Policymakers	Various	LA	C5, C7, C11, C12, C13, C14	P12		7	Searches - MEDLINE

For peer review only

			production of an NHSHC modelling tool for commissioners									
Ismail(56)	2015	Journal article	To explore the challenges and barriers faced by staff involved in the delivery of the NHSHC	Qualitative interview study	Providers	Leeds	LA	C1, C7, C9, C14	P6, P10, P12	A35, A36, A44	10	Existing review
Ismail(57)	2016	Journal article	To provide an insight into the process of patients receiving NHSHCs and determine the extent to which they were supported to reduce CVD risk through behaviour change	Qualitative interview study	Attendees	Leeds	PCT		P2, P27	A2, A6, A8, A11, A12, A30, A31, A38	10	Existing review
Ismail(58)	2019	Research report (NIHR)	To test the clinical and cost-effectiveness of an 'enhanced lifestyle motivational interviewing' intervention for patients at high risk of CVD in group and individual	RCT	n/a	South London	LA		P6, P7	A6, A16, A17, A26, A27, A30, A31, A32, A34, A40, A44	13	Searches - WoS

For peer review only

			settings, compared with usual care									
Jones(59)	2020	Conference poster	To describe local CVD prevention work and NHSHCs	n/a	n/a	Telford	LA / CCG	C1	P6, P17	A14	4	NHSHC website
Kearney(60)	2015	Conference presentation	To describe factors that influence GP engagement with NHSHCs and potential solutions	n/a	n/a	n/a	LA	C4	P1		2	NHSHC website
Kennedy(61)	2019	Journal article	To evaluate uptake, risk factor detection and management from the NHSHC	Quasi-RCT	n/a	Hampshire	PCT, Transition, LA		P13		1	Existing review
Kirkpatrick(62)	2016	Conference presentation	To describe staff development and training for NHSHC providers	n/a	Providers	Salford	LA		P8		1	NHSHC website
Krska(63)	2010	Evaluation report	To evaluate a CVD screening service offered in pharmacy settings and obtain views of prospective users about	Mixed methods	n/a	Sefton	PCT		P25	A2	2	NHSHC website

For peer review only

			the acceptability of the service									
Krska(64)	2013	Conference abstract	To compare the views of the general public, pharmacists and patients invited to the NHSHC and to compare experiences of NHSHCs delivered by pharmacies and general practices	Survey and qualitative interviews	Providers, attendees	Sefton	PCT			A14	1	Searches - Embase
Krska(65)	2014	Journal article	To explore the views and experiences of patients with potentially high-CVD risk	Survey	Attendees	Sefton	PCT		P7, P10	A15	3	Existing review
Krska(66)	2016 (1)	Journal article	To seek the views of GPs and practice managers on NHSHC implementatio n	Survey	Providers	Sefton	PCT	C9	P10, P13		3	Existing review
Krska(67)	2016 (2)	Journal article	To evaluate NHSHC implementatio n in relation to data recording, advice provided, referrals,	Cross-sectional	Providers	Sefton	PCT	C10			1	Existing review

For peer review only

			prescribing and new diagnoses									
Kumar(68)	2015	Conference presentation	To describe work to improve GP engagement with NHSHCs	n/a	n/a	Stoke on Trent, Havering (London)	LA	C1			1	NHSHC website
Lake(69)	2010	Conference presentation	To describe a community based NHSHC service	n/a	n/a	Camden	PCT	C11	P19, P28	A5, A14	4	NHSHC website
Lambert(70)	2016	Journal article	To assess indicators of programme reach available to local service commissioners	Assessment of indicators	n/a	NE England	PCT, Transition		P12		1	Existing review
Lennon(71)	2020	Conference presentation	To describe local responses to Covid-19 in relation to NHSHCs and social prescribing	n/a	n/a	Redbridge	LA	C6			1	Stakeholder group
Liverpool City Council(72)	2020	Conference poster	To describe the local Health Trainer service available to NHSHC attendees	n/a	n/a	Liverpool	LA		P6, P8, P19, P26, P27, P28	A6, A14, A16, A30, A36, A40	12	NHSHC website
Local Government Association(73)	2015	Other report	Report on the transfer of public health responsibilities to LAs	n/a	Commissioners	Various	LA	C1, C5, C8, C11, C12, C13			6	Existing review

For peer review only

Local Authority A(74)[b]	2019	Evaluation report	Evaluation of local NHSHC programme	Mixed methods	Commissioners	[Redacted]	LA	C1, C2, C11	P16, P17	A15	6	Survey respondent
Local Authority B(75)[b]	2019	Evaluation report	Evaluation of local NHSHC programme	Mixed methods	Commissioners	[Redacted]	LA	C7, C11, C12			3	Survey respondent
Local Authority C(76)[b]	nd	Working document	Report describing quality assurance considerations in relation to NHSHCs	n/a	Commissioners	[Redacted]	LA	C1, C2, C4, C7	P9		5	Survey respondent
Local Authority C(77)[b]	2019	Evaluation report	To measure, monitor and report on performance and quality of NHSHCs against a quality framework	Mixed methods	Commissioners	[Redacted]	LA	C2, C7, C10, C12			4	Survey respondent
London Borough of Bromley(78)	2018	Conference presentation	To assess the prevention of diabetes through NHSHCs	Cross-sectional	n/a	Bromley	LA	C14	P8, P25		3	NHSHC website
Loo(79)	2011	Conference abstract	To derive information on community pharmacists' activities and attitudes in relation to NHSHCs	Survey	Providers	Various	PCT		P13		1	Existing review
Lumley(80)	2015	Conference presentation	To describe the role of the NHSHC in addressing high blood pressure	n/a	Commissioners, providers	Blackpool	LA	C1, C7			2	NHSHC website

For peer review only

For peer review only - http://bmjopen.bmj.com/site/about/guidelines.xhtml

Maddern(81)	2020	Conference abstract	To describe the role of local political scrutiny in developing and improving NHSHCs	n/a	Commissioners	Wiltshire	LA	C11			1	NHSHC website
Martin(82)	2011	Practitioner article	To describe challenges in delivery of NHSHCs	n/a	Provider	East Sussex	PCT	C13	P14		2	Searches - CINAHL
McDermott(83)	2015	Conference presentation	Summary of research exploring the implementation of NHSHCs in primary care settings	Qualitative interview study	Providers	Lambeth and Lewisham	PCT, Transition	C9	P1, P10, P13		4	NHSHC website
McMillan(84)	2018	Journal article	Report on a workshop with members of the public to inform design of a 'digital adjunct' intervention for the NHSHC	Co-production	Attendees	Manchester	LA			A6, A44	2	Searches - MEDLINE
McNaughton (85)	2011	Journal article	To evaluate delivery of the NHSHC in community pharmacies	Qualitative interview study	Providers	Tees Valley	PCT	C13	P20, P27	A14, A15	5	Existing review
McNaughton (86)	2014	Journal article	To understand factors that influenced adherence to medication and advice in 'high risk' patients identified by the NHSHC	Qualitative interview study	Attendees	North East England	PCT			A3, A9, A10, A25, A27, A28, A35, A38, A40, A42	10	Existing review

For peer review only

Mills(87)	2017	Journal article	To synthesise data concerning the views of commissioners, managers and healthcare professionals towards the NHSHC	Systematic review	Commissioners, providers	n/a	n/a		P10, P13		2	Searches - MEDLINE
Nahar(88)	2020	Journal article	Protocol for a mixed methods implementation study of a community based CVD risk assessment and coaching intervention	Mixed methods (protocol)	n/a	Sussex, Nottingham	LA			A14	1	Searches - MEDLINE
Nasir(89)	2018	Conference abstract	To describe a local project that aimed to increase NHSHC uptake and referrals to local lifestyle services	Cross-sectional	n/a	Kingston (London)	LA			A35, A37	2	NHSHC website
Newman(90)	2018	Conference presentation	To describe Health Coaching techniques	n/a	n/a	n/a	LA		P27		1	NHSHC website
NHS Greenwich(91)	2011	Evaluation report	To assess the outcomes of a community-based NHSHC delivery model	Mixed methods	n/a	Greenwich	PCT		P14	A4, A13, A17	4	Existing review
NHS Midlands and East(92)	2011	Evaluation report	To evaluate a pilot programme aiming to	Mixed methods	n/a	Bedfordshire; Great Yarmouth	PCT	C2, C7	P1, P7, P10, P17, P19	A40	8	NHSHC website

For peer review only

			deliver lifestyle interventions through the NHSHC			and Waveney						
NICE(93)	2014	Guidance	Guidance for local authorities on commissioning and delivery of the NHSHC	n/a	Commissioners	England	LA	C11			1	Searches - HMIC
Nicholas(94)	2012	Journal article	To evaluate the organisation of the NHSHC in general practices	Survey	Providers	Lambeth and Lewisham	PCT		P2		1	Existing review
O'Flaherty(95)	2021	Research report	To develop a model for commissioners to quantify cost-effectiveness and potential for equitable population health again of the NHSHC	Mixed methods	Commissioners	Various	LA	C7			1	Search alerts
Onyia(96)	2016	Conference presentation	To describe the impact of Health Trainers on uptake of lifestyle services after NHSHCs	Cross-sectional	n/a	Halton	LA		P6, P8, P25, P26		4	NHSHC website
Oswald(97)	2010	Evaluation report	To evaluate the implementation ad outcomes of the 'Tees Vascular Assessment Programme'	Mixed methods	n/a	Tees Valley	PCT	C8, C9, C13, C14	P1, P2, P7, P11, P14, P28	A1, A2, A4, A9, A13, A10, A18, A28, A29, A31, A32, A33, A40, A41, A42, A43	26	Existing review

For peer review only

			(precursor to NHSHC)									
Palladino(98)	2020	Journal article	To assess associations between coverage of the NHSHC and detection and management of incident cases of non-diabetic hyperglycaemia and type 2 diabetes	Retrospective cohort	n/a	England	PCT		P5		1	Searches - MEDLINE
Patel(99)	2020	Journal article	To describe the uptake and outputs of the NHSHC programme	Cross-sectional	n/a	England	Transition, LA		P3, P12		2	Searches - MEDLINE
Paxton(100)	2020	Journal article	To assess the fidelity of delivery of NHSHCs in general practice	Fidelity assessment	n/a	East of England	PCT		P20, P21		2	Searches - MEDLINE
Perkins(101)	2020	Conference presentation	Presentation describing CVD prevention initiatives	n/a	n/a	England	LA			A2, A38	2	NHSHC website
Perry(102)	2014	Journal article	To explore experiences of engaging with a community-based NHSHC	Qualitative interview study	Attendees	Knowsley	PCT		P14, P28	A2, A4, A7, A13, A18, A25, A35, A38, A40, A44	12	Existing review

For peer review only

Public Health England(103)	2020	Guidance	Best Practice Guidance for commissioners and providers	n/a	n/a	England	LA		P12	A12	2	NHSHC website
Rawlinson(104)	2019	Journal article	To evaluate an 'enhanced health promotion service' in a physiotherapy-led musculoskeletal service providing NHSHCs and diabetes checks	Mixed methods	n/a	Salford	LA	C6	P6, P7, P8, P14, P17, P19, P26		8	Searches - CINAHL
Research Works(105)	2013	Evaluation report	To assess commissioners' and providers' experiences of the NHSHC and gain an understanding of the engagement of public health professionals with NHSHCs	Qualitative interview study	Commissioners, providers	Various	PCT, Transition	C1, C4, C7, C11, C13	P7, P9, P10, P13, P14, P19	A13	12	Existing review
Richardson(106)	2016	News article	Article responding to research demonstrating lower coverage of NHSHCs and limited evidence of effectiveness of NHSHCs	n/a	n/a	n/a	LA		P18	A16	2	Searches - CINAHL

For peer review only - http://bmjopen.bmj.com/site/about/guidelines.xhtml

Riley(107)	2015	Journal article	To examine the feasibility and acceptability of community outreach NHSHCs targeted at the Afro-Caribbean community	Ethnography	Providers, attendees	Bristol	LA		P10	A2, A3, A13, A14, A16, A17, A21, A23, A25, A26, A40, A44	13	Existing review
Riley(108)	2016	Journal article	To examine the experiences of patients attending and healthcare professionals conducting NHSHCs	Qualitative interview study	Providers, attendees	Bristol	LA		P4, P8, P10, P27	A1, A3, A11, A19, A20, A21, A22, A25, A33, A35, A38, A44	16	Existing review
Robson(109)	2015	Journal article	To describe implementation and results of the NHSHCs	Cross-sectional	n/a	City & Hackney, Newham, Tower Hamlets	PCT		P9		1	Existing review
Robson(110)	2017	Journal article	To describe the coverage and impact of NHSHCs on cardiovascular risk management and identification of new comorbidities	Retrospective cohort	n/a	City & Hackney, Newham, Tower Hamlets	PCT, Transition, LA		P9		1	Existing review

For peer review only

Saramunee(111)	2015	Journal article	To explore the experience of and willingness to use seven pharmacy public health services related to cardiovascular risk among the general public in England (including the NHSHC)	Mixed methods	Attendees	Sefton				A15, A29	2	Searches - MEDLINE
Shaw(112)	2015	Journal article	To explore health care professionals' and patients' experiences of delivering and receiving the NHSHC	Qualitative interview study	Providers, attendees	Birmingham	PCT	C14	P8, P12, P23, P24, P27	A2, A3, A4, A6, A9, A24, A26, A32, A35, A36, A38, A40	18	Existing review
Shaw(113)	2016	Journal article	To evaluate the implementation of the NHSHC from the perspective of GPs	Qualitative interview study	Providers	Birmingham	PCT		P1, P5, P6, P9, P10, P11, P13, P17, P20, P21, P26, P27	A1, A6, A10, A12, A20, A30, A39	19	Existing review
Simon(114)	2020	Conference presentation	Presentation describing physical activity interventions for prevention and management of health conditions	n/a	n/a	England	LA		P26		1	NHSHC website

For peer review only

Solutions Strategy(115)	2017	Evaluation report	To evaluate the pilot and assess the feasibility of extending the NHSHC to include a dementia risk reduction component	Mixed methods	n/a	Birmingham, Bury, Manchester, Southampton			P26	A13, A15, A26	4	Searches - HMIC
Strutt(116)	2011	PhD thesis	To investigate patients' experiences of and responses to NHSHCs	Qualitative interview study / ethnography	Attendees	n/a	PCT		P1, P16	A2, A5, A6, A7, A11, A13, A15, A20, A21, A22, A25, A26, A29, A30, A34, A35, A44	19	Existing review
Tanner(117)	2020	Research report	To update PHE-commissioned rapid evidence synthesis and summarise evidence to address several research questions relating to the implementation of NHSHCs	Systematic review	Various	n/a	n/a		P2		1	NHSHC website
Thompson(118)	2016	Conference presentation	Presentation describing PHE's 'StARS' framework for assessment of NHSHCs	n/a	Commissioners	England	LA	C11			1	NHSHC website
Thompson(119)	2019	Conference abstract	Summary of implementation and outcomes of an integrated	Cross-sectional	Commissioner	Slough	LA	C3	P19		2	NHSHC website

			cardiovascular service									
Trueland(120)	2013	News article	Article based around an interview with NHSHC supporter Professor Michael Kirby	n/a	Commissioners	n/a	LA	C8			1	Searches - HMIC
Turner(121)	2013	Journal article	To evaluate variation in statin take up following risk assessment in the NHSHC	Qualitative interview study	Attendees	Nottingham	PCT			A15, A28, A41, A42, A43	5	Searches - HMIC
Usher-Smith(122)	2017	Research report	To provide a rapid synthesis of published research evidence on NHSHCs	Systematic review	n/a	n/a	n/a		P8		1	NHSHC website
Visram(123)	2012	Evaluation report	To investigate the implementation and accessibility of the 'health trainer community check' service	Mixed methods	Various	Durham	PCT, Transition	C1, C5, C9	P6, P12, P19, P27	A2, A3, A4, A5, A31, A32, A33	14	NHSHC website
Williams(124)	2018	Conference presentation	Presentation describing physical activity training and use of the GPPAQ tool to assess physical activity levels	n/a	n/a	England	LA		P21, P26		2	NHSHC website

nd=no date

For peer review only

[a]*Commissioning period here refers to whether the data included in the document relate to the period when NHSHCs were commissioned by PCTs or LAs. Documents contributing data that spans the 'transition' period (from mid-2012 to 2013) are also noted.*

[b] *Identifying details of LAs have been removed where unpublished material was shared by survey respondents*

For peer review only

Reference list (included documents)

1. Alageel S. Implementing multiple behaviour change interventions after health checks in primary care: a qualitative study. Cardiovascular Disease Prevention Conference 2018: Getting serious about prevention: reducing variation and optimizing care London, UK: NHS England; 2018.
2. Alageel S, Gulliford MC, McDermott L, Wright AJ. Implementing multiple health behaviour change interventions for cardiovascular risk reduction in primary care: a qualitative study. BMC Fam Pract. 2018;19(1):171.
3. Alageel S, Gulliford MC, Wright A, Khoshaba B, Burgess C. Engagement with advice to reduce cardiovascular risk following a health check programme: A qualitative study. Health Expect. 2020;23(1):193-201.
4. Alford S, Perry C. Knowsley at Heart community NHS health checks: Behaviour change evaluation. 2010. Accessed
5. Al-Osaimi A. Improving lifestyle support following the NHS Health Check programme; a whole system approach. Cardiovascular Disease Prevention Conference 2020: proactive, predictive, personalised Kia Oval, London: NHS England; 2020.
6. Artac M, Dalton AR, Majeed A, Car J, Millett C. Effectiveness of a national cardiovascular disease risk assessment program (NHS Health Check): results after one year. Prev Med. 2013;57(2):129-34.
7. Atkins L, Stefanidou C, Chadborn T, Thompson K, Michie S, Lorencatto F. Influences on NHS Health Check behaviours: a systematic review. BMC Public Health. 2020;20(1):1359.
8. Baker C, Loughren E, Crone D. Evaluation of the Gloucestershire NHS Health Check Programme, for the period July 2011 to July 2012. 2014. Available from: https://www.healthcheck.nhs.uk/commissioners-and-providers/evidence/local-evaluation/. Accessed
9. Baker C, Loughren EA, Crone D, Kallfa N. Patients' perceptions of a NHS Health Check in the primary care setting. Qual Prim Care. 2014;22:232-37.
10. Baker C, Loughren EA, Crone D, Kallfa N. Perceptions of health professionals involved in a NHS Health Check care pathway. Practice Nursing. 2015;26(12):608-12.
11. Baker C, Loughren EA, Crone D, Kallfa N. A process evaluation of the NHS Health Check care pathway in a primary care setting. J Public Health (Oxf). 2015;37(2):202-9.
12. Bell K. Integrating an outreach NHS Health Check Programme into a Lifestyle Interventions Hub. Cardiovascular Disease Prevention Conference 2019: Saving Hearts and Minds Together Manchester, UK2019.
13. Boase S, Mason D, Sutton S, Cohn S. Tinkering and tailoring individual consultations: how practice nurses try to make cardiovascular risk communication meaningful. J Clin Nurs. 2012;21(17-18):2590-8.
14. Boseley R. Personalised and proactive CVD Prevention: NHS Health Checks and Integrated Lifestyle services in East Sussex. Cardiovascular Disease Prevention Conference 2020: proactive, predictive, personalised Kia Oval, London: NHS England; 2020.
15. Brutus L, Fluke R. Croydon NHS Health Check Programme: Review and Options Appraisal. 2013. Available from: https://www.healthcheck.nhs.uk/commissioners-and-providers/evidence/local-evaluation/. Accessed
16. Burgess C. Engagement with advice to reduce cardiovascular risk following an NHS Health Check. NHS Health Check Conference: Getting Serious about Prevention London, UK: NHS England; 2016.

For peer review only - http://bmjopen.bmj.com/site/about/guidelines.xhtml

17. Burke D, Kirby H. Leeds patient insight and engagement from vulnerable groups: how to reduce inqualities. NHS Health Check 2015 - Improvement through collaboration 2015.
18. Carter P, Bodicoat DH, Davies MJ, Ashra NB, Riley D, Joshi N, et al. A retrospective evaluation of the NHS Health Check Programme in a multi-ethnic population. J Public Health (Oxf). 2016;38(3):534-42.
19. Centre for Public Scrutiny. Checking the nation's health: the value of council scrutiny. London: CfPS, 2014; 2014. Available from: http://www.cfps.org.uk/includes/scripts/force_download.php?file=../../domains/cfps.org.uk/local/media/downloads/CfPS_Nations_Health_final_online.pdf. Accessed
20. Chatterjee R, Chapman T, Brannan MG, Varney J. GPs' knowledge, use, and confidence in national physical activity and health guidelines and tools: a questionnaire-based survey of general practice in England. Br J Gen Pract. 2017;67(663):e668-e75.
21. Chipchase L, Hill P, Waterall J. NHS Birmingham East & North. An insight into the NHS Health Check Programme in Birmingham: Summary Report. 2011. Accessed
22. Clarke R, Brierley A. Using NHS Health Checks data to galvanise public health prevention. Cardiovascular Disease Prevention Conference 2020: proactive, predictive, personalised Kia Oval, London: NHS England; 2020.
23. Cochrane T, Davey R, Iqbal Z, Gidlow C, Kumar J, Chambers R, et al. NHS health checks through general practice: randomised trial of population cardiovascular risk reduction. BMC Public Health. 2012;12:944.
24. Cochrane T, Gidlow CJ, Kumar J, Mawby Y, Iqbal Z, Chambers RM. Cross-sectional review of the response and treatment uptake from the NHS Health Checks programme in Stoke on Trent. J Public Health (Oxf). 2013;35(1):92-8.
25. Coffey M, Cooper A, Brown T, Cook P, Clarke-Cornwell A. Vascular health checks in Salford: an exploration using FARSITE data. University of Salford,; 2014. Accessed
26. Coghill N, Garside L, Montgomery AA, Feder G, Horwood J. NHS health checks: a cross- sectional observational study on equity of uptake and outcomes. BMC Health Serv Res. 2018;18(1):238.
27. Collins K-a. Evaluation of the NHS Health Check Outreach Programme in Medway. NHS Medway; 2011. Available from: https://www.healthcheck.nhs.uk/commissioners-and-providers/evidence/local-evaluation/. Accessed
28. Corlett SA, Krska J. Evaluation of NHS Health Checks provided by community pharmacies. J Public Health (Oxf). 2016;38(4):e516-e23.
29. Coward C. Understanding and accessing the local physical activity system. Cardiovascular Disease Prevention Conference 2020: proactive, predictive, personalised Kia Oval, London: NHS England,; 2020.
30. Cupit C. An ethnographic study of cardiovascular disease prevention: the social organisation of measures, knowledge, interventions and tensions in English general practice: University of Leicester; 2018.
31. Dalton AR, Bottle A, Okoro C, Majeed A, Millett C. Uptake of the NHS Health Checks programme in a deprived, culturally diverse setting: cross-sectional study. J Public Health (Oxf). 2011;33(3):422-9.
32. Derbyshire County Council, Services PHIaK. Health Equity Audit: NHS Health Check Programme in Derbyshire County. 2015. Available from: https://www.healthcheck.nhs.uk/commissioners-and-providers/evidence/local-evaluation/. Accessed

For peer review only

33. Edmans T. Training for the dementia component of the NHS Health Check, NHS Southwark. Southwark Council,; 2013. Available from: https://www.healthcheck.nhs.uk/commissioners-and-providers/evidence/case-studies/. Accessed
34. Fenton K. Getting serious about preventing cardiovascular disease. Cardiovascular Disease Prevention Conference 2018: Getting serious about prevention: reducing variation and optimizing care London, UK2018.
35. Forster AS, Dodhia H, Booth H, Dregan A, Fuller F, Miller J, et al. Estimating the yield of NHS Health Checks in England: a population-based cohort study. J Public Health (Oxf). 2015;37(2):234-40.
36. Forsyth P, Ali S, Ameen M, Khan R, Khan F, Sheikh AR, et al. Pharmacist-led anticipatory care for the South Asian community. International Journal of Pharmacy Practice. Royal Pharmaceutical Society, RPS Annual Conference 2012. Birmingham United Kingdom.

(var.pagings). 20: Pharmaceutical Press; 2012. p. 88-9.
37. Frazer H. Implementing NHS Health Checks within a non-clinical integrated lifestyle service. Cardiovascular Disease Prevention Conference 2020: proactive, predictive, personalised Kia Oval, London: NHS England; 2020.
38. Gidlow CJ, Ellis NJ, Cowap L, Riley VA, Crone D, Cottrell E, et al. Quantitative examination of video-recorded NHS Health Checks: comparison of the use of QRISK2 versus JBS3 cardiovascular risk calculators. BMJ Open. 2020;10(9):e037790.
39. Gidlow C, Riley V. Physical activity and alcohol measurement in NHS Health Checks. Staffordshire University, Public Health England; 2021. Available from: https://www.healthcheck.nhs.uk/commissioners-and-providers/evidence/. Accessed
40. Gidlow CJ, Ellis NJ, Cowap L, Riley V, Crone D, Cottrell E, et al. Cardiovascular disease risk communication in NHS Health Checks using QRISK®2 and JBS3 risk calculators: the RICO qualitative and quantitative study. Health Technol Assess. 2021;25(50).
41. Graley C, Katherine MF, McCoy DC. Postcode Lotteries in Public Health - The NHS Health Checks Programme in North West London. BMC Public Health. 2011;11(738).
42. Greaves C, Gillison F, Stathi A, Bennett P, Reddy P, Dunbar J, et al. Waste the waist: a pilot randomised controlled trial of a primary care based intervention to support lifestyle change in people with high cardiovascular risk. Int J Behav Nutr Phys Act. 2015;12:1.
43. Green K, Forshaw A, D'Souza H, Macherianakis A, MEL Research. NHS Health Check Patient Journey Evaluation: Final Evaluation Report. East Sussex County Council Public Health, MEL Research; 2018. Accessed
44. Gregory A, Chowdary Q, Tobacco Control Programme P. The role of healthcare professionals in treating tobacco dependency: opportunities to reduce CVD for commissioners and practitioners. Cardiovascular Disease Prevention Conference 2018: Getting serious about prevention: reducing variation and optimizing care London, UK2018.
45. Gulliford MC, Khoshaba B, McDermott L, Cornelius V, Ashworth M, Fuller F, et al. Cardiovascular risk at health checks performed opportunistically or following an invitation letter. Cohort study. J Public Health (Oxf). 2018;40(2):e151-e6.
46. Hardman L. The BIG Bolton Health Check. 2014 October 2014. Available from: https://www.healthcheck.nhs.uk/commissioners-and-providers/evidence/case-studies/. Accessed
47. Haringay Council. Scrutiny Review Men's Health: Getting to the Heart of the Matter. A review by the Overview and Scrutiny Committee. 2012. Available from: https://www.healthcheck.nhs.uk/commissioners-and-providers/evidence/local-evaluation/. Accessed

For peer review only - http://bmjopen.bmj.com/site/about/guidelines.xhtml

48. Hawking M. The CVD Risk Report, an intervention to support CVD risk communication in NHS Health Checks: results from the pilot trial. Cardiovascular Disease Prevention Conference 2018: Getting serious about prevention: reducing variation and optimizing care London, UK: NHS England; 2018.
49. Hawking MKD, Timmis A, Wilkins F, Potter JL, Robson J. Improving cardiovascular disease risk communication in NHS Health Checks: a qualitative study. BMJ Open. 2019;9(8):e026058.
50. Hinde S, Bojke L, Richardson G, Retat L, Webber L. The cost-effectiveness of population Health Checks: have the NHS Health Checks been unfairly maligned? Journal of Public Health: From Theory to Practice. 2017;25(4):425-31.
51. Homer K, Boomla K, Hull S, Dostal I, Mathur R, Robson J. Statin prescribing for primary prevention of cardiovascular disease: a cross-sectional, observational study. Br J Gen Pract. 2015(e538).
52. Honey S, Bryant LD, Murray J, Hill K, House A. Differences in the perceived role of the healthcare provider in delivering vascular health checks: a Q methodology study. BMC Fam Pract. 2013;14:172.
53. Honey S, Hill K, Murray J, Craigs C, House A. Patients' responses to the communication of vascular risk in primary care: a qualitative study. Prim Health Care Res Dev. 2015;16(1):61-70.
54. Hooper J, Chohan P, Caley M. Case detection of disease by NHS Health Checks in Warwickshire, England and comparison with predicted performance. Public Health. 2014;128(5):475-7.
55. Hyseni L, Guzman-Castillo M, Kypridemos C, Collins B, Schwaller E, Capewell S, et al. Engaging with stakeholders to inform the development of a decision-support tool for the NHS health check programme: qualitative study. BMC Health Serv Res. 2020;20(1):394.
56. Ismail H, Kelly S. Lessons learned from England's Health Checks Programme: using qualitative research to identify and share best practice. BMC Fam Pract. 2015;16:144.
57. Ismail H, Atkin K. The NHS Health Check programme: insights from a qualitative study of patients. Health Expect. 2016;19(2):345-55.
58. Ismail K, Stahl D, Bayley A, Twist K, Stewart K, Ridge K, et al. Enhanced motivational interviewing for reducing weight and increasing physical activity in adults with high cardiovascular risk: the MOVE IT three-arm RCT. Health Technol Assess. 2019;23(69).
59. Jones T. Telford Healthy Hearts. Cardiovascular Disease Prevention Conference 2020: proactive, predictive, personalised Kia Oval, London2020.
60. Kearney M. Engaging with GPs to help deliver the NHS Health Checks. NHS Health Check 2015 - Improvement through collaboration 2015.
61. Kennedy O, Su F, Pears R, Walmsley E, Roderick P. Evaluating the effectiveness of the NHS Health Check programme in South England: a quasi-randomised controlled trial. BMJ Open. 2019;9(9):e029420.
62. Kirkpatrick W, Eden A. Developing a resilient community & primary care based workforce to deliver the NHS Health Check. NHS Health Check Conference: Getting Serious about Prevention London, UK: NHS England; 2016.
63. Krska J, Mackridge AJ, Taylor J. An Evaluation of the Cardiovascular Screening Service provided by community pharmacies in Sefton PCT: Final Report. Liverpool John Moores University; 2010. Available from: https://www.healthcheck.nhs.uk/commissioners-and-providers/evidence/local-evaluation/. Accessed
64. Krska J, Taylor J, Du Plessis R. Views and experiences of the NHS Health Check provided in pharmacies and medical practices. International Journal of Pharmacy Practice Health Services Research and Pharmacy Practice Conference 2013. Preston United Kingdom.

(var.pagings). 21: Pharmaceutical Press; 2013. p. 16.
65. Krska J, du Plessis R, Chellaswamy H. Views and experiences of the NHS Health Check provided by general medical practices: cross-sectional survey in high-risk patients. J Public Health (Oxf). 2014;37(2):210-7.
66. Krska J, du Plessis R, Chellaswamy H. Views of practice managers and general practitioners on implementing NHS Health Checks. Prim Health Care Res Dev. 2016;17(2):198-205.
67. Krska J, du Plessis R, Chellaswamy H. Implementation of NHS Health Checks in general practice: variation in delivery between practices and practitioners. Prim Health Care Res Dev. 2016;17(4):385-92.
68. Kumar J. GP Engagement "If they know you, it's harder for them to say no". NHS Health Check 2015 - Improvement through collaboration 2015.
69. Lake G. The Healthy Heart Centre: NHS Camden's approach to reducing Health Inequalities. NHS Camden; 2010. Available from: https://www.healthcheck.nhs.uk/commissioners-and-providers/evidence/local-evaluation/. Accessed
70. Lambert MF. Assessing potential local routine monitoring indicators of reach for the NHS health checks programme. Public Health. 2016;131:92-8.
71. Lennon S, London Borough of Redbridge. Supporting LTC Prevention During the COVID-19 Pandemic: Improving NHS Health Checks and Social Prescribing Initiatives (Presentation). 2020.
72. Liverpool City Council. LiveWire Liverpool Health Trainers. Cardiovascular Disease Prevention Conference 2020: proactive, predictive, personalised Kia Oval, London2020.
73. Local Government Association. Public health transformation twenty months on: adding value to tackle local health needs.; 2015. Accessed
74. Local Authority A. The [Local Authority Borough] NHS Health Checks Programme 2019 Review [Unpublished; identifying information redacted]. 2019. Accessed
75. Local Authority B. NHS Health Checks in [Local Authority Borough] [Unpublished; identifying information redacted]. 2019. Accessed
76. Local Authority C. Health Check Quality Assurance - Working Document [Unpublished; identifying information redacted]. Accessed
77. Local Authority C. NHS Health Checks [Local area] Performance and Quality Framework 2019 [Unpublished; identifying information redacted]. 2019. Accessed
78. London Borough of Bromley. Re audit of the Prevention of Diabetes through NHS Health Checks 2014-15. Public Health England,; 2018 January 2018. Available from: https://www.healthcheck.nhs.uk/commissioners-and-providers/evidence/case-studies/. Accessed
79. Loo R, Diaper C, Salami O, Kundu M, Lalkia M, Airhiavbere E, et al. The NHS Health Check: the views of community pharmacists. Int J Pharm Pract. 2011;19(13).
80. Lumley B. Tackling high blood pressure: system wide action and the role of the NHS Health Check. NHS Health Check 2015 - Improvement through collaboration 2015.
81. Maddern S. Partnership Working - the role of local political scrutiny in driving forward the NHS Health Check Programme in Wiltshire. Cardiovascular Disease Prevention Conference 2020: proactive, predictive, personalised. Kia Oval, London: NHS England; 2020.
82. Martin H. Delivering NHS health checks to all. British Journal of Healthcare Management. 2011;17(6):250-5.
83. McDermott L. Exploring the Health Check programme in Primary Care. NHS Health Check 2015 - Improvement through collaboration 2015.

For peer review only

84. McMillan B, Fox S, Lyons M, Bourke S, Mistry M, Ruddock A, et al. Using patient and public involvement to improve the research design and funding application for a project aimed at fostering a more collaborative approach to the NHS health check: the CaVIAR project (better Care Via Improved Access to Records). Research Involv Engagem. 2018;4:18.
85. McNaughton RJ, Oswald NT, Shucksmith JS, Heywood PJ, Watson PS. Making a success of providing NHS Health Checks in community pharmacies across the Tees Valley: a qualitative study. BMC Health Serv Res. 2011;11:222.
86. McNaughton RJ, Shucksmith J. Reasons for (non)compliance with intervention following identification of 'high-risk' status in the NHS Health Check programme. J Public Health (Oxf). 2014;37(2):218-25.
87. Mills K, Harte E, Martin A, MacLure C, Griffin SJ, Mant J, et al. Views of commissioners, managers and healthcare professionals on the NHS Health Check programme: a systematic review. BMJ Open. 2017;7(11):e018606.
88. Nahar P, van Marwijk H, Gibson L, Musinguzi G, Anthierens S, Ford E, et al. A protocol paper: community engagement interventions for cardiovascular disease prevention in socially disadvantaged populations in the UK: an implementation research study. Glob Health Res Policy. 2020;5:12.
89. Nasir N. Can alternate providers improve the uptake of Health Checks among high risk populations and subsequent referrals to the local lifestyle services? Getting Serious About Cardiovascular Disease Prevention 2018: Reducing Variation and Optimising Care London, UK2018.
90. Newman P. An Introduction to Health Coaching: Better Conversations, Better Health. Cardiovascular Disease Prevention Conference 2018: Getting serious about prevention: reducing variation and optimizing care London, UK2018.
91. NHS Greenwich. Evaluation of NHS Health Check Plus Community Outreach Programme in Greenwich. 2011. Accessed
92. NHS Midlands and East. A study into increasing uptake of lifestyle changes - Report. 2011. Accessed
93. NICE. Encouraging people to have NHS Health Checks and supporting them to reduce risk factors. Manchester: NICE, 2014; 2014. Available from: http://publications.nice.org.uk/encouraging-people-to-have-nhs-health-checks-and-supporting-them-to-reduce-risk-factors-lgb15. Accessed
94. Nicholas JM, Burgess C, Dodhia H, Miller J, Fuller F, Cajeat E, et al. Variations in the organization and delivery of the 'NHS health check' in primary care. J Public Health (Oxf). 2012;35(1):85-91.
95. O'Flaherty M, Lloyd-Williams F, Capewell S, Boland A, Maden M, Collins B, et al. Modelling tool to support decision-making in the NHS Health Check programme: workshops, systematic review and co-production with users. Health Technol Assess. 2021;25(35).
96. Onyia I. Delivery of the NHS Health Check by health trainers can improve conversion into uptake of lifestyle service. NHS Health Check Conference: Getting Serious about Prevention London, UK: NHS England; 2016.
97. Oswald N, McNaughton R, Watson P, Shucksmith J. Tees Vascular Assessment Programme: Evaluation commissioned by the Tees Primary Care Trusts (PCT) from the Centre for Translational Research in Public Health. 2010. Available from: https://www.healthcheck.nhs.uk/commissioners-and-providers/evidence/local-evaluation/. Accessed
98. Palladino R, Vamos EP, Chang KC, Khunti K, Majeed A, Millett C. Evaluation of the Diabetes Screening Component of a National Cardiovascular Risk Assessment Programme in England: a Retrospective Cohort Study. Sci Rep. 2020;10(1):1231.
99. Patel R, Barnard S, Thompson K, Lagord C, Clegg E, Worrall R, et al. Evaluation of the uptake and delivery of the NHS Health Check programme in England, using primary care data from 9.5 million people: a cross-sectional study. BMJ Open. 2020;10(11):e042963.
100. Paxton B, Mills K, Usher-Smith JA. Fidelity of the delivery of NHS Health Checks in general practice: an observational study. BJGP Open. 2020;4(4).

For peer review only

101. Perkins C. Cardiovascular disease prevention in the 2020s. Cardiovascular Disease Prevention Conference 2020: proactive, predictive, personalised Kia Oval, London2020.
102. Perry C, Thurston M, Alford S, Cushing J, Panter L. The NHS health check programme in England: a qualitative study. Health Promot Int. 2014;31(1):106-15.
103. Public Health England. NHS Health Check Best practice guidance For commissioners and providers. 2020. Available from: https://www.healthcheck.nhs.uk/commissioners-and-providers/national-guidance/. Accessed 31st October 2019.
104. Rawlinson G. Health promotion in physiotherapy services using NHS health and diabetes checks. British Journal of Healthcare Management. 2019;25(1):22-31.
105. Research Works, Public Health England. Understanding the implementation of NHS Health Checks. 2013. Accessed
106. Richardson J. The fall in NHS Health Checks. Nursing in Practice: The Journal for Today's Primary Care Nurse. 2016(91):1-3.
107. Riley R, Coghill N, Montgomery A, Feder G, Horwood J. The provision of NHS health checks in a community setting: an ethnographic account. BMC Health Serv Res. 2015;15:546.
108. Riley R, Coghill N, Montgomery A, Feder G, Horwood J. Experiences of patients and healthcare professionals of NHS cardiovascular health checks: a qualitative study. J Public Health (Oxf). 2016;38(3):543-51.
109. Robson J, Dostal I, Madurasinghe V, Sheikh A, Hull S, Boomla K, et al. The NHS Health Check programme: implementation in east London 2009-2011. BMJ Open. 2015;5(4):e007578.
110. Robson J, Dostal I, Madurasinghe V, Sheikh A, Hull S, Boomla K, et al. NHS Health Check comorbidity and management: an observational matched study in primary care. Br J Gen Pract. 2017;67(655):e86-e93.
111. Saramunee K, Krska J, Mackridge A, Richards J, Suttajit S, Phillips-Howard P. General public's views on pharmacy public health services: current situation and opportunities in the future. Public health. 2015;129(6):705-15.
112. Shaw RL, Pattison HM, Holland C, Cooke R. Be SMART: examining the experience of implementing the NHS Health Check in UK primary care. BMC Fam Pract. 2015;16:1.
113. Shaw RL, Lowe H, Holland C, Pattison H, Cooke R. GPs' perspectives on managing the NHS Health Check in primary care: a qualitative evaluation of implementation in one area of England. BMJ Open. 2016;6(7):e010951.
114. Simon C. Physical activity for prevention and management of health conditions and practitioner resources. Cardiovascular Disease Prevention Conference 2020: proactive, predictive, personalised Kia Oval, London: NHS England,; 2020.
115. Solutions Strategy Research Facilitation Ltd, Alzheimer's Society. NHS Health Check 40-64 dementia pilot research findings: summary research report. 2017.
116. Strutt E. Patient-centred care: Patients' experiences of and responses to the National Health Service (NHS) Health Check programme in general practice. Durham: Durham University; 2011.
117. Tanner L, Kenny R, Still M, Pearson F, Bhardwaj-Gosling R. NHS Health Check Programme Rapid Review Update. University of Sunderland, Newcastle University, Public Health England; 2020. Available from: https://www.healthcheck.nhs.uk/commissioners-and-providers/evidence/. Accessed 31st October 2020.

For peer review only

118. Thompson K. NHS Health Check StARS framework: A Systems Approach for Raising Standards. NHS Health Check Conference: Getting Serious about Prevention London, UK: NHS England; 2016.
119. Thompson S. Integrated Cardiac Disease Prevention Programme. Cardiovascular Disease Prevention Conference 2019: Saving Hearts and Minds Together. Manchester, UK2019.
120. Trueland J. 'The signs were there, but weren't picked up'. Health Serv J. 2013;123(6369):6-7.
121. Turner A. Exploratory evaluation of variation in statin take up among high risk patients in Nottingham City. Public Health. 2013.
122. Usher-Smith JA, Mant J, Martin A, Harte E, MacLure C, Meads C, et al. NHS Health Check Programme rapid evidence synthesis. Cambridge, UK: The Primary Care Unit, University of Cambridge, RAND Europe, Public Health England; 2017. Accessed 31st October 2020.
123. Visram S, Geddes L, Carr SM. Formative Evaluation of the Health Trainer Community Health Check Service in County Durham. Northumbria University, County Durham and Darlington NHS Foundation Trust; 2012. Available from: https://www.healthcheck.nhs.uk/commissioners-and-providers/evidence/local-evaluation/. Accessed
124. Williams Z. Let's talk about Physical Activity: Physical Activity Clinical Champions training and using the GPPAQ in a health check setting. Cardiovascular Disease Prevention Conference 2018: Getting serious about prevention: reducing variation and optimizing care London, UK2018.

For peer review only

Supplementary File 4

This file contains 3 tables outlining the full set of CMOCs (n=86) developed during the review. The three tables split the CMOCs up into those centred on commissioner (Table S1), provider (Table S2) and attendee (Table S3) perspectives.

Table S1 Overview of CMOCs focused on LA commissioners

	**CMOC**	**Summary of data**
Understanding and engagement with the NHSHC programme: case finding or enabling behaviour change?		
CMOC C1	When commissioners view the NHSHC as a means to improve people's lives through behaviour change (C) they will try to exert their influence over providers to ensure the programme is delivered with this in mind (O) because they believe this will maximise the potential benefits of the programme (M)	Data extracted from 12 documents:<br><br>2 research articles (one cohort study, one qualitative interview study); 4 local evaluation reports; 3 conference materials; 1 unpublished LA working document; 2 other reports focused on LA roles in NHSHCs.
CMOC C2	When commissioners view the NHSHC as a means to improve people's lives through behaviour change (C) they will try to establish closer working relationships between different parts of the wider system (the NHSHC, lifestyle services and primary care) to improve referral pathways (O) because they believe this will maximise the potential benefits of the programme (M)	Data extracted from 8 documents:<br><br>1 research article (survey); 4 local evaluation reports; 2 conference materials; 1 unpublished LA working document.

For peer review only

CMOC C3	When commissioners view the NHSHC as a means to improve people's lives through behaviour change (C) they may develop 'integrated' models of service delivery with the NHSHC delivered alongside lifestyle services (O) because they believe this will maximise the potential benefits of the programme (M)3,	Data extracted from 5 documents:<br><br>1 local evaluation report; 4 conference materials.
CMOC C4	When providers are sceptical and less engaged with the NHSHC programme (C) commissioners may be unable to exert their influence over them (O1) or establish close working relationships between different parts of the system (O2) because providers are resistant and unwilling to engage (M)	Data extracted from 3 documents:<br><br>1 local evaluation report; 1 conference presentation; 1 unpublished LA working document.
CMOC C5	When commissioners have a focus on the wider social determinants of health (C) they are more likely to commission 'alternative' NHSHC providers (i.e. to move away from a medical model based in primary care) (O) because they believe this will maximise the benefits of the programme (M)	Data extracted from 3 documents:<br><br>1 research article (qualitative interviews/workshop); 1 local evaluation report; 1 report focused on LA roles in NHSHCs.
CMOC C6	When commissioners have a focus on the wider social determinants of health (C) they may integrate NHSHC delivery with other services that address other problems (O) because they believe this will maximise the potential benefits of the programme (M)	Data extracted from 3 documents:<br><br>1 research article (mixed methods evaluation); 2 conference materials.

For peer review only

CMOC C7	When commissioners view the NHSHC as a means to improve people's lives through behaviour change (C) they are more likely to collect data related to what happens after the measurements and risk assessment are completed in a check (O) because they believe these are important data to monitor and evaluate programme performance (M)	Data extracted from 15 documents:<br><br>2 research articles (1 qualitative interviews/workshop, 1 qualitative interview study); 1 research report (mixed methods study); 7 local evaluation reports; 4 conference materials; 1 unpublished LA working document.
CMOC C8	When commissioners view the NHSHC as a means to improve people's lives through behaviour change (C) they are more likely to collect data related to the needs of the local population to inform the commissioning of lifestyle support services (O) because they believe this will maximise the benefits of the programme (M)	Data extracted from 6 documents:<br><br>1 PhD thesis (ethnography); 1 local evaluation report; 2 conference materials; 1 report focused on LA roles in NHSHCs; 1 news article.
CMOC C9	In some circumstances, commissioners may cede more control over delivery to primary care based providers (e.g. GP practices) (O) but the contexts in which this happens and the mechanisms underpinning this outcome are not clear (C, M not defined)	Data extracted from 8 documents:<br><br>3 research articles (1 qualitative interview study, 1 survey, 1 cross-sectional study); 4 local evaluation reports; 1 conference materials.
CMOC C10	In some circumstances, commissioners may focus only on mandatory data collection (monitoring invitation, uptake and coverage) (O) but the contexts in which this happen and the mechanisms underpinning this outcome are not clear (C, M not defined)	Data extracted from 5 documents:<br><br>3 research articles (3 cross-sectional studies); 2 local evaluation reports.
Practical constraints: politics and funding		

For peer review only

CMOC C11	Where there is local political support and engagement with the NHSHC programme (C), local delivery is more likely to be evaluated (O1) and developed or improved (O2) because commissioners and public health teams are empowered to focus on the programme (M)	Data extracted from 10 documents:<br><br>1 research article (qualitative interview study); 3 local evaluation reports; 3 conference materials; 2 other reports focused on LA roles in NHSHCs; 1 guidance document
CMOC C12	When funding for public health programmes is constrained (C1) and commissioners or public health teams are convinced of the NHSHCs long term effectiveness and value (C2) local delivery is more likely to be evaluated (O1) and developed or improved (O2) because these activities are considered worthwhile (M)	Data extracted from 5 documents:<br><br>2 research articles (1 qualitative interview study; 1 economic evaluation); 2 local evaluation reports; 1 other report focused on LA roles in NHSHCs.
CMOC C13	When funding for public health programmes is constrained (C) commissioners may select providers who offer the best value for money / lowest cost per NHSHC delivered (O) because they must prioritise mandatory public health programme delivery ('prescribed functions') before funding additional services (M)	Data extracted from 9 documents:<br><br>4 research articles (2 qualitative interview studies; 1 cross-sectional, 1 economic evaluation); 2 local evaluation reports; 1 PhD thesis (ethnography); 1 other report focused on LA roles in NHSHCs; 1 practitioner-facing article
CMOC C14	When funding for public health programmes is constrained (C) commissioners must prioritise funding mandatory services ('prescribed functions') (M) leading to cuts and reduced capacity in non-mandatory services (e.g. local lifestyle support services) (O)	Data extracted from 11 documents:<br><br>8 research articles (5 qualitative interview studies, 1 survey, 1 cross-sectional study, 1 systematic review); 1 local evaluation report; 1 PhD thesis (ethnography); 1 conference presentation.

For peer review only

Table S2 Overview of CMOCs focused on NHSHC providers

	CMOC	Summary of data
Understanding and engagement with the NHSHC programme: scepticism versus 'buy in'		
CMOC P1	When providers are sceptical about the NHSHC programme as a behaviour change intervention (C) they may prioritise completing the mandatory elements of the check and fail to engage with the delivery of advice, brief interventions or referrals (O) because they do not believe these will help attendees (M)	Data extracted from 13 documents:<br><br>3 research articles (2 qualitative interview studies, 1 survey); 3 local evaluation reports; 4 conference materials; 1 other report focused on LA roles in NHSHCs; 1 PhD thesis (ethnography).
CMOC P2	When providers are sceptical about the NHSHC programme as a behaviour change intervention (C1) or sceptical about the effectiveness of behaviour change to reduce the risk of cardiovascular disease (C2) they are more likely to consider medication (e.g. statins or antihypertensives) as an appropriate intervention for those assessed at higher risk (O) because they believe these will help attendees (M)	Data extracted from 11 documents:<br><br>7 research articles (2 qualitative interview studies; 1 survey; 2 cross-sectional studies; 1 cohort study; 1 Q-methodology study); 2 local evaluation reports; 1 research report (systematic review); 1 PhD thesis (ethnography).
CMOC P3	When providers (who are able to prescribe) are sceptical about the recommended thresholds for prescription (C) they are less likely to prescribe (O) because they do not believe it will help attendees (M).<br><br>*This CMOC may apply to other interventions but we lack data to confirm or refute this.*	Data extracted from 4 documents:<br><br>3 research articles (3 cross-sectional studies); 1 conference presentation.

For peer review only

CMOC P4	When providers are worried about labelling healthy people as sick (C) they may avoid offering advice, referrals or prescriptions (O) because they are concerned about the potential harms of overdiagnosis (M)	Data extracted from 3 documents:<br><br>2 research articles (2 qualitative interview studies); 1 PhD thesis (ethnography).
CMOC P5	When providers are highly engaged with preventive health care (C) they are more likely to be highly engaged with the NHSHC programme (O) as they understand it to be a useful means of reaching more patients with this agenda (M)	Data extracted from 2 documents:<br><br>2 research articles (1 qualitative interview study, 1 cohort).
CMOC P6	When providers believe that lifestyle modification is an effective means of reducing CVD risk (C) they are more likely to offer attendees advice, brief interventions or referrals to lifestyle support services (especially as a first line of action) (O) because they believe these may help attendees (M)	Data extracted from 11 documents:<br><br>6 research articles (2 qualitative interview studies; 1 cohort study; 1 mixed methods study; 1 Q-methodology study); 1 research report (RCT); 1 local evaluation report; 3 conference materials
CMOC P7	When providers buy in to the NHSHC as an opportunity to support behaviour change (C) they are more likely to offer attendees advice, brief interventions or referrals to lifestyle support services (O) because they believe these will help attendees (M)	Data extracted from 11 documents:<br><br>5 research articles (1 qualitative interview study; 1 survey; 1 mixed methods study; 1 Q-methodology study); 1 research report (RCT); 4 local evaluation reports; 1 conference presentation
CMOC P8	When providers have the specific skills they need to support the delivery of advice, brief interventions and referrals (C) they are more likely to engage	Data extracted from 10 documents:

For peer review only

	with and prioritise these activities (O) because they feel confident to deliver them during the NHSHC encounter (M)	3 research articles (2 qualitative interview studies, 1 mixed methods study); 1 research report (systematic review); 4 conference materials.
Practical constraints: time and money		
CMOC P9	When funding arrangements for delivery of checks incentivise volume of delivery (C) providers may prioritise completing mandatory elements of the check and minimise time spent delivering advice, brief interventions or offering referrals (O) because they are aware they do not have to complete these (M)	Data extracted from 6 documents:<br><br>3 research articles (1 qualitative interview study, 1 retrospective cohort, 1 cross-sectional); 1 local evaluation report; 1 PhD thesis (ethnography); 1 LA internal working document.
CMOC P10	When providers have many competing priorities (C) they may prioritise completing mandatory elements of the check and minimise time spent delivering advice, brief interventions or offering referrals (O) because of expediency (M)	Data extracted from 16 documents:<br><br>10 research articles (4 qualitative interview studies, 1 systematic review, 3 surveys, 1 ethnography, 1 observational study based on video recordings); 3 local evaluation reports; 1 research report (mixed methods); 1 PhD thesis (ethnography), 1 conference presentation
CMOC P11	When there is a focus on increasing the volume of checks delivered (C) providers may offer more checks 'opportunistically' (i.e. not in a standalone appointment) (O) as they feel this is more efficient (M)	Data extracted from 5 documents:<br><br>4 research articles (2 qualitative interview studies, 1 retrospective cohort, 1 survey, 1 mixed methods); 1 local evaluation report

For peer review only

CMOC P12	It is clear that data about non-mandatory elements of a check are often under-recorded or recorded inconsistently (O) but the contexts in which this happens and the mechanisms underpinning this outcome are not clear (C, M not defined). Potentially important contexts here include competing priorities, a focus on delivery of mandatory elements of checks, lack of monitoring and/or incentivisation to collect particular data items, or difficulty in recording certain activities within existing data collection systems	Data extracted from 15 documents:<br><br>10 research articles (3 qualitative interview studies, 4 cross-sectional studies, 1 pre/post study, 1 retrospective cohort, 1 study assessing the validity of indicators); 3 local evaluation reports; 1 guidance document; 1 conference abstract.
CMOC P13	When providers do not feel they are adequately compensated for delivering checks (C) they may prioritise completing mandatory elements of the check and minimise time spent delivering advice, brief interventions, or offering referrals (O) because they do not feel it is worth the cost (M)	Data extracted from 10 documents:<br><br>6 research articles (2 qualitative interview studies; 1 cross-sectional studies, 1 quasi-RCT, 1 surveys, 1 systematic review); 2 local evaluation reports; 2 conference materials
CMOC P14	When providers 'buy in' to the NHSHC programme (see CMOC P7 above) (C1) and have adequate time and/or flexibility to deliver each check (C2) they may offer more personalised and in-depth advice and support (O) because they believe these may help attendees (M)	Data extracted from 9 documents:<br><br>2 research articles (1 qualitative interview study, 1 mixed methods); 5 local evaluation reports; 1 conference presentation; 1 practitioner-facing article
Practical constraints: referrals and follow-up		
CMOC P15	When multiple modifiable risk factors are identified during a check (C1) and separate lifestyle services exist for each (C2) providers (and attendees)	Data extracted from 4 documents:

For peer review only

	may agree to prioritise addressing one risk factor first (M) so the delivery of advice, brief interventions and referrals reflect this priority (O)	1 research article (qualitative interview study); 2 local evaluation reports, 1 conference presentation
CMOC P16	When providers don't perceive available lifestyle services to be a good 'fit' for individuals (C) they may avoid making referrals to these services (O) because they do not believe it will help attendees (M)	Data extracted from 4 documents:<br><br>2 research articles (1 retrospective cohort, 1 cross-sectional study); 2 local evaluation reports; 1 PhD thesis (interviews/ethnography)
CMOC P17	When information about local lifestyle services and referral routes is disparate and difficult to access (C) it is harder for providers to make referrals (O) because providers are unaware of available services and how to refer (M)	Data extracted from 9 documents:<br><br>2 research articles (1 qualitative interview study, 1 mixed methods); 4 local evaluation reports; 3 conference materials
CMOC P18	When providers have concerns about the quality of lifestyle support services (C) they may avoid making referrals (O) because they doubt they will help attendees (M)	Data extracted from 3 documents:<br><br>2 research articles (1 survey, 1 Q-methodology study); 1 news article
CMOC P19	When providers have established relationships and referral pathways to trusted lifestyle services (C) they are more likely to offer referrals (O) because this becomes a delivery norm (M)	Data extracted from 12 documents:<br><br>1 research article (mixed methods); 5 local evaluation reports; 6 conference materials
Practical constraints: relationships and confidence in the delivery of advice		

For peer review only

CMOC P20	When providers are concerned that discussion of a particular risk factor may cause offence or upset an attendee (C) they may avoid bringing it up or discussing it in-depth (O) because they lack confidence and want to avoid confrontation (M)	Data extracted from 11 documents:<br><br>6 research articles (3 qualitative interview studies, 1 survey, 1 observational study based on video recordings, 1 fidelity assessment); 2 local evaluation reports; 1 research report (observational study); 1 PhD thesis (ethnography); 1 other report
CMOC P21	When providers lack knowledge about recommendations in relation to a particular risk factor (C) they may avoid bringing it up or discussing it in-depth during a check (O) because they lack confidence in their advice (M)	Data extracted from 4 documents:<br><br>3 research articles (1 qualitative interview study; 1 survey; 1 fidelity assessment); one conference presentation
CMOC P22	When providers perceive that an attendee is unlikely to want to, or be able to change their lifestyle (C) they may avoid giving them advice or offering referrals to support this (O) because they do not think it will help (M1) or because they are worried it could damage their relationship (M2)	Data extracted from 4 documents:<br><br>3 research articles (2 qualitative interview studies; 1 Q-methodology study); 1 local evaluation report
CMOC P23	When providers feel they themselves are not good role models for healthy lifestyles (C) they may be reluctant to deliver advice or brief interventions, or make referrals (O) because they are worried about appearing hypocritical and lacking credibility (M)	Data extracted from 2 documents:<br><br>1 research article (qualitative interview study) and 1 conference presentation

For peer review only

CMOC P24	When providers have lived experience of (trying to) make lifestyle changes (C) they may share this and empathise with attendees (O) because they want to build rapport and a therapeutic alliance during checks (M)	Data extracted from 5 documents:<br><br>3 research articles (2 qualitative interview studies, 1 Q-methodology study); 1 local evaluation report; 1 PhD thesis (ethnography)
CMOC P25	When discussing a risk factor is normalised and routine (C) providers may be more likely to deliver advice, brief interventions and offer referrals related to that risk factor (O) because they feel comfortable and practiced in doing so (M)	Data extracted from 6 documents:<br><br>1 research article (survey); 2 evaluation reports; 3 conference materials
CMOC P26	When providers receive training (C1) or have regular practice (C2) in delivering lifestyle advice, they are more likely to deliver it regularly during checks (O) because they feel more confident (M)	Data extracted from 9 documents:<br><br>3 research articles (1 qualitative interview study, 1 mixed methods, 1 RCT); 1 local evaluation reports; 5 conference materials
CMOC P27	When providers take into account attendees' own priorities, constraints and wishes during a check (C) they may adapt the advice, brief interventions or referrals offered to take these into account (e.g. make fewer but more appropriate referrals) (O) because they share the decision with attendees (M)	Data extracted from 12 documents:<br><br>8 research articles (7 qualitative interview studies, 1 Q-methodology study); 1 local evaluation report; 3 conference materials
CMOC P28	When providers are aware of attendees' own priorities, constraints and wishes (C) they may identify and emphasise the benefits of simple changes	Data extracted 7 from documents:

For peer review only

	that are more acceptable and achievable for attendees (O) because they believe this will help attendees (M)	3 research articles (2 qualitative interview studies, 1 pilot RCT); 1 local evaluation report; 3 conference materials

Table S3 Overview of CMOCs focused on NHSHC attendees

	**CMOC**	**Summary of data**
Understanding and engagement with the NHSHC programme: expectations and priorities		
CMOC A1	When attendees understand the NHSHC as a screening opportunity aimed at identifying individuals with disease (C) they may be less likely to engage with advice, brief interventions or offers of referrals (O) because what is offered does not meet their expectations (M)	Data extracted from 8 documents:<br><br>5 research articles (4 qualitative interview studies, 3 local evaluation reports)
CMOC A2	When attendees are already aware of risk factors and/or potential improvements they could make to their lifestyle (C) they may be more receptive to receiving advice, brief interventions or referrals (O) because they are mentally prepared for it (M)	Data extracted from 15 documents:<br><br>8 research articles (6 qualitative interview studies, 1 ethnography, 1 mixed methods); 4 local evaluation reports; 1 PhD (ethnography); 2 conference materials
CMOC A3	When attendees are unaware that they have risk factors for CVD and receive results that indicate that they are at high risk (C) they may need extra support and information from providers (O) because they are shocked and upset (M)	Data extracted from 6 documents:<br><br>5 research articles (4 qualitative interview studies, 1 ethnography); 1 local evaluation report

For peer review only

CMOC A4	In some circumstances attendees who receive results that indicate that they are at high risk may be prompted to make immediate lifestyle changes (O) because they are shocked and upset (M), but the contexts in which this happens (and for whom) are not clear (C undefined)	Data extracted from 8 documents:<br><br>3 research articles (3 qualitative interview studies); 4 local evaluation reports; 1 conference presentation
CMOC A5	When providers are able to explain the implications of risk factors to attendees in a way they can understand (C) attendees may be more receptive to advice, brief interventions or referrals (O) because they appreciate its importance for their own lives (M)	Data extracted from 6 documents:<br><br>1 research article (qualitative interview study); 1 local evaluation report; 1 PhD thesis (interviews/ethnography); 3 conference materials
CMOC A6	When providers are able to link advice, brief interventions and offers of referrals to attendees' own priorities for their health and lifestyle (C) attendees may be more likely to engage with these (O) because they want to achieve these (M)	Data extracted from 10 documents:<br><br>6 research articles (4 qualitative interview studies, 1 co-production study, 1 observational study using video-recordings); 1 research report (RCT); 1 local evaluation report; 1 PhD thesis (interviews/ethnography); 1 conference poster
CMOC A7	When attendees have multiple risk factors (C) they may choose to focus on the advice, brief interventions or referrals offered in relation to those lifestyle changes that are easier to change (O) because they feel it is better than nothing (M)	Data extracted from 3 documents:<br><br>2 research articles (qualitative interview studies); 1 PhD thesis (interviews/ethnography)

For peer review only

CMOC A8	When attendees feel a personal responsibility for their own health and lifestyle (C) they may be unlikely to take up offers of referrals or ongoing support or follow up (O) because they feel obliged to try to make changes on their own (M)	Data extracted from 3 documents:<br><br>2 research articles (2 qualitative interview studies); 1 local evaluation report
CMOC A9	When attendees are not motivated to change their lifestyle or behaviour (C) they are unlikely to engage with advice, brief interventions or take up offers of referrals to lifestyle services (O) because they do not believe they need to, and have other priorities (M)	Data extracted from 8 documents:<br><br>6 research articles (4 qualitative interview studies, 1 RCT, 1 cross-sectional study); 1 research report (mixed methods); 1 local evaluation report
CMOC A10	When attendees are fatalistic about their health (C) they are unlikely to engage with advice, brief interventions or take up offers of referrals to lifestyle services (O) because they think they are pointless (M)	Data extracted from 8 documents:<br><br>4 research articles (4 qualitative interview studies); 1 research report (mixed methods); 1 local evaluation report; 1 conference presentation
CMOC A11	When attendees have health concerns and priorities that fall outside the remit of the NHSHC programme (C) they may be disappointed with the check (O1) and unlikely to engage with advice, brief interventions or take up offers of referrals to lifestyle services (O2) because these do not feel important to them, and they have other priorities (M)	Data extracted from 5 documents:<br><br>2 research articles (2 qualitative interview studies); 1 local evaluation report; 2 PhD theses (ethography, interviews/ethnography).

For peer review only

CMOC A12	When attendees receive an 'opportunistic' check (C) they are less likely to (receive and) engage with advice, brief interventions or offers of referrals (O) because they do not understand that this is the purpose of the check (M)	Data extracted from 6 documents:<br><br>3 research articles (3 qualitative interview studies); 2 local evaluation reports; 1 guidance document
CMOC A13	When attendees have the opportunity and time to discuss and ask questions during a check (C) they may be more likely to receive and engage with advice, brief interventions and offers of referrals (O) because they understand that this is the purpose of the check (M)	Data extracted from 14 documents:<br><br>5 research articles (2 qualitative interview studies, 1 ethnography, 1 mixed methods study, 1 observational study using video-recordings); 6 local evaluation reports; 1 research report (mixed methods); 1 PhD thesis (interviews/ethnography)
CMOC A14	When checks are delivered in a non-medical setting (i.e. not in general practice) (C) attendees may be more likely to engage in discussions about risk factors and lifestyle change (O) because they feel relaxed and comfortable (M)	Data extracted from 8 documents:<br><br>3 research articles (1 qualitative interview study, 1 ethnography, 1 protocol for a mixed methods study); 1 local evaluation report; 4 conference materials
Understanding and engagement with the NHSHC programme: credibility and trust		
CMOC A15	When attendees receive advice, brief interventions, offers of referral or prescriptions from a professional they consider to be suitably qualified (C) they may be more likely to engage with or accept these interventions (O) because they consider them to be credible (M)	Data extracted from 10 documents:<br><br>7 research articles (3 qualitative interview studies, 2 surveys, 2 mixed methods studies); 2 local evaluation reports; 1 PhD thesis (interviews/ethnography).

For peer review only

CMOC A16	When attendees receive advice, brief interventions or offers of referral from a provider who seems to understand their circumstances (C) they may be more likely to engage with these interventions (O) because they consider them to be credible (M)	Data extracted from 5 documents:<br><br>1 research article (ethnography); 1 research report (RCT); 1 local evaluation report; 1 conference poster; 1 news article
CMOC A17	When providers can deliver culturally appropriate lifestyle advice (C) attendees may be more likely to engage with it (O) because they consider it to be more credible and relevant (M)	Data extracted from 4 documents:<br><br>1 research article (ethnography); 1 research report (RCT); 1 local evaluation report; 1 conference abstract
CMOC A18	When the measurements and risk assessment completed during a check identify attendees as 'low risk' (C) attendees may be less receptive to any advice, brief interventions or referrals offered (O) because they are reassured and do not think they need to take action (O)	Data extracted from 4 documents:<br><br>3 research articles (1 qualitative interview study, 1 survey, 1 mixed methods study); 1 local evaluation report
CMOC A19	When providers downplay risks or temper advice about lifestyle (C) attendees may feel there is no need to make any changes (O) because they are reassured (M)	Data extracted from 5 documents:<br><br>4 research articles (4 qualitative interview studies); 1 local evaluation report
CMOC A20	When providers are able to convey the importance and urgency of NHSHC risk assessments to attendees (C) attendees may be more receptive to advice, brief interventions or referrals (O) because they feel important (M)	Data extracted from 6 documents:<br><br>3 research articles (3 qualitative interview studies); 1 local evaluation report; 1 PhD thesis (interviews/ethnography); 1 conference presentation

For peer review only

CMOC A21	When the measurements and risk assessment completed during a check identify attendees as 'low risk' (C) attendees' healthy lifestyle habits may be reinforced (O) because they understand these have tangible benefits, reflected in their results (M)	Data extracted from 3 documents:<br><br>2 research articles (1 qualitative interview study, 1 ethnography); 1 PhD thesis (interviews/ethnography)
CMOC A22	When attendees receive measurements and risk assessment results from non-professionals (C1) or do not receive results at all (C2) they may be less likely to consider the results to be important (O) because they trust that providers will alert them to significant results that require action (M)	Data extracted from 4 documents:<br><br>3 research articles (2 qualitative interview studies, 1 survey); 1 PhD thesis (interviews/ethnography)
CMOC A23	When attendees perceive providers to be disengaged with the check (C) they may be less likely to engage with advice, brief interventions or offers of referral (O) because they do not believe these are important (M)	Data extracted from 4 documents:<br><br>2 research articles (1 ethnography, 1 survey); 1 PhD thesis (ethnography); 1 conference presentation
CMOC A24	When attendees receive advice, brief interventions or offers of referral from a provider who does not seem to 'practice what they preach' (C) they may be less likely to engage with these interventions (O) because they consider these to lack credibility (M)	Data extracted from 2 documents:<br><br>1 research article (qualitative interview study); 1 local evaluation report
CMOC A25	When attendees receive advice or information relating to healthy lifestyles that they already familiar with during a check (C) they may perceive the advice to be useless (O) because they have heard it before (M)	Data extracted from 8 documents:<br><br>6 research articles (4 qualitative interview studies, 1 ethnography, 1 mixed methods study); 2 PhD theses (ethnography, interviews/ethnography)

For peer review only

CMOC A26	When attendees learn something new and important to them during a check (C) they may attempt to make changes to their lifestyle in light of this (O) because they have an improved understanding of risk factors or lifestyle advice (M)	Data extracted from 6 documents:<br><br>3 research articles (1 qualitative interview study, 1 ethnography, 1 survey); 1 research report (RCT); 1 local evaluation report; 1 PhD thesis (interviews/ethnography)
CMOC A27	When attendees are aware of conflicting or inconsistent guidance about healthy lifestyles (C) they may reject or ignore advice delivered during a check (O) because they doubt its credibility (M1) or are confused (M2)	Data extracted from 4 documents:<br><br>2 research articles (2 qualitative interview studies); 1 research report (RCT); 1 PhD thesis (ethnography)
CMOC A28	When attendees are aware of controversy in relation to recommended medication (statins) (C) they may be ambivalent about accepting or adhering to a prescription (O) because they are uncertain of the benefits (M)	Data extracted from 5 documents:<br><br>3 research articles (3 qualitative interview studies; 1 local evaluation reports; 1 conference presentation
CMOC A29	When attendees have doubts about the accuracy of the tests and tools used to measure and assess risk during a check (C) they may be less receptive to advice, brief interventions, referrals or prescriptions offered to address identified risks (O) because they are unsure that the assessments are credible (M)	Data extracted from 6 documents:<br><br>2 research articles (1 qualitative interview study, 1 mixed methods study); 1 research report (mixed methods study); 2 local evaluation reports; 1 PhD thesis (interviews/ethnography)
Practical constraints: referrals and follow-up		

For peer review only

CMOC A30	When attendees receive continuity of care and follow up after a check (C) they may be more likely to engage with advice, take up referrals or attempt to make lifestyle changes (O) because they are reminded of what they are meant to be doing and why (M)	Data extracted from 10 documents:<br><br>5 research articles (4 qualitative interview studies, 1 pilot RCT); 1 research report (RCT); 2 local evaluation reports; 1 PhD thesis (interviews/ethnography); 1 conference poster
CMOC A31	When attendees receive continuity of care and follow up after a check (C) they may be more likely to engage with advice, take up referrals or attempt to make lifestyle changes (O) because they feel supported and valued (M)	Data extracted from 5 documents:<br><br>2 research articles (2 qualitative interview studies); 1 research reports (RCT); 1 local evaluation report; 1 PhD thesis (ethnography)
CMOC A32	When attendees are repeatedly offered follow up and feedback on progress after a check (C) they may be motivated to attempt and maintain lifestyle changes (O) because they can monitor their progress (M)	Data extracted from 6 documents:<br><br>3 research articles (3 qualitative interview studies); 1 research report (RCT); 2 local evaluation reports
CMOC A33	When attendees can see tangible benefits of making lifestyle changes after a check (C) they are more likely to maintain these (O) because they are motivated to continue (M)	Data extracted from 5 documents:<br><br>1 research article (qualitative interview study); 2 local evaluation reports; 2 conference materials

For peer review only

CMOC A34	When attendees are not offered any follow up (beyond the five-year NHSHC programme cycle) (C) they may lack motivation to attempt any lifestyle changes (O) because they interpret the absence of follow up to mean there is no urgent need to make changes (M)	Data extracted from 3 documents:<br><br>1 research article (survey); 1 research report (RCT); 1 PhD thesis (interviews/ethnography)
CMOC A35	When attendees don't consider local lifestyle services to be convenient, appropriate or likely to meet their needs (C) they are less likely to take up referrals or attend these services (O) because they feel it is pointless (M)	Data extracted from 14 documents:<br><br>9 research articles (7 qualitative interviews studies, 1 systematic review, 1 mixed methods study); 2 local evaluation reports; 2 PhD theses (ethnography, interviews/ethnography); 1 conference abstract
CMOC A36	When local lifestyle support services are designed to be more accessible (e.g. in terms of timing, location, cost) (C) attendees may be more likely to start and continue to attend (O) because they feel they are more convenient, affordable or relevant (M)	Data extracted from 4 documents:<br><br>3 research articles (3 qualitative interview studies); 1 conference poster
CMOC A37	When attendees have the option to try out a lifestyle service or are supported to try one by a provider (C) they may be more likely to take up an offer of a referral (O) because they feel more confident to do so (M)	Data extracted from 3 documents:<br><br>1 research article (pilot RCT); 1 local evaluation report; 1 conference abstract
Practical constraints for attendees: person-centredness		

For peer review only

CMOC A38	When attendees receive advice about healthy lifestyles that does not take account of their personal circumstances (C) they are less likely to engage with it (O) because they believe it is unworkable for them (M)	Data extracted from 10 documents:<br><br>8 research articles (7 qualitative interview studies, 1 mixed methods study); 1 PhD thesis (ethnography); 1 conference presentation
CMOC A39	When attendees receive advice about healthy lifestyles that they believe they cannot achieve (e.g. because it seems to require big changes) (C) they are less likely to engage with it (O) because they feel overwhelmed and hopeless (M)	Data extracted from 3 documents:<br><br>3 research articles (2 qualitative interview studies, 1 observational study using video recordings)
CMOC A40	When attendees receive advice about healthy lifestyles that attendees feel they can fit into their lives (e.g. around other commitments) (C) they may be more likely to engage with it (O) because they perceive the changes to be less disruptive (M)	Data extracted from 13 documents:<br><br>6 research articles (4 qualitative interview studies, 1 ethnography, 1 pilot RCT); 1 research report (RCT); 5 local evaluation reports; 1 conference poster
CMOC A41	When attendees feel that significant lifestyle change is unworkable for them (C) they may be more likely to accept prescriptions (e.g. for statins) (O) because they still want to do something to reduce their CVD risk (M)	Data extracted from 2 documents:<br><br>1 research article (qualitative interview study); 1 local evaluation report
CMOC A42	When attendees anticipate or experience medication side effects or burdens (C) they may be more ambivalent about accepting or adhering to prescriptions (O) because they are uncertain of the benefits and concerned about harms (M)	Data extracted from 4 documents:<br><br>2 research articles (2 qualitative interview studies); 1 local evaluation report; 1 conference presentation

For peer review only

CMOC A43	When attendees anticipate or experience medication side effects or burdens (C) they attempt lifestyle change (O) because they prefer this option (M)	Data extracted from 3 documents:<br><br>2 research articles (2 qualitative interview studies); 1 local evaluation report
CMOC A44	When attendees are encouraged and supported by friends, family or peers to make and sustain lifestyle changes (C) they may be more likely to attempt and maintain changes (O) but the mechanism for this is unclear (M not defined)	Data extracted from 11 documents:<br><br>7 research articles (5 qualitative interview studies, 1 ethnography, 1 co-production study); 1 research report (RCT); 1 local evaluation report; 2 PhD theses (ethnography, interviews/ethnography)

For peer review only

BMJ Open

Understanding what happens to attendees after an NHS Health Check: a realist review

Journal:	*BMJ Open*
Manuscript ID	bmjopen-2022-064237.R1
Article Type:	Original research
Date Submitted by the Author:	17-Aug-2022
Complete List of Authors:	Duddy, Claire; University of Oxford, Nuffield Department of Primary Care Health Sciences<br>Gadsby, Erica; University of Stirling, Faculty of Health Sciences and Sport<br>Hibberd, Vivienne; University of Greenwich Medway School of Pharmacy, Public Involvement in Pharmacy Studies Group; University of Kent Medway School of Pharmacy<br>Krska, Janet; University of Greenwich Medway School of Pharmacy; University of Kent Medway School of Pharmacy<br>Wong, Geoff; Oxford University, Nuffield Department of Primary Care Health Sciences
**Primary Subject Heading**:	Public health
Secondary Subject Heading:	Cardiovascular medicine, General practice / Family practice, Health policy
Keywords:	PUBLIC HEALTH, CARDIOLOGY, Health policy < HEALTH SERVICES ADMINISTRATION & MANAGEMENT, Risk management < HEALTH SERVICES ADMINISTRATION & MANAGEMENT, PREVENTIVE MEDICINE

SCHOLARONE™
Manuscripts

I, the Submitting Author has the right to grant and does grant on behalf of all authors of the Work (as defined in the below author licence), an exclusive licence and/or a non-exclusive licence for contributions from authors who are: i) UK Crown employees; ii) where BMJ has agreed a CC-BY licence shall apply, and/or iii) in accordance with the terms applicable for US Federal Government officers or employees acting as part of their official duties; on a worldwide, perpetual, irrevocable, royalty-free basis to BMJ Publishing Group Ltd ("BMJ") its licensees and where the relevant Journal is co-owned by BMJ to the co-owners of the Journal, to publish the Work in this journal and any other BMJ products and to exploit all rights, as set out in our licence.

The Submitting Author accepts and understands that any supply made under these terms is made by BMJ to the Submitting Author unless you are acting as an employee on behalf of your employer or a postgraduate student of an affiliated institution which is paying any applicable article publishing charge ("APC") for Open Access articles. Where the Submitting Author wishes to make the Work available on an Open Access basis (and intends to pay the relevant APC), the terms of reuse of such Open Access shall be governed by a Creative Commons licence – details of these licences and which Creative Commons licence will apply to this Work are set out in our licence referred to above.

Other than as permitted in any relevant BMJ Author's Self Archiving Policies, I confirm this Work has not been accepted for publication elsewhere, is not being considered for publication elsewhere and does not duplicate material already published. I confirm all authors consent to publication of this Work and authorise the granting of this licence.

For peer review only

Understanding what happens to attendees after an NHS Health Check: a realist review

Claire Duddy[1*], Erica Gadsby[2], Vivienne Hibberd[3], Janet Krska[4], Geoff Wong[1]

[1] Nuffield Department of Primary Care Health Sciences, University of Oxford, Oxford, UK

[2] Faculty of Health Sciences and Sport, University of Stirling, Stirling, UK and Centre for Health Services Studies, University of Kent, Canterbury, UK.

[3] Public Involvement in Pharmacy Studies Group, Medway School of Pharmacy, Universities of Greenwich and Kent, Chatham Maritime, UK

[4] Medway School of Pharmacy, Universities of Greenwich and Kent, Chatham Maritime, UK.

*Corresponding author

claire.duddy@phc.ox.ac.uk

01865 617855

Radcliffe Primary Care Building

Radcliffe Observatory Quarter

Woodstock Road

Oxford OX2 6GG.

Word count: **6234** (excluding title page, abstract, references, tables, acknowledgements)

For peer review only

Abstract

Objectives

The NHS Health Check offers adults aged 40-74 an assessment of their risk of developing cardiovascular disease. Attendees should be offered appropriate clinical or behavioural interventions to help them to manage or reduce these risks. This project focused on understanding variation in the advice and support offered to Health Check attendees.

Design

We conducted a realist review, assembling a diverse body of literature via database searches (MEDLINE, Embase, CINAHL, HMIC, Web of Science) and other search methods, and synthesised data extracted from documents using a realist logic of analysis. Our aim was to develop an understanding of contexts affecting delivery of the NHS Health Check and the underlying mechanisms producing outcomes related to the offer for attendees post-Check.

Results

Our findings demonstrate differences in how NHS Health Check commissioners, providers and attendees understand the primary purpose of the programme. A focus on screening for disease can produce an emphasis on high-volume delivery in primary care. When delivery models are organised around behavioural approaches to risk reduction, more emphasis is placed on advice, and referrals to 'lifestyle services'. However, constrained funding and competing priorities for providers limit what can be delivered within the programme's remit. Attendees' experiences and responses to the programme are affected by how the programme is delivered, and by the difficulty of incorporating its outputs into their lives.

Conclusions

The remit of the NHS Health Check should be reviewed with consideration of what can be effectively delivered within existing resources. Variation in delivery may be appropriate to meet local needs, but differences in how the programme's primary purpose is understood contributes to a 'postcode lottery' in post-Check advice and support. Our findings underline existing concerns that the programme may

For peer review only

generate inequitable outcomes and raise questions about whether it can deliver positive outcomes for the majority of attendees.

Study registration

PROSPERO CRD42020163822

Keywords

cardiovascular diseases, prevention, public health, lifestyle, health promotion, screening, realist review

Article Summary

Strengths and limitations of this study

- This is the first realist review focused on the NHS Health Check programme and it has generated new understanding of the causes of variation in delivery at the end of the Health Check pathway;
- The review is inclusive of a wide range of literature, including grey literature, allowing us to draw on learning from local delivery models;
- This project was strengthened by strong and consistent PPI (patient and public involvement) and professional stakeholder input, which shaped and tested the relevance of our theories throughout;
- As with any review, our findings were limited by the availability and quality of the available evidence – we sought to fill a gap in the existing literature focused on the end of the Health Check pathway but this necessarily limited the number and quality of documents available.

Background

The NHS Health Check in England is a large-scale public health programme that aims to offer adults aged 40-74 a five-yearly assessment of their risk of developing cardiovascular disease (CVD) (excluding those with a pre-existing CVD-related condition).(1) The NHS Health Check involves the measurement of multiple CVD risk factors and the delivery of advice and discussion of appropriate clinical and behavioural approaches that might help individuals to manage and reduce their CVD risk.

For peer review only - http://bmjopen.bmj.com/site/about/guidelines.xhtml

These could include, for example, referral to a GP to discuss recommended pharmacological options (generally statins or antihypertensives) or the delivery of advice, signposting or formal referral to 'lifestyle' services, such as smoking cessation and weight management programmes.(2)

The programme is concerned with both the early identification of risk factors for CVD and the delivery of early interventions to address them, via both pharmacological and non-pharmacological, 'lifestyle' or 'behavioural' means. It is also tasked with helping to address population-level health disparities, reflecting an understanding that the burden of CVD is not spread equally throughout the population, but is strongly linked to factors including deprivation and ethnicity. The intention to use the programme to address health inequalities has been present since its launch; most recently, the programme is cited in the UK Government's recent 'Levelling Up' white paper as a potential means of delivering its prevention agenda.(3) To meet its aims of preventing CVD at a population level, and contributing to reducing the health inequalities associated with CVD, the NHS Health Check must be widely taken up (especially by those with most to gain), and be effective in both assessing and reducing or managing any risks identified.

The NHS Health Check was first introduced in 2009 but the programme was relaunched in 2013, when responsibility for commissioning the programme transferred from NHS Primary Care Trusts (PCTs) to local authorities (LAs). Minimum standards for programme delivery – focused on the collection of certain measurements and targets to increase the volume of checks delivered - became statutory requirements at this time(4, 5) and Public Health England (PHE) was formed and given oversight of the programme. In 2021, the new Office for Health Improvement and Disparities (OHID) took on responsibility for the programme and published a major review (instigated and conducted by PHE), setting out an updated 'vision' for the NHS Health Check. The review's recommendations include aims to launch a digital service and to increase the size and remit of the programme by extending it to cover younger age groups and to address more conditions beyond CVD.(6)

The NHS Health Check has long been the subject of controversy, with some calling its effectiveness and cost-effectiveness into question,(7-10) and PHE responding by collating evidence that supports the programme under the auspices of the Expert Scientific and Clinical Advisory Panel (ESCAP).(11)

For peer review only - http://bmjopen.bmj.com/site/about/guidelines.xhtml

For peer review only

Observational studies included in two PHE-commissioned rapid reviews suggest that the programme is associated with increased rates of CVD risk factor and disease detection, statin prescribing and referrals to 'lifestyle services' (including smoking cessation, weight management, exercise and alcohol support services). Work undertaken to support the recent PHE/OHID review of the programme has also identified an association between Health Check attendance and improvement in a range of indicators, including: BMI, rates of smoking, blood pressure, total cholesterol, hospital admissions related to CVD or type 2 diabetes, and all-cause death, after five years,(12) although the direction of causality is unclear.

Regional and local studies of the implementation and outcomes of the programme demonstrate wide variation in programme delivery and outcomes across England. Evidence on the delivery of advice, referrals and on behaviour changes post-Health Check is especially sparse. The rapid reviews identified only six primary studies examining behaviour change, all focused only on smoking cessation.(13, 14)

Our scoping searches and an initial review of the existing research evidence relating to the programme identified a clear focus on invitation, uptake and coverage of the NHS Health Check. Far less attention has been paid to what happens after the measurements and risk assessments have been undertaken, especially in relation to the delivery of advice, onward signposting or referral and ongoing support for behaviour changes that might reduce CVD risks. The capacity of the NHS Health Check to provide attendees with appropriate advice, referrals and support – and the extent to which attendees respond to these – is a critical assumption underpinning the effectiveness of the programme.

Current best practice guidance makes recommendations for NHS Health Check commissioners and providers, describing a range of possible advice and referral options that may be made available, as well as clinical interventions that may be appropriate to offer attendees.(2) It is clear that responsibility for clinical follow up rests with primary care, and the guidance suggests that commissioners "may wish to" put referral pathways to onward services, such as smoking cessation services, in place. Patel et al's large-scale observational study of the NHS Health Check for the period 2012-2017 includes data relating to the provision of "advice, information or referral" for

For peer review only - http://bmjopen.bmj.com/site/about/guidelines.xhtml

different risk factors that may be flagged during a check.(15) Although data recording for these activities is likely to be incomplete, the figures presented in this study suggest that there is wide variation in the delivery and recording of these activities for different risk factors, and that rates of delivery fall well below the recommended thresholds for intervention. For example, only one quarter of attendees recorded as meeting the threshold for intervention are recorded as receiving advice, information or referral in relation to diet. In addition, this national-level study obscures the wide regional variation in what they term 'post-delivery management' following a check, identified in existing reviews that include local studies.(13, 14)

In consultation with our stakeholder groups (see Methods below for more details on group membership) we focused our review on these final steps in the NHS Health Check pathway. Our aim was to develop an understanding of how the NHS Health Check programme works in different settings, and for different groups to achieve its outcomes, with a specific focus on what happens after the measurements and risk assessment are complete.

Methods

We conducted a realist review to synthesise evidence that could help us to develop an understanding of the important contexts that influence the delivery of the NHS Health Check and the mechanisms that produce intended and unintended outcomes. Realist review is an interpretive, theory-driven approach, chosen because existing research clearly demonstrates that the NHS Health Check programme is a complex intervention with context-sensitive outcomes. Our realist analysis used data extracted from the literature to develop context-mechanism-outcome configurations (CMOCs). CMOCs are causal explanations that describe why and how (by which mechanisms) particular outcomes are generated in particular contexts. The NHS Health Check programme is characterised by wide variation in commissioning and delivery and hence in the experience of attendees. Our review sought to make sense of this variation, by developing a realist programme theory, based on CMOCs, that elucidates the causes of outcomes related to the final steps in the patient pathway, i.e. on what happens after the measurements and risk assessments are complete.

For peer review only - http://bmjopen.bmj.com/site/about/guidelines.xhtml

For peer review only

Our methods are described in brief below, while full details are available in a detailed protocol paper.(16) The conduct and reporting of the review followed the RAMESES (Realist and Meta-narrative Evidence Syntheses: Evolving Standards) quality(17) and reporting standards.(18) A glossary of realist terminology and RAMESES checklist are included in **Supplementary File 1**.

Patient and public involvement

Our review was informed by the involvement of two stakeholder groups, who provided us with content expertise based on their lived experience of commissioning, providing or receiving (or being eligible to receive) the NHS Health Check. Our patient and public involvement (PPI) group comprised 10 members of the public from 6 English regions and were selected to be as diverse as possible in relation to age, gender, ethnicity and geography. Our 'professional' stakeholder group were recruited via our existing networks and snowballing, and included policy makers, LA commissioners, NHS Health Check providers and a trainer, and representatives from relevant health charities. Our final meeting with the latter group was expanded to include 36 individuals, to ensure a broad range of feedback on our findings and recommendations for policy and practice.

Both groups were consulted via regular online meetings throughout the project. We asked these groups to provide feedback and responses to our emerging findings as the project developed. For example, we asked them to reflect on how our findings fit with (or did not fit with) their own knowledge and experience of commissioning, providing or attending an NHS Health Check. These discussions helped to shape our analysis, highlight areas of importance to different groups and inform the development of our recommendations. Our PPI group consistently observed a 'mismatch' between the focus of the NHS Health Check programme and their own health priorities, and a lack of understanding of the programme's purpose. This informed our analysis of data relating to attendees' understanding of their health needs and our overall focus on different groups' understanding of the purpose of the programme.

In our final meetings with each group, we asked our stakeholders for their input to help us to develop and refine recommendations to inform NHS Health Check policy, commissioning and delivery. We

For peer review only - http://bmjopen.bmj.com/site/about/guidelines.xhtml

also sought their input to help us to develop appropriate dissemination strategies, tailored project outputs and important audiences to share our findings.

Realist review methods

Our realist review followed Pawson's five iterative steps(19), as described in detail in our protocol.(16) These steps are summarised in **Table 1** below. We used the PRISMA (Preferred Reporting Items for Systematic Reviews and Meta-Analyses) checklist (where applicable) when writing our report (see **Supplementary File 1** for details).(20)

Table 1: Summary of methods

	**Aim**	**Approach**
Step 1: Locate existing theories	To identify existing theories that offer explanations from when, how and for whom the NHS Health Checks programme 'works'<br><br>See **Supplementary File 1**	Informal literature searching, focused on existing reviews and programme documentation (CD)<br>Drawing on experience and knowledge of project team and stakeholders (All)
Step 2: Search for evidence	To gather a body of literature containing data that could be used to refine the initial programme theory	Screening material included in two existing reviews(13, 14)<br>Additional searches across multiple databases (see **Supplementary File 2**)<br>Trawling NHS Health Check website for grey literature<br>Citation chaining<br>Email alert to capture new material (CD)
Step 3: Article selection	Within the material identified as described above, to select documents that could contribute relevant data for theory development	Screening title and abstract and then in full text (CD)<br>Inclusion of documents based on assessment of relevance and rigour(21)<br>10% of records screened in duplicate, with discrepancies resolved by discussion (CD, EG, GW)
Step 4: Extracting and organising data	To describe and organise included documents<br>To extract and code relevant data from included documents and begin analysis<br><br>See **Supplementary File 3.** The full set of extracted data is available on request.	Key characteristics of documents captured in an Excel spreadsheet (CD)<br>Relevant data related to our research question and focus coded in NVivo<br>10% of records coded in duplicate and checked by a third reviewer (CD, EG, GW)
Step 5: Synthesising evidence and drawing conclusions	To apply a realist logic of analysis to extracted data to develop context-mechanism-outcome configurations (CMOCs) related to the end of the NHS Health Check pathway. CMOCs are the building blocks of realist analysis,	Close examination and interpretation of data coded within each category to build CMOCs (CD)<br>Cross-case comparison to identify data that demonstrated similar or contrasting

For peer review only - http://bmjopen.bmj.com/site/about/guidelines.xhtml

	defining relationships between important outcomes, the mechanisms that produce them and the conditions in which they are likely to occur.<br><br>To develop a final programme theory	contexts and mechanisms that produced patterns of outcomes.<br>Iterative development of CMOCs as more data was considered and discussed by the project team and stakeholder groups (All)

As the project progressed, we made some minor changes to the protocol.

In Step 2, we anticipated a potential need for further searching for empirical data, but our project team agreed that the processes described above had identified sufficient material to meet our needs. During the analysis, we conducted a series of focused searches to identify material related to one substantive theory – 'street-level bureaucracy', which has been used to support our explanations of variation in delivery of the NHS Health Check.(22) The details of these searches are also reproduced in **Supplementary File 2**.

For Step 3, our original criteria were updated in light of the review project's chosen focus on what happens after measurements and risk assessments are completed. We sought in particular documents that contained data relating to later steps in the NHS Health Check pathway, including the provision of advice, signposting, referrals or prescriptions, and outcomes associated with these.

In Step 4, CD and EG coded a 10% set of the included documents independently and GW provided an additional check to ensure that all relevant data was captured and coding was applied consistently.

Results

Documents included in the review

In total, 124 documents contributed data to the review, including: 59 published research papers or reports; 20 documents reporting local evaluations; 34 conference materials (presentations, abstracts and posters) and 11 others (including policy reports, guidance, news articles and theses). Of these, 21 contributed data relating to the NHS Health Check programme as a whole, and the rest focused on specific localities. The processes of identifying, screening and selecting these are summarised below in Figure 1 (an adapted version of the PRISMA flow diagram).(20)

For peer review only - http://bmjopen.bmj.com/site/about/guidelines.xhtml

Figure 1: Summary of searching and selection processes

[Figure 1 here]

The main characteristics of the included documents are provided in **Supplementary File 3.** This table also indicates the extent to which each document contributed to the analysis, by showing the contribution of extracted data from each to specific CMOCs.

Review findings

Our findings describe what our included data told us about what happens at the end of an NHS Health Check, after the measurements and risk assessments are completed. Our explanations describe the reasoning and responses of three important groups involved in the programme: LA commissioners, NHS Health Check providers, and attendees. A narrative summary of our findings follows; it is based on our realist analysis that developed 86 CMOCs underpinned by the data included in the review. Overall, our findings illustrate how the delivery and outcomes of local NHS Health Check programmes and individual Heath Check encounters are shaped by a wide range of influences. To provide transparency, a detailed summary of the CMOCs developed and the data underpinning each is available in **Supplementary File 4**. We have included references to illustrative CMOCs to support the narrative that follows. CMOCs are labelled according to the perspective that they focus on, i.e. commissioner-focused CMOCs are designated ‘C’, provider-focused CMOCs ‘P’ and attendee-focused CMOCs ‘A’.

Understanding and engagement with the NHS Health Check

Our review findings point to variation in understandings of the primary purpose of the NHS Health Check. This variation is a critical factor that determines how the programme is commissioned, delivered and received. The programme was designed with two aims in mind: early identification of cases of diagnoseable illness or individuals with ‘high’ risk, and early intervention to support individuals to manage and reduce their CVD risk, via prescribing or by prompting or enabling behaviour change. Further to these, the programme is also cast as an opportunity to address disparities in CVD risk factors across England, although it maintains a focus on individual behaviours. Whilst

For peer review only - http://bmjopen.bmj.com/site/about/guidelines.xhtml

there is a focus in the literature on inequality in relation to invitation and coverage of the check,(23, 24) our review uncovered very little data and almost no focus on the relationship between inequalities and the delivery of advice and referral. This gap is also reflected in the evidence collated to support the recently published PHE/OHID-led review.(12)

Our analyses of the data suggest that commissioners and providers may have a tendency to prioritise some aspects of the programme over others, for example, increasing volume of delivery and delivery of risk assessments over risk management. This prioritisation is then reflected in local commissioning decisions and delivery models. When the programme is understood primarily as an opportunity to screen for cases of existing CVD or near-CVD (very high risk factors), responsibility for programme delivery and outcomes is likely to rest with primary care, and specifically in general practice, where most NHS Health Checks are provided, and where clinical and especially pharmacological follow-up should happen (see, e.g. *CMOCs C9-C10* in **Supplementary File 4**). This perspective leads to an emphasis on high-volume throughput, increasing coverage of the programme, and a focus on efficient delivery of the minimum requirements for each check, collecting mandatory data and communicating risk scores. At the other end of the spectrum, where commissioners and providers are more oriented towards using the programme as a means of supporting behaviour change in order to reduce or manage CVD risk, delivery reflects this, with greater emphasis on advice or coaching, and facilitating onward referrals to 'lifestyle services', such as smoking cessation or weight management programmes (*CMOCs C1-C8)*.

Our data demonstrates how such differences in local priorities for the NHS Health Check can drive variation in who delivers the check and the training they receive, the settings in which checks are delivered and the time allocated to each check (*CMOCs C1-C8, P14, P26*), all of which go on to affect how attendees experience the programme (*CMOCs A12-A17, A30-A37)*. In addition, these differences can affect the availability, accessibility and connectedness of referral pathways to local 'lifestyle services' that might be offered to attendees post-check, and important differences in the information that LAs collect, how they monitor and evaluate programme delivery, as well as funding models that can incentivise certain aspects of delivery (*CMOCs C2-C3, C5-C8)*. Commissioners and

For peer review only - http://bmjopen.bmj.com/site/about/guidelines.xhtml

providers each influence how the programme is shaped and implemented at local levels, exercising discretion in determining the remit and focus of the programme.

This double layer of discretion in how the NHS Health Check is ultimately enacted in different local areas means that the relationship between LA commissioners and providers is an important factor in determining what local delivery looks like. Commissioners determine service specifications and funding models and the scope of monitoring of programme delivery, but (unless they are themselves providers, as is the case for some provision in a few LAs) their day-to-day influence on actual delivery is limited (*CMOC C4*). This is a particular concern as prevailing scepticism (in particular amongst GPs) about the programme's effectiveness and concerns about the potential for overdiagnosis has led to disengagement amongst some providers. Such concerns, however, must be balanced with their reliance on income generated by the programme (*CMOCs P1-P5*). Differences in levels of engagement with the programme can reflect different understandings about its primary purpose and concerns about its ability to meet its aims, but they can also be fuelled by more practical concerns, including competing priorities and workload pressures, and the need to deliver NHS Health Checks and appropriate follow-up with limited resources (see below and *CMOCs P9-P13, P15-P18*).

Local delivery models send important signals to attendees that inform how they understand and engage with the programme in turn. Checks delivered in general practice with a focus on completing mandatory measurements and risk assessments carry the implication that the check should be understood as a screening test or a health 'MOT', that may or may not result in the need for clinical intervention (*CMOCs A1*, *A12*). Delivery in community settings, by providers trained specifically to deliver coaching or behavioural support, or otherwise with a greater focus on delivering advice or referrals, sends a different message *(CMOC A14).* In some areas, NHS Health Checks have been delivered within a wider integrated lifestyle service, facilitating connections with lifestyle services, and potentially additional services such as link workers or social prescribers. These models may enable providers to offer more holistic support that better meets the needs of individual attendees; much data from the attendee perspective suggests that this may help to generate better engagement with the programme and its aims.

For peer review only - http://bmjopen.bmj.com/site/about/guidelines.xhtml

Our data also suggest that attendees' prior expectations of the check also inform their response, and so it is important that national and local advertising about the programme are clear about its remit and purpose. Providers can transmit 'soft' signals to attendees about the programme's purpose and value, and attendees will pick up on these (*CMOCs A17-24)*. They have the potential to convey urgency, and even induce fear and anxiety in attendees, but they may also (intentionally or otherwise) imply that the check is a 'box ticking' exercise.

Finally, it is important to acknowledge that attendees' themselves can exercise discretion in their response to their check, regardless of how providers approach its delivery. The programme's focus on individual behaviour creates high expectations of individual action to address any 'lifestyle risk factors' identified. Inevitably, attendees may face considerable challenges in implementing behaviour changes within their own lives, where they too may have limited resources (*CMOCs A38-A34*). Some literature has drawn attention to the risks inherent in this individual focus, including the potential for positive health impacts to be realised only by those with the capacity to make significant lifestyle changes, and so unfairly distributing any benefits amongst attendees.(25) Such a focus risks increasing rather than decreasing health inequalities.

Practical constraints limiting the NHS Health Check

Commissioners and providers face substantial practical constraints that limit and inform their exercise of discretion in programme delivery. As noted above, the decisions of LA commissioners that are enacted in funding models, service specifications and monitoring regimes set important boundaries for providers, potentially incentivising different delivery methods and priorities. Commissioners' decisions must take into account restricted funding for public health initiatives (*CMOCs C12-C14*). Central government grants to LAs have fallen substantially since 2010. Since public health responsibilities were transferred to LAs in 2013, the public health grant has decreased by 13% in real terms.(26)(30) Spending on the NHS Health Check programme fell by 21% between 2015/16 and 2019/20, and spending on 'lifestyle services' that could potentially support attendees post-check has also fallen: expenditure on smoking cessation initiatives fell by one third over the same period, adult alcohol and drug services by 17% and weight management services by 5%.(27)(31) Reductions in

For peer review only - http://bmjopen.bmj.com/site/about/guidelines.xhtml

funding of this scale necessarily limit what commissioners can purchase (*CMOCs C14, A35-A37)*. Our findings raise the question of whether public health funding overall is adequate to support the programme's more ambitious aim of preventing CVD and reducing CVD inequalities by reducing or managing behavioural risk factors. In particular, it is apparent that the delivery of personalised, individual advice and discussion during checks, and the offer of further support afterwards, is a more intensive proposition than a programme focused on case finding (*CMOCs P9-P13)*.

Providers face other constraints. There is a need to ensure that staff involved in delivering checks are competent to do so, but some data suggest that not all providers feel confident in delivering support for behaviour change (*CMOCs P20-P23)*. Training, too, must reflect the programme's aims both to identify and to address CVD risk factors (*CMOC P26*). Wherever checks are delivered in general practice or community pharmacy settings, providers face competing priorities and demands on their time. Workload and time pressure may push providers towards 'leaner' delivery, focused on mandatory tests and capturing required measurements, leaving little time for discussion, advice and offers of referral (*CMOCs P9-P14)*.

There is a crucial relationship between the practical constraints that commissioners and providers face and their understanding and engagement with the programme. It is likely that both groups of actors adapt their understanding of the programme based on what they know about the limited resources available to deliver and support it. Downward pressure on funding inevitably incentivises leaner delivery models and, as our findings make clear, these models tend to favour the 'early identification' or case finding purpose of the programme. Provider scepticism is also likely to be compounded by the sense that both the programme itself, and subsequent services on which it might depend are underfunded and inaccessible (*CMOCs P1-P3)*.

Finally, our data make clear the impact of the varied delivery models on attendees' experience of the programme and on what they are offered (or not) post-check (*CMOCs A1, A5-A6, A11-A14, A30-A32, A34, A38-A40)*. It is unclear which delivery models allow or incentivise providers to deliver the meaningful, personalised and ongoing advice and support attendees might need. Existing ethnographic research has demonstrated that the time-constrained and highly structured nature of the

For peer review only - http://bmjopen.bmj.com/site/about/guidelines.xhtml

NHS Health Check assessments impede meaningful discussion that prioritises understanding individuals' circumstances.(28, 29) Limited access to 'lifestyle services' means these are inaccessible to many.

Variation in NHS Health Check delivery models: street-level bureaucracy

Our review findings demonstrate wide variation in how the NHS Health Check programme is implemented locally, with a specific focus in variation in the delivery of what happens after measurements and risk assessments have been completed. Inconsistencies in the recording of these activities (especially the delivery and uptake of advice and referrals post-check) prevent the development of a comprehensive picture of this variation across England. However, our review findings provide a starting point to improve understanding of what influences delivery in these areas, highlighting the discretion available to LA commissioners and individual NHS Health Check providers in making decisions about how the programme is delivered on the ground. In addition, they highlight how differences in delivery models affect how the checks are experienced by attendees, and what colours their responses to the information they receive and any offers of further intervention.

Our understanding of the processes at work in driving variation draws on Lipsky's concept of 'street-level bureaucracy', borrowed from the international relations literature.(22, 30) Previous research on the NHS Health Check,(31) and the implementation of other health policies in the UK(32, 33) have used the same theory to add explanatory value. Lipsky's framework emphasises the discretion of those charged with implementing national policies or programmes, as well as their responses to working with limited resources. For the NHS Health Check programme, it is clear that while commissioners and providers are working within the broad constraints of a legal framework(4, 5) and guidance issued by PHE(2) (now OHID), their decisions and everyday practice in delivering checks effectively determine the remit and purpose of the programme at local levels. As Lipsky describes it: "*the decisions of street-level bureaucrats, the routines they establish, and the devices they invent to cope with uncertainties and work pressures, effectively become the public policies they carry out".(30)*

For peer review only - http://bmjopen.bmj.com/site/about/guidelines.xhtml

The extent to which discretion can be exercised in relation to the NHS Health Check may be greatest at the end of the programme pathway. Processes relating to earlier steps – the identification of the eligible population, invitation and the actual measurements and risk assessments to be administered – are restricted by the programme's legal framework and clear guidance, leaving little room for local interpretation or adaptation. The later steps – especially the delivery of advice and referrals – are less prescribed and more dependent on local delivery models and other local services. Activity in these areas is less well recorded and monitored, leaving LA commissioners and providers with more discretion to determine what day-to-day local delivery of checks will look like.

Although Lipsky's original framework focuses on those directly engaged in the delivery of policies and their interaction with the recipients or subjects of those policies (in our case, the interaction of providers with attendees) our analysis also highlights the discretion of LA commissioners who make decisions about local programme specifications and support. Whilst they may not directly interact with the public, commissioners must also interpret the requirements of the programme and exercise their own discretion to ensure it meets local needs and is delivered within local constraints. The approach of the LA directly affects commissioned providers, and this double layer of discretion forms the local contexts in which NHS Health Checks are delivered across England.

Taken together, the CMOCs developed in this review point to the influence of both commissioners' and providers' understanding and engagement with the programme, as well as the effects of practical constraints that drive decision making in relation to programme delivery. These two aspects mirror Lipsky's concepts of *discretion* in the enactment of policies and programmes, and the effects of their responses to *limited resources*. Attendees' experience of, and response to the programme is affected both by the outcomes of commissioner and provider decisions, but also by other external factors related to their individual circumstances, which may be difficult to align with what the NHS Health Check expects of them.

For peer review only - http://bmjopen.bmj.com/site/about/guidelines.xhtml

Discussion

Summary of findings

The success of the NHS Health Check programme as an intervention that aims to support individuals to manage and reduce their CVD risk rests on what happens to attendees when the measurements and risk assessments have been completed. While case-finding is an important indicator of success, the delivery of advice, offers of signposting, referrals or other support, and attendees' responses to these, is equally crucial for the programme to prevent ill-health and reduce inequalities in the longer term. Our focus on this area reflected the importance of this step – confirmed by our two stakeholder groups – but also the relative lack of attention that it has received in the existing research literature. Our review therefore sought to examine the final steps in the NHS Health Check programme pathway, to understand the factors that influence the delivery of these parts of the check and what follows, and how they are received by those who receive checks.

The NHS Health Check as a whole has been the subject of several previous evidence synthesis projects, which have considered many aspects of the programme, and have similarly identified wide variation in delivery models and outcomes. Our review is the first to use a realist approach, and the first to focus specifically on the steps that follow the measurements and risk assessments made during each check. Relevant findings from existing reviews correspond with our own. The two PHE-commissioned rapid reviews identified some limited evidence of geographical variation in referrals, and captured qualitative data describing providers' doubts and scepticism about the programme, and perceived training needs. These reviews also included qualitative studies focused on attendees, which described attendees' perspectives on the quality of information delivered during checks and the important constraints imposed by "environmental", "resource" and "time" factors that limit attendees' capacity to make and sustain behaviour changes.(13, 14) Another synthesis coded evidence relating to the behaviours of commissioners, providers and attendees in relation to the programme, and reported similar findings related to providers' skills, attitudes and beliefs, as well as the resources available to deliver checks and the need to take account attendees' wider "social contexts" when delivering advice.(34)

For peer review only - http://bmjopen.bmj.com/site/about/guidelines.xhtml

Our review extends the work undertaken in these reviews, by explaining how and why variation in how the programme is delivered comes about, with a particular focus on what happens after the measurements and risk assessments are completed. Our findings point to the significance of the exercise of discretion by LA commissioners and NHS Health Check providers in the delivery of checks. Commissioners' decisions in relation to programme implementation, funding and monitoring, and providers' actions to deliver checks on the ground are influenced by multiple factors. These include their understanding of the primary purpose of the programme and engagement with its aims, but these attitudes towards the programme – and commissioners' and providers' actions in organising and providing checks – are constrained by important practical factors.

Prevailing conditions, including limited funding for public health programmes and services overall, current funding and monitoring arrangements for the NHS Health Check programme, and competing priorities for many providers tend to push towards a delivery model that prioritises the programme's aim of early detection – case finding. The prioritisation of this element of the programme may be to the detriment of work that could help to prompt or support behaviour change. These constraints raise ethical questions about the programme: if it is oriented towards early identification of risk, but cannot genuinely support early intervention to help manage and reduce risks (bar prescribing), where does this leave attendees? There is a need to consider the relationship between the NHS Health Check programme and the wider landscape of local and national services that can be offered to attendees.

In some local areas, there is evidence that commissioners and providers work against this tide, using their local NHS Health Checks as a means of supporting attendees to make behaviour changes that could help to reduce their CVD risk. Our final programme theory diagram summarises these findings and is presented below in **Figure 2**.

Figure 2: Final programme theory

[Figure 2 here]

For peer review only - http://bmjopen.bmj.com/site/about/guidelines.xhtml

Covid-19 and the NHS Health Check

We undertook this review during the Covid-19 pandemic, which had a major impact on delivery of the NHS Health Check. In April 2020, the programme was effectively paused.(35, 36) To support resumption of delivery, PHE issued 'restart guidance' in April 2021, encouraging LAs to consider restarting the programme, dependent on local safety assessments and the need to prioritise the vaccination programme.(37) This document acknowledged the pressures faced by general practice in particular, and urged LA commissioners to consider 'alternative' providers. Other communications from PHE and the Department of Health and Social care during the pandemic highlighted the potential benefits of the NHS Health Check in relation to identifying risk factors for severe Covid-19 outcomes, as many CVD risk factors are also associated with higher risks of hospitalisation and death from Covid-19.(38-40)

In light of our findings, we note that the effects of the pandemic have the potential to exacerbate some existing contexts that may adversely affect delivery of the programme's aims. In particular, pressure to 'catch up' and concerns about delayed or missed diagnoses could lead to a more intense focus on early detection and case finding.(41) Workload pressures affecting primary care providers may also increase disengagement from the NHS Health Check, and LA commissioners must consider these effects as they restart the programme in each local area.

Implications for policy and practice

Our review findings raise a number of important questions for policy makers, commissioners and providers to consider in relation to the NHS Health Check. Our main recommendation is that all three of these groups (and future researchers) should increase their focus on evaluating and improving the delivery of the later steps in the programme pathway, i.e. on what happens to attendees after the measurements and risk assessments have been completed. Our more detailed recommendations are summarised below in **Table 2**. They are organised around four important and inter-connected principles, developed in consultation with our PPI and professional stakeholder groups: clarifying the purpose of the programme; increasing engagement with the programme; focusing the Health Check

For peer review only - http://bmjopen.bmj.com/site/about/guidelines.xhtml

on attendee needs; and improving links between the NHS Health Check and other programmes and services.

Table 2: Summary of recommendations based on review findings

**Our recommendation**	**Rationale**
Ensure all national and local guidance and programme documentation reflects the importance of the programme's aims in relation to both early identification of CVD risk, and early intervention to manage/reduce that risk.	Different interpretations of the primary purpose of the programme drive variation in commissioning and delivery, and subsequently in attendee experience.
Assess overall programme funding and local funding arrangements in relation to delivery of (all aspects of) the programme's aims.	Commissioner, provider and attendee scepticism about the programme can undermine its delivery; inadequate resources for the programme and support services for attendees post-check can reduce engagement. Funding arrangements may incentivise leaner or more intensive checks.
Review national and local data monitoring and evaluation of the programme, to ensure that data relating to all key aspects of the programme (and especially the final steps in the NHS Health Check pathway) are captured.	Data collection is currently focused on invitation, uptake and coverage, incentivising high-volume, less intensive delivery that focuses on mandatory data points. Data on advice and referral (including data relating to potential disparities in provision) are needed to evaluate the delivery of these steps.
Review training for providers to assess its ability to support all aspects of programme delivery, including the final steps in the NHS Health Check pathway.	Providers and attendees have identified deficiencies in skills and knowledge, especially in relation to the delivery of advice, support and referrals. All providers should receive adequate training to ensure that they can deliver these aspects of a check or signpost/refer attendees to an appropriate source of support.
Review connections between the programme and national and local services that could offer further support for attendees, and options for longer term follow-up after measurements and risk assessments are completed.	The success of the programme rests in part on its connections with other services that could offer further support for attendees. Strengthening these connections could allow the NHS Health Check to act as a gateway to these services (as well as to primary care/general practice when necessary)
Continue to produce evidence for programme effectiveness and address the relative scarcity of evidence focused on later steps in the NHS Health Check pathway.	Commissioner, provider and attendee scepticism about the programme can undermine its delivery; evidence from research and evaluation should go beyond a focus on increasing coverage of the programme and inform practical recommendations for good practice in delivery.

For peer review only - http://bmjopen.bmj.com/site/about/guidelines.xhtml

Our recommendations must be considered alongside those made by PHE/OHID in their recently published national review of the Health Checks programme.(6) They support several of the recommendations made by OHID, and in particular the identification of the need to "build sustained engagement" and "create a learning system". In these areas, OHID's specific proposals include: ensuring a clear focus on the programme's aim to "[promote] lasting health and wellbeing"; developing a national training offer with a focus on supporting behaviour change; providing sufficient provision of post-check services; and continued evaluation of the programme.

However, our findings raise concerns about the implementation of some of the recommendations from the national review. In particular, two major recommendations ("start younger" and "address more conditions") propose to significantly expand the scope and coverage of the NHS Health Check programme. Our findings relating to attendee experience suggest that moves to make the programme more holistic may be welcomed by some, but indicate that policy makers should be cautious about any programme expansion. There is a risk that expanding the programme's scope could add to existing confusion about its primary purpose and drive further local variation in delivery. Existing capacity in primary care and community and public health services also limit the feasibility of these proposals. Without sufficient appropriate follow-up, extensions of the programme's remit risk leaving more attendees with few options for ongoing support to help them to address or manage any risks or conditions identified. Without additional investment in services and convincing evidence of their clinical and cost-effectiveness, such expansion also risks increasing provider scepticism and disengagement from the programme.

OHID's proposal to launch a digital version of the NHS Health Check should also consider our findings, and in particular, ensure that the final steps in the programme pathway are not neglected. In any delivery format, there is a need to ensure that where risks are identified, relevant advice and links to appropriate services are provided. Finally, we note that OHID's recommendation to continue to increase participation reinforces the existing focus on invitation and uptake of the NHS Health Check. Policy makers should be cognisant that measures that encourage and incentivise high volume delivery

For peer review only - http://bmjopen.bmj.com/site/about/guidelines.xhtml

of checks can detract from the delivery of high quality, personalised advice and ongoing support for longer-term behaviour change.

Strengths and limitations of this review

Our review has developed novel interpretations of existing secondary data relating to what happens at the end of the NHS Health Check pathway. Our close examination of what happens after the measurements and risk assessments have been completed during checks helps to address the relative lack of research on this particular aspect of programme delivery. The review project was strengthened by close working with our diverse PPI and professional stakeholder groups, who helped us to focus the review, provided detailed feedback on emerging findings and shaped our interpretation of the data and the development of recommendations. We included a diverse range of material in the review, and in particular, drew on the learning captured in a wide range of grey literature including local evaluations and conference materials.

As with any review, our findings are limited by the availability and quality of the literature. The material included in the review covers a wide date range and some older material may be less applicable to the present day. However, we considered each piece of data carefully before inclusion and aimed to select material that spoke to still-relevant contexts, mechanisms and outcomes. Our stakeholders also helped us to confirm the contemporary relevance of our findings. Our CMOCs vary in terms of the volume and rigour of the data that underpin them. We have provided a full and transparent account of that data in **Supplementary Files 3 and 4,** so that the strength of each is made clear to readers.

Conclusions

Our review has revealed wide variation in the delivery of advice and support, and onward signposting and referral for attendees and identified explanations for this variation. We have identified a wide range of influences that affect how LA commissioners and NHS Health Check providers develop and deliver the programme at local levels across England, which affect how attendees experience and respond to their check. Our analysis explains how differences in understanding of the primary purpose of the programme influences commissioning and implementation, and how practical constraints limit

For peer review only - http://bmjopen.bmj.com/site/about/guidelines.xhtml

what can be delivered within the programme's remit and existing resources. Based on our findings, we developed a set of recommendations for policy makers, commissioners and providers to inform future programme development. Our recommendations centre on the need for greater emphasis to be placed on the final steps in the NHS Health Check programme pathway, including in national and local guidance and programme documentation, funding models, provider training, monitoring and evaluation of the programme, and in considering how the NHS Health Check can be better linked with wider services and programmes.

List of abbreviations

BMI: Body mass index

CINAHL: Cumulative Index to Nursing and Allied Health Literature

CMOC: Context-mechanism-outcome configuration (see glossary for a definition)

CVD: Cardiovascular Disease

DHSC: Department of Health and Social Care

GP: General Practitioner

HMIC: Health Management Information Consortium

IPT: Initial Programme Theory

LA: Local authority

MOT: 'Ministry of Transport', test to determine if cars are roadworthy

NHS: National Health Service

NICE: National Institute for Health and Care Excellence

OHID: Office for Health Improvement and Disparities

PHE: Public Health England

PPI: Patient and Public Involvement

For peer review only - http://bmjopen.bmj.com/site/about/guidelines.xhtml

For peer review only

PRISMA: Preferred Reporting Items for Systematic Reviews and Meta-Analyses

RAMESES: Realist and Meta-narrative Evidence Syntheses: Evolving Standards

Required statements

Acknowledgements

We would like to express our thanks for the help, feedback and advice provided by all of our wonderful stakeholders and patient and public involvement contributors, and for the continued engagement of PHE and OHID and their help with the recruitment of stakeholders and opportunities for dissemination of our findings and outputs.

Ethics approval

Not applicable for this review

Author contributions

JK and GW conceived the research project and all of the authors contributed to its design. GW provided methodological support. CD devised and carried out the literature searches and led the analysis and interpretation of the data. The other authors (EG, VH, JK, GW) contributed to the analysis. VH led the PPI involvement. CD drafted the manuscript and the other authors (EG, VH, JK, GW) provided feedback. All of the authors read and approved the final manuscript.

Funding statement

This project was funded by the National Institute for Health Research (NIHR) Health Services and Delivery Research programme (NIHR129209). The views expressed are those of the authors and not necessarily those of the NIHR or the Department of Health and Social Care.

Competing interests

CD and GW are both members of the Royal College of General Practitioners (UK) Overdiagnosis and Overtreatment Group. GW is an NHS General Practitioner and deputy chair of the National Institute for Health Research Health Technology Assessment Prioritisation Committee: Integrated Community Health and Social Care Panel (A) and member of the Methods Group (A).

For peer review only - http://bmjopen.bmj.com/site/about/guidelines.xhtml

Data sharing statement

The datasets generated and/or analysed during the current study are not publicly available due to copyright and licensing restrictions but are available from the corresponding author on reasonable request.

References

1. NHS. NHS Health Check 2020 [Available from: https://www.nhs.uk/conditions/nhs-health-check/.
2. Public Health England. NHS Health Check Best practice guidance For commissioners and providers. 2020.
3. HM Government. Levelling Up. Levelling Up the United Kingdom. Her Majesty's Stationery Office; 2021 2 February 2022.
4. Great Britain. The Local Authorities (Public Health Functions and Entry to Premises by Local Healthwatch Representatives) Regulations. Part 2 Public Health Functions: Conduct of Health Checks. United Kingdom; 2013.
5. Great Britain. The Local Authorities (Public Health Functions and Entry to Premises by Local Healthwatch Representatives) Regulations. Part 2: Public Health Functions: Health check assessment. United Kingdom; 2013.
6. Office for Health Improvement and Disparities. NHS Health Check Programme review. 2021.
7. Abdalrahman B, Soljak M. NHS health checks: an update on the debate and program implementation in England. J Ambul Care Manage. 2015;38(1):5-9.
8. McCartney M. Where's the evidence for NHS health checks? BMJ. 2013;347:f5834.
9. Capewell S, McCartney M, Holland W. Invited debate Response to Waterall et al. J Public Health (Oxf). 2015;37(2):185-6.
10. Ford J, Ekeke N, Lahiri A, Kelly MP. Making the case for prevention. Cambridge, UK: A commissioned report by Cambridge Public Health, University of Cambridge, for the Health Foundation,; 2021.
11. Public Health England. Review of NHS Health Checks: terms of reference. 2020.
12. Office for Health Improvement and Disparities. NHS Health Check Programme review. Annex B: a summary of analyses and evidence on the current NHS Health Check programme. 2021.
13. Usher-Smith JA, Mant J, Martin A, Harte E, MacLure C, Meads C, et al. NHS Health Check Programme rapid evidence synthesis. Cambridge, UK: The Primary Care Unit, University of Cambridge, RAND Europe, Public Health England; 2017.
14. Tanner L, Kenny R, Still M, Pearson F, Bhardwaj-Gosling R. NHS Health Check Programme Rapid Review Update. University of Sunderland, Newcastle University, Public Health England; 2020.
15. Patel R, Barnard S, Thompson K, Lagord C, Clegg E, Worrall R, et al. Evaluation of the uptake and delivery of the NHS Health Check programme in England, using primary care data from 9.5 million people: a cross-sectional study. BMJ Open. 2020;10(11):e042963.
16. Duddy C, Wong G, Gadsby EW, Krska J, Hibberd V. NHS Health Check programme: a protocol for a realist review. BMJ Open. 2021;11(4):e048937.
17. The RAMESES Project. Quality Standards for Realist Synthesis (for researchers and peer-reviewers). 2014.
18. Wong G, Greenhalgh T, Westhorp G, Buckingham J, Pawson R. RAMESES publication standards: realist syntheses. BMC Med. 2013;11(1):21.
19. Pawson R. Realist synthesis: new protocols for systematic review. 2006. In: Evidence-based policy: a realist perspective [Internet]. [73-104].
20. Page MJ, McKenzie JE, Bossuyt PM, Boutron I, Hoffmann TC, Mulrow CD, et al. The PRISMA 2020 statement: an updated guideline for reporting systematic reviews. BMJ. 2021;372:n71.
21. Wong G. Data gathering in realist reviews: Looking for needles in haystacks. 2018. In: Doing Realist Research [Internet]. London: SAGE Publications Ltd; [131-46].

For peer review only - http://bmjopen.bmj.com/site/about/guidelines.xhtml

22. Lipsky M. Toward a theory of street-level bureaucracy. Madison, WI: Institute for Research on Poverty (IRP), University of Wisconsin,; 1969.
23. Bunten A, Porter L, Gold N, Bogle V. A systematic review of factors influencing NHS health check uptake: invitation methods, patient characteristics, and the impact of interventions. BMC public health. 2020;20(1):93.
24. Martin A, Saunders CL, Harte E, Griffin SJ, MacLure C, Mant J, et al. Delivery and impact of the NHS Health Check in the first 8 years: a systematic review. Br J Gen Pract. 2018;68(672):e449-e59.
25. Stol YH, Schermer MHN, Asscher ECA. Omnipresent Health Checks May Result in Over-responsibilization. Public Health Ethics. 2017;10(1):35-48.
26. The King's Fund. Spending on public health 2021 [Available from: https://www.kingsfund.org.uk/projects/nhs-in-a-nutshell/spending-public-health.
27. Finch D, Marshall L, Bunbury S. Why greater investment in the public health grant should be a priority. The Health Foundation; 2021.
28. Cupit C. An ethnographic study of cardiovascular disease prevention: the social organisation of measures, knowledge, interventions and tensions in English general practice: University of Leicester; 2018.
29. Strutt E. Patient-centred care: Patients' experiences of and responses to the National Health Service (NHS) Health Check programme in general practice. Durham: Durham University; 2011.
30. Lipsky M. Street-Level Bureaucracy: Dilemmas of the Individual in Public Service. 30th Anniversary Edition ed. New York, New York: Russell Sage Foundation; 2010.
31. Gale N, Dowswell G, Greenfield S, Marshall T. Street-level diplomacy? Communicative and adaptive work at the front line of implementing public health policies in primary care. Soc Sci Med. 2017;177:9-18.
32. Bergen A, While A. ‘Implementation deficit’ and ‘street-level bureaucracy’: policy, practice and change in the development of community nursing issues. Health Soc Care Community. 2005;13(1):1-10.
33. Cooper MJF, Sornalingam S, O’Donnell C. Street-level bureaucracy: an underused theoretical model for general practice? Br J Gen Pract. 2015;65(636):376.
34. Atkins L, Stefanidou C, Chadborn T, Thompson K, Michie S, Lorencatto F. Influences on NHS Health Check behaviours: a systematic review. BMC Public Health. 2020;20(1):1359.
35. NHS England and Improvement. COVID-19 Prioritisation within Community Health Services [Letter]. 2020.
36. Thompson K. NHS Health Check e-Bulletin - April 2020 Operational Update. 2020. Contract No.: 30th April 2020.
37. Public Health England. NHS Health Check: Restart Preparation. 2021. Contract No.: Version 11: 09 April 2021.
38. Public Health England. NHS Health Check e-Bulletin - August 2020. 2020. Contract No.: 5th January 2022.
39. Public Health England. Summary: An Umbrella Review on Cardiovascular Risk Factors, Cardiovascular Disease and COVID-19. 2021.
40. Cabinet Office. Our plan to rebuild: The UK Government's COVID-19 recovery strategy. 2020. Contract No.: 24th February 2022.
41. Oxford Academic Health Science Network. CVD prevention during and after the COVID-19 pandemic. Getting It Right First Time, Association of Directors of Public Health; 2020.

For peer review only - http://bmjopen.bmj.com/site/about/guidelines.xhtml

For peer review only

For peer review only - http://bmjopen.bmj.com/site/about/guidelines.xhtml

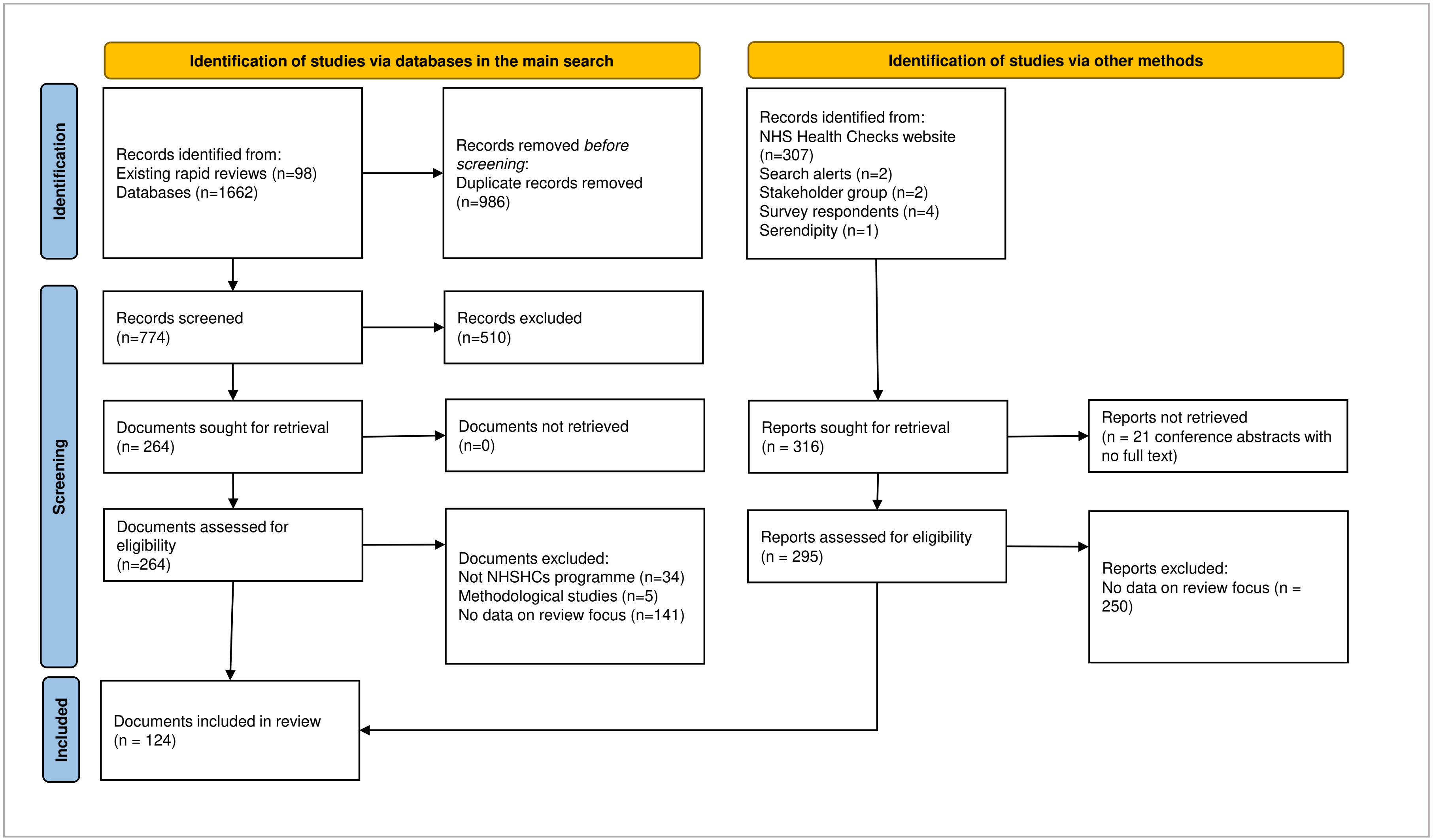
Identification of studies via databases in the main search
Identification of studies via other methods
Identification
Records identified from:
Existing rapid reviews (n=98)
Databases (n=1662)
Records removed *before screening*:
Duplicate records removed (n=986)
Records identified from:
NHS Health Checks website (n=307)
Search alerts (n=2)
Stakeholder group (n=2)
Survey respondents (n=4)
Serendipity (n=1)
Screening
Records screened (n=774)
Records excluded (n=510)
Documents sought for retrieval (n= 264)
Documents not retrieved (n=0)
Reports sought for retrieval (n = 316)
Reports not retrieved (n = 21 conference abstracts with no full text)
Documents assessed for eligibility (n=264)
Documents excluded:
Not NHSHCs programme (n=34)
Methodological studies (n=5)
No data on review focus (n=141)
Reports assessed for eligibility (n = 295)
Reports excluded:
No data on review focus (n = 250)
Included
Documents included in review (n = 124)

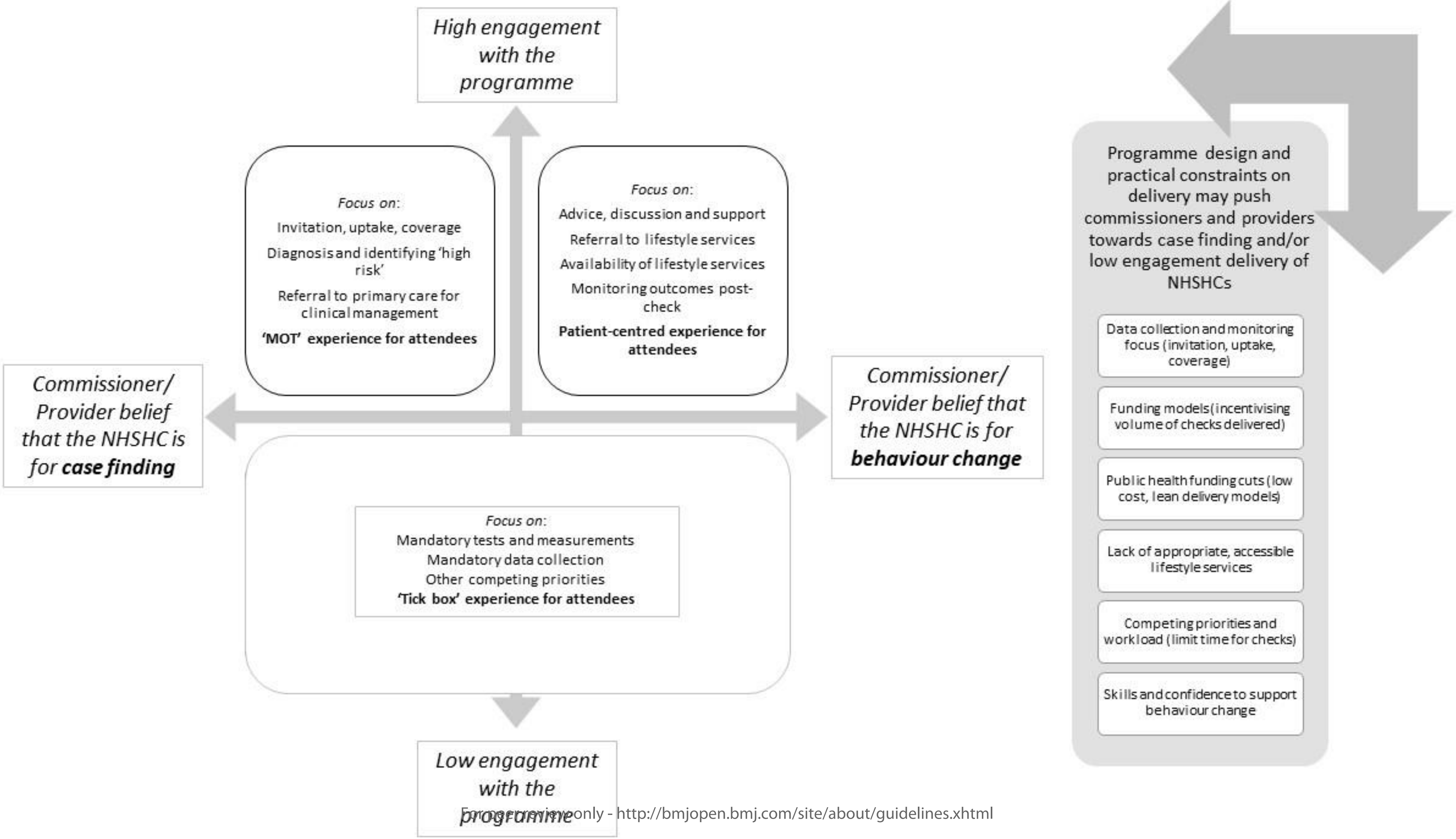

For peer review only - http://bmjopen.bmj.com/site/about/guidelines.xhtml

Supplementary File 1

This file contains a glossary of realist terminology, a diagram summarising our initial programme theory and the focus of the realist review and PRISMA and RAMESES checklists.

Glossary of realist terminology

Context: in realist research, the conditions or circumstances in which mechanisms that generate observed outcomes are 'triggered' or activated.

Context-mechanism-outcome configuration (CMOC): a heuristic used to present a realist causal explanation for an outcome, presented as a relationship between some particular context(s) and mechanism(s).

Demi-regularity: a semi-predictable pattern of outcomes that occur in the same context(s).

Mechanism: in realist research, the underlying context-sensitive causal force that generates an outcome, often conceptualised as the response of an individual actor to important context(s).

Programme theory: a set of theoretical explanations about how a programme, intervention or process is understood to work. Realist programme theories explain or the process by which outcomes of interest are thought to be generated, using causal explanations captured in the form of CMOCs.

Substantive theory: an established, formal theory drawn from any discipline that can be used to help understand the programme, intervention or process under examination.

For peer review only

Initial programme theory diagram

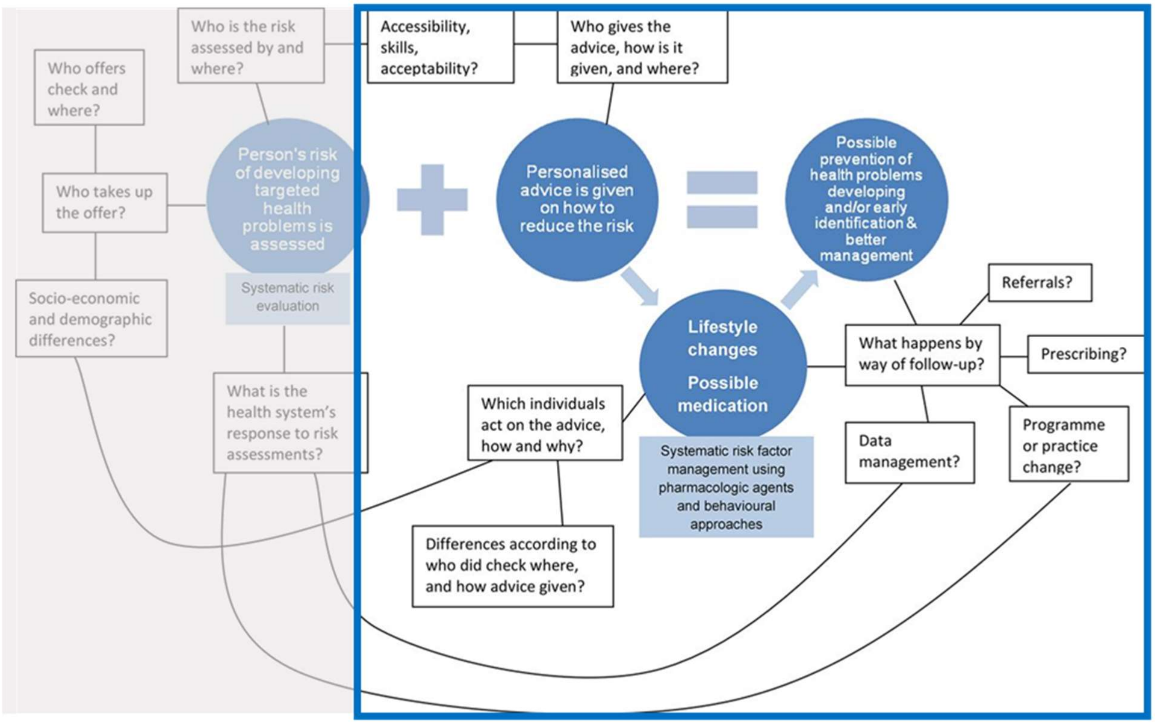

Initial programme theory for the realist review with project focus highlighted

review only

PRISMA checklist

Reporting checklist for systematic review (with or without a meta-analysis).

Based on the PRISMA guidelines.

Instructions to authors

Complete this checklist by entering the page numbers from your manuscript where readers will find each of the items listed below.

Your article may not currently address all the items on the checklist. Please modify your text to include the missing information. If you are certain that an item does not apply, please write "n/a" and provide a short explanation.

Upload your completed checklist as an extra file when you submit to a journal.

In your methods section, say that you used the PRISMAreporting guidelines, and cite them as:

Page MJ, McKenzie JE, Bossuyt PM, Boutron I, Hoffmann TC, Mulrow CD, Shamseer L, Tetzlaff JM, Akl EA, Brennan SE, Chou R, Glanville J, Grimshaw JM, Hróbjartsson A, Lalu MM, Li T, Loder EW, Mayo-Wilson E, McDonald S, McGuinness LA, Stewart LA, Thomas J, Tricco AC, Welch VA, Whiting P, Moher D. The PRISMA 2020 statement: An updated guideline for reporting systematic reviews

		Reporting Item	Page Number
**Title**			
Title	#1	Identify the report as a systematic review	1
**Abstract**			
Abstract	#2	Report an abstract addressing each item in the PRISMA 2020 for Abstracts checklist	2
**Introduction**			
Background/rationale	#3	Describe the rationale for the review in the context of existing knowledge	3-6
Objectives	#4	Provide an explicit statement of the objective(s) or question(s) the review addresses	6, protocol
**Methods**			

For peer review only

Eligibility criteria	#5	Specify the inclusion and exclusion criteria for the review and how studies were grouped for the syntheses	8-9, protocol
Information sources	#6	Specify all databases, registers, websites, organisations, reference lists, and other sources searched or consulted to identify studies. Specify the date when each source was last searched or consulted	8, Supplementary File 2
Search strategy	#7	Present the full search strategies for all databases, registers, and websites, including any filters and limits used	Supplementary File 2
Selection process	#8	Specify the methods used to decide whether a study met the inclusion criteria of the review, including how many reviewers screened each record and each report retrieved, whether they worked independently, and, if applicable, details of automation tools used in the process	8, Table 1 and protocol
Data collection process	#9	Specify the methods used to collect data from reports, including how many reviewers collected data from each report, whether they worked independently, any processes for obtaining or confirming data from study investigators, and, if applicable, details of automation tools used in the process	8-9, Table 1 and protocol
Data items	#10a	List and define all outcomes for which data were sought. Specify whether all results that were compatible with each outcome domain in each study were sought (for example, for all measures, time points, analyses), and, if not, the methods used to decide which results to collect	n/a

For peer review only

Study risk of bias assessment	#11	Specify the methods used to assess risk of bias in the included studies, including details of the tool(s) used, how many reviewers assessed each study and whether they worked independently, and, if applicable, details of automation tools used in the process	n/a
Effect measures	#12	Specify for each outcome the effect measure(s) (such as risk ratio, mean difference) used in the synthesis or presentation of results	n/a
Synthesis methods	#13a	Describe the processes used to decide which studies were eligible for each synthesis (such as tabulating the study intervention characteristics and comparing against the planned groups for each synthesis (item #5))	8-9, Table 1, protocol
Synthesis methods	#13b	Describe any methods required to prepare the data for presentation or synthesis, such as handling of missing summary statistics or data conversions	n/a
Synthesis methods	#13c	Describe any methods used to tabulate or visually display results of individual studies and syntheses	n/a
Synthesis methods	#13d	Describe any methods used to synthesise results and provide a rationale for the choice(s). If meta-analysis was performed, describe the model(s), method(s) to identify the presence and extent of statistical heterogeneity, and software package(s) used	8-9, Table 1, protocol
Synthesis methods	#13e	Describe any methods used to explore possible causes of heterogeneity among study results (such as subgroup analysis, meta-regression)	n/a

For peer review only

Synthesis methods	#13f	Describe any sensitivity analyses conducted to assess robustness of the synthesised results	n/a
Reporting bias assessment	#14	Describe any methods used to assess risk of bias due to missing results in a synthesis (arising from reporting biases)	n/a
Certainty assessment	#15	Describe any methods used to assess certainty (or confidence) in the body of evidence for an outcome	n/a
Data items	#10b	List and define all other variables for which data were sought (such as participant and intervention characteristics, funding sources). Describe any assumptions made about any missing or unclear information	n/a
**Results**			
Study selection	#16a	Describe the results of the search and selection process, from the number of records identified in the search to the number of studies included in the review, ideally using a flow diagram (http://www.prisma-statement.org/PRISMAStatement/FlowDiagram)	9, Figure 1
Study selection	#16b	Cite studies that might appear to meet the inclusion criteria, but which were excluded, and explain why they were excluded	n/a
Study characteristics	#17	Cite each included study and present its characteristics	Supplementary File 3
Risk of bias in studies	#18	Present assessments of risk of bias for each included study	n/a
Results of individual studies	#19	For all outcomes, present for each study (a) summary statistics for each group (where appropriate) and (b) an effect estimate and its	n/a

For peer review only

		precision (such as confidence/credible interval), ideally using structured tables or plots	
Results of syntheses	#20a	For each synthesis, briefly summarise the characteristics and risk of bias among contributing studies	n/a
Results of syntheses	#20b	Present results of all statistical syntheses conducted. If meta-analysis was done, present for each the summary estimate and its precision (such as confidence/credible interval) and measures of statistical heterogeneity. If comparing groups, describe the direction of the effect	n/a
Results of syntheses	#20c	Present results of all investigations of possible causes of heterogeneity among study results	n/a
Results of syntheses	#20d	Present results of all sensitivity analyses conducted to assess the robustness of the synthesised results	n/a
Risk of reporting biases in syntheses	#21	Present assessments of risk of bias due to missing results (arising from reporting biases) for each synthesis assessed	n/a
Certainty of evidence	#22	Present assessments of certainty (or confidence) in the body of evidence for each outcome assessed	n/a
**Discussion**			
Results in context	#23a	Provide a general interpretation of the results in the context of other evidence	16-18
Limitations of included studies	#23b	Discuss any limitations of the evidence included in the review	20-21
Limitations of the review methods	#23c	Discuss any limitations of the review processes used	20-21

For peer review only

Implications	#23d	Discuss implications of the results for practice, policy, and future research	19-20
**Other information**			
Registration and protocol	#24a	Provide registration information for the review, including register name and registration number, or state that the review was not registered	3
Registration and protocol	#24b	Indicate where the review protocol can be accessed, or state that a protocol was not prepared	6, 8
Registration and protocol	#24c	Describe and explain any amendments to information provided at registration or in the protocol	8-9
Support	#25	Describe sources of financial or non-financial support for the review, and the role of the funders or sponsors in the review	23
Competing interests	#26	Declare any competing interests of review authors	23
Availability of data, code, and other materials	#27	Report which of the following are publicly available and where they can be found: template data collection forms; data extracted from included studies; data used for all analyses; analytic code; any other materials used in the review	23

Notes:

- 4: 6, protocol
- 5: 8-9, protocol
- 6: 8, Supplementary File 2
- 7: Supplementary File 2
- 8: 8, Table 1 and protocol

For peer review only

- 9: 8-9, Table 1 and protocol
- 13a: 8-9, Table 1, protocol
- 13d: 8-9, Table 1, protocol
- 16a: 9, Figure 1
- 17: Supplementary File 3 The PRISMA checklist is distributed under the terms of the Creative Commons Attribution License CC-BY. This checklist was completed on 25. April 2022 using https://www.goodreports.org/, a tool made by the EQUATOR Network in collaboration with Penelope.ai

For peer review only

RAMESES checklist

List of items when reporting a realist synthesis

Reporting item	Description of item	Reported on page(s):
TITLE		
1	In the title, identify the document as a realist synthesis or review	1
ABSTRACT		
2	While acknowledging publication requirements and house style, abstracts should ideally contain brief details of: the study's background, review question or objectives; search strategy; methods of selection, appraisal, analysis and synthesis of sources; main results; and implications for practice.	2
INTRODUCTION		
3 Rationale for review	Explain why the review is needed and what it is likely to contribute to existing understanding of the topic area.	5-6
4 Objectives and focus of review	State the objective(s) of the review and/or the review question(s). Define and provide a rationale for the focus of the review.	5-6
METHODS		
5 Changes in the review process	Any changes made to the review process that was initially planned should be briefly described and justified.	9
6 Rationale for using realist synthesis	Explain why realist synthesis was considered the most appropriate method to use.	6
7 Scoping the literature	Describe and justify the initial process of exploratory scoping of the literature.	Table 1, page 8 Supplementary File 1
8 Searching processes	While considering specific requirements of the journal or other publication outlet, state and provide a rationale for how the iterative searching was done. Provide details on all the sources accessed for information in the review. Where searching in electronic databases has taken place, the details should include, for example, name of database, search terms, dates of coverage and date last searched. If individuals	Table 1, page 8 Supplementary File 2

For peer review only

		familiar with the relevant literature and/or topic area were contacted, indicate how they were identified and selected.	
9	Selection and appraisal of documents	Explain how judgements were made about including and excluding data from documents, and justify these.	Table 1, page 8
10	Data extraction	Describe and explain which data or information were extracted from the included documents and justify this selection.	Table 1, page 8
11	Analysis and synthesis processes	Describe the analysis and synthesis processes in detail. This section should include information on the constructs analyzed and describe the analytic process.	Table 1, page 9
RESULTS			
12	Document flow diagram	Provide details on the number of documents assessed for eligibility and included in the review with reasons for exclusion at each stage as well as an indication of their source of origin (for example, from searching databases, reference lists and so on). You may consider using the example templates (which are likely to need modification to suit the data) that are provided.	Figure 1, page 10
13	Document characteristics	Provide information on the characteristics of the documents included in the review.	Supplementary File 3
14	Main findings	Present the key findings with a specific focus on theory building and testing.	10-16
DISCUSSION			
15	Summary of findings	Summarize the main findings, taking into account the review's objective(s), research question(s), focus and intended audience(s).	17-19
16	Strengths, limitations and future research directions	Discuss both the strengths of the review and its limitations. These should include (but need not be restricted to) (a) consideration of all the steps in the review process and (b) comment on the overall strength of evidence supporting the explanatory insights which emerged.<br>The limitations identified may point to areas where further work is needed.	22
17	Comparison with existing literature	Where applicable, compare and contrast the review's findings with the existing literature (for example, other reviews) on the same topic.	17-18
18	Conclusion and recommendations	List the main implications of the findings and place these in the context of other relevant literature. If appropriate, offer recommendations for policy and practice.	19-23

For peer review only

19	Funding	Provide details of funding source (if any) for the review, the role played by the funder (if any) and any conflicts of interests of the reviewers.	24

For peer review only

Supplementary File 2

This file provides the full details of the search strategies employed in the review to identify documents containing relevant data.

Searches to locate existing theories (Step 1)

We searched PubMed and Web of Science (Core Collection) using a combination of terms relating to HCs, and terms relating to theory, adopting a slightly modified version of Booth and Carroll's (2015) 'BeHEMoTh' approach. As the HC programme is a specific health context (H) and we were interested in theorisation of all steps in the programme, no individual behaviours (Be) were specified.

PubMed (11th October 2020)

1. "Health Check" OR "Health Checks" (3847)
2. UK OR United Kingdom OR England OR Britain OR British (1790010)
3. model OR models OR modelling OR theor* OR concept* OR framework* (4625627)
4. 1 AND 2 AND 3 (130 hits)

Web of Science (SCI-EXPANDED, SSCI, AHCI, ESCI) (11th October 2020)

1. "Health Check" OR "Health Checks" (Topic) (3474)
2. UK OR "United Kingdom" OR England OR Britain OR British (Topic) (623802)
3. Model OR models OR modelling OR theor* OR concept* OR framework* (Topic) (11284654)
4. 1 AND 2 AND 3 (70 hits)

These searches identified 11 studies utilising 6 theoretical models or frameworks. An additional search for each of these named theories, plus terms relating to the NHSHC, was conducted in Google Scholar. In addition, we also searched Google Scholar for papers relating to the NHSHC that also cited the key citations related to each identified theory. These two steps were conducted in October 2020 and identified a further 8 (5+3) papers.

Searches for evidence on NHSHC (Step 2)

MEDLINE (via Ovid, 11th November 2020)

1. health check*.ti,ab,kw (6084)
2. (NHS or National Health Service or United Kingdom or UK or England or English).ti,ab,kw (370078)
3. exp England/ (107308)
4. 2 or 3 (440075)
5. 1 and 4 (468)

For peer review only

6. limit 5 to (english language and yr=”2008-Current”) (325)

Embase (via Ovid, 11th November 2020)

1. health check*.ti,ab,kw (8572)
2. (NHS or National Health Service or United Kingdom or UK or England or English).ti,ab,kw (487112)
3. england/ (24573)
4. 2 or 3 (494513)
5. 1 and 4 (521)
6. limit 5 to (english language and yr=”2008-Current”) (459)

CINAHL (via EbscoHost, 11th November 2020)

1. TX “health check* (2391)
2. TI (NHS OR “National Health Service” OR “United Kingdom” OR UK OR England OR English) OR AB (NHS OR “National Health Service” OR “United Kingdom” OR UK or England OR English) (178298)
3. MH “England” (62863)
4. S2 OR S3
5. S1 AND S4
6. Limiters: Published Date: 20080101-20201231; English Language; Expanders: Apply equivalent subjects (378)

HMIC (via Ovid, 11th November 2020)

1. health check*.mp (598)
2. (NHS or National Health Service or United Kingdom or UK or England or English).mp (118285)
3. exp england/ (26501)
4. exp health authorities in england/ (6680)
5. or/2-4 (135421)
6. 1 and 5 (321)
7. limit 6 to (english language and yr=”2008-Current”) (191)

Web of Science (SCI-EXPANDED, SSCI; 11th November 2020)

1. TOPIC: (“health check*”) (5144)
2. TOPIC: (NHS OR “national health service” OR “united kingdom” OR UK OR England OR English) (522053)
3. #1 AND #2 (358)

For peer review only

4. Refined by: PUBLICATION YEARS: (2020 OR 2012 OR 2019 OR 2011 OR 2018 OR 2010 OR 2017 OR 2009 OR 2016 OR 2008 OR 2015 OR 2014 OR 2013) AND LANGUAGES: (ENGLISH) (309)

Additional searches for theory

A short series of highly focused searches for documents describing street-level bureaucrats or street-level bureaucracy were run in Google Scholar in October and November 2021. The details of these searches and results screened (on screen) are provided below.

Google Scholar, 6th October 2021

("street level bureaucracy" OR "street level bureaucrats") AND ("public health") (8,160)

("street level bureaucracy" OR "street level bureaucrats") AND ("primary care") (2090)

("street level bureaucracy" OR "street level bureaucrats") AND ("general practice" OR “general practices”) (889)

("street level bureaucracy" OR "street level bureaucrats") AND ("local authority" OR “local authorities”) (6320)

For peer review only

Supplementary File 3

This file provides the full details of the documents included in the review.

First author	Year	Document type	Aim or purpose of document	Study design (if applicable)	Perspective(s)	Local area(s)	Commis-sioning period[a]	Contributed data to CMOCs			Total contrib-utions to CMOCs	Source
								C-CMOCs	P-CMOCs	A-CMOCs		
Alageel(1)	2018 (1)	Conference presentation	Summary of results of qualitative interview study (Alageel 2018)	Qualitative interview study	Providers	Lambeth and Lewisham	LA		P7		1	NHSHC website
Alageel(2)	2018 (2)	Journal article	To identify barriers and facilitators to implementing multiple health behaviour change interventions for CVD risk reduction in primary care	Qualitative interview study	Providers	Lambeth and Lewisham	LA	C14	P1, P2, P6, P7, P8, P10, P11, P13, P15, P20, P22, P24, P27	A1, A2, A27, A30, A32, A35, A38, A39	22	Existing review
Alageel(3)	2020	Journal article	To examine factors that might influence engagement and adherence to lifestyle change interventions and medication amongst people recently	Qualitative interview study	Attendees	Lambeth and Lewisham	LA	C14	P23	A2, A6, A10, A12, A15, A25, A28, A30, A31, A32, A35, A36, A38, A43, A44	17	Existing review

For peer review only

			assessed at medium or high risk of CVD									
Alford(4)	2010	Evaluation report	To explore behaviour changes made following community based NHSHC and experience of NHSHCs	Qualitative interview study	Attendees	Knowsley	PCT			A40, A44	2	Existing review
Al-Osaimi(5)	2020	Conference abstract	To describe the implementation of software to manage the NHSHC	n/a	Commissioners	Sandwell	LA	C2, C7			2	NHSHC website
Artac(6)	2013	Journal article	To assess whether the NHSHC was associated with a reduction in CVD risk after one year	Pre/post	Attendees	Hammersmith and Fulham	PCT		P12		1	Existing review
Atkins(7)	2020	Journal article	To identify behaviours and actors relevant to uptake, delivery and follow up of NHSHCs and influences on those behaviours	Systematic review	n/a	n/a	n/a	C14		A35	2	Searches - MEDLINE

For peer review only

Baker(8)	2014 (1)	Evaluation report	To assess the impact of the NHSHC programme and inform future commissioning (summary)	Cross-sectional	Attendees	Gloucestershire	PCT	C9	P12, P17		3	NHSHC website
Baker(9)	2014 (2)	Journal article	To investigate the perceptions and opinions of patients who attended an NHSHC	Survey	Attendees	Gloucestershire	PCT	C14	P1, P8, P20, P25	A1, A15, A18, A22, A23, A26, A34	12	Existing review
Baker(10)	2015 (1)	Journal article	To investigate health professionals' experiences and perspectives of implementation of the NHSHC	Survey including qualitative analysis of free text responses	Providers	Gloucestershire	PCT	C2	P10, P11, P18		4	Existing review
Baker(11)	2015 (2)	Journal article	To investigate how the local NHSHC pathway was followed and interpreted	Cross-sectional	n/a	Gloucestershire	PCT		P2, P12		2	Existing review
Bell(12)	2019	Conference abstract	To describe the outcomes of NHSHCs delivered in an integrated model with lifestyle services	Cross-sectional	Commissioners	Medway	LA	C3	P19		2	NHSHC website

For peer review only

Boase(13)	2012	Journal article	To explore the perspectives of practice nurses in their role of communicating CVD risk to patients	Qualitative interview study	Providers	Cambridge	PCT		P4, P22, P27	A8, A19	5	Searches - MEDLINE
Boseley(14)	2020	Conference presentation	To describe a local model of NHSHC delivery with integrated lifestyle services	Mixed methods	Commissioners	East Sussex	LA	C3, C6, C7	P14, P15, P19	A4	7	NHSHC website
Brutus(15)	2013	Evaluation report	To review the design of the local NHSHC programme and analyse delivery options available	Cross-sectional	Various	Croydon	PCT	C1, C2, C7	P1, P13, P15, P19	A6, A40	9	NHSHC website
Burgess(16)	2016	Conference presentation	Summary results of a qualitative interview study (Alageel 2020)	Qualitative interview study	Attendees	Lambeth and Lewisham	LA		P1, P23	A2, A5, A10, A20, A23, A28, A33, A42	10	NHSHC website
Burke(17)	2015	Conference presentation	To describe local delivery of NHSHCs with a focus on vulnerable groups	n/a	Commissioners	Leeds	LA			A5, A38	2	NHSHC website
Carter(18)	2016	Journal article	To evaluate local outcomes of NHSHCs with a focus on diagnosis and management after checks	Retrospective cohort	Attendees	Leicester	PCT, Transition	C1			1	Existing review

For peer review only

Centre for Public Scrutiny(19)	2014	Other report	A report on the role of Council Scrutiny in local reviews of NHSHC programmes	n/a	n/a	Various	LA	C1, C11	P1		3	Searches - HMIC
Chatterjee(20)	2017	Journal article	To assess the knowledge, use, and confidence in national physical activity and Chief Medical Officer guidelines and tools among GPs in England	Survey	Providers	England	LA		P21		1	Searches - MEDLINE
Chipchase(21)	2011	Evaluation report	To explore people's views and experiences of the NHSHC	Qualitative interview study	Various	Birmingham	PCT		P1, P14	A1, A2, A11, A12, A19, A20, A40	9	Existing review
Clarke(22)	2020	Conference presentation	To describe the of data to inform local CVD prevention work	n/a	Commissioners	Barnsley	LA	C8		A33	2	NHSHC website
Cochrane(23)	2012 (1)	Journal article	To compare changes in population CVD risk factors between those who receive an NHSHC and those who receive an NHSHC with additional	RCT	n/a	Stoke on Trent	PCT		P26	A9	2	Existing review

For peer review only - http://bmjopen.bmj.com/site/about/guidelines.xhtml

			lifestyle support									
Cochrane(24)	2013	Journal article	To review response, attendance and treatment uptake following NHSHCs	Cross-sectional	n/a	Stoke on Trent	PCT			A9	1	Existing review
Coffey(25)	2014	Evaluation report	To assess the level of uptake of NHSHCs	Cross-sectional	n/a	Salford	PCT		P12, P20		2	Existing review
Coghill(26)	2018	Journal article	To assess uptake and outcomes of NHSHCs in general practice	Cross-sectional	n/a	Bristol	PCT, Transition, LA		P12, P16		2	Existing review
Collins(27)	2011	Evaluation report	To evaluate the effectiveness of an 'outreach' delivery model for NHSHCs	Mixed methods	n/a	Medway	PCT		P17	A4	2	NHSHC website
Corlett(28)	2016	Journal article	To assess the findings of NHSHCs delivered in pharmacy settings, referrals to GPs and client views	Mixed methods	n/a	Lewisham	Transition, LA			A2, A13, A15, A25, A35, A38	6	Existing review
Coward(29)	2020	Conference presentation	To describe processes linking NHSHCs with	n/a	n/a	Dorset	LA	C2, C7, C8	P17, P19, P26, P27, P28		8	NHSHC website

For peer review only

			local services that aim to promote physical activity									
Cupit(30)	2018	PhD thesis	To explore how patients' and healthcare professionals' knowledge and practices about CVD prevention are socially organised	Ethnography	n/a	Unknown	LA	C8, C13, C14	P2, P4, P9, P10, P20, P24	A11, A23, A25, A27, A31, A35, A38, A44	17	NHSHC website
Dalton(31)	2011	Journal article	To examine the uptake of NHSHCs and statin prescribing	Cross-sectional	n/a	Ealing	PCT		P2, P3		2	Existing review
Derbyshire County Council(32)	2015	Evaluation report (Health Equity Audit)	To identify health inequalities in relation to the NHSHC programme	Cross-sectional	Commissioner	Derbyshire	LA	C9, C10	P2, P12, P15, P16, P17, P20, P22, P25	A35	11	NHSHC website
Edmans(33)	2013	Other report	To describe local training provision in relation to the dementia component of NHSHCs	n/a	Providers	Southwark	LA		P20		1	NHSHC website
Fenton(34)	2018	Conference presentation	To describe local CVD prevention work and NHSHCs	Cross-sectional	n/a	Southwark	LA		P1, P3		2	NHSHC website
Forster(35)	2015	Journal article	To evaluate the 'yield' of NHSHCs (in relation to identification of	Retrospective cohort	n/a	England	PCT, Transition, LA		P2, P6, P12, P16		4	Existing review

For peer review only

			risk factors, diagnoses and prescribing)									
Forsyth(36)	2012	Conference abstract	To test a delivery model using pharmacists to deliver health checks to the South Asian community (NB describes checks delivered in Scotland)	Mixed methods	n/a	Glasgow	PCT			A17	1	Searches - Embase
Frazer(37)	2020	Conference presentation	To describe the delivery of NHSHCs with integrated lifestyle services	n/a	Commissioners	West Sussex	LA	C3			1	NHSHC website
Gidlow(38)	2020	Journal article	To examine the content of NHSHC, patient-practitioner communication balance and differences when using QRISK2 versus JBS3 CVD risk calculators	Observation (video recorded NHSHCs)	n/a	West Midlands	LA		P10, P20	A6, A13, A39	5	Searches - MEDLINE
Gidlow(39)	2021 (1)	Research report	To explore the extent of physical activity and alcohol measurement in the NHSHC	Observation (video recorded NHSHCs)	n/a	West Midlands	LA		P20		1	NHSHC website

For peer review only

Gidlow(40)	2021 (2)	Research report	To explore practitioner and patient understanding of CVD risk, associated advice or treatment and the response of patients to the NHSHC supported by the QRISK2 or JBS3 risk calculators	Mixed methods	n/a	West Midlands	LA		P10	A9, A10, A13, A29	4	Search alerts
Graley(41)	2011	Journal article	To describe a 'postcode lottery' effect in relation to NHSHCs	Cross-sectional	Commissioner	North West London	PCT	C9, C10, C13, C14	P12, P13		6	Existing review
Greaves(42)	2015	Journal article	To assess the feasibility of delivering an intervention to promote healthy eating and physical activity and of conducting a full scale RCT	Pilot RCT	n/a	Bath	PCT		P28	A30, A37, A40	4	Searches - MEDLINE
Green(43)	2018	Evaluation report	To understand the NHSHC patient journey from invitation through to behaviour change or clinical intervention	Mixed methods	Attendees	East Sussex	LA	C7	P10, P24	A1, A8, A12, A24, A29, A35	9	Stakeholder group
Gregory(44)	2018	Conference presentation	To describe the role of healthcare professionals	n/a	n/a	England	LA		P25		1	NHSHC website

For peer review only

			in treatment of tobacco dependency									
Gulliford(45)	2018	Journal article	To compare CVD risk scores for 'invited' and 'opportunistic' NHSHCs	Retrospective cohort	n/a	Lambeth and Lewisham	Transition, LA		P11		1	Existing review
Hardman(46)	2014	Evaluation report	To describe local delivery of an NHSHC programme with Health Trainers	n/a	Various	Bolton	PCT	C3	P7, P14, P19	A13, A30	6	NHSHC website
Haringay Council(47)	2012	Evaluation report	Scrutiny review' focused on men's health needs and life expectancy gap	Mixed methods	n/a	Haringay	PCT	C7		A10, A13, A14, A16, A30, A37	7	NHSHC website
Hawking(48)	2018	Conference poster	To describe a visual risk communication tool designed for use in NHSHCs	n/a	Providers, attendees	Newham	LA			A44	1	NHSHC website
Hawking(49)	2019	Journal article	To explore patient perspectives and experiences of a personalised risk report during an NHSHC	Qualitative interview study	Attendees	Newham	LA		P28	A1, A9, A10, A18, A19, A20, A40, A44	9	Existing review
Hinde(50)	2017	Journal article	To assess the cost-effectiveness of NHSHCs	Economic evaluation	n/a	England	LA	C12, C13			2	Existing review

For peer review only

Homer(51)	2015	Journal article	To determine the characteristics of patients prescribed statins for primary prevention according to their CVD risk	Cross-sectional	n/a	Newham, City & Hackney, Tower Hamlets (London)	LA		P3		1	Serendipity
Honey(52)	2013	Journal article	To identify the attitudes of primary healthcare professionals towards the delivery of lifestyle advice in NHSHCs	Q methodology	Providers	Leeds	PCT		P2, P6, P7, P18, P22, P24, P27		7	Searches - MEDLINE
Honey(53)	2015	Journal article	To examine the perspectives of patients identified as being at 'high risk' of CVD events	Qualitative interview study	Attendees	Leeds	PCT		P27	A2, A3, A4, A5, A7, A9, A10, A13, A19, A20, A22, A27, A29	14	Searches - MEDLINE
Hooper(54)	2014	Journal article	To estimate the case detection of five health conditions by NHSHCs	Cross-sectional	Attendees	Warwickshire	PCT	C10			1	Existing review
Hyseni(55)	2020	Journal article	To report the results of a workshop that aimed to facilitate engagement with stakeholders who will be involved in co-	Qualitative interview study	Commissioners, Providers, Policymakers	Various	LA	C5, C7, C11, C12, C13, C14	P12		7	Searches - MEDLINE

For peer review only

			production of an NHSHC modelling tool for commissioners									
Ismail(56)	2015	Journal article	To explore the challenges and barriers faced by staff involved in the delivery of the NHSHC	Qualitative interview study	Providers	Leeds	LA	C1, C7, C9, C14	P6, P10, P12	A35, A36, A44	10	Existing review
Ismail(57)	2016	Journal article	To provide an insight into the process of patients receiving NHSHCs and determine the extent to which they were supported to reduce CVD risk through behaviour change	Qualitative interview study	Attendees	Leeds	PCT		P2, P27	A2, A6, A8, A11, A12, A30, A31, A38	10	Existing review
Ismail(58)	2019	Research report (NIHR)	To test the clinical and cost-effectiveness of an 'enhanced lifestyle motivational interviewing' intervention for patients at high risk of CVD in group and individual	RCT	n/a	South London	LA		P6, P7	A6, A16, A17, A26, A27, A30, A31, A32, A34, A40, A44	13	Searches - WoS

For peer review only

			settings, compared with usual care									
Jones(59)	2020	Conference poster	To describe local CVD prevention work and NHSHCs	n/a	n/a	Telford	LA / CCG	C1	P6, P17	A14	4	NHSHC website
Kearney(60)	2015	Conference presentation	To describe factors that influence GP engagement with NHSHCs and potential solutions	n/a	n/a	n/a	LA	C4	P1		2	NHSHC website
Kennedy(61)	2019	Journal article	To evaluate uptake, risk factor detection and management from the NHSHC	Quasi-RCT	n/a	Hampshire	PCT, Transition, LA		P13		1	Existing review
Kirkpatrick(62)	2016	Conference presentation	To describe staff development and training for NHSHC providers	n/a	Providers	Salford	LA		P8		1	NHSHC website
Krska(63)	2010	Evaluation report	To evaluate a CVD screening service offered in pharmacy settings and obtain views of prospective users about	Mixed methods	n/a	Sefton	PCT		P25	A2	2	NHSHC website

For peer review only

			the acceptability of the service									
Krska(64)	2013	Conference abstract	To compare the views of the general public, pharmacists and patients invited to the NHSHC and to compare experiences of NHSHCs delivered by pharmacies and general practices	Survey and qualitative interviews	Providers, attendees	Sefton	PCT			A14	1	Searches - Embase
Krska(65)	2014	Journal article	To explore the views and experiences of patients with potentially high-CVD risk	Survey	Attendees	Sefton	PCT		P7, P10	A15	3	Existing review
Krska(66)	2016 (1)	Journal article	To seek the views of GPs and practice managers on NHSHC implementatio n	Survey	Providers	Sefton	PCT	C9	P10, P13		3	Existing review
Krska(67)	2016 (2)	Journal article	To evaluate NHSHC implementatio n in relation to data recording, advice provided, referrals,	Cross-sectional	Providers	Sefton	PCT	C10			1	Existing review

For peer review only

			prescribing and new diagnoses									
Kumar(68)	2015	Conference presentation	To describe work to improve GP engagement with NHSHCs	n/a	n/a	Stoke on Trent, Havering (London)	LA	C1			1	NHSHC website
Lake(69)	2010	Conference presentation	To describe a community based NHSHC service	n/a	n/a	Camden	PCT	C11	P19, P28	A5, A14	4	NHSHC website
Lambert(70)	2016	Journal article	To assess indicators of programme reach available to local service commissioners	Assessment of indicators	n/a	NE England	PCT, Transition		P12		1	Existing review
Lennon(71)	2020	Conference presentation	To describe local responses to Covid-19 in relation to NHSHCs and social prescribing	n/a	n/a	Redbridge	LA	C6			1	Stakeholde r group
Liverpool City Council(72)	2020	Conference poster	To describe the local Health Trainer service available to NHSHC attendees	n/a	n/a	Liverpool	LA		P6, P8, P19, P26, P27, P28	A6, A14, A16, A30, A36, A40	12	NHSHC website
Local Government Association(73)	2015	Other report	Report on the transfer of public health responsibilities to LAs	n/a	Commissioner s	Various	LA	C1, C5, C8, C11, C12, C13			6	Existing review

For peer review only

Local Authority A(74)[, b]	2019	Evaluation report	Evaluation of local NHSHC programme	Mixed methods	Commissioners	[Redacted]	LA	C1, C2, C11	P16, P17	A15	6	Survey respondent
Local Authority B(75)[, b]	2019	Evaluation report	Evaluation of local NHSHC programme	Mixed methods	Commissioners	[Redacted]	LA	C7, C11, C12			3	Survey respondent
Local Authority C(76)[, b]	nd	Working document	Report describing quality assurance considerations in relation to NHSHCs	n/a	Commissioners	[Redacted]	LA	C1, C2, C4, C7	P9		5	Survey respondent
Local Authority C(77)[, b]	2019	Evaluation report	To measure, monitor and report on performance and quality of NHSHCs against a quality framework	Mixed methods	Commissioners	[Redacted]	LA	C2, C7, C10, C12			4	Survey respondent
London Borough of Bromley(78)	2018	Conference presentation	To assess the prevention of diabetes through NHSHCs	Cross-sectional	n/a	Bromley	LA	C14	P8, P25		3	NHSHC website
Loo(79)	2011	Conference abstract	To derive information on community pharmacists' activities and attitudes in relation to NHSHCs	Survey	Providers	Various	PCT		P13		1	Existing review
Lumley(80)	2015	Conference presentation	To describe the role of the NHSHC in addressing high blood pressure	n/a	Commissioners, providers	Blackpool	LA	C1, C7			2	NHSHC website

For peer review only - http://bmjopen.bmj.com/site/about/guidelines.xhtml

Maddern(81)	2020	Conference abstract	To describe the role of local political scrutiny in developing and improving NHSHCs	n/a	Commissioners	Wiltshire	LA	C11			1	NHSHC website
Martin(82)	2011	Practitioner article	To describe challenges in delivery of NHSHCs	n/a	Provider	East Sussex	PCT	C13	P14		2	Searches - CINAHL
McDermott(83)	2015	Conference presentation	Summary of research exploring the implementation of NHSHCs in primary care settings	Qualitative interview study	Providers	Lambeth and Lewisham	PCT, Transition	C9	P1, P10, P13		4	NHSHC website
McMillan(84)	2018	Journal article	Report on a workshop with members of the public to inform design of a 'digital adjunct' intervention for the NHSHC	Co-production	Attendees	Manchester	LA			A6, A44	2	Searches - MEDLINE
McNaughton (85)	2011	Journal article	To evaluate delivery of the NHSHC in community pharmacies	Qualitative interview study	Providers	Tees Valley	PCT	C13	P20, P27	A14, A15	5	Existing review
McNaughton (86)	2014	Journal article	To understand factors that influenced adherence to medication and advice in 'high risk' patients identified by the NHSHC	Qualitative interview study	Attendees	North East England	PCT			A3, A9, A10, A25, A27, A28, A35, A38, A40, A42	10	Existing review

For peer review only

Mills(87)	2017	Journal article	To synthesise data concerning the views of commissioners, managers and healthcare professionals towards the NHSHC	Systematic review	Commissioners, providers	n/a	n/a		P10, P13		2	Searches - MEDLINE
Nahar(88)	2020	Journal article	Protocol for a mixed methods implementation study of a community based CVD risk assessment and coaching intervention	Mixed methods (protocol)	n/a	Sussex, Nottingham	LA			A14	1	Searches - MEDLINE
Nasir(89)	2018	Conference abstract	To describe a local project that aimed to increase NHSHC uptake and referrals to local lifestyle services	Cross-sectional	n/a	Kingston (London)	LA			A35, A37	2	NHSHC website
Newman(90)	2018	Conference presentation	To describe Health Coaching techniques	n/a	n/a	n/a	LA		P27		1	NHSHC website
NHS Greenwich(91)	2011	Evaluation report	To assess the outcomes of a community-based NHSHC delivery model	Mixed methods	n/a	Greenwich	PCT		P14	A4, A13, A17	4	Existing review
NHS Midlands and East(92)	2011	Evaluation report	To evaluate a pilot programme aiming to	Mixed methods	n/a	Bedfordshire; Great Yarmouth	PCT	C2, C7	P1, P7, P10, P17, P19	A40	8	NHSHC website

For peer review only

			deliver lifestyle interventions through the NHSHC			and Waveney						
NICE(93)	2014	Guidance	Guidance for local authorities on commissioning and delivery of the NHSHC	n/a	Commissioners	England	LA	C11			1	Searches - HMIC
Nicholas(94)	2012	Journal article	To evaluate the organisation of the NHSHC in general practices	Survey	Providers	Lambeth and Lewisham	PCT		P2		1	Existing review
O'Flaherty(95)	2021	Research report	To develop a model for commissioners to quantify cost-effectiveness and potential for equitable population health again of the NHSHC	Mixed methods	Commissioners	Various	LA	C7			1	Search alerts
Onyia(96)	2016	Conference presentation	To describe the impact of Health Trainers on uptake of lifestyle services after NHSHCs	Cross-sectional	n/a	Halton	LA		P6, P8, P25, P26		4	NHSHC website
Oswald(97)	2010	Evaluation report	To evaluate the implementation ad outcomes of the 'Tees Vascular Assessment Programme'	Mixed methods	n/a	Tees Valley	PCT	C8, C9, C13, C14	P1, P2, P7, P11, P14, P28	A1, A2, A4, A9, A13, A10, A18, A28, A29, A31, A32, A33, A40, A41, A42, A43	26	Existing review

For peer review only

			(precursor to NHSHC)									
Palladino(98)	2020	Journal article	To assess associations between coverage of the NHSHC and detection and management of incident cases of non-diabetic hyperglycaemia and type 2 diabetes	Retrospective cohort	n/a	England	PCT		P5		1	Searches - MEDLINE
Patel(99)	2020	Journal article	To describe the uptake and outputs of the NHSHC programme	Cross-sectional	n/a	England	Transition, LA		P3, P12		2	Searches - MEDLINE
Paxton(100)	2020	Journal article	To assess the fidelity of delivery of NHSHCs in general practice	Fidelity assessment	n/a	East of England	PCT		P20, P21		2	Searches - MEDLINE
Perkins(101)	2020	Conference presentation	Presentation describing CVD prevention initiatives	n/a	n/a	England	LA			A2, A38	2	NHSHC website
Perry(102)	2014	Journal article	To explore experiences of engaging with a community-based NHSHC	Qualitative interview study	Attendees	Knowsley	PCT		P14, P28	A2, A4, A7, A13, A18, A25, A35, A38, A40, A44	12	Existing review

For peer review only

Public Health England(103)	2020	Guidance	Best Practice Guidance for commissioners and providers	n/a	n/a	England	LA		P12	A12	2	NHSHC website
Rawlinson(104)	2019	Journal article	To evaluate an 'enhanced health promotion service' in a physiotherapy-led musculoskeletal service providing NHSHCs and diabetes checks	Mixed methods	n/a	Salford	LA	C6	P6, P7, P8, P14, P17, P19, P26		8	Searches - CINAHL
Research Works(105)	2013	Evaluation report	To assess commissioners' and providers' experiences of the NHSHC and gain an understanding of the engagement of public health professionals with NHSHCs	Qualitative interview study	Commissioners, providers	Various	PCT, Transition	C1, C4, C7, C11, C13	P7, P9, P10, P13, P14, P19	A13	12	Existing review
Richardson(106)	2016	News article	Article responding to research demonstrating lower coverage of NHSHCs and limited evidence of effectiveness of NHSHCs	n/a	n/a	n/a	LA		P18	A16	2	Searches - CINAHL

For peer review only

Riley(107)	2015	Journal article	To examine the feasibility and acceptability of community outreach NHSHCs targeted at the Afro-Caribbean community	Ethnography	Providers, attendees	Bristol	LA		P10	A2, A3, A13, A14, A16, A17, A21, A23, A25, A26, A40, A44	13	Existing review
Riley(108)	2016	Journal article	To examine the experiences of patients attending and healthcare professionals conducting NHSHCs	Qualitative interview study	Providers, attendees	Bristol	LA		P4, P8, P10, P27	A1, A3, A11, A19, A20, A21, A22, A25, A33, A35, A38, A44	16	Existing review
Robson(109)	2015	Journal article	To describe implementation and results of the NHSHCs	Cross-sectional	n/a	City & Hackney, Newham, Tower Hamlets	PCT		P9		1	Existing review
Robson(110)	2017	Journal article	To describe the coverage and impact of NHSHCs on cardiovascular risk management and identification of new comorbidities	Retrospective cohort	n/a	City & Hackney, Newham, Tower Hamlets	PCT, Transition, LA		P9		1	Existing review

For peer review only

Saramunee(111)	2015	Journal article	To explore the experience of and willingness to use seven pharmacy public health services related to cardiovascular risk among the general public in England (including the NHSHC)	Mixed methods	Attendees	Sefton				A15, A29	2	Searches - MEDLINE
Shaw(112)	2015	Journal article	To explore health care professionals' and patients' experiences of delivering and receiving the NHSHC	Qualitative interview study	Providers, attendees	Birmingham	PCT	C14	P8, P12, P23, P24, P27	A2, A3, A4, A6, A9, A24, A26, A32, A35, A36, A38, A40	18	Existing review
Shaw(113)	2016	Journal article	To evaluate the implementation of the NHSHC from the perspective of GPs	Qualitative interview study	Providers	Birmingham	PCT		P1, P5, P6, P9, P10, P11, P13, P17, P20, P21, P26, P27	A1, A6, A10, A12, A20, A30, A39	19	Existing review
Simon(114)	2020	Conference presentation	Presentation describing physical activity interventions for prevention and management of health conditions	n/a	n/a	England	LA		P26		1	NHSHC website

Solutions Strategy(115)	2017	Evaluation report	To evaluate the pilot and assess the feasibility of extending the NHSHC to include a dementia risk reduction component	Mixed methods	n/a	Birmingham, Bury, Manchester, Southampton			P26	A13, A15, A26	4	Searches - HMIC
Strutt(116)	2011	PhD thesis	To investigate patients' experiences of and responses to NHSHCs	Qualitative interview study / ethnography	Attendees	n/a	PCT		P1, P16	A2, A5, A6, A7, A11, A13, A15, A20, A21, A22, A25, A26, A29, A30, A34, A35, A44	19	Existing review
Tanner(117)	2020	Research report	To update PHE-commissioned rapid evidence synthesis and summarise evidence to address several research questions relating to the implementation of NHSHCs	Systematic review	Various	n/a	n/a		P2		1	NHSHC website
Thompson(118)	2016	Conference presentation	Presentation describing PHE's 'StARS' framework for assessment of NHSHCs	n/a	Commissioners	England	LA	C11			1	NHSHC website
Thompson(119)	2019	Conference abstract	Summary of implementation and outcomes of an integrated	Cross-sectional	Commissioner	Slough	LA	C3	P19		2	NHSHC website

For peer review only

			cardiovascular service									
Trueland(120)	2013	News article	Article based around an interview with NHSHC supporter Professor Michael Kirby	n/a	Commissioners	n/a	LA	C8			1	Searches - HMIC
Turner(121)	2013	Journal article	To evaluate variation in statin take up following risk assessment in the NHSHC	Qualitative interview study	Attendees	Nottingham	PCT			A15, A28, A41, A42, A43	5	Searches - HMIC
Usher-Smith(122)	2017	Research report	To provide a rapid synthesis of published research evidence on NHSHCs	Systematic review	n/a	n/a	n/a		P8		1	NHSHC website
Visram(123)	2012	Evaluation report	To investigate the implementation and accessibility of the 'health trainer community check' service	Mixed methods	Various	Durham	PCT, Transition	C1, C5, C9	P6, P12, P19, P27	A2, A3, A4, A5, A31, A32, A33	14	NHSHC website
Williams(124)	2018	Conference presentation	Presentation describing physical activity training and use of the GPPAQ tool to assess physical activity levels	n/a	n/a	England	LA		P21, P26		2	NHSHC website

nd=no date

For peer review only

[a] *Commissioning period here refers to whether the data included in the document relate to the period when NHSHCs were commissioned by PCTs or LAs. Documents contributing data that spans the 'transition' period (from mid-2012 to 2013) are also noted.*

[b] *Identifying details of LAs have been removed where unpublished material was shared by survey respondents*

For peer review only

Reference list (included documents)

1. Alageel S. Implementing multiple behaviour change interventions after health checks in primary care: a qualitative study. Cardiovascular Disease Prevention Conference 2018: Getting serious about prevention: reducing variation and optimizing care London, UK: NHS England; 2018.
2. Alageel S, Gulliford MC, McDermott L, Wright AJ. Implementing multiple health behaviour change interventions for cardiovascular risk reduction in primary care: a qualitative study. BMC Fam Pract. 2018;19(1):171.
3. Alageel S, Gulliford MC, Wright A, Khoshaba B, Burgess C. Engagement with advice to reduce cardiovascular risk following a health check programme: A qualitative study. Health Expect. 2020;23(1):193-201.
4. Alford S, Perry C. Knowsley at Heart community NHS health checks: Behaviour change evaluation. 2010. Accessed
5. Al-Osaimi A. Improving lifestyle support following the NHS Health Check programme; a whole system approach. Cardiovascular Disease Prevention Conference 2020: proactive, predictive, personalised Kia Oval, London: NHS England; 2020.
6. Artac M, Dalton AR, Majeed A, Car J, Millett C. Effectiveness of a national cardiovascular disease risk assessment program (NHS Health Check): results after one year. Prev Med. 2013;57(2):129-34.
7. Atkins L, Stefanidou C, Chadborn T, Thompson K, Michie S, Lorencatto F. Influences on NHS Health Check behaviours: a systematic review. BMC Public Health. 2020;20(1):1359.
8. Baker C, Loughren E, Crone D. Evaluation of the Gloucestershire NHS Health Check Programme, for the period July 2011 to July 2012. 2014. Available from: https://www.healthcheck.nhs.uk/commissioners-and-providers/evidence/local-evaluation/. Accessed
9. Baker C, Loughren EA, Crone D, Kallfa N. Patients' perceptions of a NHS Health Check in the primary care setting. Qual Prim Care. 2014;22:232-37.
10. Baker C, Loughren EA, Crone D, Kallfa N. Perceptions of health professionals involved in a NHS Health Check care pathway. Practice Nursing. 2015;26(12):608-12.
11. Baker C, Loughren EA, Crone D, Kallfa N. A process evaluation of the NHS Health Check care pathway in a primary care setting. J Public Health (Oxf). 2015;37(2):202-9.
12. Bell K. Integrating an outreach NHS Health Check Programme into a Lifestyle Interventions Hub. Cardiovascular Disease Prevention Conference 2019: Saving Hearts and Minds Together Manchester, UK2019.
13. Boase S, Mason D, Sutton S, Cohn S. Tinkering and tailoring individual consultations: how practice nurses try to make cardiovascular risk communication meaningful. J Clin Nurs. 2012;21(17-18):2590-8.
14. Boseley R. Personalised and proactive CVD Prevention: NHS Health Checks and Integrated Lifestyle services in East Sussex. Cardiovascular Disease Prevention Conference 2020: proactive, predictive, personalised Kia Oval, London: NHS England; 2020.
15. Brutus L, Fluke R. Croydon NHS Health Check Programme: Review and Options Appraisal. 2013. Available from: https://www.healthcheck.nhs.uk/commissioners-and-providers/evidence/local-evaluation/. Accessed
16. Burgess C. Engagement with advice to reduce cardiovascular risk following an NHS Health Check. NHS Health Check Conference: Getting Serious about Prevention London, UK: NHS England; 2016.

For peer review only - http://bmjopen.bmj.com/site/about/guidelines.xhtml

17. Burke D, Kirby H. Leeds patient insight and engagement from vulnerable groups: how to reduce inqualities. NHS Health Check 2015 - Improvement through collaboration 2015.
18. Carter P, Bodicoat DH, Davies MJ, Ashra NB, Riley D, Joshi N, et al. A retrospective evaluation of the NHS Health Check Programme in a multi-ethnic population. J Public Health (Oxf). 2016;38(3):534-42.
19. Centre for Public Scrutiny. Checking the nation's health: the value of council scrutiny. London: CfPS, 2014; 2014. Available from: http://www.cfps.org.uk/includes/scripts/force_download.php?file=../../domains/cfps.org.uk/local/media/downloads/CfPS_Nations_Health_final_online.pdf. Accessed
20. Chatterjee R, Chapman T, Brannan MG, Varney J. GPs' knowledge, use, and confidence in national physical activity and health guidelines and tools: a questionnaire-based survey of general practice in England. Br J Gen Pract. 2017;67(663):e668-e75.
21. Chipchase L, Hill P, Waterall J. NHS Birmingham East & North. An insight into the NHS Health Check Programme in Birmingham: Summary Report. 2011. Accessed
22. Clarke R, Brierley A. Using NHS Health Checks data to galvanise public health prevention. Cardiovascular Disease Prevention Conference 2020: proactive, predictive, personalised Kia Oval, London: NHS England; 2020.
23. Cochrane T, Davey R, Iqbal Z, Gidlow C, Kumar J, Chambers R, et al. NHS health checks through general practice: randomised trial of population cardiovascular risk reduction. BMC Public Health. 2012;12:944.
24. Cochrane T, Gidlow CJ, Kumar J, Mawby Y, Iqbal Z, Chambers RM. Cross-sectional review of the response and treatment uptake from the NHS Health Checks programme in Stoke on Trent. J Public Health (Oxf). 2013;35(1):92-8.
25. Coffey M, Cooper A, Brown T, Cook P, Clarke-Cornwell A. Vascular health checks in Salford: an exploration using FARSITE data. University of Salford,; 2014. Accessed
26. Coghill N, Garside L, Montgomery AA, Feder G, Horwood J. NHS health checks: a cross- sectional observational study on equity of uptake and outcomes. BMC Health Serv Res. 2018;18(1):238.
27. Collins K-a. Evaluation of the NHS Health Check Outreach Programme in Medway. NHS Medway; 2011. Available from: https://www.healthcheck.nhs.uk/commissioners-and-providers/evidence/local-evaluation/. Accessed
28. Corlett SA, Krska J. Evaluation of NHS Health Checks provided by community pharmacies. J Public Health (Oxf). 2016;38(4):e516-e23.
29. Coward C. Understanding and accessing the local physical activity system. Cardiovascular Disease Prevention Conference 2020: proactive, predictive, personalised Kia Oval, London: NHS England,; 2020.
30. Cupit C. An ethnographic study of cardiovascular disease prevention: the social organisation of measures, knowledge, interventions and tensions in English general practice: University of Leicester; 2018.
31. Dalton AR, Bottle A, Okoro C, Majeed A, Millett C. Uptake of the NHS Health Checks programme in a deprived, culturally diverse setting: cross-sectional study. J Public Health (Oxf). 2011;33(3):422-9.
32. Derbyshire County Council, Services PHIaK. Health Equity Audit: NHS Health Check Programme in Derbyshire County. 2015. Available from: https://www.healthcheck.nhs.uk/commissioners-and-providers/evidence/local-evaluation/. Accessed

For peer review only

33. Edmans T. Training for the dementia component of the NHS Health Check, NHS Southwark. Southwark Council,; 2013. Available from: https://www.healthcheck.nhs.uk/commissioners-and-providers/evidence/case-studies/. Accessed
34. Fenton K. Getting serious about preventing cardiovascular disease. Cardiovascular Disease Prevention Conference 2018: Getting serious about prevention: reducing variation and optimizing care London, UK2018.
35. Forster AS, Dodhia H, Booth H, Dregan A, Fuller F, Miller J, et al. Estimating the yield of NHS Health Checks in England: a population-based cohort study. J Public Health (Oxf). 2015;37(2):234-40.
36. Forsyth P, Ali S, Ameen M, Khan R, Khan F, Sheikh AR, et al. Pharmacist-led anticipatory care for the South Asian community. International Journal of Pharmacy Practice. Royal Pharmaceutical Society, RPS Annual Conference 2012. Birmingham United Kingdom.

(var.pagings). 20: Pharmaceutical Press; 2012. p. 88-9.
37. Frazer H. Implementing NHS Health Checks within a non-clinical integrated lifestyle service. Cardiovascular Disease Prevention Conference 2020: proactive, predictive, personalised Kia Oval, London: NHS England; 2020.
38. Gidlow CJ, Ellis NJ, Cowap L, Riley VA, Crone D, Cottrell E, et al. Quantitative examination of video-recorded NHS Health Checks: comparison of the use of QRISK2 versus JBS3 cardiovascular risk calculators. BMJ Open. 2020;10(9):e037790.
39. Gidlow C, Riley V. Physical activity and alcohol measurement in NHS Health Checks. Staffordshire University, Public Health England; 2021. Available from: https://www.healthcheck.nhs.uk/commissioners-and-providers/evidence/. Accessed
40. Gidlow CJ, Ellis NJ, Cowap L, Riley V, Crone D, Cottrell E, et al. Cardiovascular disease risk communication in NHS Health Checks using QRISK®2 and JBS3 risk calculators: the RICO qualitative and quantitative study. Health Technol Assess. 2021;25(50).
41. Graley C, Katherine MF, McCoy DC. Postcode Lotteries in Public Health - The NHS Health Checks Programme in North West London. BMC Public Health. 2011;11(738).
42. Greaves C, Gillison F, Stathi A, Bennett P, Reddy P, Dunbar J, et al. Waste the waist: a pilot randomised controlled trial of a primary care based intervention to support lifestyle change in people with high cardiovascular risk. Int J Behav Nutr Phys Act. 2015;12:1.
43. Green K, Forshaw A, D'Souza H, Macherianakis A, MEL Research. NHS Health Check Patient Journey Evaluation: Final Evaluation Report. East Sussex County Council Public Health, MEL Research; 2018. Accessed
44. Gregory A, Chowdary Q, Tobacco Control Programme P. The role of healthcare professionals in treating tobacco dependency: opportunities to reduce CVD for commissioners and practitioners. Cardiovascular Disease Prevention Conference 2018: Getting serious about prevention: reducing variation and optimizing care London, UK2018.
45. Gulliford MC, Khoshaba B, McDermott L, Cornelius V, Ashworth M, Fuller F, et al. Cardiovascular risk at health checks performed opportunistically or following an invitation letter. Cohort study. J Public Health (Oxf). 2018;40(2):e151-e6.
46. Hardman L. The BIG Bolton Health Check. 2014 October 2014. Available from: https://www.healthcheck.nhs.uk/commissioners-and-providers/evidence/case-studies/. Accessed
47. Haringay Council. Scrutiny Review Men's Health: Getting to the Heart of the Matter. A review by the Overview and Scrutiny Committee. 2012. Available from: https://www.healthcheck.nhs.uk/commissioners-and-providers/evidence/local-evaluation/. Accessed

For peer review only

48. Hawking M. The CVD Risk Report, an intervention to support CVD risk communication in NHS Health Checks: results from the pilot trial. Cardiovascular Disease Prevention Conference 2018: Getting serious about prevention: reducing variation and optimizing care London, UK: NHS England; 2018.
49. Hawking MKD, Timmis A, Wilkins F, Potter JL, Robson J. Improving cardiovascular disease risk communication in NHS Health Checks: a qualitative study. BMJ Open. 2019;9(8):e026058.
50. Hinde S, Bojke L, Richardson G, Retat L, Webber L. The cost-effectiveness of population Health Checks: have the NHS Health Checks been unfairly maligned? Journal of Public Health: From Theory to Practice. 2017;25(4):425-31.
51. Homer K, Boomla K, Hull S, Dostal I, Mathur R, Robson J. Statin prescribing for primary prevention of cardiovascular disease: a cross-sectional, observational study. Br J Gen Pract. 2015(e538).
52. Honey S, Bryant LD, Murray J, Hill K, House A. Differences in the perceived role of the healthcare provider in delivering vascular health checks: a Q methodology study. BMC Fam Pract. 2013;14:172.
53. Honey S, Hill K, Murray J, Craigs C, House A. Patients' responses to the communication of vascular risk in primary care: a qualitative study. Prim Health Care Res Dev. 2015;16(1):61-70.
54. Hooper J, Chohan P, Caley M. Case detection of disease by NHS Health Checks in Warwickshire, England and comparison with predicted performance. Public Health. 2014;128(5):475-7.
55. Hyseni L, Guzman-Castillo M, Kypridemos C, Collins B, Schwaller E, Capewell S, et al. Engaging with stakeholders to inform the development of a decision-support tool for the NHS health check programme: qualitative study. BMC Health Serv Res. 2020;20(1):394.
56. Ismail H, Kelly S. Lessons learned from England's Health Checks Programme: using qualitative research to identify and share best practice. BMC Fam Pract. 2015;16:144.
57. Ismail H, Atkin K. The NHS Health Check programme: insights from a qualitative study of patients. Health Expect. 2016;19(2):345-55.
58. Ismail K, Stahl D, Bayley A, Twist K, Stewart K, Ridge K, et al. Enhanced motivational interviewing for reducing weight and increasing physical activity in adults with high cardiovascular risk: the MOVE IT three-arm RCT. Health Technol Assess. 2019;23(69).
59. Jones T. Telford Healthy Hearts. Cardiovascular Disease Prevention Conference 2020: proactive, predictive, personalised Kia Oval, London2020.
60. Kearney M. Engaging with GPs to help deliver the NHS Health Checks. NHS Health Check 2015 - Improvement through collaboration 2015.
61. Kennedy O, Su F, Pears R, Walmsley E, Roderick P. Evaluating the effectiveness of the NHS Health Check programme in South England: a quasi-randomised controlled trial. BMJ Open. 2019;9(9):e029420.
62. Kirkpatrick W, Eden A. Developing a resilient community & primary care based workforce to deliver the NHS Health Check. NHS Health Check Conference: Getting Serious about Prevention London, UK: NHS England; 2016.
63. Krska J, Mackridge AJ, Taylor J. An Evaluation of the Cardiovascular Screening Service provided by community pharmacies in Sefton PCT: Final Report. Liverpool John Moores University; 2010. Available from: https://www.healthcheck.nhs.uk/commissioners-and-providers/evidence/local-evaluation/. Accessed
64. Krska J, Taylor J, Du Plessis R. Views and experiences of the NHS Health Check provided in pharmacies and medical practices. International Journal of Pharmacy Practice Health Services Research and Pharmacy Practice Conference 2013. Preston United Kingdom.

For peer review only - http://bmjopen.bmj.com/site/about/guidelines.xhtml

(var.pagings). 21: Pharmaceutical Press; 2013. p. 16.
65. Krska J, du Plessis R, Chellaswamy H. Views and experiences of the NHS Health Check provided by general medical practices: cross-sectional survey in high-risk patients. J Public Health (Oxf). 2014;37(2):210-7.
66. Krska J, du Plessis R, Chellaswamy H. Views of practice managers and general practitioners on implementing NHS Health Checks. Prim Health Care Res Dev. 2016;17(2):198-205.
67. Krska J, du Plessis R, Chellaswamy H. Implementation of NHS Health Checks in general practice: variation in delivery between practices and practitioners. Prim Health Care Res Dev. 2016;17(4):385-92.
68. Kumar J. GP Engagement "If they know you, it's harder for them to say no". NHS Health Check 2015 - Improvement through collaboration 2015.
69. Lake G. The Healthy Heart Centre: NHS Camden's approach to reducing Health Inequalities. NHS Camden; 2010. Available from: https://www.healthcheck.nhs.uk/commissioners-and-providers/evidence/local-evaluation/. Accessed
70. Lambert MF. Assessing potential local routine monitoring indicators of reach for the NHS health checks programme. Public Health. 2016;131:92-8.
71. Lennon S, London Borough of Redbridge. Supporting LTC Prevention During the COVID-19 Pandemic: Improving NHS Health Checks and Social Prescribing Initiatives (Presentation). 2020.
72. Liverpool City Council. LiveWire Liverpool Health Trainers. Cardiovascular Disease Prevention Conference 2020: proactive, predictive, personalised Kia Oval, London2020.
73. Local Government Association. Public health transformation twenty months on: adding value to tackle local health needs.; 2015. Accessed
74. Local Authority A. The [Local Authority Borough] NHS Health Checks Programme 2019 Review [Unpublished; identifying information redacted]. 2019. Accessed
75. Local Authority B. NHS Health Checks in [Local Authority Borough] [Unpublished; identifying information redacted]. 2019. Accessed
76. Local Authority C. Health Check Quality Assurance - Working Document [Unpublished; identifying information redacted]. Accessed
77. Local Authority C. NHS Health Checks [Local area] Performance and Quality Framework 2019 [Unpublished; identifying information redacted]. 2019. Accessed
78. London Borough of Bromley. Re audit of the Prevention of Diabetes through NHS Health Checks 2014-15. Public Health England,; 2018 January 2018. Available from: https://www.healthcheck.nhs.uk/commissioners-and-providers/evidence/case-studies/. Accessed
79. Loo R, Diaper C, Salami O, Kundu M, Lalkia M, Airhiavbere E, et al. The NHS Health Check: the views of community pharmacists. Int J Pharm Pract. 2011;19(13).
80. Lumley B. Tackling high blood pressure: system wide action and the role of the NHS Health Check. NHS Health Check 2015 - Improvement through collaboration 2015.
81. Maddern S. Partnership Working - the role of local political scrutiny in driving forward the NHS Health Check Programme in Wiltshire. Cardiovascular Disease Prevention Conference 2020: proactive, predictive, personalised. Kia Oval, London: NHS England; 2020.
82. Martin H. Delivering NHS health checks to all. British Journal of Healthcare Management. 2011;17(6):250-5.
83. McDermott L. Exploring the Health Check programme in Primary Care. NHS Health Check 2015 - Improvement through collaboration 2015.

For peer review only

84. McMillan B, Fox S, Lyons M, Bourke S, Mistry M, Ruddock A, et al. Using patient and public involvement to improve the research design and funding application for a project aimed at fostering a more collaborative approach to the NHS health check: the CaVIAR project (better Care Via Improved Access to Records). Research Involv Engagem. 2018;4:18.
85. McNaughton RJ, Oswald NT, Shucksmith JS, Heywood PJ, Watson PS. Making a success of providing NHS Health Checks in community pharmacies across the Tees Valley: a qualitative study. BMC Health Serv Res. 2011;11:222.
86. McNaughton RJ, Shucksmith J. Reasons for (non)compliance with intervention following identification of 'high-risk' status in the NHS Health Check programme. J Public Health (Oxf). 2014;37(2):218-25.
87. Mills K, Harte E, Martin A, MacLure C, Griffin SJ, Mant J, et al. Views of commissioners, managers and healthcare professionals on the NHS Health Check programme: a systematic review. BMJ Open. 2017;7(11):e018606.
88. Nahar P, van Marwijk H, Gibson L, Musinguzi G, Anthierens S, Ford E, et al. A protocol paper: community engagement interventions for cardiovascular disease prevention in socially disadvantaged populations in the UK: an implementation research study. Glob Health Res Policy. 2020;5:12.
89. Nasir N. Can alternate providers improve the uptake of Health Checks among high risk populations and subsequent referrals to the local lifestyle services? Getting Serious About Cardiovascular Disease Prevention 2018: Reducing Variation and Optimising Care London, UK2018.
90. Newman P. An Introduction to Health Coaching: Better Conversations, Better Health. Cardiovascular Disease Prevention Conference 2018: Getting serious about prevention: reducing variation and optimizing care London, UK2018.
91. NHS Greenwich. Evaluation of NHS Health Check Plus Community Outreach Programme in Greenwich. 2011. Accessed
92. NHS Midlands and East. A study into increasing uptake of lifestyle changes - Report. 2011. Accessed
93. NICE. Encouraging people to have NHS Health Checks and supporting them to reduce risk factors. Manchester: NICE, 2014; 2014. Available from: http://publications.nice.org.uk/encouraging-people-to-have-nhs-health-checks-and-supporting-them-to-reduce-risk-factors-lgb15. Accessed
94. Nicholas JM, Burgess C, Dodhia H, Miller J, Fuller F, Cajeat E, et al. Variations in the organization and delivery of the 'NHS health check' in primary care. J Public Health (Oxf). 2012;35(1):85-91.
95. O'Flaherty M, Lloyd-Williams F, Capewell S, Boland A, Maden M, Collins B, et al. Modelling tool to support decision-making in the NHS Health Check programme: workshops, systematic review and co-production with users. Health Technol Assess. 2021;25(35).
96. Onyia I. Delivery of the NHS Health Check by health trainers can improve conversion into uptake of lifestyle service. NHS Health Check Conference: Getting Serious about Prevention London, UK: NHS England; 2016.
97. Oswald N, McNaughton R, Watson P, Shucksmith J. Tees Vascular Assessment Programme: Evaluation commissioned by the Tees Primary Care Trusts (PCT) from the Centre for Translational Research in Public Health. 2010. Available from: https://www.healthcheck.nhs.uk/commissioners-and-providers/evidence/local-evaluation/. Accessed
98. Palladino R, Vamos EP, Chang KC, Khunti K, Majeed A, Millett C. Evaluation of the Diabetes Screening Component of a National Cardiovascular Risk Assessment Programme in England: a Retrospective Cohort Study. Sci Rep. 2020;10(1):1231.
99. Patel R, Barnard S, Thompson K, Lagord C, Clegg E, Worrall R, et al. Evaluation of the uptake and delivery of the NHS Health Check programme in England, using primary care data from 9.5 million people: a cross-sectional study. BMJ Open. 2020;10(11):e042963.
100. Paxton B, Mills K, Usher-Smith JA. Fidelity of the delivery of NHS Health Checks in general practice: an observational study. BJGP Open. 2020;4(4).

101. Perkins C. Cardiovascular disease prevention in the 2020s. Cardiovascular Disease Prevention Conference 2020: proactive, predictive, personalised Kia Oval, London2020.
102. Perry C, Thurston M, Alford S, Cushing J, Panter L. The NHS health check programme in England: a qualitative study. Health Promot Int. 2014;31(1):106-15.
103. Public Health England. NHS Health Check Best practice guidance For commissioners and providers. 2020. Available from: https://www.healthcheck.nhs.uk/commissioners-and-providers/national-guidance/. Accessed 31st October 2019.
104. Rawlinson G. Health promotion in physiotherapy services using NHS health and diabetes checks. British Journal of Healthcare Management. 2019;25(1):22-31.
105. Research Works, Public Health England. Understanding the implementation of NHS Health Checks. 2013. Accessed
106. Richardson J. The fall in NHS Health Checks. Nursing in Practice: The Journal for Today's Primary Care Nurse. 2016(91):1-3.
107. Riley R, Coghill N, Montgomery A, Feder G, Horwood J. The provision of NHS health checks in a community setting: an ethnographic account. BMC Health Serv Res. 2015;15:546.
108. Riley R, Coghill N, Montgomery A, Feder G, Horwood J. Experiences of patients and healthcare professionals of NHS cardiovascular health checks: a qualitative study. J Public Health (Oxf). 2016;38(3):543-51.
109. Robson J, Dostal I, Madurasinghe V, Sheikh A, Hull S, Boomla K, et al. The NHS Health Check programme: implementation in east London 2009-2011. BMJ Open. 2015;5(4):e007578.
110. Robson J, Dostal I, Madurasinghe V, Sheikh A, Hull S, Boomla K, et al. NHS Health Check comorbidity and management: an observational matched study in primary care. Br J Gen Pract. 2017;67(655):e86-e93.
111. Saramunee K, Krska J, Mackridge A, Richards J, Suttajit S, Phillips-Howard P. General public's views on pharmacy public health services: current situation and opportunities in the future. Public health. 2015;129(6):705-15.
112. Shaw RL, Pattison HM, Holland C, Cooke R. Be SMART: examining the experience of implementing the NHS Health Check in UK primary care. BMC Fam Pract. 2015;16:1.
113. Shaw RL, Lowe H, Holland C, Pattison H, Cooke R. GPs' perspectives on managing the NHS Health Check in primary care: a qualitative evaluation of implementation in one area of England. BMJ Open. 2016;6(7):e010951.
114. Simon C. Physical activity for prevention and management of health conditions and practitioner resources. Cardiovascular Disease Prevention Conference 2020: proactive, predictive, personalised Kia Oval, London: NHS England,; 2020.
115. Solutions Strategy Research Facilitation Ltd, Alzheimer's Society. NHS Health Check 40-64 dementia pilot research findings: summary research report. 2017.
116. Strutt E. Patient-centred care: Patients' experiences of and responses to the National Health Service (NHS) Health Check programme in general practice. Durham: Durham University; 2011.
117. Tanner L, Kenny R, Still M, Pearson F, Bhardwaj-Gosling R. NHS Health Check Programme Rapid Review Update. University of Sunderland, Newcastle University, Public Health England; 2020. Available from: https://www.healthcheck.nhs.uk/commissioners-and-providers/evidence/. Accessed 31st October 2020.

For peer review only

118. Thompson K. NHS Health Check StARS framework: A Systems Approach for Raising Standards. NHS Health Check Conference: Getting Serious about Prevention London, UK: NHS England; 2016.
119. Thompson S. Integrated Cardiac Disease Prevention Programme. Cardiovascular Disease Prevention Conference 2019: Saving Hearts and Minds Together. Manchester, UK2019.
120. Trueland J. 'The signs were there, but weren't picked up'. Health Serv J. 2013;123(6369):6-7.
121. Turner A. Exploratory evaluation of variation in statin take up among high risk patients in Nottingham City. Public Health. 2013.
122. Usher-Smith JA, Mant J, Martin A, Harte E, MacLure C, Meads C, et al. NHS Health Check Programme rapid evidence synthesis. Cambridge, UK: The Primary Care Unit, University of Cambridge, RAND Europe, Public Health England; 2017. Accessed 31st October 2020.
123. Visram S, Geddes L, Carr SM. Formative Evaluation of the Health Trainer Community Health Check Service in County Durham. Northumbria University, County Durham and Darlington NHS Foundation Trust; 2012. Available from: https://www.healthcheck.nhs.uk/commissioners-and-providers/evidence/local-evaluation/. Accessed
124. Williams Z. Let's talk about Physical Activity: Physical Activity Clinical Champions training and using the GPPAQ in a health check setting. Cardiovascular Disease Prevention Conference 2018: Getting serious about prevention: reducing variation and optimizing care London, UK2018.

For peer review only

Supplementary File 4

This file contains 3 tables outlining the full set of CMOCs (n=86) developed during the review. The three tables split the CMOCs up into those centred on commissioner (Table S1), provider (Table S2) and attendee (Table S3) perspectives.

Table S1 Overview of CMOCs focused on LA commissioners

	**CMOC**	**Summary of data**
Understanding and engagement with the NHSHC programme: case finding or enabling behaviour change?		
CMOC C1	When commissioners view the NHSHC as a means to improve people's lives through behaviour change (C) they will try to exert their influence over providers to ensure the programme is delivered with this in mind (O) because they believe this will maximise the potential benefits of the programme (M)	Data extracted from 12 documents:<br><br>2 research articles (one cohort study, one qualitative interview study); 4 local evaluation reports; 3 conference materials; 1 unpublished LA working document; 2 other reports focused on LA roles in NHSHCs.
CMOC C2	When commissioners view the NHSHC as a means to improve people's lives through behaviour change (C) they will try to establish closer working relationships between different parts of the wider system (the NHSHC, lifestyle services and primary care) to improve referral pathways (O) because they believe this will maximise the potential benefits of the programme (M)	Data extracted from 8 documents:<br><br>1 research article (survey); 4 local evaluation reports; 2 conference materials; 1 unpublished LA working document.

For peer review only

CMOC C3	When commissioners view the NHSHC as a means to improve people's lives through behaviour change (C) they may develop 'integrated' models of service delivery with the NHSHC delivered alongside lifestyle services (O) because they believe this will maximise the potential benefits of the programme (M)3,	Data extracted from 5 documents:<br><br>1 local evaluation report; 4 conference materials.
CMOC C4	When providers are sceptical and less engaged with the NHSHC programme (C) commissioners may be unable to exert their influence over them (O1) or establish close working relationships between different parts of the system (O2) because providers are resistant and unwilling to engage (M)	Data extracted from 3 documents:<br><br>1 local evaluation report; 1 conference presentation; 1 unpublished LA working document.
CMOC C5	When commissioners have a focus on the wider social determinants of health (C) they are more likely to commission 'alternative' NHSHC providers (i.e. to move away from a medical model based in primary care) (O) because they believe this will maximise the benefits of the programme (M)	Data extracted from 3 documents:<br><br>1 research article (qualitative interviews/workshop); 1 local evaluation report; 1 report focused on LA roles in NHSHCs.
CMOC C6	When commissioners have a focus on the wider social determinants of health (C) they may integrate NHSHC delivery with other services that address other problems (O) because they believe this will maximise the potential benefits of the programme (M)	Data extracted from 3 documents:<br><br>1 research article (mixed methods evaluation); 2 conference materials.

For peer review only

CMOC C7	When commissioners view the NHSHC as a means to improve people's lives through behaviour change (C) they are more likely to collect data related to what happens after the measurements and risk assessment are completed in a check (O) because they believe these are important data to monitor and evaluate programme performance (M)	Data extracted from 15 documents:<br><br>2 research articles (1 qualitative interviews/workshop, 1 qualitative interview study); 1 research report (mixed methods study); 7 local evaluation reports; 4 conference materials; 1 unpublished LA working document.
CMOC C8	When commissioners view the NHSHC as a means to improve people's lives through behaviour change (C) they are more likely to collect data related to the needs of the local population to inform the commissioning of lifestyle support services (O) because they believe this will maximise the benefits of the programme (M)	Data extracted from 6 documents:<br><br>1 PhD thesis (ethnography); 1 local evaluation report; 2 conference materials; 1 report focused on LA roles in NHSHCs; 1 news article.
CMOC C9	In some circumstances, commissioners may cede more control over delivery to primary care based providers (e.g. GP practices) (O) but the contexts in which this happens and the mechanisms underpinning this outcome are not clear (C, M not defined)	Data extracted from 8 documents:<br><br>3 research articles (1 qualitative interview study, 1 survey, 1 cross-sectional study); 4 local evaluation reports; 1 conference materials.
CMOC C10	In some circumstances, commissioners may focus only on mandatory data collection (monitoring invitation, uptake and coverage) (O) but the contexts in which this happen and the mechanisms underpinning this outcome are not clear (C, M not defined)	Data extracted from 5 documents:<br><br>3 research articles (3 cross-sectional studies); 2 local evaluation reports.
Practical constraints: politics and funding		

For peer review only

CMOC C11	Where there is local political support and engagement with the NHSHC programme (C), local delivery is more likely to be evaluated (O1) and developed or improved (O2) because commissioners and public health teams are empowered to focus on the programme (M)	Data extracted from 10 documents:<br><br>1 research article (qualitative interview study); 3 local evaluation reports; 3 conference materials; 2 other reports focused on LA roles in NHSHCs; 1 guidance document
CMOC C12	When funding for public health programmes is constrained (C1) and commissioners or public health teams are convinced of the NHSHCs long term effectiveness and value (C2) local delivery is more likely to be evaluated (O1) and developed or improved (O2) because these activities are considered worthwhile (M)	Data extracted from 5 documents:<br><br>2 research articles (1 qualitative interview study; 1 economic evaluation); 2 local evaluation reports; 1 other report focused on LA roles in NHSHCs.
CMOC C13	When funding for public health programmes is constrained (C) commissioners may select providers who offer the best value for money / lowest cost per NHSHC delivered (O) because they must prioritise mandatory public health programme delivery ('prescribed functions') before funding additional services (M)	Data extracted from 9 documents:<br><br>4 research articles (2 qualitative interview studies; 1 cross-sectional, 1 economic evaluation); 2 local evaluation reports; 1 PhD thesis (ethnography); 1 other report focused on LA roles in NHSHCs; 1 practitioner-facing article
CMOC C14	When funding for public health programmes is constrained (C) commissioners must prioritise funding mandatory services ('prescribed functions') (M) leading to cuts and reduced capacity in non-mandatory services (e.g. local lifestyle support services) (O)	Data extracted from 11 documents:<br><br>8 research articles (5 qualitative interview studies, 1 survey, 1 cross-sectional study, 1 systematic review); 1 local evaluation report; 1 PhD thesis (ethnography); 1 conference presentation.

For peer review only

Table S2 Overview of CMOCs focused on NHSHC providers

	CMOC	Summary of data
Understanding and engagement with the NHSHC programme: scepticism versus 'buy in'		
CMOC P1	When providers are sceptical about the NHSHC programme as a behaviour change intervention (C) they may prioritise completing the mandatory elements of the check and fail to engage with the delivery of advice, brief interventions or referrals (O) because they do not believe these will help attendees (M)	Data extracted from 13 documents:<br><br>3 research articles (2 qualitative interview studies, 1 survey); 3 local evaluation reports; 4 conference materials; 1 other report focused on LA roles in NHSHCs; 1 PhD thesis (ethnography).
CMOC P2	When providers are sceptical about the NHSHC programme as a behaviour change intervention (C1) or sceptical about the effectiveness of behaviour change to reduce the risk of cardiovascular disease (C2) they are more likely to consider medication (e.g. statins or antihypertensives) as an appropriate intervention for those assessed at higher risk (O) because they believe these will help attendees (M)	Data extracted from 11 documents:<br><br>7 research articles (2 qualitative interview studies; 1 survey; 2 cross-sectional studies; 1 cohort study; 1 Q-methodology study); 2 local evaluation reports; 1 research report (systematic review); 1 PhD thesis (ethnography).
CMOC P3	When providers (who are able to prescribe) are sceptical about the recommended thresholds for prescription (C) they are less likely to prescribe (O) because they do not believe it will help attendees (M).<br><br>*This CMOC may apply to other interventions but we lack data to confirm or refute this.*	Data extracted from 4 documents:<br><br>3 research articles (3 cross-sectional studies); 1 conference presentation.

For peer review only

CMOC P4	When providers are worried about labelling healthy people as sick (C) they may avoid offering advice, referrals or prescriptions (O) because they are concerned about the potential harms of overdiagnosis (M)	Data extracted from 3 documents:<br><br>2 research articles (2 qualitative interview studies); 1 PhD thesis (ethnography).
CMOC P5	When providers are highly engaged with preventive health care (C) they are more likely to be highly engaged with the NHSHC programme (O) as they understand it to be a useful means of reaching more patients with this agenda (M)	Data extracted from 2 documents:<br><br>2 research articles (1 qualitative interview study, 1 cohort).
CMOC P6	When providers believe that lifestyle modification is an effective means of reducing CVD risk (C) they are more likely to offer attendees advice, brief interventions or referrals to lifestyle support services (especially as a first line of action) (O) because they believe these may help attendees (M)	Data extracted from 11 documents:<br><br>6 research articles (2 qualitative interview studies; 1 cohort study; 1 mixed methods study; 1 Q-methodology study); 1 research report (RCT); 1 local evaluation report; 3 conference materials
CMOC P7	When providers buy in to the NHSHC as an opportunity to support behaviour change (C) they are more likely to offer attendees advice, brief interventions or referrals to lifestyle support services (O) because they believe these will help attendees (M)	Data extracted from 11 documents:<br><br>5 research articles (1 qualitative interview study; 1 survey; 1 mixed methods study; 1 Q-methodology study); 1 research report (RCT); 4 local evaluation reports; 1 conference presentation
CMOC P8	When providers have the specific skills they need to support the delivery of advice, brief interventions and referrals (C) they are more likely to engage	Data extracted from 10 documents:

For peer review only

	with and prioritise these activities (O) because they feel confident to deliver them during the NHSHC encounter (M)	3 research articles (2 qualitative interview studies, 1 mixed methods study); 1 research report (systematic review); 4 conference materials.
Practical constraints: time and money		
CMOC P9	When funding arrangements for delivery of checks incentivise volume of delivery (C) providers may prioritise completing mandatory elements of the check and minimise time spent delivering advice, brief interventions or offering referrals (O) because they are aware they do not have to complete these (M)	Data extracted from 6 documents:<br><br>3 research articles (1 qualitative interview study, 1 retrospective cohort, 1 cross-sectional); 1 local evaluation report; 1 PhD thesis (ethnography); 1 LA internal working document.
CMOC P10	When providers have many competing priorities (C) they may prioritise completing mandatory elements of the check and minimise time spent delivering advice, brief interventions or offering referrals (O) because of expediency (M)	Data extracted from 16 documents:<br><br>10 research articles (4 qualitative interview studies, 1 systematic review, 3 surveys, 1 ethnography, 1 observational study based on video recordings); 3 local evaluation reports; 1 research report (mixed methods); 1 PhD thesis (ethnography), 1 conference presentation
CMOC P11	When there is a focus on increasing the volume of checks delivered (C) providers may offer more checks 'opportunistically' (i.e. not in a standalone appointment) (O) as they feel this is more efficient (M)	Data extracted from 5 documents:<br><br>4 research articles (2 qualitative interview studies, 1 retrospective cohort, 1 survey, 1 mixed methods); 1 local evaluation report

For peer review only

CMOC P12	It is clear that data about non-mandatory elements of a check are often under-recorded or recorded inconsistently (O) but the contexts in which this happens and the mechanisms underpinning this outcome are not clear (C, M not defined). Potentially important contexts here include competing priorities, a focus on delivery of mandatory elements of checks, lack of monitoring and/or incentivisation to collect particular data items, or difficulty in recording certain activities within existing data collection systems	Data extracted from 15 documents:<br><br>10 research articles (3 qualitative interview studies, 4 cross-sectional studies, 1 pre/post study, 1 retrospective cohort, 1 study assessing the validity of indicators); 3 local evaluation reports; 1 guidance document; 1 conference abstract.
CMOC P13	When providers do not feel they are adequately compensated for delivering checks (C) they may prioritise completing mandatory elements of the check and minimise time spent delivering advice, brief interventions, or offering referrals (O) because they do not feel it is worth the cost (M)	Data extracted from 10 documents:<br><br>6 research articles (2 qualitative interview studies; 1 cross-sectional studies, 1 quasi-RCT, 1 surveys, 1 systematic review); 2 local evaluation reports; 2 conference materials
CMOC P14	When providers 'buy in' to the NHSHC programme (see CMOC P7 above) (C1) and have adequate time and/or flexibility to deliver each check (C2) they may offer more personalised and in-depth advice and support (O) because they believe these may help attendees (M)	Data extracted from 9 documents:<br><br>2 research articles (1 qualitative interview study, 1 mixed methods); 5 local evaluation reports; 1 conference presentation; 1 practitioner-facing article
Practical constraints: referrals and follow-up		
CMOC P15	When multiple modifiable risk factors are identified during a check (C1) and separate lifestyle services exist for each (C2) providers (and attendees)	Data extracted from 4 documents:

For peer review only - http://bmjopen.bmj.com/site/about/guidelines.xhtml

	may agree to prioritise addressing one risk factor first (M) so the delivery of advice, brief interventions and referrals reflect this priority (O)	1 research article (qualitative interview study); 2 local evaluation reports, 1 conference presentation
CMOC P16	When providers don't perceive available lifestyle services to be a good 'fit' for individuals (C) they may avoid making referrals to these services (O) because they do not believe it will help attendees (M)	Data extracted from 4 documents:<br><br>2 research articles (1 retrospective cohort, 1 cross-sectional study); 2 local evaluation reports; 1 PhD thesis (interviews/ethnography)
CMOC P17	When information about local lifestyle services and referral routes is disparate and difficult to access (C) it is harder for providers to make referrals (O) because providers are unaware of available services and how to refer (M)	Data extracted from 9 documents:<br><br>2 research articles (1 qualitative interview study, 1 mixed methods); 4 local evaluation reports; 3 conference materials
CMOC P18	When providers have concerns about the quality of lifestyle support services (C) they may avoid making referrals (O) because they doubt they will help attendees (M)	Data extracted from 3 documents:<br><br>2 research articles (1 survey, 1 Q-methodology study); 1 news article
CMOC P19	When providers have established relationships and referral pathways to trusted lifestyle services (C) they are more likely to offer referrals (O) because this becomes a delivery norm (M)	Data extracted from 12 documents:<br><br>1 research article (mixed methods); 5 local evaluation reports; 6 conference materials
Practical constraints: relationships and confidence in the delivery of advice		

For peer review only

CMOC P20	When providers are concerned that discussion of a particular risk factor may cause offence or upset an attendee (C) they may avoid bringing it up or discussing it in-depth (O) because they lack confidence and want to avoid confrontation (M)	Data extracted from 11 documents:<br><br>6 research articles (3 qualitative interview studies, 1 survey, 1 observational study based on video recordings, 1 fidelity assessment); 2 local evaluation reports; 1 research report (observational study); 1 PhD thesis (ethnography); 1 other report
CMOC P21	When providers lack knowledge about recommendations in relation to a particular risk factor (C) they may avoid bringing it up or discussing it in-depth during a check (O) because they lack confidence in their advice (M)	Data extracted from 4 documents:<br><br>3 research articles (1 qualitative interview study; 1 survey; 1 fidelity assessment); one conference presentation
CMOC P22	When providers perceive that an attendee is unlikely to want to, or be able to change their lifestyle (C) they may avoid giving them advice or offering referrals to support this (O) because they do not think it will help (M1) or because they are worried it could damage their relationship (M2)	Data extracted from 4 documents:<br><br>3 research articles (2 qualitative interview studies; 1 Q-methodology study); 1 local evaluation report
CMOC P23	When providers feel they themselves are not good role models for healthy lifestyles (C) they may be reluctant to deliver advice or brief interventions, or make referrals (O) because they are worried about appearing hypocritical and lacking credibility (M)	Data extracted from 2 documents:<br><br>1 research article (qualitative interview study) and 1 conference presentation

For peer review only

CMOC P24	When providers have lived experience of (trying to) make lifestyle changes (C) they may share this and empathise with attendees (O) because they want to build rapport and a therapeutic alliance during checks (M)	Data extracted from 5 documents:<br><br>3 research articles (2 qualitative interview studies, 1 Q-methodology study); 1 local evaluation report; 1 PhD thesis (ethnography)
CMOC P25	When discussing a risk factor is normalised and routine (C) providers may be more likely to deliver advice, brief interventions and offer referrals related to that risk factor (O) because they feel comfortable and practiced in doing so (M)	Data extracted from 6 documents:<br><br>1 research article (survey); 2 evaluation reports; 3 conference materials
CMOC P26	When providers receive training (C1) or have regular practice (C2) in delivering lifestyle advice, they are more likely to deliver it regularly during checks (O) because they feel more confident (M)	Data extracted from 9 documents:<br><br>3 research articles (1 qualitative interview study, 1 mixed methods, 1 RCT); 1 local evaluation reports; 5 conference materials
CMOC P27	When providers take into account attendees' own priorities, constraints and wishes during a check (C) they may adapt the advice, brief interventions or referrals offered to take these into account (e.g. make fewer but more appropriate referrals) (O) because they share the decision with attendees (M)	Data extracted from 12 documents:<br><br>8 research articles (7 qualitative interview studies, 1 Q-methodology study); 1 local evaluation report; 3 conference materials
CMOC P28	When providers are aware of attendees' own priorities, constraints and wishes (C) they may identify and emphasise the benefits of simple changes	Data extracted 7 from documents:

For peer review only

	that are more acceptable and achievable for attendees (O) because they believe this will help attendees (M)	3 research articles (2 qualitative interview studies, 1 pilot RCT); 1 local evaluation report; 3 conference materials

Table S3 Overview of CMOCs focused on NHSHC attendees

	**CMOC**	**Summary of data**
Understanding and engagement with the NHSHC programme: expectations and priorities		
CMOC A1	When attendees understand the NHSHC as a screening opportunity aimed at identifying individuals with disease (C) they may be less likely to engage with advice, brief interventions or offers of referrals (O) because what is offered does not meet their expectations (M)	Data extracted from 8 documents:<br><br>5 research articles (4 qualitative interview studies, 3 local evaluation reports)
CMOC A2	When attendees are already aware of risk factors and/or potential improvements they could make to their lifestyle (C) they may be more receptive to receiving advice, brief interventions or referrals (O) because they are mentally prepared for it (M)	Data extracted from 15 documents:<br><br>8 research articles (6 qualitative interview studies, 1 ethnography, 1 mixed methods); 4 local evaluation reports; 1 PhD (ethnography); 2 conference materials
CMOC A3	When attendees are unaware that they have risk factors for CVD and receive results that indicate that they are at high risk (C) they may need extra support and information from providers (O) because they are shocked and upset (M)	Data extracted from 6 documents:<br><br>5 research articles (4 qualitative interview studies, 1 ethnography); 1 local evaluation report

For peer review only

CMOC A4	In some circumstances attendees who receive results that indicate that they are at high risk may be prompted to make immediate lifestyle changes (O) because they are shocked and upset (M), but the contexts in which this happens (and for whom) are not clear (C undefined)	Data extracted from 8 documents:<br><br>3 research articles (3 qualitative interview studies); 4 local evaluation reports; 1 conference presentation
CMOC A5	When providers are able to explain the implications of risk factors to attendees in a way they can understand (C) attendees may be more receptive to advice, brief interventions or referrals (O) because they appreciate its importance for their own lives (M)	Data extracted from 6 documents:<br><br>1 research article (qualitative interview study); 1 local evaluation report; 1 PhD thesis (interviews/ethnography); 3 conference materials
CMOC A6	When providers are able to link advice, brief interventions and offers of referrals to attendees' own priorities for their health and lifestyle (C) attendees may be more likely to engage with these (O) because they want to achieve these (M)	Data extracted from 10 documents:<br><br>6 research articles (4 qualitative interview studies, 1 co-production study, 1 observational study using video-recordings); 1 research report (RCT); 1 local evaluation report; 1 PhD thesis (interviews/ethnography); 1 conference poster
CMOC A7	When attendees have multiple risk factors (C) they may choose to focus on the advice, brief interventions or referrals offered in relation to those lifestyle changes that are easier to change (O) because they feel it is better than nothing (M)	Data extracted from 3 documents:<br><br>2 research articles (qualitative interview studies); 1 PhD thesis (interviews/ethnography)

For peer review only

CMOC A8	When attendees feel a personal responsibility for their own health and lifestyle (C) they may be unlikely to take up offers of referrals or ongoing support or follow up (O) because they feel obliged to try to make changes on their own (M)	Data extracted from 3 documents:<br><br>2 research articles (2 qualitative interview studies); 1 local evaluation report
CMOC A9	When attendees are not motivated to change their lifestyle or behaviour (C) they are unlikely to engage with advice, brief interventions or take up offers of referrals to lifestyle services (O) because they do not believe they need to, and have other priorities (M)	Data extracted from 8 documents:<br><br>6 research articles (4 qualitative interview studies, 1 RCT, 1 cross-sectional study); 1 research report (mixed methods); 1 local evaluation report
CMOC A10	When attendees are fatalistic about their health (C) they are unlikely to engage with advice, brief interventions or take up offers of referrals to lifestyle services (O) because they think they are pointless (M)	Data extracted from 8 documents:<br><br>4 research articles (4 qualitative interview studies); 1 research report (mixed methods); 1 local evaluation report; 1 conference presentation
CMOC A11	When attendees have health concerns and priorities that fall outside the remit of the NHSHC programme (C) they may be disappointed with the check (O1) and unlikely to engage with advice, brief interventions or take up offers of referrals to lifestyle services (O2) because these do not feel important to them, and they have other priorities (M)	Data extracted from 5 documents:<br><br>2 research articles (2 qualitative interview studies); 1 local evaluation report; 2 PhD theses (ethography, interviews/ethnography).

For peer review only

CMOC A12	When attendees receive an ‘opportunistic’ check (C) they are less likely to (receive and) engage with advice, brief interventions or offers of referrals (O) because they do not understand that this is the purpose of the check (M)	Data extracted from 6 documents:<br><br>3 research articles (3 qualitative interview studies); 2 local evaluation reports; 1 guidance document
CMOC A13	When attendees have the opportunity and time to discuss and ask questions during a check (C) they may be more likely to receive and engage with advice, brief interventions and offers of referrals (O) because they understand that this is the purpose of the check (M)	Data extracted from 14 documents:<br><br>5 research articles (2 qualitative interview studies, 1 ethnography, 1 mixed methods study, 1 observational study using video-recordings); 6 local evaluation reports; 1 research report (mixed methods); 1 PhD thesis (interviews/ethnography)
CMOC A14	When checks are delivered in a non-medical setting (i.e. not in general practice) (C) attendees may be more likely to engage in discussions about risk factors and lifestyle change (O) because they feel relaxed and comfortable (M)	Data extracted from 8 documents:<br><br>3 research articles (1 qualitative interview study, 1 ethnography, 1 protocol for a mixed methods study); 1 local evaluation report; 4 conference materials
Understanding and engagement with the NHSHC programme: credibility and trust		
CMOC A15	When attendees receive advice, brief interventions, offers of referral or prescriptions from a professional they consider to be suitably qualified (C) they may be more likely to engage with or accept these interventions (O) because they consider them to be credible (M)	Data extracted from 10 documents:<br><br>7 research articles (3 qualitative interview studies, 2 surveys, 2 mixed methods studies); 2 local evaluation reports; 1 PhD thesis (interviews/ethnography).

For peer review only

CMOC A16	When attendees receive advice, brief interventions or offers of referral from a provider who seems to understand their circumstances (C) they may be more likely to engage with these interventions (O) because they consider them to be credible (M)	Data extracted from 5 documents:<br><br>1 research article (ethnography); 1 research report (RCT); 1 local evaluation report; 1 conference poster; 1 news article
CMOC A17	When providers can deliver culturally appropriate lifestyle advice (C) attendees may be more likely to engage with it (O) because they consider it to be more credible and relevant (M)	Data extracted from 4 documents:<br><br>1 research article (ethnography); 1 research report (RCT); 1 local evaluation report; 1 conference abstract
CMOC A18	When the measurements and risk assessment completed during a check identify attendees as 'low risk' (C) attendees may be less receptive to any advice, brief interventions or referrals offered (O) because they are reassured and do not think they need to take action (O)	Data extracted from 4 documents:<br><br>3 research articles (1 qualitative interview study, 1 survey, 1 mixed methods study); 1 local evaluation report
CMOC A19	When providers downplay risks or temper advice about lifestyle (C) attendees may feel there is no need to make any changes (O) because they are reassured (M)	Data extracted from 5 documents:<br><br>4 research articles (4 qualitative interview studies); 1 local evaluation report
CMOC A20	When providers are able to convey the importance and urgency of NHSHC risk assessments to attendees (C) attendees may be more receptive to advice, brief interventions or referrals (O) because they feel important (M)	Data extracted from 6 documents:<br><br>3 research articles (3 qualitative interview studies); 1 local evaluation report; 1 PhD thesis (interviews/ethnography); 1 conference presentation

For peer review only

CMOC A21	When the measurements and risk assessment completed during a check identify attendees as 'low risk' (C) attendees' healthy lifestyle habits may be reinforced (O) because they understand these have tangible benefits, reflected in their results (M)	Data extracted from 3 documents:<br><br>2 research articles (1 qualitative interview study, 1 ethnography); 1 PhD thesis (interviews/ethnography)
CMOC A22	When attendees receive measurements and risk assessment results from non-professionals (C1) or do not receive results at all (C2) they may be less likely to consider the results to be important (O) because they trust that providers will alert them to significant results that require action (M)	Data extracted from 4 documents:<br><br>3 research articles (2 qualitative interview studies, 1 survey); 1 PhD thesis (interviews/ethnography)
CMOC A23	When attendees perceive providers to be disengaged with the check (C) they may be less likely to engage with advice, brief interventions or offers of referral (O) because they do not believe these are important (M)	Data extracted from 4 documents:<br><br>2 research articles (1 ethnography, 1 survey); 1 PhD thesis (ethnography); 1 conference presentation
CMOC A24	When attendees receive advice, brief interventions or offers of referral from a provider who does not seem to 'practice what they preach' (C) they may be less likely to engage with these interventions (O) because they consider these to lack credibility (M)	Data extracted from 2 documents:<br><br>1 research article (qualitative interview study); 1 local evaluation report
CMOC A25	When attendees receive advice or information relating to healthy lifestyles that they already familiar with during a check (C) they may perceive the advice to be useless (O) because they have heard it before (M)	Data extracted from 8 documents:<br><br>6 research articles (4 qualitative interview studies, 1 ethnography, 1 mixed methods study); 2 PhD theses (ethnography, interviews/ethnography)

For peer review only

CMOC A26	When attendees learn something new and important to them during a check (C) they may attempt to make changes to their lifestyle in light of this (O) because they have an improved understanding of risk factors or lifestyle advice (M)	Data extracted from 6 documents:<br><br>3 research articles (1 qualitative interview study, 1 ethnography, 1 survey); 1 research report (RCT); 1 local evaluation report; 1 PhD thesis (interviews/ethnography)
CMOC A27	When attendees are aware of conflicting or inconsistent guidance about healthy lifestyles (C) they may reject or ignore advice delivered during a check (O) because they doubt its credibility (M1) or are confused (M2)	Data extracted from 4 documents:<br><br>2 research articles (2 qualitative interview studies); 1 research report (RCT); 1 PhD thesis (ethnography)
CMOC A28	When attendees are aware of controversy in relation to recommended medication (statins) (C) they may be ambivalent about accepting or adhering to a prescription (O) because they are uncertain of the benefits (M)	Data extracted from 5 documents:<br><br>3 research articles (3 qualitative interview studies; 1 local evaluation reports; 1 conference presentation
CMOC A29	When attendees have doubts about the accuracy of the tests and tools used to measure and assess risk during a check (C) they may be less receptive to advice, brief interventions, referrals or prescriptions offered to address identified risks (O) because they are unsure that the assessments are credible (M)	Data extracted from 6 documents:<br><br>2 research articles (1 qualitative interview study, 1 mixed methods study); 1 research report (mixed methods study); 2 local evaluation reports; 1 PhD thesis (interviews/ethnography)
Practical constraints: referrals and follow-up		

For peer review only

CMOC A30	When attendees receive continuity of care and follow up after a check (C) they may be more likely to engage with advice, take up referrals or attempt to make lifestyle changes (O) because they are reminded of what they are meant to be doing and why (M)	Data extracted from 10 documents:<br><br>5 research articles (4 qualitative interview studies, 1 pilot RCT); 1 research report (RCT); 2 local evaluation reports; 1 PhD thesis (interviews/ethnography); 1 conference poster
CMOC A31	When attendees receive continuity of care and follow up after a check (C) they may be more likely to engage with advice, take up referrals or attempt to make lifestyle changes (O) because they feel supported and valued (M)	Data extracted from 5 documents:<br><br>2 research articles (2 qualitative interview studies); 1 research reports (RCT); 1 local evaluation report; 1 PhD thesis (ethnography)
CMOC A32	When attendees are repeatedly offered follow up and feedback on progress after a check (C) they may be motivated to attempt and maintain lifestyle changes (O) because they can monitor their progress (M)	Data extracted from 6 documents:<br><br>3 research articles (3 qualitative interview studies); 1 research report (RCT); 2 local evaluation reports
CMOC A33	When attendees can see tangible benefits of making lifestyle changes after a check (C) they are more likely to maintain these (O) because they are motivated to continue (M)	Data extracted from 5 documents:<br><br>1 research article (qualitative interview study); 2 local evaluation reports; 2 conference materials

For peer review only

CMOC A34	When attendees are not offered any follow up (beyond the five-year NHSHC programme cycle) (C) they may lack motivation to attempt any lifestyle changes (O) because they interpret the absence of follow up to mean there is no urgent need to make changes (M)	Data extracted from 3 documents:<br><br>1 research article (survey); 1 research report (RCT); 1 PhD thesis (interviews/ethnography)
CMOC A35	When attendees don't consider local lifestyle services to be convenient, appropriate or likely to meet their needs (C) they are less likely to take up referrals or attend these services (O) because they feel it is pointless (M)	Data extracted from 14 documents:<br><br>9 research articles (7 qualitative interviews studies, 1 systematic review, 1 mixed methods study); 2 local evaluation reports; 2 PhD theses (ethnography, interviews/ethnography); 1 conference abstract
CMOC A36	When local lifestyle support services are designed to be more accessible (e.g. in terms of timing, location, cost) (C) attendees may be more likely to start and continue to attend (O) because they feel they are more convenient, affordable or relevant (M)	Data extracted from 4 documents:<br><br>3 research articles (3 qualitative interview studies); 1 conference poster
CMOC A37	When attendees have the option to try out a lifestyle service or are supported to try one by a provider (C) they may be more likely to take up an offer of a referral (O) because they feel more confident to do so (M)	Data extracted from 3 documents:<br><br>1 research article (pilot RCT); 1 local evaluation report; 1 conference abstract
Practical constraints for attendees: person-centredness		

CMOC A38	When attendees receive advice about healthy lifestyles that does not take account of their personal circumstances (C) they are less likely to engage with it (O) because they believe it is unworkable for them (M)	Data extracted from 10 documents:<br><br>8 research articles (7 qualitative interview studies, 1 mixed methods study); 1 PhD thesis (ethnography); 1 conference presentation
CMOC A39	When attendees receive advice about healthy lifestyles that they believe they cannot achieve (e.g. because it seems to require big changes) (C) they are less likely to engage with it (O) because they feel overwhelmed and hopeless (M)	Data extracted from 3 documents:<br><br>3 research articles (2 qualitative interview studies, 1 observational study using video recordings)
CMOC A40	When attendees receive advice about healthy lifestyles that attendees feel they can fit into their lives (e.g. around other commitments) (C) they may be more likely to engage with it (O) because they perceive the changes to be less disruptive (M)	Data extracted from 13 documents:<br><br>6 research articles (4 qualitative interview studies, 1 ethnography, 1 pilot RCT); 1 research report (RCT); 5 local evaluation reports; 1 conference poster
CMOC A41	When attendees feel that significant lifestyle change is unworkable for them (C) they may be more likely to accept prescriptions (e.g. for statins) (O) because they still want to do something to reduce their CVD risk (M)	Data extracted from 2 documents:<br><br>1 research article (qualitative interview study); 1 local evaluation report
CMOC A42	When attendees anticipate or experience medication side effects or burdens (C) they may be more ambivalent about accepting or adhering to prescriptions (O) because they are uncertain of the benefits and concerned about harms (M)	Data extracted from 4 documents:<br><br>2 research articles (2 qualitative interview studies); 1 local evaluation report; 1 conference presentation

For peer review only

CMOC A43	When attendees anticipate or experience medication side effects or burdens (C) they attempt lifestyle change (O) because they prefer this option (M)	Data extracted from 3 documents:<br><br>2 research articles (2 qualitative interview studies); 1 local evaluation report
CMOC A44	When attendees are encouraged and supported by friends, family or peers to make and sustain lifestyle changes (C) they may be more likely to attempt and maintain changes (O) but the mechanism for this is unclear (M not defined)	Data extracted from 11 documents:<br><br>7 research articles (5 qualitative interview studies, 1 ethnography, 1 co-production study); 1 research report (RCT); 1 local evaluation report; 2 PhD theses (ethnography, interviews/ethnography)

For peer review only

BMJ Open

Understanding what happens to attendees after an NHS Health Check: a realist review

Journal:	*BMJ Open*
Manuscript ID	bmjopen-2022-064237.R2
Article Type:	Original research
Date Submitted by the Author:	12-Sep-2022
Complete List of Authors:	Duddy, Claire; University of Oxford, Nuffield Department of Primary Care Health Sciences<br>Gadsby, Erica; University of Stirling, Faculty of Health Sciences and Sport<br>Hibberd, Vivienne; University of Greenwich Medway School of Pharmacy, Public Involvement in Pharmacy Studies Group; University of Kent Medway School of Pharmacy<br>Krska, Janet; University of Greenwich Medway School of Pharmacy; University of Kent Medway School of Pharmacy<br>Wong, Geoff; Oxford University, Nuffield Department of Primary Care Health Sciences
**Primary Subject Heading**:	Public health
Secondary Subject Heading:	Cardiovascular medicine, General practice / Family practice, Health policy
Keywords:	PUBLIC HEALTH, CARDIOLOGY, Health policy < HEALTH SERVICES ADMINISTRATION & MANAGEMENT, Risk management < HEALTH SERVICES ADMINISTRATION & MANAGEMENT, PREVENTIVE MEDICINE

SCHOLARONE™
Manuscripts

I, the Submitting Author has the right to grant and does grant on behalf of all authors of the Work (as defined in the below author licence), an exclusive licence and/or a non-exclusive licence for contributions from authors who are: i) UK Crown employees; ii) where BMJ has agreed a CC-BY licence shall apply, and/or iii) in accordance with the terms applicable for US Federal Government officers or employees acting as part of their official duties; on a worldwide, perpetual, irrevocable, royalty-free basis to BMJ Publishing Group Ltd ("BMJ") its licensees and where the relevant Journal is co-owned by BMJ to the co-owners of the Journal, to publish the Work in this journal and any other BMJ products and to exploit all rights, as set out in our licence.

The Submitting Author accepts and understands that any supply made under these terms is made by BMJ to the Submitting Author unless you are acting as an employee on behalf of your employer or a postgraduate student of an affiliated institution which is paying any applicable article publishing charge ("APC") for Open Access articles. Where the Submitting Author wishes to make the Work available on an Open Access basis (and intends to pay the relevant APC), the terms of reuse of such Open Access shall be governed by a Creative Commons licence – details of these licences and which Creative Commons licence will apply to this Work are set out in our licence referred to above.

Other than as permitted in any relevant BMJ Author's Self Archiving Policies, I confirm this Work has not been accepted for publication elsewhere, is not being considered for publication elsewhere and does not duplicate material already published. I confirm all authors consent to publication of this Work and authorise the granting of this licence.

For peer review only

Understanding what happens to attendees after an NHS Health Check: a realist review

Claire Duddy[1*], Erica Gadsby[2], Vivienne Hibberd[3], Janet Krska[4], Geoff Wong[1]

[1] Nuffield Department of Primary Care Health Sciences, University of Oxford, Oxford, UK

[2] Faculty of Health Sciences and Sport, University of Stirling, Stirling, UK and Centre for Health Services Studies, University of Kent, Canterbury, UK.

[3] Public Involvement in Pharmacy Studies Group, Medway School of Pharmacy, Universities of Greenwich and Kent, Chatham Maritime, UK

[4] Medway School of Pharmacy, Universities of Greenwich and Kent, Chatham Maritime, UK.

*Corresponding author

claire.duddy@phc.ox.ac.uk

01865 617855

Radcliffe Primary Care Building

Radcliffe Observatory Quarter

Woodstock Road

Oxford OX2 6GG.

Word count: **7040** (excluding title page, abstract, references, tables, acknowledgements)

For peer review only - http://bmjopen.bmj.com/site/about/guidelines.xhtml

For peer review only

Abstract

Objectives

The NHS Health Check offers adults aged 40-74 an assessment of their risk of developing cardiovascular disease. Attendees should be offered appropriate clinical or behavioural interventions to help them to manage or reduce these risks. This project focused on understanding variation in the advice and support offered to Health Check attendees.

Design

We conducted a realist review, assembling a diverse body of literature via database searches (MEDLINE, Embase, CINAHL, HMIC, Web of Science) and other search methods, and synthesised data extracted from documents using a realist logic of analysis. Our aim was to develop an understanding of contexts affecting delivery of the NHS Health Check and the underlying mechanisms producing outcomes related to the offer for attendees post-Check.

Results

Our findings demonstrate differences in how NHS Health Check commissioners, providers and attendees understand the primary purpose of the programme. A focus on screening for disease can produce an emphasis on high-volume delivery in primary care. When delivery models are organised around behavioural approaches to risk reduction, more emphasis is placed on advice, and referrals to 'lifestyle services'. However, constrained funding and competing priorities for providers limit what can be delivered within the programme's remit. Attendees' experiences and responses to the programme are affected by how the programme is delivered, and by the difficulty of incorporating its outputs into their lives.

Conclusions

The remit of the NHS Health Check should be reviewed with consideration of what can be effectively delivered within existing resources. Variation in delivery may be appropriate to meet local needs, but differences in how the programme's primary purpose is understood contributes to a 'postcode lottery' in post-Check advice and support. Our findings underline existing concerns that the programme may

For peer review only

generate inequitable outcomes and raise questions about whether it can deliver positive outcomes for the majority of attendees.

Study registration

PROSPERO CRD42020163822

Keywords

cardiovascular diseases, prevention, public health, lifestyle, health promotion, screening, realist review

Article Summary

Strengths and limitations of this study

- This is the first realist review focused on the NHS Health Check programme and it has generated new understanding of the causes of variation in delivery at the end of the Health Check pathway;
- The review is inclusive of a wide range of literature, including grey literature, allowing us to draw on learning from local delivery models;
- This project was strengthened by strong and consistent PPI (patient and public involvement) and professional stakeholder input, which shaped and tested the relevance of our theories throughout;
- As with any review, our findings were limited by the availability and quality of the available evidence – we sought to fill a gap in the existing literature focused on the end of the Health Check pathway but this necessarily limited the number and quality of documents available.

Background

The NHS Health Check in England is a large-scale public health programme that aims to offer adults aged 40-74 a five-yearly assessment of their risk of developing cardiovascular disease (CVD) (excluding those with a pre-existing CVD-related condition).(1) The NHS Health Check involves the measurement of multiple CVD risk factors and the delivery of advice and discussion of appropriate clinical and behavioural approaches that might help individuals to manage and reduce their CVD risk.

For peer review only - http://bmjopen.bmj.com/site/about/guidelines.xhtml

These could include, for example, referral to a GP to discuss recommended pharmacological options (generally statins or antihypertensives) or the delivery of advice, signposting or formal referral to 'lifestyle' services, such as smoking cessation and weight management programmes.(2)

The programme is concerned with both the early identification of risk factors for CVD and the delivery of early interventions to address them, via both pharmacological and non-pharmacological, 'lifestyle' or 'behavioural' means. It is also tasked with helping to address population-level health disparities, reflecting an understanding that the burden of CVD is not spread equally throughout the population, but is strongly linked to factors including deprivation and ethnicity. The intention to use the programme to address health inequalities has been present since its launch; most recently, the programme is cited in the UK Government's recent 'Levelling Up' white paper as a potential means of delivering its prevention agenda.(3) To meet its aims of preventing CVD at a population level, and contributing to reducing the health inequalities associated with CVD, the NHS Health Check must be widely taken up (especially by those with most to gain), and be effective in both assessing and reducing or managing any risks identified.

The NHS Health Check was first introduced in 2009 but the programme was relaunched in 2013, when responsibility for commissioning the programme transferred from NHS Primary Care Trusts (PCTs) to local authorities (LAs). Minimum standards for programme delivery – focused on the collection of certain measurements and targets to increase the volume of checks delivered - became statutory requirements at this time(4, 5) and Public Health England (PHE) was formed and given oversight of the programme. In 2021, the new Office for Health Improvement and Disparities (OHID) took on responsibility for the programme and published a major review (instigated and conducted by PHE), setting out an updated 'vision' for the NHS Health Check. The review's recommendations include aims to launch a digital service and to increase the size and remit of the programme by extending it to cover younger age groups and to address more conditions beyond CVD.(6)

The NHS Health Check has long been the subject of controversy, with some calling its effectiveness and cost-effectiveness into question,(7-10) and PHE responding by collating evidence that supports the programme under the auspices of the Expert Scientific and Clinical Advisory Panel (ESCAP).(11)

For peer review only - http://bmjopen.bmj.com/site/about/guidelines.xhtml

For peer review only

Observational studies included in two PHE-commissioned rapid reviews suggest that the programme is associated with increased rates of CVD risk factor and disease detection, statin prescribing and referrals to 'lifestyle services' (including smoking cessation, weight management, exercise and alcohol support services). Work undertaken to support the recent PHE/OHID review of the programme has also identified an association between Health Check attendance and improvement in a range of indicators, including: BMI, rates of smoking, blood pressure, total cholesterol, hospital admissions related to CVD or type 2 diabetes, and all-cause death, after five years,(12) although the direction of causality is unclear.

Regional and local studies of the implementation and outcomes of the programme demonstrate wide variation in programme delivery and outcomes across England. Evidence on the delivery of advice, referrals and on behaviour changes post-Health Check is especially sparse. The rapid reviews identified only six primary studies examining behaviour change, all focused only on smoking cessation.(13, 14)

Our scoping searches and an initial review of the existing research evidence relating to the programme identified a clear focus on invitation, uptake and coverage of the NHS Health Check. Far less attention has been paid to what happens after the measurements and risk assessments have been undertaken, especially in relation to the delivery of advice, onward signposting or referral and ongoing support for behaviour changes that might reduce CVD risks. The capacity of the NHS Health Check to provide attendees with appropriate advice, referrals and support – and the extent to which attendees respond to these – is a critical assumption underpinning the effectiveness of the programme.

Current best practice guidance makes recommendations for NHS Health Check commissioners and providers, describing a range of possible advice and referral options that may be made available, as well as clinical interventions that may be appropriate to offer attendees.(2) It is clear that responsibility for clinical follow up rests with primary care, and the guidance suggests that commissioners "may wish to" put referral pathways to onward services, such as smoking cessation services, in place. Patel et al's large-scale observational study of the NHS Health Check for the period 2012-2017 includes data relating to the provision of "advice, information or referral" for

For peer review only - http://bmjopen.bmj.com/site/about/guidelines.xhtml

different risk factors that may be flagged during a check.(15) Although data recording for these activities is likely to be incomplete, the figures presented in this study suggest that there is wide variation in the delivery and recording of these activities for different risk factors, and that rates of delivery fall well below the recommended thresholds for intervention. For example, only one quarter of attendees recorded as meeting the threshold for intervention are recorded as receiving advice, information or referral in relation to diet. In addition, this national-level study obscures the wide regional variation in what they term 'post-delivery management' following a check, identified in existing reviews that include local studies.(13, 14)

In consultation with our stakeholder groups (see Methods below for more details on group membership) we focused our review on these final steps in the NHS Health Check pathway. Our aim was to develop an understanding of how the NHS Health Check programme works in different settings, and for different groups to achieve its outcomes, with a specific focus on what happens after the measurements and risk assessment are complete.

Methods

We conducted a realist review to synthesise evidence that could help us to develop an understanding of the important contexts that influence the delivery of the NHS Health Check and the mechanisms that produce intended and unintended outcomes. Realist review is an interpretive, theory-driven approach, chosen because existing research clearly demonstrates that the NHS Health Check programme is a complex intervention with context-sensitive outcomes. Our realist analysis used data extracted from the literature to develop context-mechanism-outcome configurations (CMOCs). CMOCs are causal explanations that describe why and how (by which mechanisms) particular outcomes are generated in particular contexts. Following the realist approach, our findings are an interpretation of a constellation of data extracted from the documents, built on direct evidence but also on silences and contrasts observed across multiple data points extracted from multiple documents.

The NHS Health Check programme is characterised by wide variation in commissioning and delivery and hence in the experience of attendees. Our review sought to make sense of this variation, by developing a realist programme theory, based on CMOCs, that elucidates the causes of outcomes

For peer review only - http://bmjopen.bmj.com/site/about/guidelines.xhtml

related to the final steps in the patient pathway, i.e. on what happens after the measurements and risk assessments are complete.

Our methods are described in brief below, while full details are available in a detailed protocol paper.(16) The conduct and reporting of the review followed the RAMESES (Realist and Meta-narrative Evidence Syntheses: Evolving Standards) quality(17) and reporting standards.(18) A glossary of realist terminology and RAMESES checklist are included in **Supplementary File 1**.

Patient and public involvement

Our review was informed by the involvement of two stakeholder groups, who provided us with content expertise based on their lived experience of commissioning, providing or receiving (or being eligible to receive) the NHS Health Check. Our patient and public involvement (PPI) group comprised 10 members of the public from 6 English regions and were selected to be as diverse as possible in relation to age, gender, ethnicity and geography. Our 'professional' stakeholder group were recruited via our existing networks and snowballing, and included policy makers, LA commissioners, NHS Health Check providers and a trainer, and representatives from relevant health charities. Our final meeting with the latter group was expanded to include 36 individuals, to ensure a broad range of feedback on our findings and recommendations for policy and practice.

Both groups were consulted via regular online meetings throughout the project. We asked these groups to provide feedback and responses to our emerging findings as the project developed. For example, we asked them to reflect on how our findings fit with (or did not fit with) their own knowledge and experience of commissioning, providing or attending an NHS Health Check. These discussions helped to shape our analysis, highlight areas of importance to different groups and inform the development of our recommendations. Our PPI group consistently observed a 'mismatch' between the focus of the NHS Health Check programme and their own health priorities, and a lack of understanding of the programme's purpose. This informed our analysis of data relating to attendees' understanding of their health needs and our overall focus on different groups' understanding of the purpose of the programme.

For peer review only - http://bmjopen.bmj.com/site/about/guidelines.xhtml

In our final meetings with each group, we asked our stakeholders for their input to help us to develop and refine recommendations to inform NHS Health Check policy, commissioning and delivery. We also sought their input to help us to develop appropriate dissemination strategies, tailored project outputs and important audiences to share our findings.

Realist review methods

Our realist review followed Pawson's five iterative steps(19), as described in detail in our protocol.(16) These steps are summarised in **Table 1** below. We used the PRISMA (Preferred Reporting Items for Systematic Reviews and Meta-Analyses) checklist (where applicable) when writing our report (see **Supplementary File 1** for details).(20)

Table 1: Summary of methods

	Aim	Approach
Step 1: Locate existing theories	To identify existing theories that offer explanations from when, how and for whom the NHS Health Checks programme 'works'<br><br>See **Supplementary File 1**	Informal literature searching, focused on existing reviews and programme documentation (CD)<br>Drawing on experience and knowledge of project team and stakeholders (All)
Step 2: Search for evidence	To gather a body of literature containing data that could be used to refine the initial programme theory	Screening material included in two existing reviews(13, 14)<br>Additional searches across multiple databases (see **Supplementary File 2**)<br>Trawling NHS Health Check website for grey literature<br>Citation chaining<br>Email alert to capture new material (CD)
Step 3: Article selection	Within the material identified as described above, to select documents that could contribute relevant data for theory development	Screening title and abstract and then in full text (CD)<br>Inclusion of documents based on assessment of relevance and rigour(21)<br>10% of records screened in duplicate, with discrepancies resolved by discussion (CD, EG, GW)
Step 4: Extracting and organising data	To describe and organise included documents<br>To extract and code relevant data from included documents and begin analysis<br><br>See **Supplementary File 3.** The full set of extracted data is available on request.	Key characteristics of documents captured in an Excel spreadsheet (CD)<br>Relevant data related to our research question and focus coded in NVivo<br>10% of records coded in duplicate and checked by a third reviewer (CD, EG, GW)

For peer review only - http://bmjopen.bmj.com/site/about/guidelines.xhtml

Step 5: Synthesising evidence and drawing conclusions	To apply a realist logic of analysis to extracted data to develop context-mechanism-outcome configurations (CMOCs) related to the end of the NHS Health Check pathway. CMOCs are the building blocks of realist analysis, defining relationships between important outcomes, the mechanisms that produce them and the conditions in which they are likely to occur.<br><br>To develop a final programme theory	Close examination and interpretation of data coded within each category to build CMOCs (CD)<br>Cross-case comparison to identify data that demonstrated similar or contrasting contexts and mechanisms that produced patterns of outcomes.<br>Iterative development of CMOCs as more data was considered and discussed by the project team and stakeholder groups (All)

As the project progressed, we made some minor changes to the protocol.

In Step 2, we anticipated a potential need for further searching for empirical data, but our project team agreed that the processes described above had identified sufficient material to meet our needs. During the analysis, we conducted a series of focused searches to identify material related to one substantive theory – 'street-level bureaucracy', which has been used to support our explanations of variation in delivery of the NHS Health Check.(22) The details of these searches are also reproduced in **Supplementary File 2**.

For Step 3, our original criteria were updated in light of the review project's chosen focus on what happens after measurements and risk assessments are completed. We sought in particular documents that contained data relating to later steps in the NHS Health Check pathway, including the provision of advice, signposting, referrals or prescriptions, and outcomes associated with these.

In Step 4, CD and EG coded a 10% set of the included documents independently and GW provided an additional check to ensure that all relevant data was captured and coding was applied consistently.

Results

Documents included in the review

In total, 124 documents contributed data to the review, including: 59 published research papers or reports; 20 documents reporting local evaluations; 34 conference materials (presentations, abstracts and posters) and 11 others (including policy reports, guidance, news articles and theses). Of these, 21

For peer review only - http://bmjopen.bmj.com/site/about/guidelines.xhtml

contributed data relating to the NHS Health Check programme as a whole, and the rest focused on specific localities. The processes of identifying, screening and selecting these are summarised below in Figure 1 (an adapted version of the PRISMA flow diagram).(20)

Figure 1: Summary of searching and selection processes

[Figure 1 here]

The main characteristics of the included documents are provided in **Supplementary File 3.** This table also indicates the extent to which each document contributed to the analysis, by showing the contribution of extracted data from each to specific CMOCs.

Review findings

Our findings describe what our included data told us about what happens at the end of an NHS Health Check, after the measurements and risk assessments are completed. Our explanations describe the reasoning and responses of three important groups involved in the programme: LA commissioners, NHS Health Check providers, and attendees. A narrative summary of our findings follows; it is based on our realist analysis that developed 86 CMOCs underpinned by the data included in the review. Overall, our findings illustrate how the delivery and outcomes of local NHS Health Check programmes and individual Heath Check encounters are shaped by a wide range of influences. To provide transparency, a detailed summary of the CMOCs developed and the data underpinning each is available in **Supplementary File 4**. We have included references to illustrative CMOCs to support the narrative that follows. CMOCs are labelled according to the perspective that they focus on, i.e. commissioner-focused CMOCs are designated ‘C’, provider-focused CMOCs ‘P’ and attendee-focused CMOCs ‘A’.

Understanding and engagement with the NHS Health Check

Our review findings point to variation in understandings of the primary purpose of the NHS Health Check. This variation is a critical factor that determines how the programme is commissioned, delivered and received. The programme was designed with two aims in mind: early identification of cases of diagnoseable illness or individuals with ‘high’ risk, and early intervention to support

For peer review only - http://bmjopen.bmj.com/site/about/guidelines.xhtml

individuals to manage and reduce their CVD risk, via prescribing or by prompting or enabling behaviour change. Further to these, the programme is also cast as an opportunity to address disparities in CVD risk factors across England, although it maintains a focus on individual behaviours. Whilst there is a focus in the literature on inequality in relation to invitation and coverage of the check,(23, 24) our review uncovered very little data and almost no focus on the relationship between inequalities and the delivery of advice and referral. This gap is also reflected in the evidence collated to support the recently published PHE/OHID-led review.(12)

Our analyses of the data suggest that commissioners and providers may have a tendency to prioritise some aspects of the programme over others, for example, increasing volume of delivery and delivery of risk assessments over risk management. This prioritisation is then reflected in local commissioning decisions and delivery models. When the programme is understood primarily as an opportunity to screen for cases of existing CVD or near-CVD (very high risk factors), responsibility for programme delivery and outcomes is likely to rest with primary care, and specifically in general practice, where most NHS Health Checks are provided, and where clinical and especially pharmacological follow-up should happen (see, e.g. *CMOCs C9-C10* in **Supplementary File 4**). This perspective leads to an emphasis on high-volume throughput, increasing coverage of the programme, and a focus on efficient delivery of the minimum requirements for each check, collecting mandatory data and communicating risk scores. At the other end of the spectrum, where commissioners and providers are more oriented towards using the programme as a means of supporting behaviour change in order to reduce or manage CVD risk, delivery reflects this, with greater emphasis on advice or coaching, and facilitating onward referrals to 'lifestyle services', such as smoking cessation or weight management programmes (*CMOCs C1-C8)*.

Our data demonstrates how such differences in local priorities for the NHS Health Check can drive variation in who delivers the check and the training they receive, the settings in which checks are delivered and the time allocated to each check (*CMOCs C1-C8, P14, P26*), all of which go on to affect how attendees experience the programme (*CMOCs A12-A17, A30-A37)*. In addition, these differences can affect the availability, accessibility and connectedness of referral pathways to local

For peer review only - http://bmjopen.bmj.com/site/about/guidelines.xhtml

'lifestyle services' that might be offered to attendees post-check, and important differences in the information that LAs collect, how they monitor and evaluate programme delivery, as well as funding models that can incentivise certain aspects of delivery (*CMOCs C2-C3, C5-C8)*. Commissioners and providers each influence how the programme is shaped and implemented at local levels, exercising discretion in determining the remit and focus of the programme.

This double layer of discretion in how the NHS Health Check is ultimately enacted in different local areas means that the relationship between LA commissioners and providers is an important factor in determining what local delivery looks like. Commissioners determine service specifications and funding models and the scope of monitoring of programme delivery, but (unless they are themselves providers, as is the case for some provision in a few LAs) their day-to-day influence on actual delivery is limited (*CMOC C4*). This is a particular concern as prevailing scepticism (in particular amongst GPs) about the programme's effectiveness and concerns about the potential for overdiagnosis has led to disengagement amongst some providers. Such concerns, however, must be balanced with their reliance on income generated by the programme (*CMOCs P1-P5*). Differences in levels of engagement with the programme can reflect different understandings about its primary purpose and concerns about its ability to meet its aims, but they can also be fuelled by more practical concerns, including competing priorities and workload pressures, and the need to deliver NHS Health Checks and appropriate follow-up with limited resources (see below and *CMOCs P9-P13, P15-P18*).

Local delivery models send important signals to attendees that inform how they understand and engage with the programme in turn. Checks delivered in general practice with a focus on completing mandatory measurements and risk assessments carry the implication that the check should be understood as a screening test or a health 'MOT', that may or may not result in the need for clinical intervention (*CMOCs A1*, *A12*). Delivery in community settings, by providers trained specifically to deliver coaching or behavioural support, or otherwise with a greater focus on delivering advice or referrals, sends a different message *(CMOC A14).* In some areas, NHS Health Checks have been delivered within a wider integrated lifestyle service, facilitating connections with lifestyle services, and potentially additional services such as link workers or social prescribers. These models may

For peer review only - http://bmjopen.bmj.com/site/about/guidelines.xhtml

enable providers to offer more holistic support that better meets the needs of individual attendees; much data from the attendee perspective suggests that this may help to generate better engagement with the programme and its aims.

Our data also suggest that attendees' prior expectations of the check also inform their response, and so it is important that national and local advertising about the programme are clear about its remit and purpose. Providers can transmit 'soft' signals to attendees about the programme's purpose and value, and attendees will pick up on these (*CMOCs A17-24)*. They have the potential to convey urgency, and even induce fear and anxiety in attendees, but they may also (intentionally or otherwise) imply that the check is a 'box ticking' exercise.

Finally, it is important to acknowledge that attendees' themselves can exercise discretion in their response to their check, regardless of how providers approach its delivery. The programme's focus on individual behaviour creates high expectations of individual action to address any 'lifestyle risk factors' identified. Inevitably, attendees may face considerable challenges in implementing behaviour changes within their own lives, where they too may have limited resources (*CMOCs A38-A34*). Some literature has drawn attention to the risks inherent in this individual focus, including the potential for positive health impacts to be realised only by those with the capacity to make significant lifestyle changes, and so unfairly distributing any benefits amongst attendees.(25) Such a focus risks increasing rather than decreasing health inequalities.

Practical constraints limiting the NHS Health Check

Commissioners and providers face substantial practical constraints that limit and inform their exercise of discretion in programme delivery. As noted above, the decisions of LA commissioners that are enacted in funding models, service specifications and monitoring regimes set important boundaries for providers, potentially incentivising different delivery methods and priorities. Commissioners' decisions must take into account restricted funding for public health initiatives (*CMOCs C12-C14*). Central government grants to LAs have fallen substantially since 2010. Since public health responsibilities were transferred to LAs in 2013, the public health grant has decreased by 13% in real terms.(26) Spending on the NHS Health Check programme fell by 21% between 2015/16 and

For peer review only - http://bmjopen.bmj.com/site/about/guidelines.xhtml

2019/20, and spending on 'lifestyle services' that could potentially support attendees post-check has also fallen: expenditure on smoking cessation initiatives fell by one third over the same period, adult alcohol and drug services by 17% and weight management services by 5%.(27) Reductions in funding of this scale necessarily limit what commissioners can purchase (*CMOCs C14, A35-A37)*. Our findings raise the question of whether public health funding overall is adequate to support the programme's more ambitious aim of preventing CVD and reducing CVD inequalities by reducing or managing behavioural risk factors. In particular, it is apparent that the delivery of personalised, individual advice and discussion during checks, and the offer of further support afterwards, is a more intensive proposition than a programme focused on case finding (*CMOCs P9-P13)*.

Providers face other constraints. There is a need to ensure that staff involved in delivering checks are competent to do so, but some data suggest that not all providers feel confident in delivering support for behaviour change (*CMOCs P20-P23)*. Training, too, must reflect the programme's aims both to identify and to address CVD risk factors (*CMOC P26*). Wherever checks are delivered in general practice or community pharmacy settings, providers face competing priorities and demands on their time. Workload and time pressure may push providers towards 'leaner' delivery, focused on mandatory tests and capturing required measurements, leaving little time for discussion, advice and offers of referral (*CMOCs P9-P14)*.

There is a crucial relationship between the practical constraints that commissioners and providers face and their understanding and engagement with the programme. It is likely that both groups of actors adapt their understanding of the programme based on what they know about the limited resources available to deliver and support it. Downward pressure on funding inevitably incentivises leaner delivery models and, as our findings make clear, these models tend to favour the 'early identification' or case finding purpose of the programme. Provider scepticism is also likely to be compounded by the sense that both the programme itself, and subsequent services on which it might depend are underfunded and inaccessible (*CMOCs P1-P3)*.

Finally, our data make clear the impact of the varied delivery models on attendees' experience of the programme and on what they are offered (or not) post-check (*CMOCs A1, A5-A6, A11-A14, A30-A32,*

For peer review only - http://bmjopen.bmj.com/site/about/guidelines.xhtml

A34, A38-A40). It is unclear which delivery models allow or incentivise providers to deliver the meaningful, personalised and ongoing advice and support attendees might need. Existing ethnographic research has demonstrated that the time-constrained and highly structured nature of the NHS Health Check assessments impede meaningful discussion that prioritises understanding individuals' circumstances.(28, 29) Limited access to 'lifestyle services' means these are inaccessible to many.

Variation in NHS Health Check delivery models: street-level bureaucracy

Our review findings demonstrate wide variation in how the NHS Health Check programme is implemented locally, with a specific focus in variation in the delivery of what happens after measurements and risk assessments have been completed. Inconsistencies in the recording of these activities (especially the delivery and uptake of advice and referrals post-check) prevent the development of a comprehensive picture of this variation across England. However, our review findings provide a starting point to improve understanding of what influences delivery in these areas, highlighting the discretion available to LA commissioners and individual NHS Health Check providers in making decisions about how the programme is delivered on the ground. In addition, they highlight how differences in delivery models affect how the checks are experienced by attendees, and what colours their responses to the information they receive and any offers of further intervention.

Our understanding of the processes at work in driving variation draws on Lipsky's concept of 'street-level bureaucracy', borrowed from the international relations literature.(22, 30) Previous research on the NHS Health Check,(31) and the implementation of other health policies in the UK(32, 33) have used the same theory to add explanatory value. Lipsky's framework emphasises the discretion of those charged with implementing national policies or programmes, as well as their responses to working with limited resources. For the NHS Health Check programme, it is clear that while commissioners and providers are working within the broad constraints of a legal framework(4, 5) and guidance issued by PHE(2) (now OHID), their decisions and everyday practice in delivering checks effectively determine the remit and purpose of the programme at local levels. As Lipsky describes it: "*the decisions of street-level bureaucrats, the routines they establish, and the devices they invent to*

For peer review only - http://bmjopen.bmj.com/site/about/guidelines.xhtml

cope with uncertainties and work pressures, effectively become the public policies they carry out".(30)

The extent to which discretion can be exercised in relation to the NHS Health Check may be greatest at the end of the programme pathway. Processes relating to earlier steps – the identification of the eligible population, invitation and the actual measurements and risk assessments to be administered – are restricted by the programme's legal framework and clear guidance, leaving little room for local interpretation or adaptation. The later steps – especially the delivery of advice and referrals – are less prescribed and more dependent on local delivery models and other local services. Activity in these areas is less well recorded and monitored, leaving LA commissioners and providers with more discretion to determine what day-to-day local delivery of checks will look like.

Although Lipsky's original framework focuses on those directly engaged in the delivery of policies and their interaction with the recipients or subjects of those policies (in our case, the interaction of providers with attendees) our analysis also highlights the discretion of LA commissioners who make decisions about local programme specifications and support. Whilst they may not directly interact with the public, commissioners must also interpret the requirements of the programme and exercise their own discretion to ensure it meets local needs and is delivered within local constraints. The approach of the LA directly affects commissioned providers, and this double layer of discretion forms the local contexts in which NHS Health Checks are delivered across England.

Taken together, the CMOCs developed in this review point to the influence of both commissioners' and providers' understanding and engagement with the programme, as well as the effects of practical constraints that drive decision making in relation to programme delivery. These two aspects mirror Lipsky's concepts of *discretion* in the enactment of policies and programmes, and the effects of their responses to *limited resources*. Attendees' experience of, and response to the programme is affected both by the outcomes of commissioner and provider decisions, but also by other external factors related to their individual circumstances, which may be difficult to align with what the NHS Health Check expects of them.

For peer review only - http://bmjopen.bmj.com/site/about/guidelines.xhtml

Discussion

Summary of findings

The success of the NHS Health Check programme as an intervention that aims to support individuals to manage and reduce their CVD risk rests on what happens to attendees when the measurements and risk assessments have been completed. While case-finding is an important indicator of success, the delivery of advice, offers of signposting, referrals or other support, and attendees' responses to these, is equally crucial for the programme to prevent ill-health and reduce inequalities in the longer term. Our focus on this area reflected the importance of this step – confirmed by our two stakeholder groups – but also the relative lack of attention that it has received in the existing research literature. Our review therefore sought to examine the final steps in the NHS Health Check programme pathway, to understand the factors that influence the delivery of these parts of the check and what follows, and how they are received by those who receive checks.

The NHS Health Check as a whole has been the subject of several previous evidence synthesis projects, which have considered many aspects of the programme, and have similarly identified wide variation in delivery models and outcomes. Our review is the first to use a realist approach, and the first to focus specifically on the steps that follow the measurements and risk assessments made during each check. Relevant findings from existing reviews correspond with our own. The two PHE-commissioned rapid reviews identified some limited evidence of geographical variation in referrals, and captured qualitative data describing providers' doubts and scepticism about the programme, and perceived training needs. These reviews also included qualitative studies focused on attendees, which described attendees' perspectives on the quality of information delivered during checks and the important constraints imposed by "environmental", "resource" and "time" factors that limit attendees' capacity to make and sustain behaviour changes.(13, 14) Another synthesis coded evidence relating to the behaviours of commissioners, providers and attendees in relation to the programme, and reported similar findings related to providers' skills, attitudes and beliefs, as well as the resources available to deliver checks and the need to take account attendees' wider "social contexts" when delivering advice.(34)

For peer review only - http://bmjopen.bmj.com/site/about/guidelines.xhtml

Our review extends the work undertaken in these reviews, by explaining how and why variation in how the programme is delivered comes about, with a particular focus on what happens after the measurements and risk assessments are completed. Our findings point to the significance of the exercise of discretion by LA commissioners and NHS Health Check providers in the delivery of checks. Commissioners' decisions in relation to programme implementation, funding and monitoring, and providers' actions to deliver checks on the ground are influenced by multiple factors. These include their understanding of the primary purpose of the programme and engagement with its aims, but these attitudes towards the programme – and commissioners' and providers' actions in organising and providing checks – are constrained by important practical factors.

Prevailing conditions, including limited funding for public health programmes and services overall, current funding and monitoring arrangements for the NHS Health Check programme, and competing priorities for many providers tend to push towards a delivery model that prioritises the programme's aim of early detection – case finding. The prioritisation of this element of the programme may be to the detriment of work that could help to prompt or support behaviour change. These constraints raise ethical questions about the programme: if it is oriented towards early identification of risk, but cannot genuinely support early intervention to help manage and reduce risks (bar prescribing), where does this leave attendees? There is a need to consider the relationship between the NHS Health Check programme and the wider landscape of local and national services that can be offered to attendees.

In some local areas, there is evidence that commissioners and providers work against this tide, using their local NHS Health Checks as a means of supporting attendees to make behaviour changes that could help to reduce their CVD risk. Our final programme theory diagram summarises these findings and is presented below in **Figure 2**.

Figure 2: Final programme theory

[Figure 2 here]

For peer review only - http://bmjopen.bmj.com/site/about/guidelines.xhtml

Covid-19 and the NHS Health Check

We undertook this review during the Covid-19 pandemic, which had a major impact on delivery of the NHS Health Check. In April 2020, the programme was effectively paused.(35, 36) To support resumption of delivery, PHE issued 'restart guidance' in April 2021, encouraging LAs to consider restarting the programme, dependent on local safety assessments and the need to prioritise the vaccination programme.(37) This document acknowledged the pressures faced by general practice in particular, and urged LA commissioners to consider 'alternative' providers. Other communications from PHE and the Department of Health and Social care during the pandemic highlighted the potential benefits of the NHS Health Check in relation to identifying risk factors for severe Covid-19 outcomes, as many CVD risk factors are also associated with higher risks of hospitalisation and death from Covid-19.(38-40)

In light of our findings, we note that the effects of the pandemic have the potential to exacerbate some existing contexts that may adversely affect delivery of the programme's aims. In particular, pressure to 'catch up' and concerns about delayed or missed diagnoses could lead to a more intense focus on early detection and case finding.(41) Workload pressures affecting primary care providers may also increase disengagement from the NHS Health Check, and LA commissioners must consider these effects as they restart the programme in each local area.

Implications for policy and practice

Our review findings raise a number of important questions for policy makers, commissioners and providers to consider in relation to the NHS Health Check. Our main recommendation is that all three of these groups (and future researchers) should increase their focus on evaluating and improving the delivery of the later steps in the programme pathway, i.e. on what happens to attendees after the measurements and risk assessments have been completed. Our more detailed recommendations are summarised below in **Table 2**. They are organised around four important and inter-connected principles, developed in consultation with our PPI and professional stakeholder groups: clarifying the purpose of the programme; increasing engagement with the programme; focusing the Health Check

For peer review only - http://bmjopen.bmj.com/site/about/guidelines.xhtml

on attendee needs; and improving links between the NHS Health Check and other programmes and services.

Table 2: Summary of recommendations based on review findings

Our recommendation	Rationale
Ensure all national and local guidance and programme documentation reflects the importance of the programme's aims in relation to both early identification of CVD risk, and early intervention to manage/reduce that risk.	Different interpretations of the primary purpose of the programme drive variation in commissioning and delivery, and subsequently in attendee experience.
Assess overall programme funding and local funding arrangements in relation to delivery of (all aspects of) the programme's aims.	Commissioner, provider and attendee scepticism about the programme can undermine its delivery; inadequate resources for the programme and support services for attendees post-check can reduce engagement. Funding arrangements may incentivise leaner or more intensive checks.
Review national and local data monitoring and evaluation of the programme, to ensure that data relating to all key aspects of the programme (and especially the final steps in the NHS Health Check pathway) are captured.	Data collection is currently focused on invitation, uptake and coverage, incentivising high-volume, less intensive delivery that focuses on mandatory data points. Data on advice and referral (including data relating to potential disparities in provision) are needed to evaluate the delivery of these steps.
Review training for providers to assess its ability to support all aspects of programme delivery, including the final steps in the NHS Health Check pathway.	Providers and attendees have identified deficiencies in skills and knowledge, especially in relation to the delivery of advice, support and referrals. All providers should receive adequate training to ensure that they can deliver these aspects of a check or signpost/refer attendees to an appropriate source of support.
Review connections between the programme and national and local services that could offer further support for attendees, and options for longer term follow-up after measurements and risk assessments are completed.	The success of the programme rests in part on its connections with other services that could offer further support for attendees. Strengthening these connections could allow the NHS Health Check to act as a gateway to these services (as well as to primary care/general practice when necessary)
Continue to produce evidence for programme effectiveness and address the relative scarcity of evidence focused on later steps in the NHS Health Check pathway.	Commissioner, provider and attendee scepticism about the programme can undermine its delivery; evidence from research and evaluation should go beyond a focus on increasing coverage of the programme and inform practical recommendations for good practice in delivery.

For peer review only - http://bmjopen.bmj.com/site/about/guidelines.xhtml

Our recommendations must be considered alongside those made by PHE/OHID in their recently published national review of the Health Checks programme.(6) They support several of the recommendations made by OHID, and in particular the identification of the need to "build sustained engagement" and "create a learning system". In these areas, OHID's specific proposals include: ensuring a clear focus on the programme's aim to "[promote] lasting health and wellbeing"; developing a national training offer with a focus on supporting behaviour change; providing sufficient provision of post-check services; and continued evaluation of the programme.

However, our findings raise concerns about the implementation of some of the recommendations from the national review. In particular, two major recommendations ("start younger" and "address more conditions") propose to significantly expand the scope and coverage of the NHS Health Check programme. Our findings relating to attendee experience suggest that moves to make the programme more holistic may be welcomed by some, but indicate that policy makers should be cautious about any programme expansion. There is a risk that expanding the programme's scope could add to existing confusion about its primary purpose and drive further local variation in delivery. Existing capacity in primary care and community and public health services also limit the feasibility of these proposals. Without sufficient appropriate follow-up, extensions of the programme's remit risk leaving more attendees with few options for ongoing support to help them to address or manage any risks or conditions identified. Without additional investment in services and convincing evidence of their clinical and cost-effectiveness, such expansion also risks increasing provider scepticism and disengagement from the programme.

OHID's proposal to launch a digital version of the NHS Health Check should also consider our findings, and in particular, ensure that the final steps in the programme pathway are not neglected. In any delivery format, there is a need to ensure that where risks are identified, relevant advice and links to appropriate services are provided. Finally, we note that OHID's recommendation to continue to increase participation reinforces the existing focus on invitation and uptake of the NHS Health Check. Policy makers should be cognisant that measures that encourage and incentivise high volume delivery

For peer review only

of checks can detract from the delivery of high quality, personalised advice and ongoing support for longer-term behaviour change.

Strengths and limitations of this review

Our review has developed novel interpretations of existing secondary data relating to what happens at the end of the NHS Health Check pathway. Our close examination of what happens after the measurements and risk assessments have been completed during checks helps to address the relative lack of research on this particular aspect of programme delivery. The review project was strengthened by close working with our diverse PPI and professional stakeholder groups, who helped us to focus the review, provided detailed feedback on emerging findings and shaped our interpretation of the data and the development of recommendations. We included a diverse range of material in the review, and in particular, drew on the learning captured in a wide range of grey literature including local evaluations and conference materials.

As with any review, our findings are limited by the availability and quality of the literature. The material included in the review covers a wide date range and some older material may be less applicable to the present day. However, we considered each piece of data carefully before inclusion and aimed to select material that spoke to still-relevant contexts, mechanisms and outcomes. Our stakeholders also helped us to confirm the contemporary relevance of our findings. Our CMOCs vary in terms of the volume and rigour of the data that underpin them. We have provided a full and transparent account of that data in **Supplementary Files 3 and 4,** so that the strength of each is made clear to readers, such that they can make their own judgement on the plausibility of our interpretations.

Conclusions

Our review has revealed wide variation in the delivery of advice and support, and onward signposting and referral for attendees and identified explanations for this variation. We have identified a wide range of influences that affect how LA commissioners and NHS Health Check providers develop and deliver the programme at local levels across England, which affect how attendees experience and respond to their check. Our analysis explains how differences in understanding of the primary purpose

For peer review only - http://bmjopen.bmj.com/site/about/guidelines.xhtml

of the programme influences commissioning and implementation, and how practical constraints limit what can be delivered within the programme's remit and existing resources. Based on our findings, we developed a set of recommendations for policy makers, commissioners and providers to inform future programme development. Our recommendations centre on the need for greater emphasis to be placed on the final steps in the NHS Health Check programme pathway, including in national and local guidance and programme documentation, funding models, provider training, monitoring and evaluation of the programme, and in considering how the NHS Health Check can be better linked with wider services and programmes.

List of abbreviations

BMI: Body mass index

CINAHL: Cumulative Index to Nursing and Allied Health Literature

CMOC: Context-mechanism-outcome configuration (see glossary for a definition)

CVD: Cardiovascular Disease

DHSC: Department of Health and Social Care

GP: General Practitioner

HMIC: Health Management Information Consortium

IPT: Initial Programme Theory

LA: Local authority

MOT: 'Ministry of Transport', test to determine if cars are roadworthy

NHS: National Health Service

NICE: National Institute for Health and Care Excellence

OHID: Office for Health Improvement and Disparities

PHE: Public Health England

For peer review only - http://bmjopen.bmj.com/site/about/guidelines.xhtml

PPI: Patient and Public Involvement

PRISMA: Preferred Reporting Items for Systematic Reviews and Meta-Analyses

RAMESES: Realist and Meta-narrative Evidence Syntheses: Evolving Standards

Required statements

Acknowledgements

We would like to express our thanks for the help, feedback and advice provided by all of our wonderful stakeholders and patient and public involvement contributors, and for the continued engagement of PHE and OHID and their help with the recruitment of stakeholders and opportunities for dissemination of our findings and outputs.

Ethics approval

Not applicable for this review

Author contributions

JK and GW conceived the research project and all of the authors contributed to its design. GW provided methodological support. CD devised and carried out the literature searches and led the analysis and interpretation of the data. The other authors (EG, VH, JK, GW) contributed to the analysis. VH led the PPI involvement. CD drafted the manuscript and the other authors (EG, VH, JK, GW) provided feedback. All of the authors read and approved the final manuscript.

Funding statement

This project was funded by the National Institute for Health Research (NIHR) Health Services and Delivery Research programme (NIHR129209). The views expressed are those of the authors and not necessarily those of the NIHR or the Department of Health and Social Care.

Competing interests

CD and GW are both members of the Royal College of General Practitioners (UK) Overdiagnosis and Overtreatment Group. GW is an NHS General Practitioner and deputy chair of the National Institute

For peer review only - http://bmjopen.bmj.com/site/about/guidelines.xhtml

for Health Research Health Technology Assessment Prioritisation Committee: Integrated Community Health and Social Care Panel (A) and member of the Methods Group (A).

Data sharing statement

The datasets generated and/or analysed during the current study are not publicly available due to copyright and licensing restrictions but are available from the corresponding author on reasonable request.

References

1. NHS. NHS Health Check 2020 [Available from: https://www.nhs.uk/conditions/nhs-health-check/.
2. Public Health England. NHS Health Check Best practice guidance For commissioners and providers. 2020.
3. HM Government. Levelling Up. Levelling Up the United Kingdom. Her Majesty's Stationery Office; 2021 2 February 2022.
4. Great Britain. The Local Authorities (Public Health Functions and Entry to Premises by Local Healthwatch Representatives) Regulations. Part 2 Public Health Functions: Conduct of Health Checks. United Kingdom; 2013.
5. Great Britain. The Local Authorities (Public Health Functions and Entry to Premises by Local Healthwatch Representatives) Regulations. Part 2: Public Health Functions: Health check assessment. United Kingdom; 2013.
6. Office for Health Improvement and Disparities. NHS Health Check Programme review. 2021.
7. Abdalrahman B, Soljak M. NHS health checks: an update on the debate and program implementation in England. J Ambul Care Manage. 2015;38(1):5-9.
8. McCartney M. Where's the evidence for NHS health checks? BMJ. 2013;347:f5834.
9. Capewell S, McCartney M, Holland W. Invited debate Response to Waterall et al. J Public Health (Oxf). 2015;37(2):185-6.
10. Ford J, Ekeke N, Lahiri A, Kelly MP. Making the case for prevention. Cambridge, UK: A commissioned report by Cambridge Public Health, University of Cambridge, for the Health Foundation,; 2021.
11. Public Health England. Review of NHS Health Checks: terms of reference. 2020.
12. Office for Health Improvement and Disparities. NHS Health Check Programme review. Annex B: a summary of analyses and evidence on the current NHS Health Check programme. 2021.
13. Usher-Smith JA, Mant J, Martin A, Harte E, MacLure C, Meads C, et al. NHS Health Check Programme rapid evidence synthesis. Cambridge, UK: The Primary Care Unit, University of Cambridge, RAND Europe, Public Health England; 2017.
14. Tanner L, Kenny R, Still M, Pearson F, Bhardwaj-Gosling R. NHS Health Check Programme Rapid Review Update. University of Sunderland, Newcastle University, Public Health England; 2020.
15. Patel R, Barnard S, Thompson K, Lagord C, Clegg E, Worrall R, et al. Evaluation of the uptake and delivery of the NHS Health Check programme in England, using primary care data from 9.5 million people: a cross-sectional study. BMJ Open. 2020;10(11):e042963.
16. Duddy C, Wong G, Gadsby EW, Krska J, Hibberd V. NHS Health Check programme: a protocol for a realist review. BMJ Open. 2021;11(4):e048937.
17. The RAMESES Project. Quality Standards for Realist Synthesis (for researchers and peer-reviewers). 2014.
18. Wong G, Greenhalgh T, Westhorp G, Buckingham J, Pawson R. RAMESES publication standards: realist syntheses. BMC Med. 2013;11(1):21.

For peer review only - http://bmjopen.bmj.com/site/about/guidelines.xhtml
For peer review only

19. Pawson R. Realist synthesis: new protocols for systematic review. 2006. In: Evidence-based policy: a realist perspective [Internet]. [73-104].
20. Page MJ, McKenzie JE, Bossuyt PM, Boutron I, Hoffmann TC, Mulrow CD, et al. The PRISMA 2020 statement: an updated guideline for reporting systematic reviews. BMJ. 2021;372:n71.
21. Wong G. Data gathering in realist reviews: Looking for needles in haystacks. 2018. In: Doing Realist Research [Internet]. London: SAGE Publications Ltd; [131-46].
22. Lipsky M. Toward a theory of street-level bureaucracy. Madison, WI: Institute for Research on Poverty (IRP), University of Wisconsin,; 1969.
23. Bunten A, Porter L, Gold N, Bogle V. A systematic review of factors influencing NHS health check uptake: invitation methods, patient characteristics, and the impact of interventions. BMC public health. 2020;20(1):93.
24. Martin A, Saunders CL, Harte E, Griffin SJ, MacLure C, Mant J, et al. Delivery and impact of the NHS Health Check in the first 8 years: a systematic review. Br J Gen Pract. 2018;68(672):e449-e59.
25. Stol YH, Schermer MHN, Asscher ECA. Omnipresent Health Checks May Result in Over-responsibilization. Public Health Ethics. 2017;10(1):35-48.
26. The King's Fund. Spending on public health 2021 [Available from: https://www.kingsfund.org.uk/projects/nhs-in-a-nutshell/spending-public-health.
27. Finch D, Marshall L, Bunbury S. Why greater investment in the public health grant should be a priority. The Health Foundation; 2021.
28. Cupit C. An ethnographic study of cardiovascular disease prevention: the social organisation of measures, knowledge, interventions and tensions in English general practice: University of Leicester; 2018.
29. Strutt E. Patient-centred care: Patients' experiences of and responses to the National Health Service (NHS) Health Check programme in general practice. Durham: Durham University; 2011.
30. Lipsky M. Street-Level Bureaucracy: Dilemmas of the Individual in Public Service. 30th Anniversary Edition ed. New York, New York: Russell Sage Foundation; 2010.
31. Gale N, Dowswell G, Greenfield S, Marshall T. Street-level diplomacy? Communicative and adaptive work at the front line of implementing public health policies in primary care. Soc Sci Med. 2017;177:9-18.
32. Bergen A, While A. 'Implementation deficit' and 'street-level bureaucracy': policy, practice and change in the development of community nursing issues. Health Soc Care Community. 2005;13(1):1-10.
33. Cooper MJF, Sornalingam S, O'Donnell C. Street-level bureaucracy: an underused theoretical model for general practice? Br J Gen Pract. 2015;65(636):376.
34. Atkins L, Stefanidou C, Chadborn T, Thompson K, Michie S, Lorencatto F. Influences on NHS Health Check behaviours: a systematic review. BMC Public Health. 2020;20(1):1359.
35. NHS England and Improvement. COVID-19 Prioritisation within Community Health Services [Letter]. 2020.
36. Thompson K. NHS Health Check e-Bulletin - April 2020 Operational Update. 2020. Contract No.: 30th April 2020.
37. Public Health England. NHS Health Check: Restart Preparation. 2021. Contract No.: Version 11: 09 April 2021.
38. Public Health England. NHS Health Check e-Bulletin - August 2020. 2020. Contract No.: 5th January 2022.
39. Public Health England. Summary: An Umbrella Review on Cardiovascular Risk Factors, Cardiovascular Disease and COVID-19. 2021.
40. Cabinet Office. Our plan to rebuild: The UK Government's COVID-19 recovery strategy. 2020. Contract No.: 24th February 2022.
41. Oxford Academic Health Science Network. CVD prevention during and after the COVID-19 pandemic. Getting It Right First Time, Association of Directors of Public Health; 2020.

For peer review only - http://bmjopen.bmj.com/site/about/guidelines.xhtml

For peer review only

For peer review only - http://bmjopen.bmj.com/site/about/guidelines.xhtml

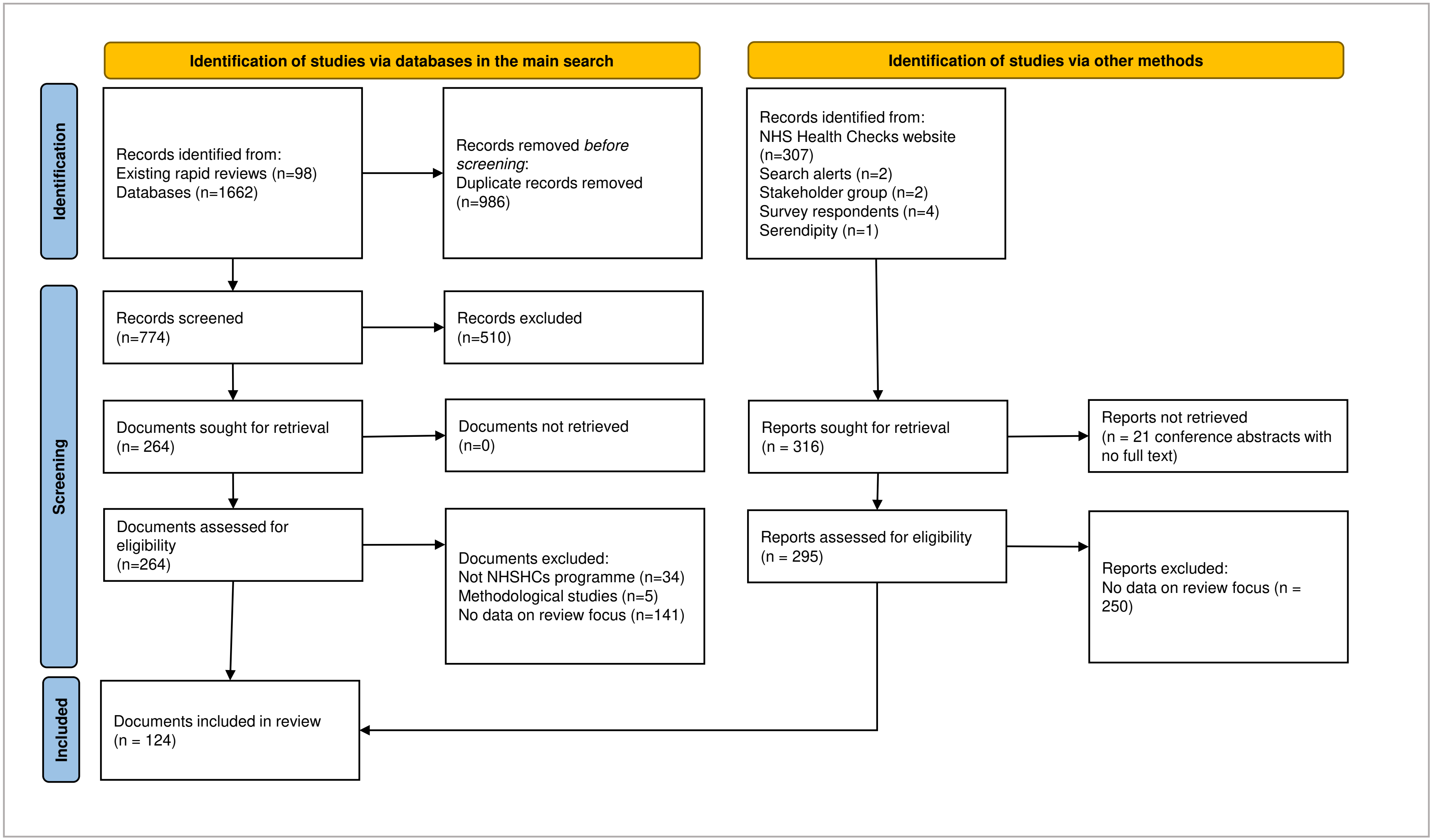
Identification of studies via databases in the main search
Identification of studies via other methods
Identification
Screening
Included
Records identified from:
Existing rapid reviews (n=98)
Databases (n=1662)
Records removed *before screening*:
Duplicate records removed
(n=986)
Records identified from:
NHS Health Checks website
(n=307)
Search alerts (n=2)
Stakeholder group (n=2)
Survey respondents (n=4)
Serendipity (n=1)
Records screened
(n=774)
Records excluded
(n=510)
Documents sought for retrieval
(n= 264)
Documents not retrieved
(n=0)
Reports sought for retrieval
(n = 316)
Reports not retrieved
(n = 21 conference abstracts with no full text)
Documents assessed for eligibility
(n=264)
Documents excluded:
Not NHSHCs programme (n=34)
Methodological studies (n=5)
No data on review focus (n=141)
Reports assessed for eligibility
(n = 295)
Reports excluded:
No data on review focus (n = 250)
Documents included in review
(n = 124)

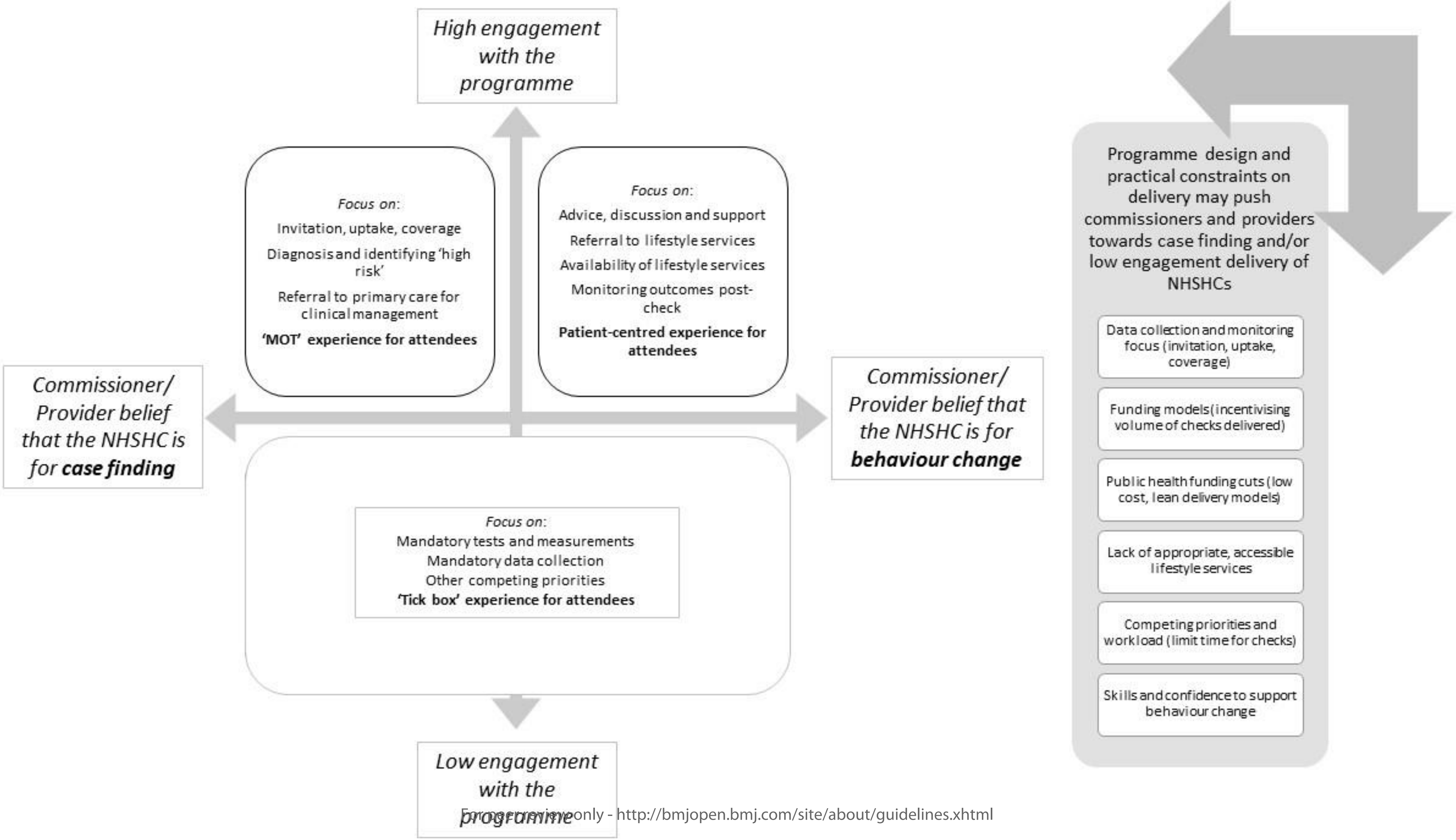
High engagement with the programme
Focus on:
Invitation, uptake, coverage
Diagnosis and identifying 'high risk'
Referral to primary care for clinical management
'MOT' experience for attendees
Focus on:
Advice, discussion and support
Referral to lifestyle services
Availability of lifestyle services
Monitoring outcomes post-check
Patient-centred experience for attendees
Commissioner/ Provider belief that the NHSHC is for case finding
Commissioner/ Provider belief that the NHSHC is for behaviour change
Focus on:
Mandatory tests and measurements
Mandatory data collection
Other competing priorities
'Tick box' experience for attendees
Low engagement with the programme
Programme design and practical constraints on delivery may push commissioners and providers towards case finding and/or low engagement delivery of NHSHCs
Data collection and monitoring focus (invitation, uptake, coverage)
Funding models (incentivising volume of checks delivered)
Public health funding cuts (low cost, lean delivery models)
Lack of appropriate, accessible lifestyle services
Competing priorities and workload (limit time for checks)
Skills and confidence to support behaviour change

Supplementary File 1

This file contains a glossary of realist terminology, a diagram summarising our initial programme theory and the focus of the realist review and PRISMA and RAMESES checklists.

Glossary of realist terminology

Context: in realist research, the conditions or circumstances in which mechanisms that generate observed outcomes are 'triggered' or activated.

Context-mechanism-outcome configuration (CMOC): a heuristic used to present a realist causal explanation for an outcome, presented as a relationship between some particular context(s) and mechanism(s).

Demi-regularity: a semi-predictable pattern of outcomes that occur in the same context(s).

Mechanism: in realist research, the underlying context-sensitive causal force that generates an outcome, often conceptualised as the response of an individual actor to important context(s).

Programme theory: a set of theoretical explanations about how a programme, intervention or process is understood to work. Realist programme theories explain or the process by which outcomes of interest are thought to be generated, using causal explanations captured in the form of CMOCs.

Substantive theory: an established, formal theory drawn from any discipline that can be used to help understand the programme, intervention or process under examination.

For peer review only

Initial programme theory diagram

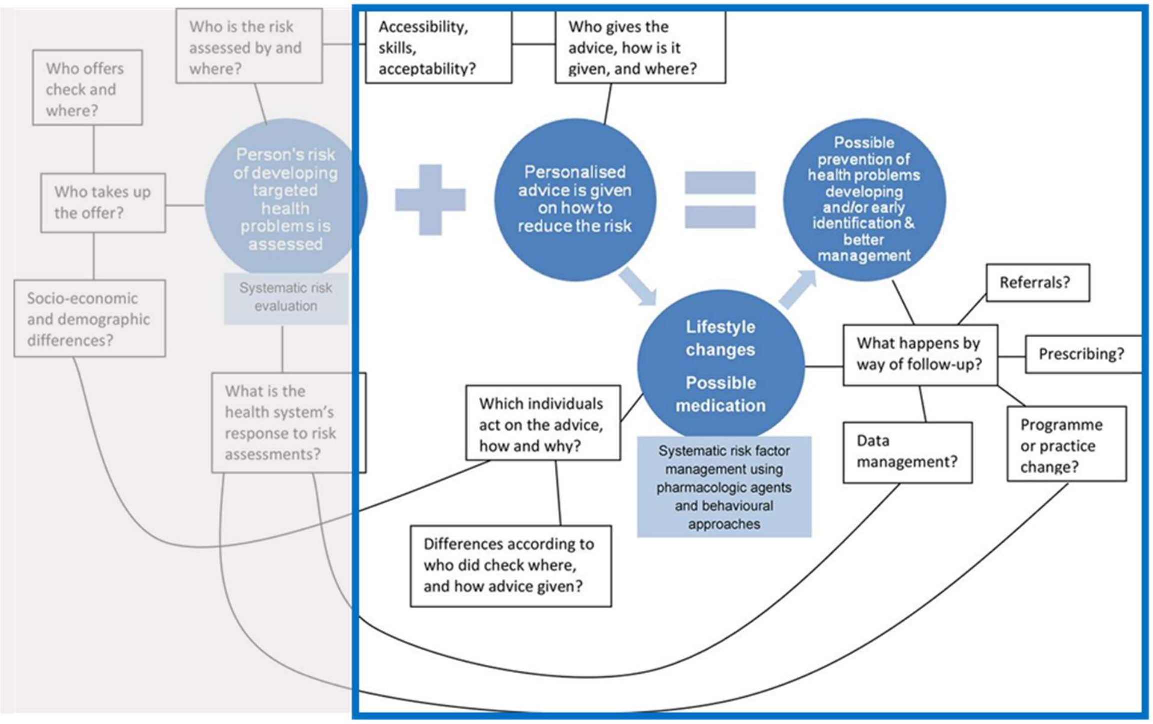

Initial programme theory for the realist review with project focus highlighted

review only

PRISMA checklist

Reporting checklist for systematic review (with or without a meta-analysis).

Based on the PRISMA guidelines.

Instructions to authors

Complete this checklist by entering the page numbers from your manuscript where readers will find each of the items listed below.

Your article may not currently address all the items on the checklist. Please modify your text to include the missing information. If you are certain that an item does not apply, please write "n/a" and provide a short explanation.

Upload your completed checklist as an extra file when you submit to a journal.

In your methods section, say that you used the PRISMAreporting guidelines, and cite them as:

Page MJ, McKenzie JE, Bossuyt PM, Boutron I, Hoffmann TC, Mulrow CD, Shamseer L, Tetzlaff JM, Akl EA, Brennan SE, Chou R, Glanville J, Grimshaw JM, Hróbjartsson A, Lalu MM, Li T, Loder EW, Mayo-Wilson E, McDonald S, McGuinness LA, Stewart LA, Thomas J, Tricco AC, Welch VA, Whiting P, Moher D. The PRISMA 2020 statement: An updated guideline for reporting systematic reviews

		Reporting Item	Page Number
**Title**			
Title	#1	Identify the report as a systematic review	1
**Abstract**			
Abstract	#2	Report an abstract addressing each item in the PRISMA 2020 for Abstracts checklist	2
**Introduction**			
Background/rationale	#3	Describe the rationale for the review in the context of existing knowledge	3-6
Objectives	#4	Provide an explicit statement of the objective(s) or question(s) the review addresses	6, protocol
**Methods**			

For peer review only

Eligibility criteria	#5	Specify the inclusion and exclusion criteria for the review and how studies were grouped for the syntheses	8-9, protocol
Information sources	#6	Specify all databases, registers, websites, organisations, reference lists, and other sources searched or consulted to identify studies. Specify the date when each source was last searched or consulted	8, Supplementary File 2
Search strategy	#7	Present the full search strategies for all databases, registers, and websites, including any filters and limits used	Supplementary File 2
Selection process	#8	Specify the methods used to decide whether a study met the inclusion criteria of the review, including how many reviewers screened each record and each report retrieved, whether they worked independently, and, if applicable, details of automation tools used in the process	8, Table 1 and protocol
Data collection process	#9	Specify the methods used to collect data from reports, including how many reviewers collected data from each report, whether they worked independently, any processes for obtaining or confirming data from study investigators, and, if applicable, details of automation tools used in the process	8-9, Table 1 and protocol
Data items	#10a	List and define all outcomes for which data were sought. Specify whether all results that were compatible with each outcome domain in each study were sought (for example, for all measures, time points, analyses), and, if not, the methods used to decide which results to collect	n/a

For peer review only

Study risk of bias assessment	#11	Specify the methods used to assess risk of bias in the included studies, including details of the tool(s) used, how many reviewers assessed each study and whether they worked independently, and, if applicable, details of automation tools used in the process	n/a
Effect measures	#12	Specify for each outcome the effect measure(s) (such as risk ratio, mean difference) used in the synthesis or presentation of results	n/a
Synthesis methods	#13a	Describe the processes used to decide which studies were eligible for each synthesis (such as tabulating the study intervention characteristics and comparing against the planned groups for each synthesis (item #5))	8-9, Table 1, protocol
Synthesis methods	#13b	Describe any methods required to prepare the data for presentation or synthesis, such as handling of missing summary statistics or data conversions	n/a
Synthesis methods	#13c	Describe any methods used to tabulate or visually display results of individual studies and syntheses	n/a
Synthesis methods	#13d	Describe any methods used to synthesise results and provide a rationale for the choice(s). If meta-analysis was performed, describe the model(s), method(s) to identify the presence and extent of statistical heterogeneity, and software package(s) used	8-9, Table 1, protocol
Synthesis methods	#13e	Describe any methods used to explore possible causes of heterogeneity among study results (such as subgroup analysis, meta-regression)	n/a

For peer review only

Synthesis methods	#13f	Describe any sensitivity analyses conducted to assess robustness of the synthesised results	n/a
Reporting bias assessment	#14	Describe any methods used to assess risk of bias due to missing results in a synthesis (arising from reporting biases)	n/a
Certainty assessment	#15	Describe any methods used to assess certainty (or confidence) in the body of evidence for an outcome	n/a
Data items	#10b	List and define all other variables for which data were sought (such as participant and intervention characteristics, funding sources). Describe any assumptions made about any missing or unclear information	n/a
**Results**			
Study selection	#16a	Describe the results of the search and selection process, from the number of records identified in the search to the number of studies included in the review, ideally using a flow diagram (http://www.prisma-statement.org/PRISMAStatement/FlowDiagram)	9, Figure 1
Study selection	#16b	Cite studies that might appear to meet the inclusion criteria, but which were excluded, and explain why they were excluded	n/a
Study characteristics	#17	Cite each included study and present its characteristics	Supplementary File 3
Risk of bias in studies	#18	Present assessments of risk of bias for each included study	n/a
Results of individual studies	#19	For all outcomes, present for each study (a) summary statistics for each group (where appropriate) and (b) an effect estimate and its	n/a

For peer review only

		precision (such as confidence/credible interval), ideally using structured tables or plots	
Results of syntheses	#20a	For each synthesis, briefly summarise the characteristics and risk of bias among contributing studies	n/a
Results of syntheses	#20b	Present results of all statistical syntheses conducted. If meta-analysis was done, present for each the summary estimate and its precision (such as confidence/credible interval) and measures of statistical heterogeneity. If comparing groups, describe the direction of the effect	n/a
Results of syntheses	#20c	Present results of all investigations of possible causes of heterogeneity among study results	n/a
Results of syntheses	#20d	Present results of all sensitivity analyses conducted to assess the robustness of the synthesised results	n/a
Risk of reporting biases in syntheses	#21	Present assessments of risk of bias due to missing results (arising from reporting biases) for each synthesis assessed	n/a
Certainty of evidence	#22	Present assessments of certainty (or confidence) in the body of evidence for each outcome assessed	n/a
**Discussion**			
Results in context	#23a	Provide a general interpretation of the results in the context of other evidence	16-18
Limitations of included studies	#23b	Discuss any limitations of the evidence included in the review	20-21
Limitations of the review methods	#23c	Discuss any limitations of the review processes used	20-21

For peer review only

Implications	#23d	Discuss implications of the results for practice, policy, and future research	19-20
**Other information**			
Registration and protocol	#24a	Provide registration information for the review, including register name and registration number, or state that the review was not registered	3
Registration and protocol	#24b	Indicate where the review protocol can be accessed, or state that a protocol was not prepared	6, 8
Registration and protocol	#24c	Describe and explain any amendments to information provided at registration or in the protocol	8-9
Support	#25	Describe sources of financial or non-financial support for the review, and the role of the funders or sponsors in the review	23
Competing interests	#26	Declare any competing interests of review authors	23
Availability of data, code, and other materials	#27	Report which of the following are publicly available and where they can be found: template data collection forms; data extracted from included studies; data used for all analyses; analytic code; any other materials used in the review	23

Notes:

- 4: 6, protocol
- 5: 8-9, protocol
- 6: 8, Supplementary File 2
- 7: Supplementary File 2
- 8: 8, Table 1 and protocol

For peer review only

- 9: 8-9, Table 1 and protocol
- 13a: 8-9, Table 1, protocol
- 13d: 8-9, Table 1, protocol
- 16a: 9, Figure 1
- 17: Supplementary File 3 The PRISMA checklist is distributed under the terms of the Creative Commons Attribution License CC-BY. This checklist was completed on 25. April 2022 using https://www.goodreports.org/, a tool made by the EQUATOR Network in collaboration with Penelope.ai

For peer review only

RAMESES checklist

List of items when reporting a realist synthesis

Reporting item		Description of item	Reported on page(s):
TITLE			
1		In the title, identify the document as a realist synthesis or review	1
ABSTRACT			
2		While acknowledging publication requirements and house style, abstracts should ideally contain brief details of: the study's background, review question or objectives; search strategy; methods of selection, appraisal, analysis and synthesis of sources; main results; and implications for practice.	2
INTRODUCTION			
3	Rationale for review	Explain why the review is needed and what it is likely to contribute to existing understanding of the topic area.	5-6
4	Objectives and focus of review	State the objective(s) of the review and/or the review question(s). Define and provide a rationale for the focus of the review.	5-6
METHODS			
5	Changes in the review process	Any changes made to the review process that was initially planned should be briefly described and justified.	9
6	Rationale for using realist synthesis	Explain why realist synthesis was considered the most appropriate method to use.	6
7	Scoping the literature	Describe and justify the initial process of exploratory scoping of the literature.	Table 1, page 8 Supplementary File 1
8	Searching processes	While considering specific requirements of the journal or other publication outlet, state and provide a rationale for how the iterative searching was done. Provide details on all the sources accessed for information in the review. Where searching in electronic databases has taken place, the details should include, for example, name of database, search terms, dates of coverage and date last searched. If individuals	Table 1, page 8 Supplementary File 2

For peer review only

		familiar with the relevant literature and/or topic area were contacted, indicate how they were identified and selected.	
9	Selection and appraisal of documents	Explain how judgements were made about including and excluding data from documents, and justify these.	Table 1, page 8
10	Data extraction	Describe and explain which data or information were extracted from the included documents and justify this selection.	Table 1, page 8
11	Analysis and synthesis processes	Describe the analysis and synthesis processes in detail. This section should include information on the constructs analyzed and describe the analytic process.	Table 1, page 9
RESULTS			
12	Document flow diagram	Provide details on the number of documents assessed for eligibility and included in the review with reasons for exclusion at each stage as well as an indication of their source of origin (for example, from searching databases, reference lists and so on). You may consider using the example templates (which are likely to need modification to suit the data) that are provided.	Figure 1, page 10
13	Document characteristics	Provide information on the characteristics of the documents included in the review.	Supplementary File 3
14	Main findings	Present the key findings with a specific focus on theory building and testing.	10-16
DISCUSSION			
15	Summary of findings	Summarize the main findings, taking into account the review's objective(s), research question(s), focus and intended audience(s).	17-19
16	Strengths, limitations and future research directions	Discuss both the strengths of the review and its limitations. These should include (but need not be restricted to) (a) consideration of all the steps in the review process and (b) comment on the overall strength of evidence supporting the explanatory insights which emerged.<br>The limitations identified may point to areas where further work is needed.	22
17	Comparison with existing literature	Where applicable, compare and contrast the review's findings with the existing literature (for example, other reviews) on the same topic.	17-18
18	Conclusion and recommendations	List the main implications of the findings and place these in the context of other relevant literature. If appropriate, offer recommendations for policy and practice.	19-23

For peer review only

19	Funding	Provide details of funding source (if any) for the review, the role played by the funder (if any) and any conflicts of interests of the reviewers.	24

For peer review only

Supplementary File 2

This file provides the full details of the search strategies employed in the review to identify documents containing relevant data.

Searches to locate existing theories (Step 1)

We searched PubMed and Web of Science (Core Collection) using a combination of terms relating to HCs, and terms relating to theory, adopting a slightly modified version of Booth and Carroll's (2015) 'BeHEMoTh' approach. As the HC programme is a specific health context (H) and we were interested in theorisation of all steps in the programme, no individual behaviours (Be) were specified.

PubMed (11th October 2020)

1. "Health Check" OR "Health Checks" (3847)
2. UK OR United Kingdom OR England OR Britain OR British (1790010)
3. model OR models OR modelling OR theor* OR concept* OR framework* (4625627)
4. 1 AND 2 AND 3 (130 hits)

Web of Science (SCI-EXPANDED, SSCI, AHCI, ESCI) (11th October 2020)

1. "Health Check" OR "Health Checks" (Topic) (3474)
2. UK OR "United Kingdom" OR England OR Britain OR British (Topic) (623802)
3. Model OR models OR modelling OR theor* OR concept* OR framework* (Topic) (11284654)
4. 1 AND 2 AND 3 (70 hits)

These searches identified 11 studies utilising 6 theoretical models or frameworks. An additional search for each of these named theories, plus terms relating to the NHSHC, was conducted in Google Scholar. In addition, we also searched Google Scholar for papers relating to the NHSHC that also cited the key citations related to each identified theory. These two steps were conducted in October 2020 and identified a further 8 (5+3) papers.

Searches for evidence on NHSHC (Step 2)

MEDLINE (via Ovid, 11th November 2020)

1. health check*.ti,ab,kw (6084)
2. (NHS or National Health Service or United Kingdom or UK or England or English).ti,ab,kw (370078)
3. exp England/ (107308)
4. 2 or 3 (440075)
5. 1 and 4 (468)

For peer review only

6. limit 5 to (english language and yr="2008-Current") (325)

Embase (via Ovid, 11th November 2020)

1. health check*.ti,ab,kw (8572)
2. (NHS or National Health Service or United Kingdom or UK or England or English).ti,ab,kw (487112)
3. england/ (24573)
4. 2 or 3 (494513)
5. 1 and 4 (521)
6. limit 5 to (english language and yr="2008-Current") (459)

CINAHL (via EbscoHost, 11th November 2020)

1. TX "health check* (2391)
2. TI (NHS OR "National Health Service" OR "United Kingdom" OR UK OR England OR English) OR AB (NHS OR "National Health Service" OR "United Kingdom" OR UK or England OR English) (178298)
3. MH "England" (62863)
4. S2 OR S3
5. S1 AND S4
6. Limiters: Published Date: 20080101-20201231; English Language; Expanders: Apply equivalent subjects (378)

HMIC (via Ovid, 11th November 2020)

1. health check*.mp (598)
2. (NHS or National Health Service or United Kingdom or UK or England or English).mp (118285)
3. exp england/ (26501)
4. exp health authorities in england/ (6680)
5. or/2-4 (135421)
6. 1 and 5 (321)
7. limit 6 to (english language and yr="2008-Current") (191)

Web of Science (SCI-EXPANDED, SSCI; 11th November 2020)

1. TOPIC: ("health check*") (5144)
2. TOPIC: (NHS OR "national health service" OR "united kingdom" OR UK OR England OR English) (522053)
3. #1 AND #2 (358)

4. Refined by: PUBLICATION YEARS: (2020 OR 2012 OR 2019 OR 2011 OR 2018 OR 2010 OR 2017 OR 2009 OR 2016 OR 2008 OR 2015 OR 2014 OR 2013) AND LANGUAGES: (ENGLISH) (309)

Additional searches for theory

A short series of highly focused searches for documents describing street-level bureaucrats or street-level bureaucracy were run in Google Scholar in October and November 2021. The details of these searches and results screened (on screen) are provided below.

Google Scholar, 6th October 2021

("street level bureaucracy" OR "street level bureaucrats") AND ("public health") (8,160)

("street level bureaucracy" OR "street level bureaucrats") AND ("primary care") (2090)

("street level bureaucracy" OR "street level bureaucrats") AND ("general practice" OR “general practices”) (889)

("street level bureaucracy" OR "street level bureaucrats") AND ("local authority" OR “local authorities”) (6320)

For peer review only

Supplementary File 3

This file provides the full details of the documents included in the review.

First author	Year	Document type	Aim or purpose of document	Study design (if applicable)	Perspective(s)	Local area(s)	Commis-sioning period[a]	Contributed data to CMOCs			Total contrib-utions to CMOCs	Source
								C-CMOCs	P-CMOCs	A-CMOCs		
Alageel(1)	2018 (1)	Conference presentation	Summary of results of qualitative interview study (Alageel 2018)	Qualitative interview study	Providers	Lambeth and Lewisham	LA		P7		1	NHSHC website
Alageel(2)	2018 (2)	Journal article	To identify barriers and facilitators to implementing multiple health behaviour change interventions for CVD risk reduction in primary care	Qualitative interview study	Providers	Lambeth and Lewisham	LA	C14	P1, P2, P6, P7, P8, P10, P11, P13, P15, P20, P22, P24, P27	A1, A2, A27, A30, A32, A35, A38, A39	22	Existing review
Alageel(3)	2020	Journal article	To examine factors that might influence engagement and adherence to lifestyle change interventions and medication amongst people recently	Qualitative interview study	Attendees	Lambeth and Lewisham	LA	C14	P23	A2, A6, A10, A12, A15, A25, A28, A30, A31, A32, A35, A36, A38, A43, A44	17	Existing review

For peer review only

			assessed at medium or high risk of CVD									
Alford(4)	2010	Evaluation report	To explore behaviour changes made following community based NHSHC and experience of NHSHCs	Qualitative interview study	Attendees	Knowsley	PCT			A40, A44	2	Existing review
Al-Osaimi(5)	2020	Conference abstract	To describe the implementatio n of software to manage the NHSHC	n/a	Commissioner s	Sandwell	LA	C2, C7			2	NHSHC website
Artac(6)	2013	Journal article	To assess whether the NHSHC was associated with a reduction in CVD risk after one year	Pre/post	Attendees	Hammersmit h and Fulham	PCT		P12		1	Existing review
Atkins(7)	2020	Journal article	To identify behaviours and actors relevant to uptake, delivery and follow up of NHSHCs and influences on those behaviours	Systematic review	n/a	n/a	n/a	C14		A35	2	Searches - MEDLINE

For peer review only

Baker(8)	2014 (1)	Evaluation report	To assess the impact of the NHSHC programme and inform future commissioning (summary)	Cross-sectional	Attendees	Gloucestershire	PCT	C9	P12, P17		3	NHSHC website
Baker(9)	2014 (2)	Journal article	To investigate the perceptions and opinions of patients who attended an NHSHC	Survey	Attendees	Gloucestershire	PCT	C14	P1, P8, P20, P25	A1, A15, A18, A22, A23, A26, A34	12	Existing review
Baker(10)	2015 (1)	Journal article	To investigate health professionals' experiences and perspectives of implementation of the NHSHC	Survey including qualitative analysis of free text responses	Providers	Gloucestershire	PCT	C2	P10, P11, P18		4	Existing review
Baker(11)	2015 (2)	Journal article	To investigate how the local NHSHC pathway was followed and interpreted	Cross-sectional	n/a	Gloucestershire	PCT		P2, P12		2	Existing review
Bell(12)	2019	Conference abstract	To describe the outcomes of NHSHCs delivered in an integrated model with lifestyle services	Cross-sectional	Commissioners	Medway	LA	C3	P19		2	NHSHC website

For peer review only

Boase(13)	2012	Journal article	To explore the perspectives of practice nurses in their role of communicating CVD risk to patients	Qualitative interview study	Providers	Cambridge	PCT		P4, P22, P27	A8, A19	5	Searches - MEDLINE
Boseley(14)	2020	Conference presentation	To describe a local model of NHSHC delivery with integrated lifestyle services	Mixed methods	Commissioners	East Sussex	LA	C3, C6, C7	P14, P15, P19	A4	7	NHSHC website
Brutus(15)	2013	Evaluation report	To review the design of the local NHSHC programme and analyse delivery options available	Cross-sectional	Various	Croydon	PCT	C1, C2, C7	P1, P13, P15, P19	A6, A40	9	NHSHC website
Burgess(16)	2016	Conference presentation	Summary results of a qualitative interview study (Alageel 2020)	Qualitative interview study	Attendees	Lambeth and Lewisham	LA		P1, P23	A2, A5, A10, A20, A23, A28, A33, A42	10	NHSHC website
Burke(17)	2015	Conference presentation	To describe local delivery of NHSHCs with a focus on vulnerable groups	n/a	Commissioners	Leeds	LA			A5, A38	2	NHSHC website
Carter(18)	2016	Journal article	To evaluate local outcomes of NHSHCs with a focus on diagnosis and management after checks	Retrospective cohort	Attendees	Leicester	PCT, Transition	C1			1	Existing review

For peer review only

Centre for Public Scrutiny(19)	2014	Other report	A report on the role of Council Scrutiny in local reviews of NHSHC programmes	n/a	n/a	Various	LA	C1, C11	P1		3	Searches - HMIC
Chatterjee(20)	2017	Journal article	To assess the knowledge, use, and confidence in national physical activity and Chief Medical Officer guidelines and tools among GPs in England	Survey	Providers	England	LA		P21		1	Searches - MEDLINE
Chipchase(21)	2011	Evaluation report	To explore people's views and experiences of the NHSHC	Qualitative interview study	Various	Birmingham	PCT		P1, P14	A1, A2, A11, A12, A19, A20, A40	9	Existing review
Clarke(22)	2020	Conference presentation	To describe the of data to inform local CVD prevention work	n/a	Commissioners	Barnsley	LA	C8		A33	2	NHSHC website
Cochrane(23)	2012 (1)	Journal article	To compare changes in population CVD risk factors between those who receive an NHSHC and those who receive an NHSHC with additional	RCT	n/a	Stoke on Trent	PCT		P26	A9	2	Existing review

For peer review only - http://bmjopen.bmj.com/site/about/guidelines.xhtml

			lifestyle support									
Cochrane(24)	2013	Journal article	To review response, attendance and treatment uptake following NHSHCs	Cross-sectional	n/a	Stoke on Trent	PCT			A9	1	Existing review
Coffey(25)	2014	Evaluation report	To assess the level of uptake of NHSHCs	Cross-sectional	n/a	Salford	PCT		P12, P20		2	Existing review
Coghill(26)	2018	Journal article	To assess uptake and outcomes of NHSHCs in general practice	Cross-sectional	n/a	Bristol	PCT, Transition, LA		P12, P16		2	Existing review
Collins(27)	2011	Evaluation report	To evaluate the effectiveness of an 'outreach' delivery model for NHSHCs	Mixed methods	n/a	Medway	PCT		P17	A4	2	NHSHC website
Corlett(28)	2016	Journal article	To assess the findings of NHSHCs delivered in pharmacy settings, referrals to GPs and client views	Mixed methods	n/a	Lewisham	Transition, LA			A2, A13, A15, A25, A35, A38	6	Existing review
Coward(29)	2020	Conference presentation	To describe processes linking NHSHCs with	n/a	n/a	Dorset	LA	C2, C7, C8	P17, P19, P26, P27, P28		8	NHSHC website

For peer review only

			local services that aim to promote physical activity									
Cupit(30)	2018	PhD thesis	To explore how patients' and healthcare professionals' knowledge and practices about CVD prevention are socially organised	Ethnography	n/a	Unknown	LA	C8, C13, C14	P2, P4, P9, P10, P20, P24	A11, A23, A25, A27, A31, A35, A38, A44	17	NHSHC website
Dalton(31)	2011	Journal article	To examine the uptake of NHSHCs and statin prescribing	Cross-sectional	n/a	Ealing	PCT		P2, P3		2	Existing review
Derbyshire County Council(32)	2015	Evaluation report (Health Equity Audit)	To identify health inequalities in relation to the NHSHC programme	Cross-sectional	Commissioner	Derbyshire	LA	C9, C10	P2, P12, P15, P16, P17, P20, P22, P25	A35	11	NHSHC website
Edmans(33)	2013	Other report	To describe local training provision in relation to the dementia component of NHSHCs	n/a	Providers	Southwark	LA		P20		1	NHSHC website
Fenton(34)	2018	Conference presentation	To describe local CVD prevention work and NHSHCs	Cross-sectional	n/a	Southwark	LA		P1, P3		2	NHSHC website
Forster(35)	2015	Journal article	To evaluate the 'yield' of NHSHCs (in relation to identification of	Retrospective cohort	n/a	England	PCT, Transition, LA		P2, P6, P12, P16		4	Existing review

For peer review only

			risk factors, diagnoses and prescribing)									
Forsyth(36)	2012	Conference abstract	To test a delivery model using pharmacists to deliver health checks to the South Asian community (NB describes checks delivered in Scotland)	Mixed methods	n/a	Glasgow	PCT			A17	1	Searches - Embase
Frazer(37)	2020	Conference presentation	To describe the delivery of NHSHCs with integrated lifestyle services	n/a	Commissioners	West Sussex	LA	C3			1	NHSHC website
Gidlow(38)	2020	Journal article	To examine the content of NHSHC, patient-practitioner communication balance and differences when using QRISK2 versus JBS3 CVD risk calculators	Observation (video recorded NHSHCs)	n/a	West Midlands	LA		P10, P20	A6, A13, A39	5	Searches - MEDLINE
Gidlow(39)	2021 (1)	Research report	To explore the extent of physical activity and alcohol measurement in the NHSHC	Observation (video recorded NHSHCs)	n/a	West Midlands	LA		P20		1	NHSHC website

For peer review only

Gidlow(40)	2021 (2)	Research report	To explore practitioner and patient understanding of CVD risk, associated advice or treatment and the response of patients to the NHSHC supported by the QRISK2 or JBS3 risk calculators	Mixed methods	n/a	West Midlands	LA		P10	A9, A10, A13, A29	4	Search alerts
Graley(41)	2011	Journal article	To describe a 'postcode lottery' effect in relation to NHSHCs	Cross-sectional	Commissioner	North West London	PCT	C9, C10, C13, C14	P12, P13		6	Existing review
Greaves(42)	2015	Journal article	To assess the feasibility of delivering an intervention to promote healthy eating and physical activity and of conducting a full scale RCT	Pilot RCT	n/a	Bath	PCT		P28	A30, A37, A40	4	Searches - MEDLINE
Green(43)	2018	Evaluation report	To understand the NHSHC patient journey from invitation through to behaviour change or clinical intervention	Mixed methods	Attendees	East Sussex	LA	C7	P10, P24	A1, A8, A12, A24, A29, A35	9	Stakeholder group
Gregory(44)	2018	Conference presentation	To describe the role of healthcare professionals	n/a	n/a	England	LA		P25		1	NHSHC website

			in treatment of tobacco dependency									
Gulliford(45)	2018	Journal article	To compare CVD risk scores for 'invited' and 'opportunistic' NHSHCs	Retrospective cohort	n/a	Lambeth and Lewisham	Transition, LA		P11		1	Existing review
Hardman(46)	2014	Evaluation report	To describe local delivery of an NHSHC programme with Health Trainers	n/a	Various	Bolton	PCT	C3	P7, P14, P19	A13, A30	6	NHSHC website
Haringay Council(47)	2012	Evaluation report	Scrutiny review' focused on men's health needs and life expectancy gap	Mixed methods	n/a	Haringay	PCT	C7		A10, A13, A14, A16, A30, A37	7	NHSHC website
Hawking(48)	2018	Conference poster	To describe a visual risk communication tool designed for use in NHSHCs	n/a	Providers, attendees	Newham	LA			A44	1	NHSHC website
Hawking(49)	2019	Journal article	To explore patient perspectives and experiences of a personalised risk report during an NHSHC	Qualitative interview study	Attendees	Newham	LA		P28	A1, A9, A10, A18, A19, A20, A40, A44	9	Existing review
Hinde(50)	2017	Journal article	To assess the cost-effectiveness of NHSHCs	Economic evaluation	n/a	England	LA	C12, C13			2	Existing review

For peer review only

Homer(51)	2015	Journal article	To determine the characteristics of patients prescribed statins for primary prevention according to their CVD risk	Cross-sectional	n/a	Newham, City & Hackney, Tower Hamlets (London)	LA		P3		1	Serendipity
Honey(52)	2013	Journal article	To identify the attitudes of primary healthcare professionals towards the delivery of lifestyle advice in NHSHCs	Q methodology	Providers	Leeds	PCT		P2, P6, P7, P18, P22, P24, P27		7	Searches - MEDLINE
Honey(53)	2015	Journal article	To examine the perspectives of patients identified as being at 'high risk' of CVD events	Qualitative interview study	Attendees	Leeds	PCT		P27	A2, A3, A4, A5, A7, A9, A10, A13, A19, A20, A22, A27, A29	14	Searches - MEDLINE
Hooper(54)	2014	Journal article	To estimate the case detection of five health conditions by NHSHCs	Cross-sectional	Attendees	Warwickshire	PCT	C10			1	Existing review
Hyseni(55)	2020	Journal article	To report the results of a workshop that aimed to facilitate engagement with stakeholders who will be involved in co-	Qualitative interview study	Commissioners, Providers, Policymakers	Various	LA	C5, C7, C11, C12, C13, C14	P12		7	Searches - MEDLINE

For peer review only

			production of an NHSHC modelling tool for commissioners									
Ismail(56)	2015	Journal article	To explore the challenges and barriers faced by staff involved in the delivery of the NHSHC	Qualitative interview study	Providers	Leeds	LA	C1, C7, C9, C14	P6, P10, P12	A35, A36, A44	10	Existing review
Ismail(57)	2016	Journal article	To provide an insight into the process of patients receiving NHSHCs and determine the extent to which they were supported to reduce CVD risk through behaviour change	Qualitative interview study	Attendees	Leeds	PCT		P2, P27	A2, A6, A8, A11, A12, A30, A31, A38	10	Existing review
Ismail(58)	2019	Research report (NIHR)	To test the clinical and cost-effectiveness of an 'enhanced lifestyle motivational interviewing' intervention for patients at high risk of CVD in group and individual	RCT	n/a	South London	LA		P6, P7	A6, A16, A17, A26, A27, A30, A31, A32, A34, A40, A44	13	Searches - WoS

			settings, compared with usual care									
Jones(59)	2020	Conference poster	To describe local CVD prevention work and NHSHCs	n/a	n/a	Telford	LA / CCG	C1	P6, P17	A14	4	NHSHC website
Kearney(60)	2015	Conference presentation	To describe factors that influence GP engagement with NHSHCs and potential solutions	n/a	n/a	n/a	LA	C4	P1		2	NHSHC website
Kennedy(61)	2019	Journal article	To evaluate uptake, risk factor detection and management from the NHSHC	Quasi-RCT	n/a	Hampshire	PCT, Transition, LA		P13		1	Existing review
Kirkpatrick(62)	2016	Conference presentation	To describe staff development and training for NHSHC providers	n/a	Providers	Salford	LA		P8		1	NHSHC website
Krska(63)	2010	Evaluation report	To evaluate a CVD screening service offered in pharmacy settings and obtain views of prospective users about	Mixed methods	n/a	Sefton	PCT		P25	A2	2	NHSHC website

For peer review only

			the acceptability of the service									
Krska(64)	2013	Conference abstract	To compare the views of the general public, pharmacists and patients invited to the NHSHC and to compare experiences of NHSHCs delivered by pharmacies and general practices	Survey and qualitative interviews	Providers, attendees	Sefton	PCT			A14	1	Searches - Embase
Krska(65)	2014	Journal article	To explore the views and experiences of patients with potentially high-CVD risk	Survey	Attendees	Sefton	PCT		P7, P10	A15	3	Existing review
Krska(66)	2016 (1)	Journal article	To seek the views of GPs and practice managers on NHSHC implementatio n	Survey	Providers	Sefton	PCT	C9	P10, P13		3	Existing review
Krska(67)	2016 (2)	Journal article	To evaluate NHSHC implementatio n in relation to data recording, advice provided, referrals,	Cross-sectional	Providers	Sefton	PCT	C10			1	Existing review

For peer review only - http://bmjopen.bmj.com/site/about/guidelines.xhtml

			prescribing and new diagnoses									
Kumar(68)	2015	Conference presentation	To describe work to improve GP engagement with NHSHCs	n/a	n/a	Stoke on Trent, Havering (London)	LA	C1			1	NHSHC website
Lake(69)	2010	Conference presentation	To describe a community based NHSHC service	n/a	n/a	Camden	PCT	C11	P19, P28	A5, A14	4	NHSHC website
Lambert(70)	2016	Journal article	To assess indicators of programme reach available to local service commissioners	Assessment of indicators	n/a	NE England	PCT, Transition		P12		1	Existing review
Lennon(71)	2020	Conference presentation	To describe local responses to Covid-19 in relation to NHSHCs and social prescribing	n/a	n/a	Redbridge	LA	C6			1	Stakeholder group
Liverpool City Council(72)	2020	Conference poster	To describe the local Health Trainer service available to NHSHC attendees	n/a	n/a	Liverpool	LA		P6, P8, P19, P26, P27, P28	A6, A14, A16, A30, A36, A40	12	NHSHC website
Local Government Association(73)	2015	Other report	Report on the transfer of public health responsibilities to LAs	n/a	Commissioners	Various	LA	C1, C5, C8, C11, C12, C13			6	Existing review

For peer review only

Local Authority A(74)[, b]	2019	Evaluation report	Evaluation of local NHSHC programme	Mixed methods	Commissioners	[Redacted]	LA	C1, C2, C11	P16, P17	A15	6	Survey respondent
Local Authority B(75)[, b]	2019	Evaluation report	Evaluation of local NHSHC programme	Mixed methods	Commissioners	[Redacted]	LA	C7, C11, C12			3	Survey respondent
Local Authority C(76)[, b]	nd	Working document	Report describing quality assurance considerations in relation to NHSHCs	n/a	Commissioners	[Redacted]	LA	C1, C2, C4, C7	P9		5	Survey respondent
Local Authority C(77)[, b]	2019	Evaluation report	To measure, monitor and report on performance and quality of NHSHCs against a quality framework	Mixed methods	Commissioners	[Redacted]	LA	C2, C7, C10, C12			4	Survey respondent
London Borough of Bromley(78)	2018	Conference presentation	To assess the prevention of diabetes through NHSHCs	Cross-sectional	n/a	Bromley	LA	C14	P8, P25		3	NHSHC website
Loo(79)	2011	Conference abstract	To derive information on community pharmacists' activities and attitudes in relation to NHSHCs	Survey	Providers	Various	PCT		P13		1	Existing review
Lumley(80)	2015	Conference presentation	To describe the role of the NHSHC in addressing high blood pressure	n/a	Commissioners, providers	Blackpool	LA	C1, C7			2	NHSHC website

For peer review only

Maddern(81)	2020	Conference abstract	To describe the role of local political scrutiny in developing and improving NHSHCs	n/a	Commissioners	Wiltshire	LA	C11			1	NHSHC website
Martin(82)	2011	Practitioner article	To describe challenges in delivery of NHSHCs	n/a	Provider	East Sussex	PCT	C13	P14		2	Searches - CINAHL
McDermott(83)	2015	Conference presentation	Summary of research exploring the implementation of NHSHCs in primary care settings	Qualitative interview study	Providers	Lambeth and Lewisham	PCT, Transition	C9	P1, P10, P13		4	NHSHC website
McMillan(84)	2018	Journal article	Report on a workshop with members of the public to inform design of a 'digital adjunct' intervention for the NHSHC	Co-production	Attendees	Manchester	LA			A6, A44	2	Searches - MEDLINE
McNaughton (85)	2011	Journal article	To evaluate delivery of the NHSHC in community pharmacies	Qualitative interview study	Providers	Tees Valley	PCT	C13	P20, P27	A14, A15	5	Existing review
McNaughton (86)	2014	Journal article	To understand factors that influenced adherence to medication and advice in 'high risk' patients identified by the NHSHC	Qualitative interview study	Attendees	North East England	PCT			A3, A9, A10, A25, A27, A28, A35, A38, A40, A42	10	Existing review

For peer review only

Mills(87)	2017	Journal article	To synthesise data concerning the views of commissioners, managers and healthcare professionals towards the NHSHC	Systematic review	Commissioners, providers	n/a	n/a		P10, P13		2	Searches - MEDLINE
Nahar(88)	2020	Journal article	Protocol for a mixed methods implementation study of a community based CVD risk assessment and coaching intervention	Mixed methods (protocol)	n/a	Sussex, Nottingham	LA			A14	1	Searches - MEDLINE
Nasir(89)	2018	Conference abstract	To describe a local project that aimed to increase NHSHC uptake and referrals to local lifestyle services	Cross-sectional	n/a	Kingston (London)	LA			A35, A37	2	NHSHC website
Newman(90)	2018	Conference presentation	To describe Health Coaching techniques	n/a	n/a	n/a	LA		P27		1	NHSHC website
NHS Greenwich(91)	2011	Evaluation report	To assess the outcomes of a community-based NHSHC delivery model	Mixed methods	n/a	Greenwich	PCT		P14	A4, A13, A17	4	Existing review
NHS Midlands and East(92)	2011	Evaluation report	To evaluate a pilot programme aiming to	Mixed methods	n/a	Bedfordshire; Great Yarmouth	PCT	C2, C7	P1, P7, P10, P17, P19	A40	8	NHSHC website

For peer review only

			deliver lifestyle interventions through the NHSHC			and Waveney						
NICE(93)	2014	Guidance	Guidance for local authorities on commissioning and delivery of the NHSHC	n/a	Commissioners	England	LA	C11			1	Searches - HMIC
Nicholas(94)	2012	Journal article	To evaluate the organisation of the NHSHC in general practices	Survey	Providers	Lambeth and Lewisham	PCT		P2		1	Existing review
O'Flaherty(95)	2021	Research report	To develop a model for commissioners to quantify cost-effectiveness and potential for equitable population health again of the NHSHC	Mixed methods	Commissioners	Various	LA	C7			1	Search alerts
Onyia(96)	2016	Conference presentation	To describe the impact of Health Trainers on uptake of lifestyle services after NHSHCs	Cross-sectional	n/a	Halton	LA		P6, P8, P25, P26		4	NHSHC website
Oswald(97)	2010	Evaluation report	To evaluate the implementation ad outcomes of the 'Tees Vascular Assessment Programme'	Mixed methods	n/a	Tees Valley	PCT	C8, C9, C13, C14	P1, P2, P7, P11, P14, P28	A1, A2, A4, A9, A13, A10, A18, A28, A29, A31, A32, A33, A40, A41, A42, A43	26	Existing review

			(precursor to NHSHC)									
Palladino(98)	2020	Journal article	To assess associations between coverage of the NHSHC and detection and management of incident cases of non-diabetic hyperglycaemia and type 2 diabetes	Retrospective cohort	n/a	England	PCT		P5		1	Searches - MEDLINE
Patel(99)	2020	Journal article	To describe the uptake and outputs of the NHSHC programme	Cross-sectional	n/a	England	Transition, LA		P3, P12		2	Searches - MEDLINE
Paxton(100)	2020	Journal article	To assess the fidelity of delivery of NHSHCs in general practice	Fidelity assessment	n/a	East of England	PCT		P20, P21		2	Searches - MEDLINE
Perkins(101)	2020	Conference presentation	Presentation describing CVD prevention initiatives	n/a	n/a	England	LA			A2, A38	2	NHSHC website
Perry(102)	2014	Journal article	To explore experiences of engaging with a community-based NHSHC	Qualitative interview study	Attendees	Knowsley	PCT		P14, P28	A2, A4, A7, A13, A18, A25, A35, A38, A40, A44	12	Existing review

For peer review only

Public Health England(103)	2020	Guidance	Best Practice Guidance for commissioners and providers	n/a	n/a	England	LA		P12	A12	2	NHSHC website
Rawlinson(104)	2019	Journal article	To evaluate an 'enhanced health promotion service' in a physiotherapy-led musculoskeletal service providing NHSHCs and diabetes checks	Mixed methods	n/a	Salford	LA	C6	P6, P7, P8, P14, P17, P19, P26		8	Searches - CINAHL
Research Works(105)	2013	Evaluation report	To assess commissioners' and providers' experiences of the NHSHC and gain an understanding of the engagement of public health professionals with NHSHCs	Qualitative interview study	Commissioners, providers	Various	PCT, Transition	C1, C4, C7, C11, C13	P7, P9, P10, P13, P14, P19	A13	12	Existing review
Richardson(106)	2016	News article	Article responding to research demonstrating lower coverage of NHSHCs and limited evidence of effectiveness of NHSHCs	n/a	n/a	n/a	LA		P18	A16	2	Searches - CINAHL

For peer review only

Riley(107)	2015	Journal article	To examine the feasibility and acceptability of community outreach NHSHCs targeted at the Afro-Caribbean community	Ethnography	Providers, attendees	Bristol	LA		P10	A2, A3, A13, A14, A16, A17, A21, A23, A25, A26, A40, A44	13	Existing review
Riley(108)	2016	Journal article	To examine the experiences of patients attending and healthcare professionals conducting NHSHCs	Qualitative interview study	Providers, attendees	Bristol	LA		P4, P8, P10, P27	A1, A3, A11, A19, A20, A21, A22, A25, A33, A35, A38, A44	16	Existing review
Robson(109)	2015	Journal article	To describe implementation and results of the NHSHCs	Cross-sectional	n/a	City & Hackney, Newham, Tower Hamlets	PCT		P9		1	Existing review
Robson(110)	2017	Journal article	To describe the coverage and impact of NHSHCs on cardiovascular risk management and identification of new comorbidities	Retrospective cohort	n/a	City & Hackney, Newham, Tower Hamlets	PCT, Transition, LA		P9		1	Existing review

Saramunee(111)	2015	Journal article	To explore the experience of and willingness to use seven pharmacy public health services related to cardiovascular risk among the general public in England (including the NHSHC)	Mixed methods	Attendees	Sefton				A15, A29	2	Searches - MEDLINE
Shaw(112)	2015	Journal article	To explore health care professionals' and patients' experiences of delivering and receiving the NHSHC	Qualitative interview study	Providers, attendees	Birmingham	PCT	C14	P8, P12, P23, P24, P27	A2, A3, A4, A6, A9, A24, A26, A32, A35, A36, A38, A40	18	Existing review
Shaw(113)	2016	Journal article	To evaluate the implementation of the NHSHC from the perspective of GPs	Qualitative interview study	Providers	Birmingham	PCT		P1, P5, P6, P9, P10, P11, P13, P17, P20, P21, P26, P27	A1, A6, A10, A12, A20, A30, A39	19	Existing review
Simon(114)	2020	Conference presentation	Presentation describing physical activity interventions for prevention and management of health conditions	n/a	n/a	England	LA		P26		1	NHSHC website

For peer review only - http://bmjopen.bmj.com/site/about/guidelines.xhtml

Solutions Strategy(115)	2017	Evaluation report	To evaluate the pilot and assess the feasibility of extending the NHSHC to include a dementia risk reduction component	Mixed methods	n/a	Birmingham, Bury, Manchester, Southampton			P26	A13, A15, A26	4	Searches - HMIC
Strutt(116)	2011	PhD thesis	To investigate patients' experiences of and responses to NHSHCs	Qualitative interview study / ethnography	Attendees	n/a	PCT		P1, P16	A2, A5, A6, A7, A11, A13, A15, A20, A21, A22, A25, A26, A29, A30, A34, A35, A44	19	Existing review
Tanner(117)	2020	Research report	To update PHE-commissioned rapid evidence synthesis and summarise evidence to address several research questions relating to the implementation of NHSHCs	Systematic review	Various	n/a	n/a		P2		1	NHSHC website
Thompson(118)	2016	Conference presentation	Presentation describing PHE's 'StARS' framework for assessment of NHSHCs	n/a	Commissioners	England	LA	C11			1	NHSHC website
Thompson(119)	2019	Conference abstract	Summary of implementation and outcomes of an integrated	Cross-sectional	Commissioner	Slough	LA	C3	P19		2	NHSHC website

For peer review only

			cardiovascular service									
Trueland(120)	2013	News article	Article based around an interview with NHSHC supporter Professor Michael Kirby	n/a	Commissioners	n/a	LA	C8			1	Searches - HMIC
Turner(121)	2013	Journal article	To evaluate variation in statin take up following risk assessment in the NHSHC	Qualitative interview study	Attendees	Nottingham	PCT			A15, A28, A41, A42, A43	5	Searches - HMIC
Usher-Smith(122)	2017	Research report	To provide a rapid synthesis of published research evidence on NHSHCs	Systematic review	n/a	n/a	n/a		P8		1	NHSHC website
Visram(123)	2012	Evaluation report	To investigate the implementation and accessibility of the 'health trainer community check' service	Mixed methods	Various	Durham	PCT, Transition	C1, C5, C9	P6, P12, P19, P27	A2, A3, A4, A5, A31, A32, A33	14	NHSHC website
Williams(124)	2018	Conference presentation	Presentation describing physical activity training and use of the GPPAQ tool to assess physical activity levels	n/a	n/a	England	LA		P21, P26		2	NHSHC website

nd=no date

For peer review only

[a] *Commissioning period here refers to whether the data included in the document relate to the period when NHSHCs were commissioned by PCTs or LAs. Documents contributing data that spans the 'transition' period (from mid-2012 to 2013) are also noted.*

[b] *Identifying details of LAs have been removed where unpublished material was shared by survey respondents*

For peer review only

Reference list (included documents)

1. Alageel S. Implementing multiple behaviour change interventions after health checks in primary care: a qualitative study. Cardiovascular Disease Prevention Conference 2018: Getting serious about prevention: reducing variation and optimizing care London, UK: NHS England; 2018.
2. Alageel S, Gulliford MC, McDermott L, Wright AJ. Implementing multiple health behaviour change interventions for cardiovascular risk reduction in primary care: a qualitative study. BMC Fam Pract. 2018;19(1):171.
3. Alageel S, Gulliford MC, Wright A, Khoshaba B, Burgess C. Engagement with advice to reduce cardiovascular risk following a health check programme: A qualitative study. Health Expect. 2020;23(1):193-201.
4. Alford S, Perry C. Knowsley at Heart community NHS health checks: Behaviour change evaluation. 2010. Accessed
5. Al-Osaimi A. Improving lifestyle support following the NHS Health Check programme; a whole system approach. Cardiovascular Disease Prevention Conference 2020: proactive, predictive, personalised Kia Oval, London: NHS England; 2020.
6. Artac M, Dalton AR, Majeed A, Car J, Millett C. Effectiveness of a national cardiovascular disease risk assessment program (NHS Health Check): results after one year. Prev Med. 2013;57(2):129-34.
7. Atkins L, Stefanidou C, Chadborn T, Thompson K, Michie S, Lorencatto F. Influences on NHS Health Check behaviours: a systematic review. BMC Public Health. 2020;20(1):1359.
8. Baker C, Loughren E, Crone D. Evaluation of the Gloucestershire NHS Health Check Programme, for the period July 2011 to July 2012. 2014. Available from: https://www.healthcheck.nhs.uk/commissioners-and-providers/evidence/local-evaluation/. Accessed
9. Baker C, Loughren EA, Crone D, Kallfa N. Patients' perceptions of a NHS Health Check in the primary care setting. Qual Prim Care. 2014;22:232-37.
10. Baker C, Loughren EA, Crone D, Kallfa N. Perceptions of health professionals involved in a NHS Health Check care pathway. Practice Nursing. 2015;26(12):608-12.
11. Baker C, Loughren EA, Crone D, Kallfa N. A process evaluation of the NHS Health Check care pathway in a primary care setting. J Public Health (Oxf). 2015;37(2):202-9.
12. Bell K. Integrating an outreach NHS Health Check Programme into a Lifestyle Interventions Hub. Cardiovascular Disease Prevention Conference 2019: Saving Hearts and Minds Together Manchester, UK2019.
13. Boase S, Mason D, Sutton S, Cohn S. Tinkering and tailoring individual consultations: how practice nurses try to make cardiovascular risk communication meaningful. J Clin Nurs. 2012;21(17-18):2590-8.
14. Boseley R. Personalised and proactive CVD Prevention: NHS Health Checks and Integrated Lifestyle services in East Sussex. Cardiovascular Disease Prevention Conference 2020: proactive, predictive, personalised Kia Oval, London: NHS England; 2020.
15. Brutus L, Fluke R. Croydon NHS Health Check Programme: Review and Options Appraisal. 2013. Available from: https://www.healthcheck.nhs.uk/commissioners-and-providers/evidence/local-evaluation/. Accessed
16. Burgess C. Engagement with advice to reduce cardiovascular risk following an NHS Health Check. NHS Health Check Conference: Getting Serious about Prevention London, UK: NHS England; 2016.

For peer review only

17. Burke D, Kirby H. Leeds patient insight and engagement from vulnerable groups: how to reduce inqualities. NHS Health Check 2015 - Improvement through collaboration 2015.
18. Carter P, Bodicoat DH, Davies MJ, Ashra NB, Riley D, Joshi N, et al. A retrospective evaluation of the NHS Health Check Programme in a multi-ethnic population. J Public Health (Oxf). 2016;38(3):534-42.
19. Centre for Public Scrutiny. Checking the nation’s health: the value of council scrutiny. London: CfPS, 2014; 2014. Available from: http://www.cfps.org.uk/includes/scripts/force_download.php?file=../../domains/cfps.org.uk/local/media/downloads/CfPS_Nations_Health_final_online.pdf. Accessed
20. Chatterjee R, Chapman T, Brannan MG, Varney J. GPs' knowledge, use, and confidence in national physical activity and health guidelines and tools: a questionnaire-based survey of general practice in England. Br J Gen Pract. 2017;67(663):e668-e75.
21. Chipchase L, Hill P, Waterall J. NHS Birmingham East & North. An insight into the NHS Health Check Programme in Birmingham: Summary Report. 2011. Accessed
22. Clarke R, Brierley A. Using NHS Health Checks data to galvanise public health prevention. Cardiovascular Disease Prevention Conference 2020: proactive, predictive, personalised Kia Oval, London: NHS England; 2020.
23. Cochrane T, Davey R, Iqbal Z, Gidlow C, Kumar J, Chambers R, et al. NHS health checks through general practice: randomised trial of population cardiovascular risk reduction. BMC Public Health. 2012;12:944.
24. Cochrane T, Gidlow CJ, Kumar J, Mawby Y, Iqbal Z, Chambers RM. Cross-sectional review of the response and treatment uptake from the NHS Health Checks programme in Stoke on Trent. J Public Health (Oxf). 2013;35(1):92-8.
25. Coffey M, Cooper A, Brown T, Cook P, Clarke-Cornwell A. Vascular health checks in Salford: an exploration using FARSITE data. University of Salford,; 2014. Accessed
26. Coghill N, Garside L, Montgomery AA, Feder G, Horwood J. NHS health checks: a cross- sectional observational study on equity of uptake and outcomes. BMC Health Serv Res. 2018;18(1):238.
27. Collins K-a. Evaluation of the NHS Health Check Outreach Programme in Medway. NHS Medway; 2011. Available from: https://www.healthcheck.nhs.uk/commissioners-and-providers/evidence/local-evaluation/. Accessed
28. Corlett SA, Krska J. Evaluation of NHS Health Checks provided by community pharmacies. J Public Health (Oxf). 2016;38(4):e516-e23.
29. Coward C. Understanding and accessing the local physical activity system. Cardiovascular Disease Prevention Conference 2020: proactive, predictive, personalised Kia Oval, London: NHS England,; 2020.
30. Cupit C. An ethnographic study of cardiovascular disease prevention: the social organisation of measures, knowledge, interventions and tensions in English general practice: University of Leicester; 2018.
31. Dalton AR, Bottle A, Okoro C, Majeed A, Millett C. Uptake of the NHS Health Checks programme in a deprived, culturally diverse setting: cross-sectional study. J Public Health (Oxf). 2011;33(3):422-9.
32. Derbyshire County Council, Services PHIaK. Health Equity Audit: NHS Health Check Programme in Derbyshire County. 2015. Available from: https://www.healthcheck.nhs.uk/commissioners-and-providers/evidence/local-evaluation/. Accessed

For peer review only

33. Edmans T. Training for the dementia component of the NHS Health Check, NHS Southwark. Southwark Council,; 2013. Available from: https://www.healthcheck.nhs.uk/commissioners-and-providers/evidence/case-studies/. Accessed
34. Fenton K. Getting serious about preventing cardiovascular disease. Cardiovascular Disease Prevention Conference 2018: Getting serious about prevention: reducing variation and optimizing care London, UK2018.
35. Forster AS, Dodhia H, Booth H, Dregan A, Fuller F, Miller J, et al. Estimating the yield of NHS Health Checks in England: a population-based cohort study. J Public Health (Oxf). 2015;37(2):234-40.
36. Forsyth P, Ali S, Ameen M, Khan R, Khan F, Sheikh AR, et al. Pharmacist-led anticipatory care for the South Asian community. International Journal of Pharmacy Practice. Royal Pharmaceutical Society, RPS Annual Conference 2012. Birmingham United Kingdom.

(var.pagings). 20: Pharmaceutical Press; 2012. p. 88-9.
37. Frazer H. Implementing NHS Health Checks within a non-clinical integrated lifestyle service. Cardiovascular Disease Prevention Conference 2020: proactive, predictive, personalised Kia Oval, London: NHS England; 2020.
38. Gidlow CJ, Ellis NJ, Cowap L, Riley VA, Crone D, Cottrell E, et al. Quantitative examination of video-recorded NHS Health Checks: comparison of the use of QRISK2 versus JBS3 cardiovascular risk calculators. BMJ Open. 2020;10(9):e037790.
39. Gidlow C, Riley V. Physical activity and alcohol measurement in NHS Health Checks. Staffordshire University, Public Health England; 2021. Available from: https://www.healthcheck.nhs.uk/commissioners-and-providers/evidence/. Accessed
40. Gidlow CJ, Ellis NJ, Cowap L, Riley V, Crone D, Cottrell E, et al. Cardiovascular disease risk communication in NHS Health Checks using QRISK®2 and JBS3 risk calculators: the RICO qualitative and quantitative study. Health Technol Assess. 2021;25(50).
41. Graley C, Katherine MF, McCoy DC. Postcode Lotteries in Public Health - The NHS Health Checks Programme in North West London. BMC Public Health. 2011;11(738).
42. Greaves C, Gillison F, Stathi A, Bennett P, Reddy P, Dunbar J, et al. Waste the waist: a pilot randomised controlled trial of a primary care based intervention to support lifestyle change in people with high cardiovascular risk. Int J Behav Nutr Phys Act. 2015;12:1.
43. Green K, Forshaw A, D'Souza H, Macherianakis A, MEL Research. NHS Health Check Patient Journey Evaluation: Final Evaluation Report. East Sussex County Council Public Health, MEL Research; 2018. Accessed
44. Gregory A, Chowdary Q, Tobacco Control Programme P. The role of healthcare professionals in treating tobacco dependency: opportunities to reduce CVD for commissioners and practitioners. Cardiovascular Disease Prevention Conference 2018: Getting serious about prevention: reducing variation and optimizing care London, UK2018.
45. Gulliford MC, Khoshaba B, McDermott L, Cornelius V, Ashworth M, Fuller F, et al. Cardiovascular risk at health checks performed opportunistically or following an invitation letter. Cohort study. J Public Health (Oxf). 2018;40(2):e151-e6.
46. Hardman L. The BIG Bolton Health Check. 2014 October 2014. Available from: https://www.healthcheck.nhs.uk/commissioners-and-providers/evidence/case-studies/. Accessed
47. Haringay Council. Scrutiny Review Men's Health: Getting to the Heart of the Matter. A review by the Overview and Scrutiny Committee. 2012. Available from: https://www.healthcheck.nhs.uk/commissioners-and-providers/evidence/local-evaluation/. Accessed

For peer review only

48. Hawking M. The CVD Risk Report, an intervention to support CVD risk communication in NHS Health Checks: results from the pilot trial. Cardiovascular Disease Prevention Conference 2018: Getting serious about prevention: reducing variation and optimizing care London, UK: NHS England; 2018.
49. Hawking MKD, Timmis A, Wilkins F, Potter JL, Robson J. Improving cardiovascular disease risk communication in NHS Health Checks: a qualitative study. BMJ Open. 2019;9(8):e026058.
50. Hinde S, Bojke L, Richardson G, Retat L, Webber L. The cost-effectiveness of population Health Checks: have the NHS Health Checks been unfairly maligned? Journal of Public Health: From Theory to Practice. 2017;25(4):425-31.
51. Homer K, Boomla K, Hull S, Dostal I, Mathur R, Robson J. Statin prescribing for primary prevention of cardiovascular disease: a cross-sectional, observational study. Br J Gen Pract. 2015(e538).
52. Honey S, Bryant LD, Murray J, Hill K, House A. Differences in the perceived role of the healthcare provider in delivering vascular health checks: a Q methodology study. BMC Fam Pract. 2013;14:172.
53. Honey S, Hill K, Murray J, Craigs C, House A. Patients' responses to the communication of vascular risk in primary care: a qualitative study. Prim Health Care Res Dev. 2015;16(1):61-70.
54. Hooper J, Chohan P, Caley M. Case detection of disease by NHS Health Checks in Warwickshire, England and comparison with predicted performance. Public Health. 2014;128(5):475-7.
55. Hyseni L, Guzman-Castillo M, Kypridemos C, Collins B, Schwaller E, Capewell S, et al. Engaging with stakeholders to inform the development of a decision-support tool for the NHS health check programme: qualitative study. BMC Health Serv Res. 2020;20(1):394.
56. Ismail H, Kelly S. Lessons learned from England's Health Checks Programme: using qualitative research to identify and share best practice. BMC Fam Pract. 2015;16:144.
57. Ismail H, Atkin K. The NHS Health Check programme: insights from a qualitative study of patients. Health Expect. 2016;19(2):345-55.
58. Ismail K, Stahl D, Bayley A, Twist K, Stewart K, Ridge K, et al. Enhanced motivational interviewing for reducing weight and increasing physical activity in adults with high cardiovascular risk: the MOVE IT three-arm RCT. Health Technol Assess. 2019;23(69).
59. Jones T. Telford Healthy Hearts. Cardiovascular Disease Prevention Conference 2020: proactive, predictive, personalised Kia Oval, London2020.
60. Kearney M. Engaging with GPs to help deliver the NHS Health Checks. NHS Health Check 2015 - Improvement through collaboration 2015.
61. Kennedy O, Su F, Pears R, Walmsley E, Roderick P. Evaluating the effectiveness of the NHS Health Check programme in South England: a quasi-randomised controlled trial. BMJ Open. 2019;9(9):e029420.
62. Kirkpatrick W, Eden A. Developing a resilient community & primary care based workforce to deliver the NHS Health Check. NHS Health Check Conference: Getting Serious about Prevention London, UK: NHS England; 2016.
63. Krska J, Mackridge AJ, Taylor J. An Evaluation of the Cardiovascular Screening Service provided by community pharmacies in Sefton PCT: Final Report. Liverpool John Moores University; 2010. Available from: https://www.healthcheck.nhs.uk/commissioners-and-providers/evidence/local-evaluation/. Accessed
64. Krska J, Taylor J, Du Plessis R. Views and experiences of the NHS Health Check provided in pharmacies and medical practices. International Journal of Pharmacy Practice Health Services Research and Pharmacy Practice Conference 2013. Preston United Kingdom.

For peer review only - http://bmjopen.bmj.com/site/about/guidelines.xhtml

(var.pagings). 21: Pharmaceutical Press; 2013. p. 16.
65. Krska J, du Plessis R, Chellaswamy H. Views and experiences of the NHS Health Check provided by general medical practices: cross-sectional survey in high-risk patients. J Public Health (Oxf). 2014;37(2):210-7.
66. Krska J, du Plessis R, Chellaswamy H. Views of practice managers and general practitioners on implementing NHS Health Checks. Prim Health Care Res Dev. 2016;17(2):198-205.
67. Krska J, du Plessis R, Chellaswamy H. Implementation of NHS Health Checks in general practice: variation in delivery between practices and practitioners. Prim Health Care Res Dev. 2016;17(4):385-92.
68. Kumar J. GP Engagement "If they know you, it's harder for them to say no". NHS Health Check 2015 - Improvement through collaboration 2015.
69. Lake G. The Healthy Heart Centre: NHS Camden's approach to reducing Health Inequalities. NHS Camden; 2010. Available from: https://www.healthcheck.nhs.uk/commissioners-and-providers/evidence/local-evaluation/. Accessed
70. Lambert MF. Assessing potential local routine monitoring indicators of reach for the NHS health checks programme. Public Health. 2016;131:92-8.
71. Lennon S, London Borough of Redbridge. Supporting LTC Prevention During the COVID-19 Pandemic: Improving NHS Health Checks and Social Prescribing Initiatives (Presentation). 2020.
72. Liverpool City Council. LiveWire Liverpool Health Trainers. Cardiovascular Disease Prevention Conference 2020: proactive, predictive, personalised Kia Oval, London2020.
73. Local Government Association. Public health transformation twenty months on: adding value to tackle local health needs.; 2015. Accessed
74. Local Authority A. The [Local Authority Borough] NHS Health Checks Programme 2019 Review [Unpublished; identifying information redacted]. 2019. Accessed
75. Local Authority B. NHS Health Checks in [Local Authority Borough] [Unpublished; identifying information redacted]. 2019. Accessed
76. Local Authority C. Health Check Quality Assurance - Working Document [Unpublished; identifying information redacted]. Accessed
77. Local Authority C. NHS Health Checks [Local area] Performance and Quality Framework 2019 [Unpublished; identifying information redacted]. 2019. Accessed
78. London Borough of Bromley. Re audit of the Prevention of Diabetes through NHS Health Checks 2014-15. Public Health England,; 2018 January 2018. Available from: https://www.healthcheck.nhs.uk/commissioners-and-providers/evidence/case-studies/. Accessed
79. Loo R, Diaper C, Salami O, Kundu M, Lalkia M, Airhiavbere E, et al. The NHS Health Check: the views of community pharmacists. Int J Pharm Pract. 2011;19(13).
80. Lumley B. Tackling high blood pressure: system wide action and the role of the NHS Health Check. NHS Health Check 2015 - Improvement through collaboration 2015.
81. Maddern S. Partnership Working - the role of local political scrutiny in driving forward the NHS Health Check Programme in Wiltshire. Cardiovascular Disease Prevention Conference 2020: proactive, predictive, personalised. Kia Oval, London: NHS England; 2020.
82. Martin H. Delivering NHS health checks to all. British Journal of Healthcare Management. 2011;17(6):250-5.
83. McDermott L. Exploring the Health Check programme in Primary Care. NHS Health Check 2015 - Improvement through collaboration 2015.

For peer review only

84. McMillan B, Fox S, Lyons M, Bourke S, Mistry M, Ruddock A, et al. Using patient and public involvement to improve the research design and funding application for a project aimed at fostering a more collaborative approach to the NHS health check: the CaVIAR project (better Care Via Improved Access to Records). Research Involv Engagem. 2018;4:18.
85. McNaughton RJ, Oswald NT, Shucksmith JS, Heywood PJ, Watson PS. Making a success of providing NHS Health Checks in community pharmacies across the Tees Valley: a qualitative study. BMC Health Serv Res. 2011;11:222.
86. McNaughton RJ, Shucksmith J. Reasons for (non)compliance with intervention following identification of 'high-risk' status in the NHS Health Check programme. J Public Health (Oxf). 2014;37(2):218-25.
87. Mills K, Harte E, Martin A, MacLure C, Griffin SJ, Mant J, et al. Views of commissioners, managers and healthcare professionals on the NHS Health Check programme: a systematic review. BMJ Open. 2017;7(11):e018606.
88. Nahar P, van Marwijk H, Gibson L, Musinguzi G, Anthierens S, Ford E, et al. A protocol paper: community engagement interventions for cardiovascular disease prevention in socially disadvantaged populations in the UK: an implementation research study. Glob Health Res Policy. 2020;5:12.
89. Nasir N. Can alternate providers improve the uptake of Health Checks among high risk populations and subsequent referrals to the local lifestyle services? Getting Serious About Cardiovascular Disease Prevention 2018: Reducing Variation and Optimising Care London, UK2018.
90. Newman P. An Introduction to Health Coaching: Better Conversations, Better Health. Cardiovascular Disease Prevention Conference 2018: Getting serious about prevention: reducing variation and optimizing care London, UK2018.
91. NHS Greenwich. Evaluation of NHS Health Check Plus Community Outreach Programme in Greenwich. 2011. Accessed
92. NHS Midlands and East. A study into increasing uptake of lifestyle changes - Report. 2011. Accessed
93. NICE. Encouraging people to have NHS Health Checks and supporting them to reduce risk factors. Manchester: NICE, 2014; 2014. Available from: http://publications.nice.org.uk/encouraging-people-to-have-nhs-health-checks-and-supporting-them-to-reduce-risk-factors-lgb15. Accessed
94. Nicholas JM, Burgess C, Dodhia H, Miller J, Fuller F, Cajeat E, et al. Variations in the organization and delivery of the 'NHS health check' in primary care. J Public Health (Oxf). 2012;35(1):85-91.
95. O'Flaherty M, Lloyd-Williams F, Capewell S, Boland A, Maden M, Collins B, et al. Modelling tool to support decision-making in the NHS Health Check programme: workshops, systematic review and co-production with users. Health Technol Assess. 2021;25(35).
96. Onyia I. Delivery of the NHS Health Check by health trainers can improve conversion into uptake of lifestyle service. NHS Health Check Conference: Getting Serious about Prevention London, UK: NHS England; 2016.
97. Oswald N, McNaughton R, Watson P, Shucksmith J. Tees Vascular Assessment Programme: Evaluation commissioned by the Tees Primary Care Trusts (PCT) from the Centre for Translational Research in Public Health. 2010. Available from: https://www.healthcheck.nhs.uk/commissioners-and-providers/evidence/local-evaluation/. Accessed
98. Palladino R, Vamos EP, Chang KC, Khunti K, Majeed A, Millett C. Evaluation of the Diabetes Screening Component of a National Cardiovascular Risk Assessment Programme in England: a Retrospective Cohort Study. Sci Rep. 2020;10(1):1231.
99. Patel R, Barnard S, Thompson K, Lagord C, Clegg E, Worrall R, et al. Evaluation of the uptake and delivery of the NHS Health Check programme in England, using primary care data from 9.5 million people: a cross-sectional study. BMJ Open. 2020;10(11):e042963.
100. Paxton B, Mills K, Usher-Smith JA. Fidelity of the delivery of NHS Health Checks in general practice: an observational study. BJGP Open. 2020;4(4).

101. Perkins C. Cardiovascular disease prevention in the 2020s. Cardiovascular Disease Prevention Conference 2020: proactive, predictive, personalised Kia Oval, London2020.
102. Perry C, Thurston M, Alford S, Cushing J, Panter L. The NHS health check programme in England: a qualitative study. Health Promot Int. 2014;31(1):106-15.
103. Public Health England. NHS Health Check Best practice guidance For commissioners and providers. 2020. Available from: https://www.healthcheck.nhs.uk/commissioners-and-providers/national-guidance/. Accessed 31st October 2019.
104. Rawlinson G. Health promotion in physiotherapy services using NHS health and diabetes checks. British Journal of Healthcare Management. 2019;25(1):22-31.
105. Research Works, Public Health England. Understanding the implementation of NHS Health Checks. 2013. Accessed
106. Richardson J. The fall in NHS Health Checks. Nursing in Practice: The Journal for Today's Primary Care Nurse. 2016(91):1-3.
107. Riley R, Coghill N, Montgomery A, Feder G, Horwood J. The provision of NHS health checks in a community setting: an ethnographic account. BMC Health Serv Res. 2015;15:546.
108. Riley R, Coghill N, Montgomery A, Feder G, Horwood J. Experiences of patients and healthcare professionals of NHS cardiovascular health checks: a qualitative study. J Public Health (Oxf). 2016;38(3):543-51.
109. Robson J, Dostal I, Madurasinghe V, Sheikh A, Hull S, Boomla K, et al. The NHS Health Check programme: implementation in east London 2009-2011. BMJ Open. 2015;5(4):e007578.
110. Robson J, Dostal I, Madurasinghe V, Sheikh A, Hull S, Boomla K, et al. NHS Health Check comorbidity and management: an observational matched study in primary care. Br J Gen Pract. 2017;67(655):e86-e93.
111. Saramunee K, Krska J, Mackridge A, Richards J, Suttajit S, Phillips-Howard P. General public's views on pharmacy public health services: current situation and opportunities in the future. Public health. 2015;129(6):705-15.
112. Shaw RL, Pattison HM, Holland C, Cooke R. Be SMART: examining the experience of implementing the NHS Health Check in UK primary care. BMC Fam Pract. 2015;16:1.
113. Shaw RL, Lowe H, Holland C, Pattison H, Cooke R. GPs' perspectives on managing the NHS Health Check in primary care: a qualitative evaluation of implementation in one area of England. BMJ Open. 2016;6(7):e010951.
114. Simon C. Physical activity for prevention and management of health conditions and practitioner resources. Cardiovascular Disease Prevention Conference 2020: proactive, predictive, personalised Kia Oval, London: NHS England,; 2020.
115. Solutions Strategy Research Facilitation Ltd, Alzheimer's Society. NHS Health Check 40-64 dementia pilot research findings: summary research report. 2017.
116. Strutt E. Patient-centred care: Patients' experiences of and responses to the National Health Service (NHS) Health Check programme in general practice. Durham: Durham University; 2011.
117. Tanner L, Kenny R, Still M, Pearson F, Bhardwaj-Gosling R. NHS Health Check Programme Rapid Review Update. University of Sunderland, Newcastle University, Public Health England; 2020. Available from: https://www.healthcheck.nhs.uk/commissioners-and-providers/evidence/. Accessed 31st October 2020.

For peer review only

118. Thompson K. NHS Health Check StARS framework: A Systems Approach for Raising Standards. NHS Health Check Conference: Getting Serious about Prevention London, UK: NHS England; 2016.
119. Thompson S. Integrated Cardiac Disease Prevention Programme. Cardiovascular Disease Prevention Conference 2019: Saving Hearts and Minds Together. Manchester, UK2019.
120. Trueland J. 'The signs were there, but weren't picked up'. Health Serv J. 2013;123(6369):6-7.
121. Turner A. Exploratory evaluation of variation in statin take up among high risk patients in Nottingham City. Public Health. 2013.
122. Usher-Smith JA, Mant J, Martin A, Harte E, MacLure C, Meads C, et al. NHS Health Check Programme rapid evidence synthesis. Cambridge, UK: The Primary Care Unit, University of Cambridge, RAND Europe, Public Health England; 2017. Accessed 31st October 2020.
123. Visram S, Geddes L, Carr SM. Formative Evaluation of the Health Trainer Community Health Check Service in County Durham. Northumbria University, County Durham and Darlington NHS Foundation Trust; 2012. Available from: https://www.healthcheck.nhs.uk/commissioners-and-providers/evidence/local-evaluation/. Accessed
124. Williams Z. Let's talk about Physical Activity: Physical Activity Clinical Champions training and using the GPPAQ in a health check setting. Cardiovascular Disease Prevention Conference 2018: Getting serious about prevention: reducing variation and optimizing care London, UK2018.

For peer review only

Supplementary File 4

This file contains 3 tables outlining the full set of CMOCs (n=86) developed during the review. The three tables split the CMOCs up into those centred on commissioner (Table S1), provider (Table S2) and attendee (Table S3) perspectives.

Table S1 Overview of CMOCs focused on LA commissioners

	**CMOC**	**Summary of data**
Understanding and engagement with the NHSHC programme: case finding or enabling behaviour change?		
CMOC C1	When commissioners view the NHSHC as a means to improve people's lives through behaviour change (C) they will try to exert their influence over providers to ensure the programme is delivered with this in mind (O) because they believe this will maximise the potential benefits of the programme (M)	Data extracted from 12 documents:<br><br>2 research articles (one cohort study, one qualitative interview study); 4 local evaluation reports; 3 conference materials; 1 unpublished LA working document; 2 other reports focused on LA roles in NHSHCs.
CMOC C2	When commissioners view the NHSHC as a means to improve people's lives through behaviour change (C) they will try to establish closer working relationships between different parts of the wider system (the NHSHC, lifestyle services and primary care) to improve referral pathways (O) because they believe this will maximise the potential benefits of the programme (M)	Data extracted from 8 documents:<br><br>1 research article (survey); 4 local evaluation reports; 2 conference materials; 1 unpublished LA working document.

For peer review only

CMOC C3	When commissioners view the NHSHC as a means to improve people's lives through behaviour change (C) they may develop 'integrated' models of service delivery with the NHSHC delivered alongside lifestyle services (O) because they believe this will maximise the potential benefits of the programme (M)3,	Data extracted from 5 documents:<br><br>1 local evaluation report; 4 conference materials.
CMOC C4	When providers are sceptical and less engaged with the NHSHC programme (C) commissioners may be unable to exert their influence over them (O1) or establish close working relationships between different parts of the system (O2) because providers are resistant and unwilling to engage (M)	Data extracted from 3 documents:<br><br>1 local evaluation report; 1 conference presentation; 1 unpublished LA working document.
CMOC C5	When commissioners have a focus on the wider social determinants of health (C) they are more likely to commission 'alternative' NHSHC providers (i.e. to move away from a medical model based in primary care) (O) because they believe this will maximise the benefits of the programme (M)	Data extracted from 3 documents:<br><br>1 research article (qualitative interviews/workshop); 1 local evaluation report; 1 report focused on LA roles in NHSHCs.
CMOC C6	When commissioners have a focus on the wider social determinants of health (C) they may integrate NHSHC delivery with other services that address other problems (O) because they believe this will maximise the potential benefits of the programme (M)	Data extracted from 3 documents:<br><br>1 research article (mixed methods evaluation); 2 conference materials.

For peer review only

CMOC C7	When commissioners view the NHSHC as a means to improve people's lives through behaviour change (C) they are more likely to collect data related to what happens after the measurements and risk assessment are completed in a check (O) because they believe these are important data to monitor and evaluate programme performance (M)	Data extracted from 15 documents:<br><br>2 research articles (1 qualitative interviews/workshop, 1 qualitative interview study); 1 research report (mixed methods study); 7 local evaluation reports; 4 conference materials; 1 unpublished LA working document.
CMOC C8	When commissioners view the NHSHC as a means to improve people's lives through behaviour change (C) they are more likely to collect data related to the needs of the local population to inform the commissioning of lifestyle support services (O) because they believe this will maximise the benefits of the programme (M)	Data extracted from 6 documents:<br><br>1 PhD thesis (ethnography); 1 local evaluation report; 2 conference materials; 1 report focused on LA roles in NHSHCs; 1 news article.
CMOC C9	In some circumstances, commissioners may cede more control over delivery to primary care based providers (e.g. GP practices) (O) but the contexts in which this happens and the mechanisms underpinning this outcome are not clear (C, M not defined)	Data extracted from 8 documents:<br><br>3 research articles (1 qualitative interview study, 1 survey, 1 cross-sectional study); 4 local evaluation reports; 1 conference materials.
CMOC C10	In some circumstances, commissioners may focus only on mandatory data collection (monitoring invitation, uptake and coverage) (O) but the contexts in which this happen and the mechanisms underpinning this outcome are not clear (C, M not defined)	Data extracted from 5 documents:<br><br>3 research articles (3 cross-sectional studies); 2 local evaluation reports.
Practical constraints: politics and funding		

For peer review only

CMOC C11	Where there is local political support and engagement with the NHSHC programme (C), local delivery is more likely to be evaluated (O1) and developed or improved (O2) because commissioners and public health teams are empowered to focus on the programme (M)	Data extracted from 10 documents:<br><br>1 research article (qualitative interview study); 3 local evaluation reports; 3 conference materials; 2 other reports focused on LA roles in NHSHCs; 1 guidance document
CMOC C12	When funding for public health programmes is constrained (C1) and commissioners or public health teams are convinced of the NHSHCs long term effectiveness and value (C2) local delivery is more likely to be evaluated (O1) and developed or improved (O2) because these activities are considered worthwhile (M)	Data extracted from 5 documents:<br><br>2 research articles (1 qualitative interview study; 1 economic evaluation); 2 local evaluation reports; 1 other report focused on LA roles in NHSHCs.
CMOC C13	When funding for public health programmes is constrained (C) commissioners may select providers who offer the best value for money / lowest cost per NHSHC delivered (O) because they must prioritise mandatory public health programme delivery ('prescribed functions') before funding additional services (M)	Data extracted from 9 documents:<br><br>4 research articles (2 qualitative interview studies; 1 cross-sectional, 1 economic evaluation); 2 local evaluation reports; 1 PhD thesis (ethnography); 1 other report focused on LA roles in NHSHCs; 1 practitioner-facing article
CMOC C14	When funding for public health programmes is constrained (C) commissioners must prioritise funding mandatory services ('prescribed functions') (M) leading to cuts and reduced capacity in non-mandatory services (e.g. local lifestyle support services) (O)	Data extracted from 11 documents:<br><br>8 research articles (5 qualitative interview studies, 1 survey, 1 cross-sectional study, 1 systematic review); 1 local evaluation report; 1 PhD thesis (ethnography); 1 conference presentation.

For peer review only

Table S2 Overview of CMOCs focused on NHSHC providers

	**CMOC**	**Summary of data**
Understanding and engagement with the NHSHC programme: scepticism versus 'buy in'		
CMOC P1	When providers are sceptical about the NHSHC programme as a behaviour change intervention (C) they may prioritise completing the mandatory elements of the check and fail to engage with the delivery of advice, brief interventions or referrals (O) because they do not believe these will help attendees (M)	Data extracted from 13 documents:<br><br>3 research articles (2 qualitative interview studies, 1 survey); 3 local evaluation reports; 4 conference materials; 1 other report focused on LA roles in NHSHCs; 1 PhD thesis (ethnography).
CMOC P2	When providers are sceptical about the NHSHC programme as a behaviour change intervention (C1) or sceptical about the effectiveness of behaviour change to reduce the risk of cardiovascular disease (C2) they are more likely to consider medication (e.g. statins or antihypertensives) as an appropriate intervention for those assessed at higher risk (O) because they believe these will help attendees (M)	Data extracted from 11 documents:<br><br>7 research articles (2 qualitative interview studies; 1 survey; 2 cross-sectional studies; 1 cohort study; 1 Q-methodology study); 2 local evaluation reports; 1 research report (systematic review); 1 PhD thesis (ethnography).
CMOC P3	When providers (who are able to prescribe) are sceptical about the recommended thresholds for prescription (C) they are less likely to prescribe (O) because they do not believe it will help attendees (M).<br><br>*This CMOC may apply to other interventions but we lack data to confirm or refute this.*	Data extracted from 4 documents:<br><br>3 research articles (3 cross-sectional studies); 1 conference presentation.

For peer review only

CMOC P4	When providers are worried about labelling healthy people as sick (C) they may avoid offering advice, referrals or prescriptions (O) because they are concerned about the potential harms of overdiagnosis (M)	Data extracted from 3 documents:<br><br>2 research articles (2 qualitative interview studies); 1 PhD thesis (ethnography).
CMOC P5	When providers are highly engaged with preventive health care (C) they are more likely to be highly engaged with the NHSHC programme (O) as they understand it to be a useful means of reaching more patients with this agenda (M)	Data extracted from 2 documents:<br><br>2 research articles (1 qualitative interview study, 1 cohort).
CMOC P6	When providers believe that lifestyle modification is an effective means of reducing CVD risk (C) they are more likely to offer attendees advice, brief interventions or referrals to lifestyle support services (especially as a first line of action) (O) because they believe these may help attendees (M)	Data extracted from 11 documents:<br><br>6 research articles (2 qualitative interview studies; 1 cohort study; 1 mixed methods study; 1 Q-methodology study); 1 research report (RCT); 1 local evaluation report; 3 conference materials
CMOC P7	When providers buy in to the NHSHC as an opportunity to support behaviour change (C) they are more likely to offer attendees advice, brief interventions or referrals to lifestyle support services (O) because they believe these will help attendees (M)	Data extracted from 11 documents:<br><br>5 research articles (1 qualitative interview study; 1 survey; 1 mixed methods study; 1 Q-methodology study); 1 research report (RCT); 4 local evaluation reports; 1 conference presentation
CMOC P8	When providers have the specific skills they need to support the delivery of advice, brief interventions and referrals (C) they are more likely to engage	Data extracted from 10 documents:

For peer review only

	with and prioritise these activities (O) because they feel confident to deliver them during the NHSHC encounter (M)	3 research articles (2 qualitative interview studies, 1 mixed methods study); 1 research report (systematic review); 4 conference materials.
Practical constraints: time and money		
CMOC P9	When funding arrangements for delivery of checks incentivise volume of delivery (C) providers may prioritise completing mandatory elements of the check and minimise time spent delivering advice, brief interventions or offering referrals (O) because they are aware they do not have to complete these (M)	Data extracted from 6 documents:<br><br>3 research articles (1 qualitative interview study, 1 retrospective cohort, 1 cross-sectional); 1 local evaluation report; 1 PhD thesis (ethnography); 1 LA internal working document.
CMOC P10	When providers have many competing priorities (C) they may prioritise completing mandatory elements of the check and minimise time spent delivering advice, brief interventions or offering referrals (O) because of expediency (M)	Data extracted from 16 documents:<br><br>10 research articles (4 qualitative interview studies, 1 systematic review, 3 surveys, 1 ethnography, 1 observational study based on video recordings); 3 local evaluation reports; 1 research report (mixed methods); 1 PhD thesis (ethnography), 1 conference presentation
CMOC P11	When there is a focus on increasing the volume of checks delivered (C) providers may offer more checks 'opportunistically' (i.e. not in a standalone appointment) (O) as they feel this is more efficient (M)	Data extracted from 5 documents:<br><br>4 research articles (2 qualitative interview studies, 1 retrospective cohort, 1 survey, 1 mixed methods); 1 local evaluation report

For peer review only

CMOC P12	It is clear that data about non-mandatory elements of a check are often under-recorded or recorded inconsistently (O) but the contexts in which this happens and the mechanisms underpinning this outcome are not clear (C, M not defined). Potentially important contexts here include competing priorities, a focus on delivery of mandatory elements of checks, lack of monitoring and/or incentivisation to collect particular data items, or difficulty in recording certain activities within existing data collection systems	Data extracted from 15 documents:<br><br>10 research articles (3 qualitative interview studies, 4 cross-sectional studies, 1 pre/post study, 1 retrospective cohort, 1 study assessing the validity of indicators); 3 local evaluation reports; 1 guidance document; 1 conference abstract.
CMOC P13	When providers do not feel they are adequately compensated for delivering checks (C) they may prioritise completing mandatory elements of the check and minimise time spent delivering advice, brief interventions, or offering referrals (O) because they do not feel it is worth the cost (M)	Data extracted from 10 documents:<br><br>6 research articles (2 qualitative interview studies; 1 cross-sectional studies, 1 quasi-RCT, 1 surveys, 1 systematic review); 2 local evaluation reports; 2 conference materials
CMOC P14	When providers 'buy in' to the NHSHC programme (see CMOC P7 above) (C1) and have adequate time and/or flexibility to deliver each check (C2) they may offer more personalised and in-depth advice and support (O) because they believe these may help attendees (M)	Data extracted from 9 documents:<br><br>2 research articles (1 qualitative interview study, 1 mixed methods); 5 local evaluation reports; 1 conference presentation; 1 practitioner-facing article
Practical constraints: referrals and follow-up		
CMOC P15	When multiple modifiable risk factors are identified during a check (C1) and separate lifestyle services exist for each (C2) providers (and attendees)	Data extracted from 4 documents:

For peer review only

	may agree to prioritise addressing one risk factor first (M) so the delivery of advice, brief interventions and referrals reflect this priority (O)	1 research article (qualitative interview study); 2 local evaluation reports, 1 conference presentation
CMOC P16	When providers don't perceive available lifestyle services to be a good 'fit' for individuals (C) they may avoid making referrals to these services (O) because they do not believe it will help attendees (M)	Data extracted from 4 documents:<br><br>2 research articles (1 retrospective cohort, 1 cross-sectional study); 2 local evaluation reports; 1 PhD thesis (interviews/ethnography)
CMOC P17	When information about local lifestyle services and referral routes is disparate and difficult to access (C) it is harder for providers to make referrals (O) because providers are unaware of available services and how to refer (M)	Data extracted from 9 documents:<br><br>2 research articles (1 qualitative interview study, 1 mixed methods); 4 local evaluation reports; 3 conference materials
CMOC P18	When providers have concerns about the quality of lifestyle support services (C) they may avoid making referrals (O) because they doubt they will help attendees (M)	Data extracted from 3 documents:<br><br>2 research articles (1 survey, 1 Q-methodology study); 1 news article
CMOC P19	When providers have established relationships and referral pathways to trusted lifestyle services (C) they are more likely to offer referrals (O) because this becomes a delivery norm (M)	Data extracted from 12 documents:<br><br>1 research article (mixed methods); 5 local evaluation reports; 6 conference materials
Practical constraints: relationships and confidence in the delivery of advice		

For peer review only

CMOC P20	When providers are concerned that discussion of a particular risk factor may cause offence or upset an attendee (C) they may avoid bringing it up or discussing it in-depth (O) because they lack confidence and want to avoid confrontation (M)	Data extracted from 11 documents:<br><br>6 research articles (3 qualitative interview studies, 1 survey, 1 observational study based on video recordings, 1 fidelity assessment); 2 local evaluation reports; 1 research report (observational study); 1 PhD thesis (ethnography); 1 other report
CMOC P21	When providers lack knowledge about recommendations in relation to a particular risk factor (C) they may avoid bringing it up or discussing it in-depth during a check (O) because they lack confidence in their advice (M)	Data extracted from 4 documents:<br><br>3 research articles (1 qualitative interview study; 1 survey; 1 fidelity assessment); one conference presentation
CMOC P22	When providers perceive that an attendee is unlikely to want to, or be able to change their lifestyle (C) they may avoid giving them advice or offering referrals to support this (O) because they do not think it will help (M1) or because they are worried it could damage their relationship (M2)	Data extracted from 4 documents:<br><br>3 research articles (2 qualitative interview studies; 1 Q-methodology study); 1 local evaluation report
CMOC P23	When providers feel they themselves are not good role models for healthy lifestyles (C) they may be reluctant to deliver advice or brief interventions, or make referrals (O) because they are worried about appearing hypocritical and lacking credibility (M)	Data extracted from 2 documents:<br><br>1 research article (qualitative interview study) and 1 conference presentation

For peer review only

CMOC P24	When providers have lived experience of (trying to) make lifestyle changes (C) they may share this and empathise with attendees (O) because they want to build rapport and a therapeutic alliance during checks (M)	Data extracted from 5 documents:<br><br>3 research articles (2 qualitative interview studies, 1 Q-methodology study); 1 local evaluation report; 1 PhD thesis (ethnography)
CMOC P25	When discussing a risk factor is normalised and routine (C) providers may be more likely to deliver advice, brief interventions and offer referrals related to that risk factor (O) because they feel comfortable and practiced in doing so (M)	Data extracted from 6 documents:<br><br>1 research article (survey); 2 evaluation reports; 3 conference materials
CMOC P26	When providers receive training (C1) or have regular practice (C2) in delivering lifestyle advice, they are more likely to deliver it regularly during checks (O) because they feel more confident (M)	Data extracted from 9 documents:<br><br>3 research articles (1 qualitative interview study, 1 mixed methods, 1 RCT); 1 local evaluation reports; 5 conference materials
CMOC P27	When providers take into account attendees' own priorities, constraints and wishes during a check (C) they may adapt the advice, brief interventions or referrals offered to take these into account (e.g. make fewer but more appropriate referrals) (O) because they share the decision with attendees (M)	Data extracted from 12 documents:<br><br>8 research articles (7 qualitative interview studies, 1 Q-methodology study); 1 local evaluation report; 3 conference materials
CMOC P28	When providers are aware of attendees' own priorities, constraints and wishes (C) they may identify and emphasise the benefits of simple changes	Data extracted 7 from documents:

For peer review only

	that are more acceptable and achievable for attendees (O) because they believe this will help attendees (M)	3 research articles (2 qualitative interview studies, 1 pilot RCT); 1 local evaluation report; 3 conference materials

Table S3 Overview of CMOCs focused on NHSHC attendees

	**CMOC**	**Summary of data**
Understanding and engagement with the NHSHC programme: expectations and priorities		
CMOC A1	When attendees understand the NHSHC as a screening opportunity aimed at identifying individuals with disease (C) they may be less likely to engage with advice, brief interventions or offers of referrals (O) because what is offered does not meet their expectations (M)	Data extracted from 8 documents:<br><br>5 research articles (4 qualitative interview studies, 3 local evaluation reports)
CMOC A2	When attendees are already aware of risk factors and/or potential improvements they could make to their lifestyle (C) they may be more receptive to receiving advice, brief interventions or referrals (O) because they are mentally prepared for it (M)	Data extracted from 15 documents:<br><br>8 research articles (6 qualitative interview studies, 1 ethnography, 1 mixed methods); 4 local evaluation reports; 1 PhD (ethnography); 2 conference materials
CMOC A3	When attendees are unaware that they have risk factors for CVD and receive results that indicate that they are at high risk (C) they may need extra support and information from providers (O) because they are shocked and upset (M)	Data extracted from 6 documents:<br><br>5 research articles (4 qualitative interview studies, 1 ethnography); 1 local evaluation report

For peer review only

CMOC A4	In some circumstances attendees who receive results that indicate that they are at high risk may be prompted to make immediate lifestyle changes (O) because they are shocked and upset (M), but the contexts in which this happens (and for whom) are not clear (C undefined)	Data extracted from 8 documents:<br><br>3 research articles (3 qualitative interview studies); 4 local evaluation reports; 1 conference presentation
CMOC A5	When providers are able to explain the implications of risk factors to attendees in a way they can understand (C) attendees may be more receptive to advice, brief interventions or referrals (O) because they appreciate its importance for their own lives (M)	Data extracted from 6 documents:<br><br>1 research article (qualitative interview study); 1 local evaluation report; 1 PhD thesis (interviews/ethnography); 3 conference materials
CMOC A6	When providers are able to link advice, brief interventions and offers of referrals to attendees' own priorities for their health and lifestyle (C) attendees may be more likely to engage with these (O) because they want to achieve these (M)	Data extracted from 10 documents:<br><br>6 research articles (4 qualitative interview studies, 1 co-production study, 1 observational study using video-recordings); 1 research report (RCT); 1 local evaluation report; 1 PhD thesis (interviews/ethnography); 1 conference poster
CMOC A7	When attendees have multiple risk factors (C) they may choose to focus on the advice, brief interventions or referrals offered in relation to those lifestyle changes that are easier to change (O) because they feel it is better than nothing (M)	Data extracted from 3 documents:<br><br>2 research articles (qualitative interview studies); 1 PhD thesis (interviews/ethnography)

For peer review only

CMOC A8	When attendees feel a personal responsibility for their own health and lifestyle (C) they may be unlikely to take up offers of referrals or ongoing support or follow up (O) because they feel obliged to try to make changes on their own (M)	Data extracted from 3 documents:<br><br>2 research articles (2 qualitative interview studies); 1 local evaluation report
CMOC A9	When attendees are not motivated to change their lifestyle or behaviour (C) they are unlikely to engage with advice, brief interventions or take up offers of referrals to lifestyle services (O) because they do not believe they need to, and have other priorities (M)	Data extracted from 8 documents:<br><br>6 research articles (4 qualitative interview studies, 1 RCT, 1 cross-sectional study); 1 research report (mixed methods); 1 local evaluation report
CMOC A10	When attendees are fatalistic about their health (C) they are unlikely to engage with advice, brief interventions or take up offers of referrals to lifestyle services (O) because they think they are pointless (M)	Data extracted from 8 documents:<br><br>4 research articles (4 qualitative interview studies); 1 research report (mixed methods); 1 local evaluation report; 1 conference presentation
CMOC A11	When attendees have health concerns and priorities that fall outside the remit of the NHSHC programme (C) they may be disappointed with the check (O1) and unlikely to engage with advice, brief interventions or take up offers of referrals to lifestyle services (O2) because these do not feel important to them, and they have other priorities (M)	Data extracted from 5 documents:<br><br>2 research articles (2 qualitative interview studies); 1 local evaluation report; 2 PhD theses (ethography, interviews/ethnography).

For peer review only

CMOC A12	When attendees receive an 'opportunistic' check (C) they are less likely to (receive and) engage with advice, brief interventions or offers of referrals (O) because they do not understand that this is the purpose of the check (M)	Data extracted from 6 documents:<br><br>3 research articles (3 qualitative interview studies); 2 local evaluation reports; 1 guidance document
CMOC A13	When attendees have the opportunity and time to discuss and ask questions during a check (C) they may be more likely to receive and engage with advice, brief interventions and offers of referrals (O) because they understand that this is the purpose of the check (M)	Data extracted from 14 documents:<br><br>5 research articles (2 qualitative interview studies, 1 ethnography, 1 mixed methods study, 1 observational study using video-recordings); 6 local evaluation reports; 1 research report (mixed methods); 1 PhD thesis (interviews/ethnography)
CMOC A14	When checks are delivered in a non-medical setting (i.e. not in general practice) (C) attendees may be more likely to engage in discussions about risk factors and lifestyle change (O) because they feel relaxed and comfortable (M)	Data extracted from 8 documents:<br><br>3 research articles (1 qualitative interview study, 1 ethnography, 1 protocol for a mixed methods study); 1 local evaluation report; 4 conference materials
Understanding and engagement with the NHSHC programme: credibility and trust		
CMOC A15	When attendees receive advice, brief interventions, offers of referral or prescriptions from a professional they consider to be suitably qualified (C) they may be more likely to engage with or accept these interventions (O) because they consider them to be credible (M)	Data extracted from 10 documents:<br><br>7 research articles (3 qualitative interview studies, 2 surveys, 2 mixed methods studies); 2 local evaluation reports; 1 PhD thesis (interviews/ethnography).

For peer review only

CMOC A16	When attendees receive advice, brief interventions or offers of referral from a provider who seems to understand their circumstances (C) they may be more likely to engage with these interventions (O) because they consider them to be credible (M)	Data extracted from 5 documents:<br><br>1 research article (ethnography); 1 research report (RCT); 1 local evaluation report; 1 conference poster; 1 news article
CMOC A17	When providers can deliver culturally appropriate lifestyle advice (C) attendees may be more likely to engage with it (O) because they consider it to be more credible and relevant (M)	Data extracted from 4 documents:<br><br>1 research article (ethnography); 1 research report (RCT); 1 local evaluation report; 1 conference abstract
CMOC A18	When the measurements and risk assessment completed during a check identify attendees as 'low risk' (C) attendees may be less receptive to any advice, brief interventions or referrals offered (O) because they are reassured and do not think they need to take action (O)	Data extracted from 4 documents:<br><br>3 research articles (1 qualitative interview study, 1 survey, 1 mixed methods study); 1 local evaluation report
CMOC A19	When providers downplay risks or temper advice about lifestyle (C) attendees may feel there is no need to make any changes (O) because they are reassured (M)	Data extracted from 5 documents:<br><br>4 research articles (4 qualitative interview studies); 1 local evaluation report
CMOC A20	When providers are able to convey the importance and urgency of NHSHC risk assessments to attendees (C) attendees may be more receptive to advice, brief interventions or referrals (O) because they feel important (M)	Data extracted from 6 documents:<br><br>3 research articles (3 qualitative interview studies); 1 local evaluation report; 1 PhD thesis (interviews/ethnography); 1 conference presentation

For peer review only

CMOC A21	When the measurements and risk assessment completed during a check identify attendees as 'low risk' (C) attendees' healthy lifestyle habits may be reinforced (O) because they understand these have tangible benefits, reflected in their results (M)	Data extracted from 3 documents:<br><br>2 research articles (1 qualitative interview study, 1 ethnography); 1 PhD thesis (interviews/ethnography)
CMOC A22	When attendees receive measurements and risk assessment results from non-professionals (C1) or do not receive results at all (C2) they may be less likely to consider the results to be important (O) because they trust that providers will alert them to significant results that require action (M)	Data extracted from 4 documents:<br><br>3 research articles (2 qualitative interview studies, 1 survey); 1 PhD thesis (interviews/ethnography)
CMOC A23	When attendees perceive providers to be disengaged with the check (C) they may be less likely to engage with advice, brief interventions or offers of referral (O) because they do not believe these are important (M)	Data extracted from 4 documents:<br><br>2 research articles (1 ethnography, 1 survey); 1 PhD thesis (ethnography); 1 conference presentation
CMOC A24	When attendees receive advice, brief interventions or offers of referral from a provider who does not seem to 'practice what they preach' (C) they may be less likely to engage with these interventions (O) because they consider these to lack credibility (M)	Data extracted from 2 documents:<br><br>1 research article (qualitative interview study); 1 local evaluation report
CMOC A25	When attendees receive advice or information relating to healthy lifestyles that they already familiar with during a check (C) they may perceive the advice to be useless (O) because they have heard it before (M)	Data extracted from 8 documents:<br><br>6 research articles (4 qualitative interview studies, 1 ethnography, 1 mixed methods study); 2 PhD theses (ethnography, interviews/ethnography)

For peer review only

CMOC A26	When attendees learn something new and important to them during a check (C) they may attempt to make changes to their lifestyle in light of this (O) because they have an improved understanding of risk factors or lifestyle advice (M)	Data extracted from 6 documents:<br><br>3 research articles (1 qualitative interview study, 1 ethnography, 1 survey); 1 research report (RCT); 1 local evaluation report; 1 PhD thesis (interviews/ethnography)
CMOC A27	When attendees are aware of conflicting or inconsistent guidance about healthy lifestyles (C) they may reject or ignore advice delivered during a check (O) because they doubt its credibility (M1) or are confused (M2)	Data extracted from 4 documents:<br><br>2 research articles (2 qualitative interview studies); 1 research report (RCT); 1 PhD thesis (ethnography)
CMOC A28	When attendees are aware of controversy in relation to recommended medication (statins) (C) they may be ambivalent about accepting or adhering to a prescription (O) because they are uncertain of the benefits (M)	Data extracted from 5 documents:<br><br>3 research articles (3 qualitative interview studies; 1 local evaluation reports; 1 conference presentation
CMOC A29	When attendees have doubts about the accuracy of the tests and tools used to measure and assess risk during a check (C) they may be less receptive to advice, brief interventions, referrals or prescriptions offered to address identified risks (O) because they are unsure that the assessments are credible (M)	Data extracted from 6 documents:<br><br>2 research articles (1 qualitative interview study, 1 mixed methods study); 1 research report (mixed methods study); 2 local evaluation reports; 1 PhD thesis (interviews/ethnography)
Practical constraints: referrals and follow-up		

For peer review only

CMOC A30	When attendees receive continuity of care and follow up after a check (C) they may be more likely to engage with advice, take up referrals or attempt to make lifestyle changes (O) because they are reminded of what they are meant to be doing and why (M)	Data extracted from 10 documents:<br><br>5 research articles (4 qualitative interview studies, 1 pilot RCT); 1 research report (RCT); 2 local evaluation reports; 1 PhD thesis (interviews/ethnography); 1 conference poster
CMOC A31	When attendees receive continuity of care and follow up after a check (C) they may be more likely to engage with advice, take up referrals or attempt to make lifestyle changes (O) because they feel supported and valued (M)	Data extracted from 5 documents:<br><br>2 research articles (2 qualitative interview studies); 1 research reports (RCT); 1 local evaluation report; 1 PhD thesis (ethnography)
CMOC A32	When attendees are repeatedly offered follow up and feedback on progress after a check (C) they may be motivated to attempt and maintain lifestyle changes (O) because they can monitor their progress (M)	Data extracted from 6 documents:<br><br>3 research articles (3 qualitative interview studies); 1 research report (RCT); 2 local evaluation reports
CMOC A33	When attendees can see tangible benefits of making lifestyle changes after a check (C) they are more likely to maintain these (O) because they are motivated to continue (M)	Data extracted from 5 documents:<br><br>1 research article (qualitative interview study); 2 local evaluation reports; 2 conference materials

For peer review only

CMOC A34	When attendees are not offered any follow up (beyond the five-year NHSHC programme cycle) (C) they may lack motivation to attempt any lifestyle changes (O) because they interpret the absence of follow up to mean there is no urgent need to make changes (M)	Data extracted from 3 documents:<br><br>1 research article (survey); 1 research report (RCT); 1 PhD thesis (interviews/ethnography)
CMOC A35	When attendees don't consider local lifestyle services to be convenient, appropriate or likely to meet their needs (C) they are less likely to take up referrals or attend these services (O) because they feel it is pointless (M)	Data extracted from 14 documents:<br><br>9 research articles (7 qualitative interviews studies, 1 systematic review, 1 mixed methods study); 2 local evaluation reports; 2 PhD theses (ethnography, interviews/ethnography); 1 conference abstract
CMOC A36	When local lifestyle support services are designed to be more accessible (e.g. in terms of timing, location, cost) (C) attendees may be more likely to start and continue to attend (O) because they feel they are more convenient, affordable or relevant (M)	Data extracted from 4 documents:<br><br>3 research articles (3 qualitative interview studies); 1 conference poster
CMOC A37	When attendees have the option to try out a lifestyle service or are supported to try one by a provider (C) they may be more likely to take up an offer of a referral (O) because they feel more confident to do so (M)	Data extracted from 3 documents:<br><br>1 research article (pilot RCT); 1 local evaluation report; 1 conference abstract
Practical constraints for attendees: person-centredness		

For peer review only

CMOC A38	When attendees receive advice about healthy lifestyles that does not take account of their personal circumstances (C) they are less likely to engage with it (O) because they believe it is unworkable for them (M)	Data extracted from 10 documents:<br><br>8 research articles (7 qualitative interview studies, 1 mixed methods study); 1 PhD thesis (ethnography); 1 conference presentation
CMOC A39	When attendees receive advice about healthy lifestyles that they believe they cannot achieve (e.g. because it seems to require big changes) (C) they are less likely to engage with it (O) because they feel overwhelmed and hopeless (M)	Data extracted from 3 documents:<br><br>3 research articles (2 qualitative interview studies, 1 observational study using video recordings)
CMOC A40	When attendees receive advice about healthy lifestyles that attendees feel they can fit into their lives (e.g. around other commitments) (C) they may be more likely to engage with it (O) because they perceive the changes to be less disruptive (M)	Data extracted from 13 documents:<br><br>6 research articles (4 qualitative interview studies, 1 ethnography, 1 pilot RCT); 1 research report (RCT); 5 local evaluation reports; 1 conference poster
CMOC A41	When attendees feel that significant lifestyle change is unworkable for them (C) they may be more likely to accept prescriptions (e.g. for statins) (O) because they still want to do something to reduce their CVD risk (M)	Data extracted from 2 documents:<br><br>1 research article (qualitative interview study); 1 local evaluation report
CMOC A42	When attendees anticipate or experience medication side effects or burdens (C) they may be more ambivalent about accepting or adhering to prescriptions (O) because they are uncertain of the benefits and concerned about harms (M)	Data extracted from 4 documents:<br><br>2 research articles (2 qualitative interview studies); 1 local evaluation report; 1 conference presentation

For peer review only

CMOC A43	When attendees anticipate or experience medication side effects or burdens (C) they attempt lifestyle change (O) because they prefer this option (M)	Data extracted from 3 documents:<br><br>2 research articles (2 qualitative interview studies); 1 local evaluation report
CMOC A44	When attendees are encouraged and supported by friends, family or peers to make and sustain lifestyle changes (C) they may be more likely to attempt and maintain changes (O) but the mechanism for this is unclear (M not defined)	Data extracted from 11 documents:<br><br>7 research articles (5 qualitative interview studies, 1 ethnography, 1 co-production study); 1 research report (RCT); 1 local evaluation report; 2 PhD theses (ethnography, interviews/ethnography)

For peer review only
